# Discovery of spirooxindole-derived small-molecule compounds as novel HDAC/MDM2 dual inhibitors and investigation of their anticancer activity

**DOI:** 10.3389/fonc.2022.972372

**Published:** 2022-08-04

**Authors:** Qian Zhao, Shan-Shan Xiong, Can Chen, Hong-Ping Zhu, Xin Xie, Cheng Peng, Gu He, Bo Han

**Affiliations:** ^1^ State Key Laboratory of Southwestern Chinese Medicine Resources, Hospital of Chengdu University of Traditional Chinese Medicine, School of Basic Medical Sciences, Chengdu University of Traditional Chinese Medicine, Chengdu, China; ^2^ Department of Dermatology and State Key Laboratory of Biotherapy, West China Hospital, Sichuan University, Chengdu, China; ^3^ School of Pharmacy, Chengdu Medical College, Chengdu, China; ^4^ The First Affiliated Hospital, Chengdu Medical College, Chengdu, China; ^5^ Antibiotics Research and Re-evaluation Key Laboratory of Sichuan Province, Sichuan Industrial Institute of Antibiotics, Chengdu University, Chengdu, China

**Keywords:** multitarget drugs, histone deacetylase inhibitors, MDM2 inhibitors, spirooxindole, dual inhibitors, anticancer

## Abstract

Simultaneous inhibition of more than one target is considered to be a novel strategy in cancer therapy. Owing to the importance of histone deacetylases (HDACs) and p53-murine double minute 2 (MDM2) interaction in tumor development and their synergistic effects, a series of MDM2/HDAC bifunctional small-molecule inhibitors were rationally designed and synthesized by incorporating an HDAC pharmacophore into spirooxindole skeletons. These compounds exhibited good inhibitory activities against both targets. In particular, compound 11b was demonstrated to be most potent for MDM2 and HDAC, reaching the enzyme inhibition of 68% and 79%, respectively. Compound 11b also showed efficient antiproliferative activity towards MCF-7 cells with better potency than the reference drug SAHA and Nutlin-3. Furthermore, western blot analysis revealed that compound 11b increased the expression of p53 and Ac-H4 in MCF-7 cells in a dose-dependent manner. Our results indicate that dual inhibition of HDAC and MDM2 may provide a novel and efficient strategy for the discovery of antitumor drug in the future.

## 1 Introduction

Over the past few decades, drug discovery always relies on the “single target single drug” model, and numerous drugs that act on a specific biological target with high potency and selectivity have been approved successfully (1–4). However, for the treatment of some heterogeneous, complex, and multigenic diseases such as cancer, single target chemotherapeutic agents might become less effective (5, 6). The main reason is that the tumor progression always involves the dysregulation of multiple signaling pathways due to their multifaceted and dynamic nature, which hinders the efficacy of single-target drugs, and even leads to drug resistance and toxic side effects after a period of treatment (7–12). Despite the fact that specific drug combination therapies are employed as an alternative method, they are often limited by many side effects, such as drug-drug interactions and poor patient compliance (3, 13). Compared with the traditional one-drug/one-target or drug combinations philosophy, multitarget antitumor agents could simultaneously regulate two or more relevant targets, thus enabling a synergic therapeutic efficacy and fewer side effects (3, 14, 15). In this context, the discovery of multitarget drugs that combine two distinct biologically active molecules or pharmacophores has drawn much more attention.

**Figure 1:**
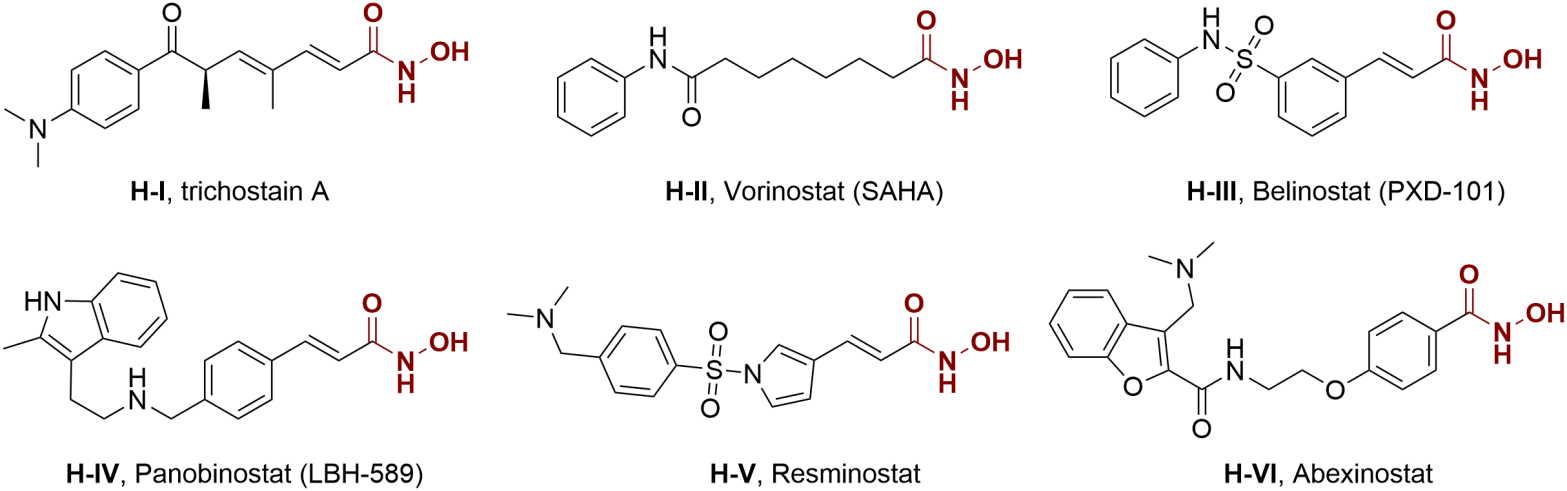
Structures of several representative HDAC inhibitors.

Actually, genetic and epigenetic aberrations have an evident influence on tumorigenesis and development, but the latter is more druggable because of its reversibility modulated by numerous enzymes (16, 17). Histone deacetylases (HDACs) represent a class of epigenetic enzymes that regulate the acetylation balance of histone proteins, are important regulatory factors in many crucial cellular functions, including angiogenesis, cell differentiation, cell proliferation, and cell apoptosis (18–23). The overexpression of HDACs can be observed in tumor initiation and proliferation, and thus they are regarded as potential epigenetic targets for cancer therapy. In 1990, Yoshida et al. reported the first potent HDAC inhibitor trichostatin A (H-I) (24). Since then, several HDAC inhibitors, such as vorinostat (SAHA, H-II), belinostat (PXD-101, H-III), and panobinostat (LBH-589, H-IV), have been approved for the treatment of hematological malignancy (25), while many others are currently in clinical trials for oncology (**Figure 1**, H-V and H-VI) (26–30). Despite the progress in hematologic tumor treatment, the therapeutic effects of some HDAC inhibitors and epigenetic agents are not significant against solid tumors. Considering the crucial role of HDAC in various cellular pathways and the above-mentioned advantages of multitarget antitumor drugs, intense interest is attracted to the discovery of HDAC inhibitor-based hybrid molecules (31–40).

**Figure 2:**
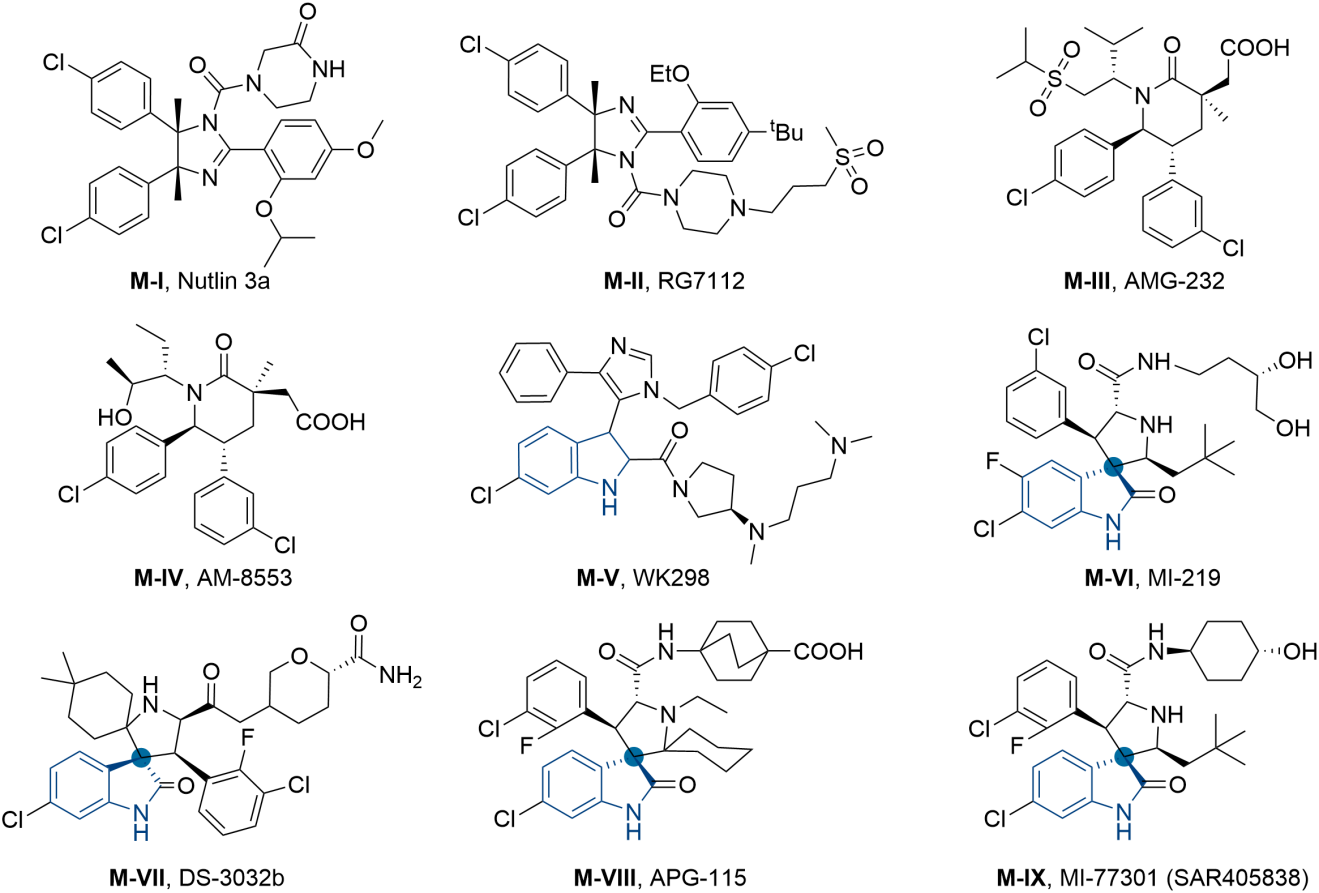
Structures of several representative MDM2 inhibitors.

On the other hand, as a “guardian of the genome”, tumor suppressor protein p53 also played a pivotal role in regulating cell cascade reactions. Currently, approximately 50% of human tumors are related to the deletion or mutation of p53, whereas others exhibited wild-type status and lost p53 functions by negative regulation of murine double-minute 2 (MDM2) protein (41, 42). In general, MDM2 protein can directly bound to the *N*-terminal transactivation domain of p53 resulting in the disappearance of transcription function or proteasomal degradation (43–46). Therefore, inhibiting the interaction of p53-MDM2 can reactivate the p53 pathway and emerge as a prospective therapeutic method for cancers. Up to now, a number of small-molecule MDM2 inhibitors have been developed, and some of them even entered preclinical or clinical trials for the treatment of multiple tumors (**Figure 2**), such as RG7122 (M-II), AMG-232 (M-III), APG-115 (M-VIII), MI-77301 (M-IX) and so on (47–52). However, the main side effect of p53-MDM2 inhibitors was thrombocytopenia and gastrointestinal toxicity, which was associated with dosage (53–55). In an effort to improve their efficacy, reducing the dose becomes an efficient way to avoid related toxicities. Therefore, modulation of multiple related targets to generate superior efficacy by hybrid molecule (single compound) is highly desirable. Our recent works and other studies have successfully demonstrated the effectiveness of MDM2-based dual inhibitors, like MDM2-GPX4, MDM2-CDK4, MDM2-XIAP, MDM2-MDM4, and so on (56–63).

Actually, p53 acetylation is one of the mechanisms for activating its function, previous studies have demonstrated that HDAC inhibitors can induce the acetylation of carboxyl-terminal lysine residue site Lys382 of p53 and activate p53 target genes (64). Therefore, inhibiting HDACs and MDM2 protein are both effective strategies to lead to the accumulation of activated p53. In addition, HDAC inhibitors could synergize with MDM2 inhibitors and enhance their antitumor activity by modifying the hyperacetylation of p53 (18, 65). These results indicated that rational design and synthesis of a single compound simultaneously inhibiting HDAC and MDM2 targets to exert desired biological functions would be an efficient strategy for developing novel antitumor agents.

**Figure 3:**
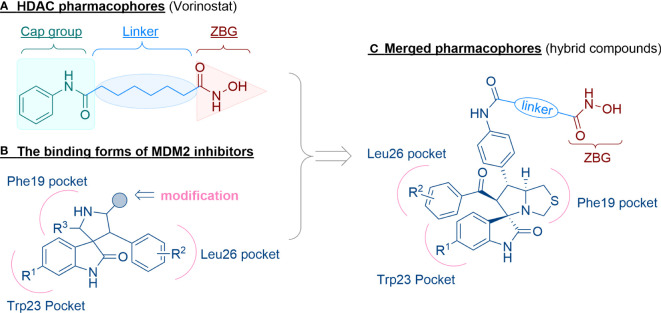
Design of novel HDAC/MDM2 dual inhibitors via merging pharmacophores. **(A)** HDAC pharmacophores; **(B)** The binding forms of MDM2 inhibitor; **(C)** Merged pharmacophores.

The general pharmacophore of most HDAC inhibitors, including Vorinostat, consists of three parts (**Figure 3A**): the hydrophobic cap group (CAP) that recognizes the protein surface and prevents other substrates from entering the active structure, a linker occupying the long tunnel of the active site, and the zinc-binding group (ZBG) that chelates with Zn^2+^ and forms hydrogen bonds in the catalytic site (66–68). More importantly, the cap group of HDAC inhibitors can be replaced by other chemical stents (or pharmacophore) to obtain new types of multi-targeted compounds. In 2018, Sheng and co-workers reported the first HDAC-MDM2 dual inhibitors with excellent anticancer activities *in vitro* and *in vivo* based on Nutlin-3a (M-I, MDM2 inhibitor) (69). Considering that spirooxindole skeletons have been well established as MDM2 inhibitors (**Figure 2**, M-VI−IX) (70–72) and encouraged by the pioneering work of Sheng’s group, we rationally designed new classes of hybrid compounds derived from spirooxindole scaffolds that could inhibit both HDAC as well as MDM2. According to the binding models of most spirooxindole derivatives and MDM2 target, three functional groups on the spirooxindole framework were assumed to occupy the Phe19, Trp23, and Leu26 pockets in p53, respectively (**Figure 3B**). As a result of the C3’ substitution in the spiro core of some inhibitors did not bury inside to MDM2 protein and was directly exposed to the solvent, this position may become appropriate to induce a hydrophobic linker and ZBG to generate HDAC-MDM2 dual inhibitors. As part of our continuing interest in assembling biologically important frameworks as lead compounds for drug discovery (73–78), we herein synthesized a series of spirooxindole-based dual HDAC-MDM2 small-molecule compounds, and evaluated their antitumor activity together with the mechanism of action (**Figure 3C**).

## 2 Results and discussion

### 2.1 Chemistry

**Figure 4:**
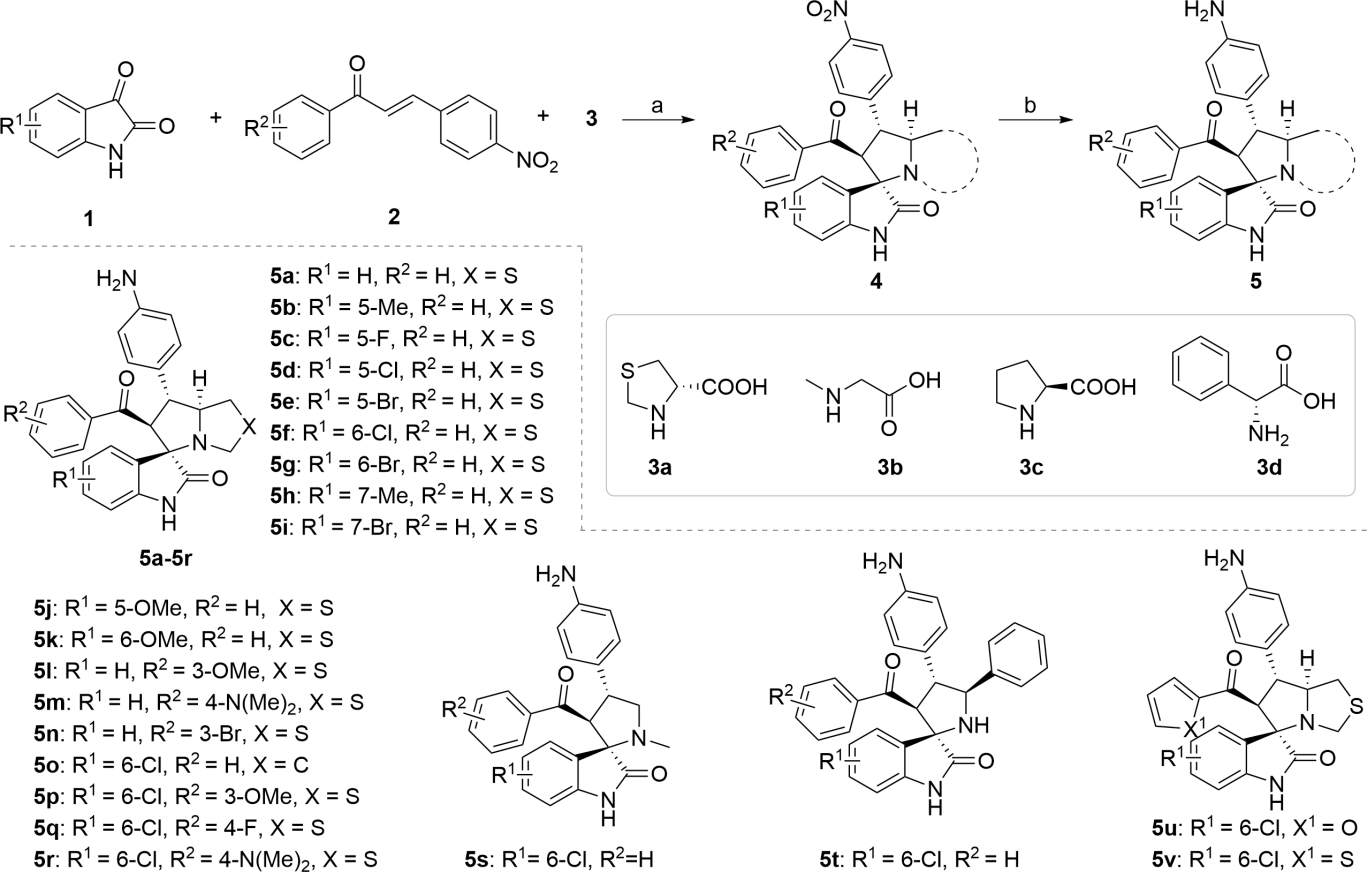
Synthesis of compounds **5a-5v**. Reagents and conditions: (a) **1** (1.0 equiv), **2** (1.0 equiv), **3** (1.0 equiv), MeOH, 65 °C, 4 h; (b) Fe (10.0 equiv), NH4Cl (5.0 equiv), MeOH/H2O = 2:1, 60 °C, 6 h.

**Figure 5:**
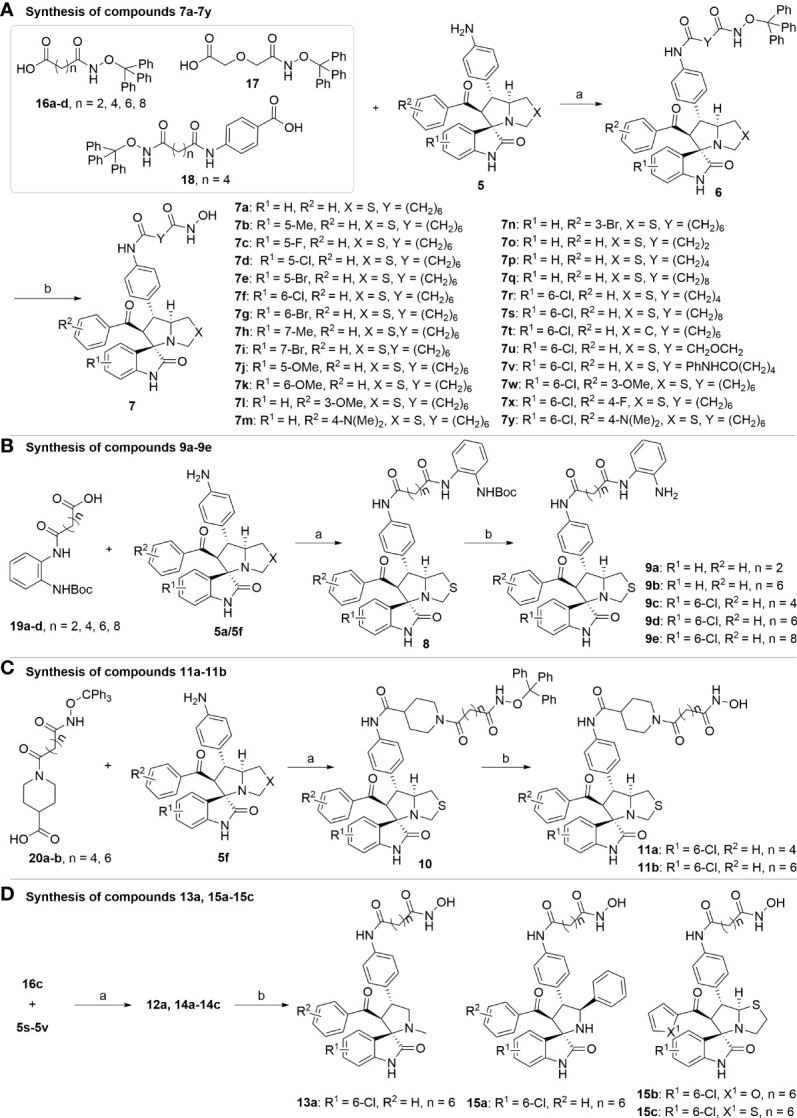
Synthesis of target compounds. **(A)** Synthesis of compounds **7a-7y**; **(B)** Synthesis of compounds **9a-9e**; **(C)** Synthesis of compounds **11a-11b**; **(D)** Synthesis of compounds **13a, 15a-15c**. Reagents and conditions: (a) HATU (1.2 equiv), DIPEA (2.0 equiv), DCM, rt; (b) TFA/DCM=1:3, rt.

The synthetic methods of target compounds are depicted in **Figures 4**, **5**. Compounds 5a-5v were prepared according to the procedure detailed in **Figure 4**. Using commercially available indirubin 1, 4-nitrochalcone derivatives 2 as the starting material to react with amino acids (thioproline 3a, sarcosine 3b, proline 3c, and phenylglycine 3d) in methanol at 65°C, a series of spirooxindole pyrrolidine, spirooxindole thiopyrrole, and their derivatives were obtained through dipolar cycloaddition reaction. Then compound 4a-4v was treated with iron powder, NH_4_Cl in MeOH/H_2_O at 60°C to produce intermediates 5a-5v.

With the key spirooxindole intermediates 5a-5v in hand, we turned our attention to introduce HDAC pharmacophore. Thinking that 2-aminoaniline and hydroxamic acid are commonly used as Zn^2+^ binding groups (ZBG) in HDAC inhibitors, we subsequently introduce different linkers. Spirooxindole intermediates 5 were reacted with 16 to generate the corresponding derivatives 6a-6y (**Figure 5A**), which was followed by hydrolysis in the presence of triethylamine could finally afford the target HDAC-MDM2 dual inhibitors 7a-7y (**Figure 5A**). Target compounds 9a-9e, 11a-11b, 13a, 15a-15c were also prepared according to the above-mentioned methods (**Figures 5B–D**).

### 2.2 Biological Evaluation

#### 2.2.1 Enzyme Inhibitory Activities

Having synthesized a series of novel spirooxindole-based derivatives, we turn to evaluate their inhibitory activities against HDAC and MDM2 at 1 μM, the results are summarized in **Tables 1**–**4**. Given the fact that hydroxamic acid groups are prevalent in HDAC inhibitors, and can strongly chelate with zinc ions (79–84), most of the target compounds are fixed with hydroxamic acid as a ZBG to the side chain.

**Table 1:** Effects of substituted group R^1^ and R^2^ on enzyme inhibition.

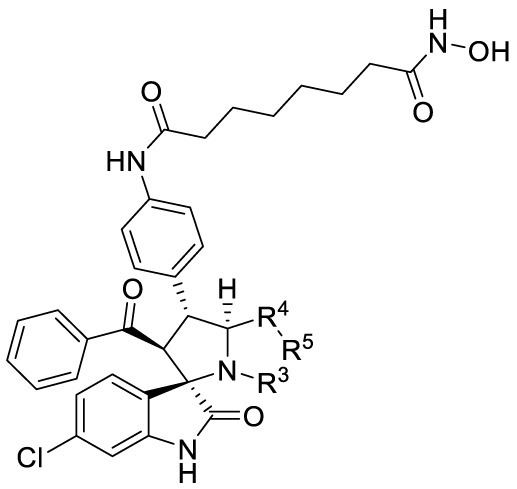				
compound	R^1^	R^2^	% enzyme inhibition	
			MDM2	HDAC
**7a**	H	Ph	39	69
**7b**	5-Me	Ph	45	71
**7c**	5-F	Ph	51	68
**7d**	5-Cl	Ph	49	67
**7e**	5-Br	Ph	48	72
**7f**	6-Cl	Ph	65	64
**7g**	6-Br	Ph	59	68
**7h**	7-Me	Ph	23	71
**7i**	7-Br	Ph	30	67
**7j**	5-OMe	Ph	50	70
**7k**	6-OMe	Ph	54	70
**7l**	H	3-OMe-C_6_H_4_	44	62
**7m**	H	4-NMe2-C_6_H_4_	40	68
**7n**	H	3-Br-C_6_H_4_	43	69
**7w**	6-Cl	3-OMe-C_6_H_4_	60	69
**7x**	6-Cl	4-F-C_6_H_4_	62	73
**7y**	6-Cl	4-NMe_2_-C_6_H_4_	54	71
**15b**	6-Cl	2-furyl	48	70
**15c**	6-Cl	2-thienyl	49	70

**Table 2:** Effects of amino acids 3 on enzyme inhibition.

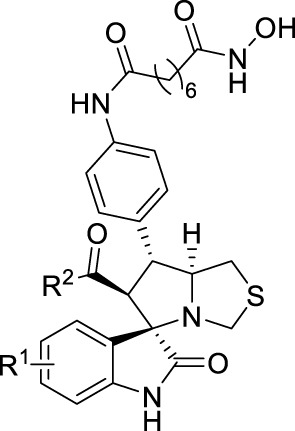					
compound	R^3^	R^4^	R^5^	% enzyme inhibition	
				MDM2	HDAC
**7f**	C	C	S	65	64
**7t**	C	C	C	61	61
**13a**	C	H	/	52	65
**15a**	H	Ph	/	67	61

**Table 3:** Effects of ZBG on enzyme inhibition.

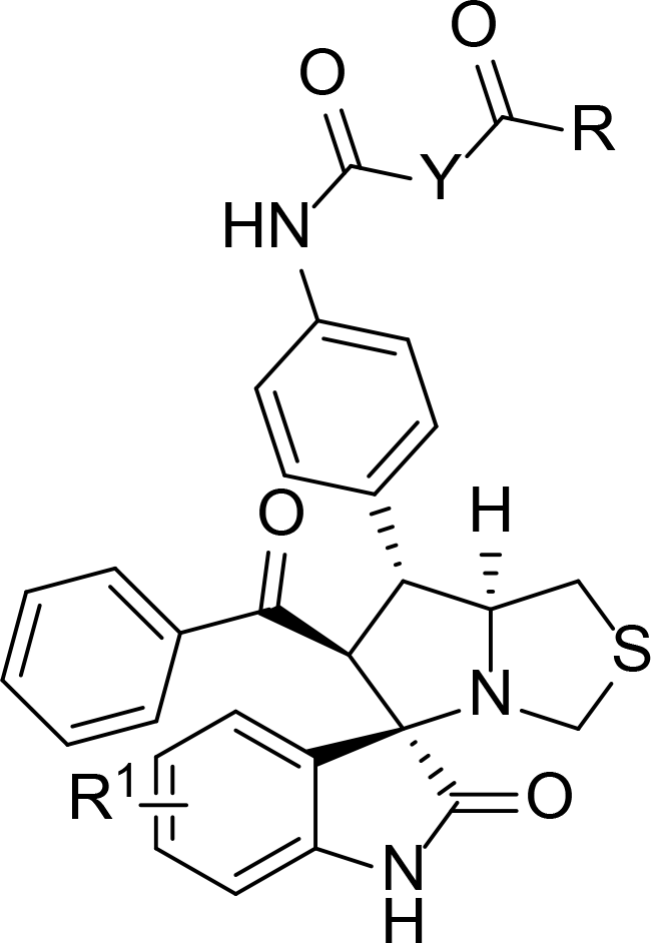					
compound	R	R^1^	Y	% enzyme inhibition	
				MDM2	HDAC
**7o**	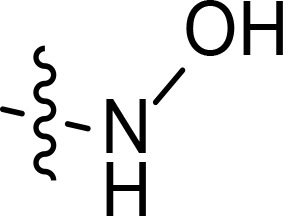	H	(CH_2_)_2_	43	54
**9a**	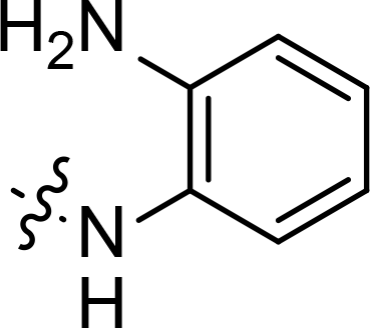	H	(CH_2_)_2_	50	37
**7a**	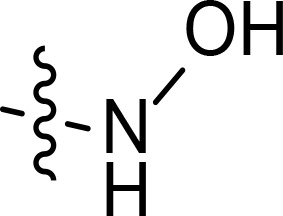	H	(CH_2_)_6_	39	69
**9b**	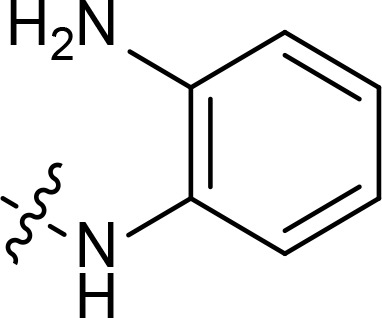	H	(CH_2_)_6_	45	45
**7r**	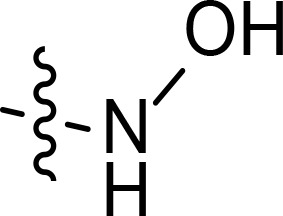	6-Cl	(CH_2_)_4_	61	55
**9c**	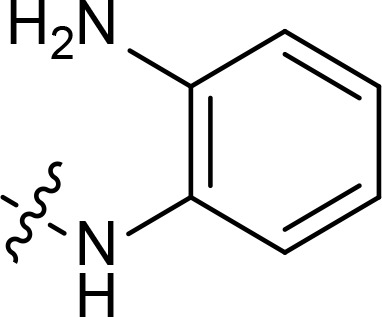	6-Cl	(CH_2_)_4_	57	40
**7d**	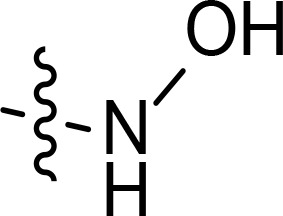	6-Cl	(CH_2_)_6_	49	67
**9d**	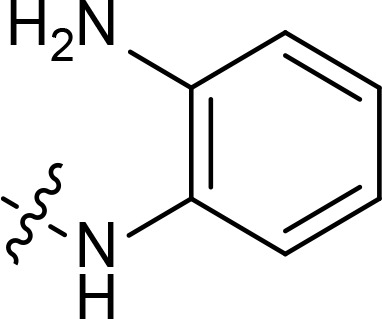	6-Cl	(CH_2_)_6_	56	55
**7s**	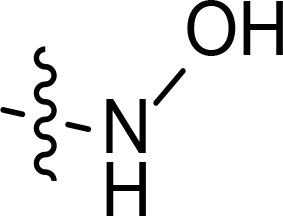	6-Cl	(CH_2_)_8_	59	68
**9e**	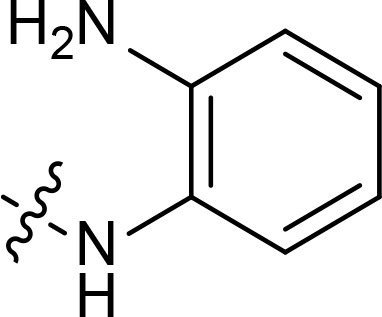	6-Cl	(CH_2_)_8_	56	47

**Table 4:** Effects of linkers on enzyme inhibition.

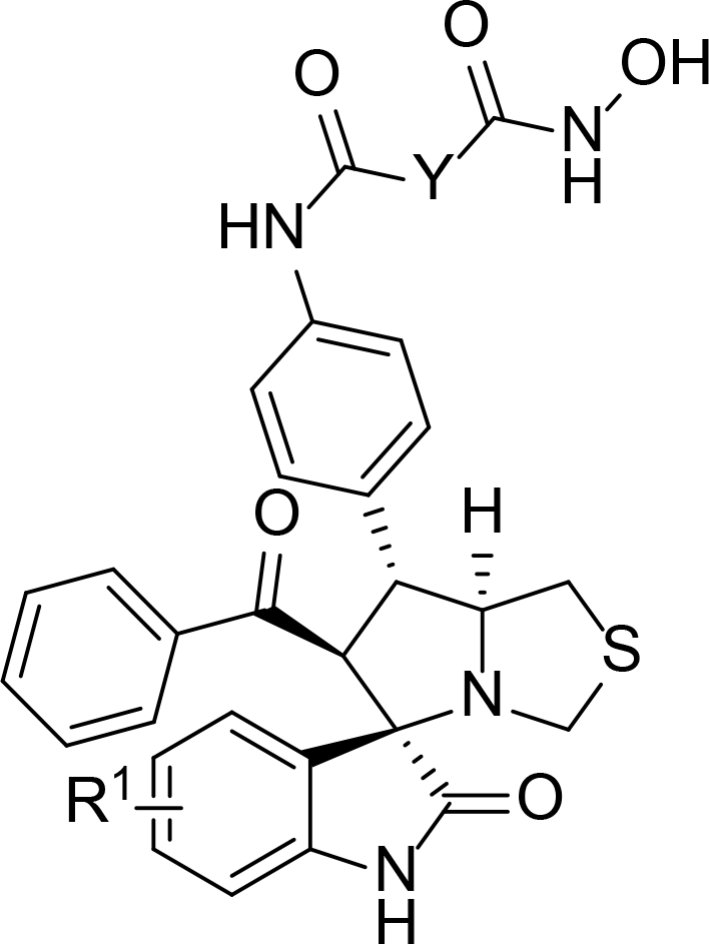				
compound	R^1^	Y	% enzyme inhibition	
			MDM2	HDAC
**7o**	H	(CH_2_)_2_	43	54
**7p**	H	(CH_2_)_4_	44	60
**7a**	H	(CH_2_)_6_	39	69
**7q**	H	(CH_2_)_8_	42	71
**7r**	6-Cl	(CH_2_)_4_	61	55
**7f**	6-Cl	(CH_2_)_6_	65	64
**7s**	6-Cl	(CH_2_)_8_	59	68
**7u**	6-Cl	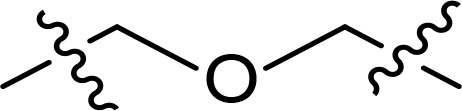	61	54
**7v**	6-Cl	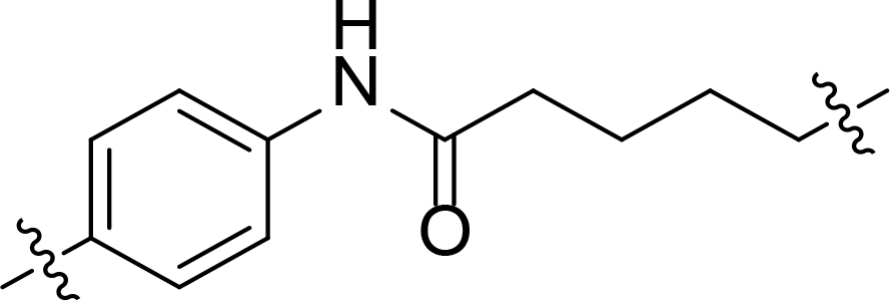	58	53
**11a**	6-Cl	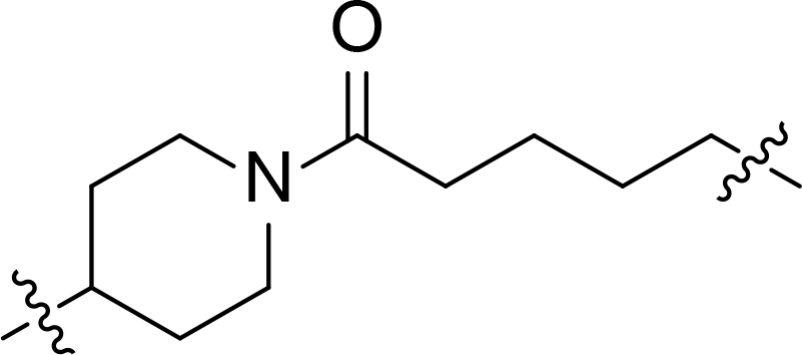	62	70
**11b**	6-Cl	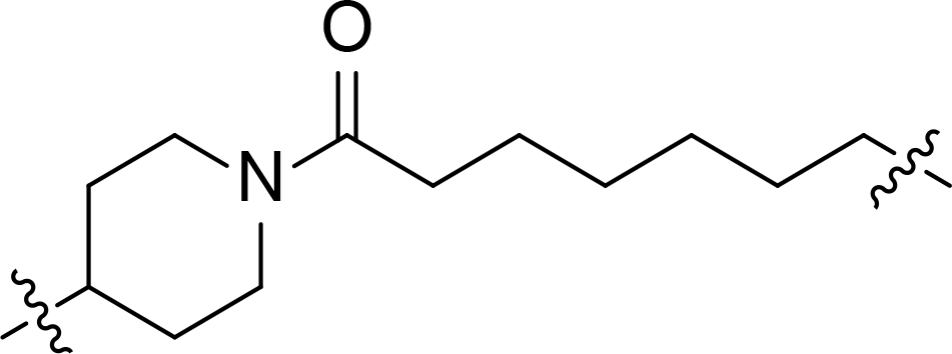	68	79

As shown in **Table 1**, we found that the variation of substituted groups at isatin scaffold has a strong influence on their MDM2 inhibition activity (7a-7k), with a chlorine atom at the C6 position exhibiting relatively good inhibitory activity against MDM2 (7f, 65%). On the other hand, various substituted group R^2^ on substrate 2 were also investigated, which did not enhance the inhibitory activity against MDM2 (7l-7y), some of them even decreased the corresponding activities on MDM2 target (15b and 15c). In fact, compounds 7w-7y, simultaneously substituted with chlorine moiety at the R^1^ position, exhibited better MDM2 inhibitory activity than those with R^1^ = H, such as 7l-7n. Moreover, the evaluation of amino acids demonstrated that thioproline-containing compound 7f exhibits the best activity, reaching the inhibition ratio of 65% and 64% toward MDM2 and HDAC, respectively (**Table 2**).

We also introduced *ortho*-aminoanilide as ZBG, however, compounds 9a-9e caused a remarkable decrease of activity against HDAC compared to 7a, 7o, 7r, 7d, 7s fixed with hydroxamic acid as ZBG (**Table 3**). Considering the importance of the ZBG on dual inhibitory activity, a SAR study on linkers was performed, as shown in **Table 4**. In general, varying the linker length significantly influences the potency, with a longer alkyl chain leading to improved activity against HDAC. It is worth mentioning that introducing oxygen atom, and benzene ring to the alkyl chain led to a decrease in HDAC inhibition efficacy (7u and 7v), but further incorporation of the piperidine ring into the linker was beneficial for the HDAC inhibitory potency (11a and 11b).

#### 2.2.2 *In Vitro* Antiproliferative Assay

**Figure 6:**
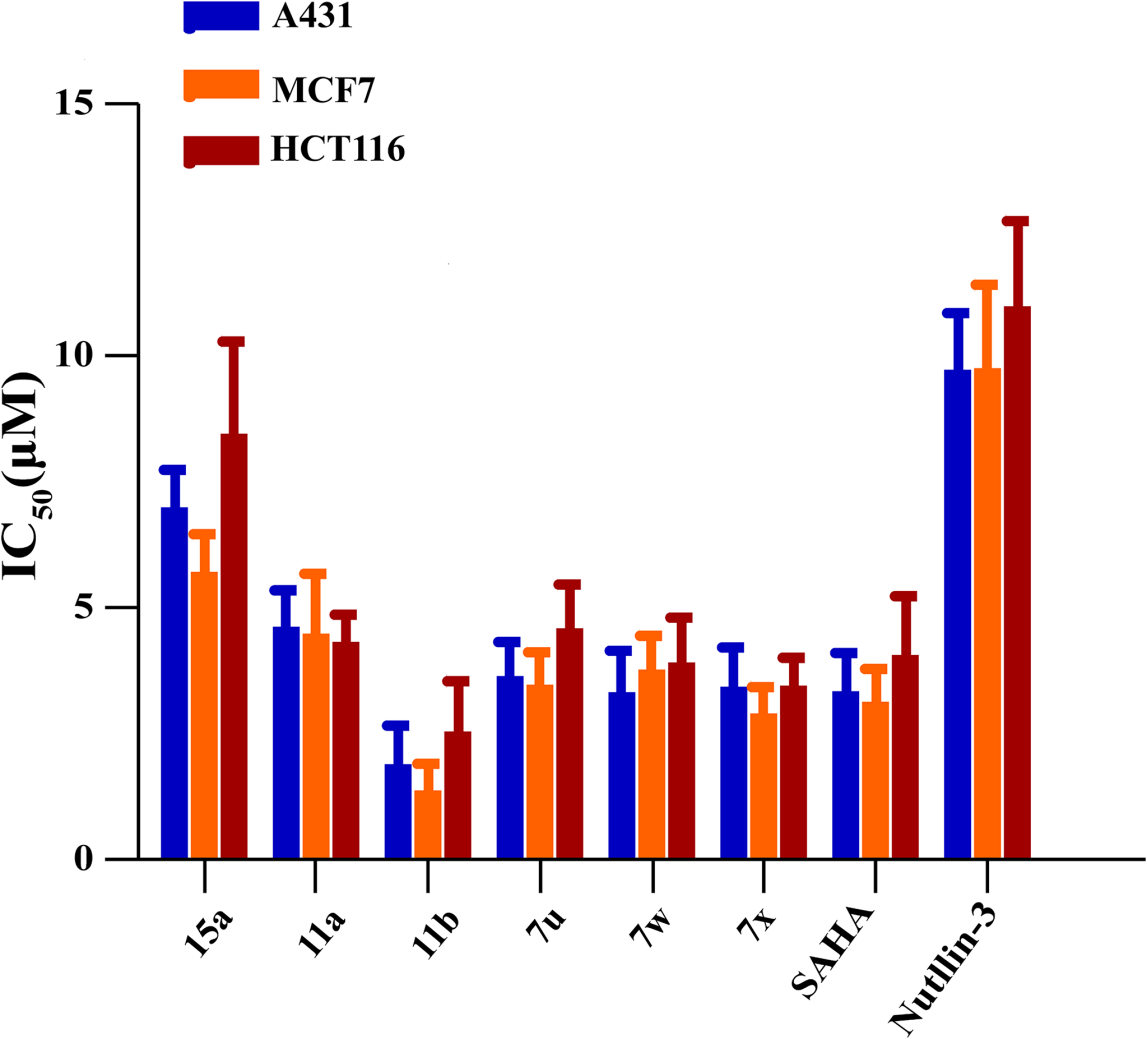
Potential binding modes of 11b to HDAC. **(A, B)** the 3D diagram of the binding modes; **(C)** 2D diagram of the binding modes.

Considering their potent enzyme inhibitory potencies, six MDM2-HDAC dual inhibitors (7u, 7w, 7x, 11a, 11b, and 15a) were selected to evaluate for their antiproliferative activities against A431 (human epidermal carcinoma), MCF-7 (human breast cancer) and HCT116 (human colorectal cancer) cell lines by MTT assay, with the hydroxamic acid-based compound SAHA and Nutlin-3 as positive controls. According to the results in **Table 5** and **Figure 6**, most compounds showed good inhibition activities against the three human cancer cells, they were more effective against the MCF-7 cells than A431 and HCT116 cells. Compound 11b was demonstrated to be the most potent against MCF-7 than both SAHA and Nutlin-3, whose IC_50_ value reached to 1.37 ± 0.45 μM. Therefore, considering the enzyme and cellular activities, the most active compound, 11b, was selected for further evaluation.

**Table 5:** Antiproliferative activities of compounds **7u**, **7w**, **7x**, **11a**, **11b,** and **15a**.

compound	IC_50_(μM)^a^		
	A431	MCF-7	HCT116
**7u**	3.64 ± 0.56	3.47 ± 0.92	4.59 ± 0.91
**7w**	3.32 ± 0.68	3.77 ± 0.93	3.91 ± 0.84
**7x**	3.43 ± 0.73	2.90 ± 0.56	3.45 ± 0.57
**11a**	4.62 ± 0.78	4.48 ± 1.46	4.32± 0.90
**11b**	1.89 ± 0.88	1.37 ± 0.45	2.54 ± 1.07
**15a**	6.99 ± 0.92	5.71 ± 1.03	8.45± 2.44
SAHA	3.34 ± 0.95	3.13 ± 0.78	4.06 ± 1.36
Nutlin-3	9.72 ± 1.66	9.75 ± 2.97	10.98 ± 2.42

a IC_50_ = compound concentration required to inhibit tumor cell proliferation by 50%; Data displayed is the average of at least three independent replicates ± standard.

#### 2.2.3 HDACs Isoform Selectivity of Compound 11b

Subsequently, to test the HDAC isoform selectivity profile, the representative compound 11b was subjected to estimate the enzyme inhibitory effects towards ten subtypes from the four respective HDAC subfamilies: HDAC1-3 (Class I), HDAC8 (Class I), HDAC4-5 (Class IIA), HDAC7 (Class IIA) and HDAC9 (Class IIA), HDAC6 (Class IIB), HDAC10 (Class IIB), and HDAC (Class IV). The result in **Table 6** showed that compound 11b exhibited obvious selectivity for HDAC1 and HDAC2 (IC_50_ = 0.058 μM and 0.064 μM) compared with other isoforms. On the contrary, it showed submicromolar IC_50_ values variation against HDAC3 (IC_50_ = 0.115 μM), HDAC6 (IC_50_ = 0.120 μM), HDAC8 (IC_50_ = 0.313 μM). Moreover, similar to positive control SAHA, compound 11b was less active on HDAC10 (IC_50_ = 1.495 μM).

**Table 6:** IC_50_ values of compound **11b** against HDAC subtypes.

Target	IC_50_(μM)^a^		Target	IC_50_(μM)^a^	
	11b	SAHA		11b	SAHA
HDAC1	0.058 ± 0.005	0.019 ± 0.004	HDAC7	>10	>10
HDAC2	0.064 ± 0.004	0.039 ± 0.007	HDAC8	0.313 ± 0.023	0.183 ± 0.027
HDAC3	0.115 ± 0.009	0.053 ± 0.006	HDAC9	>10	>10
HDAC4	>10	>10	HDAC10	1.495 ± 0.037	0.379 ± 0.022
HDAC5	>10	>10	HDAC11	>10	>10
HDAC6	0.120 ± 0.026	0.044 ± 0.005	MDM2	0.339 ± 0.068	ND

a IC_50_ = compound concentration required to inhibit tumor cell proliferation by 50%; Data displayed is the average of at least three independent replicates ± standard.

#### 2.2.4 Molecular Docking Study of Compound 11b with MDM2 and HDAC1

In order to further investigate the inhibitory activity and the interaction manner of the most active compound 11b on both targets, molecular docking studies were performed. The proposed binding modes of 11b were analyzed using the CDOCKER method (embedded in the Accelrys Discovery Studio 3.5 package) and the MDM2 and HDAC1 protein structures were downloaded from the protein data bank (PDB) database (https://www.rcsb.org, PDB ID 4LWU).

**Figure 7:**
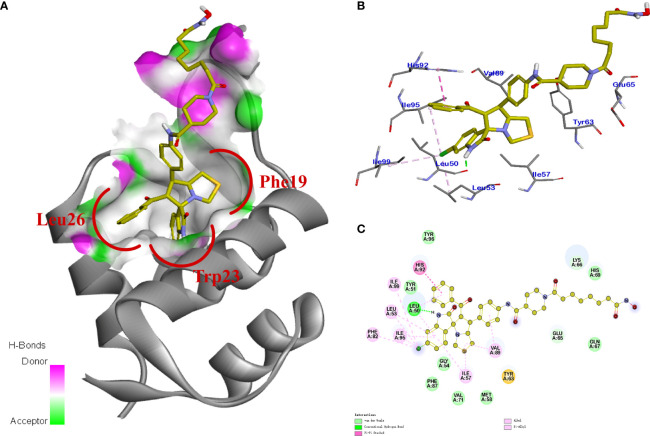
**(A)** TUNEL assay. MCF-7 cells were treated with 0.5 μM 11b. **(B)** MCF-7 cells were incubated with 5.0 μM 11b for 24 **(h)** Cell apoptosis was examined by Annexin V/PI double-stained assay. **(C)** Effects of 11b on expression levels of MDM2, p53, H4, Ac-H4 after 24 h incubation.

Previous studies revealed that spirooxindole derivatives could project into the pockets usually occupied by the three key residues (Phe19, Trp23, and Leu26) of p53. As shown in **Figure 7**, we speculated that the oxindole framework projected into the Trp23 pocket, the 3’-benzoyl group and thiazolyl moiety of compound 11b projected into the Leu26 and Phe19 residues, respectively. Actually, compound 11b mimicked the interactions of p53 binding site on MDM2 *via* hydrogen-bonding effects, and the main hydrogen-bonding interaction between spirooxindole-based cap and MDM2 appears to involve residue Leu50. Moreover, the 3’-benzoyl moiety formed π−π stacking interaction with His92 residue.

**Figure 8:**
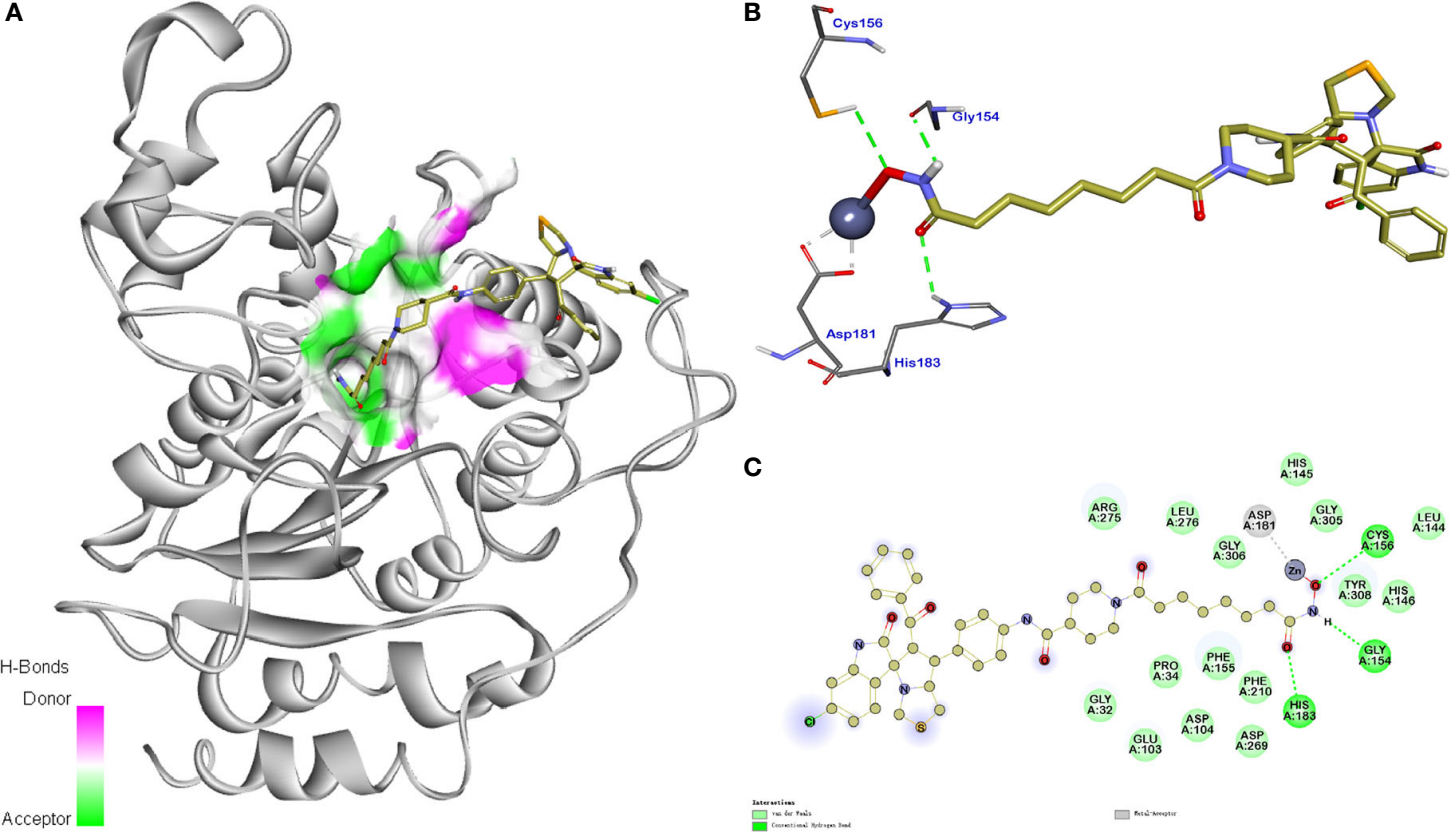
Synthesis of compounds **5a**-**5v**. Reagents and conditions: **(A)** 1 (1.0 equiv), **2** (1.0 equiv), **3** (1.0 equiv), MeOH, 65°C, 4 h; **(B)** Fe (10.0 equiv), NH_4_Cl (5.0 equiv), MeOH/H_2_O = 2:1, 60°C, 6 h.

The molecular docking study of compound 11b with HDAC1 was shown in **Figure 8**, which further demonstrated their considerable HDAC inhibitory activities. We could see that the piperidine-based linker of compound 11b protrudes deeply into the hydrophobic cavity of HDAC1, the terminal hydroxamic acid group chelated with the Zn^2+^ in a bidentate manner, thereby forming hydrogen bonding interactions with Asp181. It’s also observed that the terminal group of compound 11b could form three important hydrogen bonds with Cys156, Gyl154, and His183 in the HDAC2 active site. The molecular docking studies provided a valid explanation for the interaction modes of compound 11b with MDM2 and HDAC1, which were consistent with enzyme inhibitory activities and *in vitro* antiproliferative assay.

#### 2.2.5 Effect of Compound 11b on Cell Apoptosis

**Figure 9:**
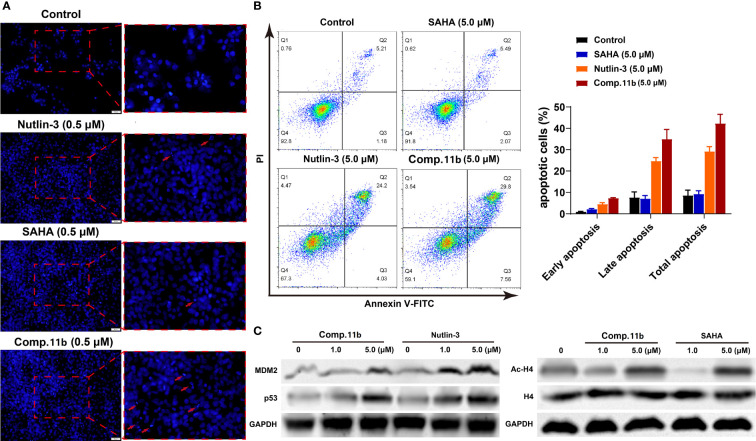
Synthesis of target compounds **7a-7y**, **9a-9e**, **11a-11b**, **13a**, **15a-15c**. Reagents and conditions: **(A)** HATU (1.2 equiv), DIPEA (2.0 equiv), DCM, rt; **(B)** TFA/DCM=1:3, rt.

It was reported that the inactivation of HDAC and/or MDM2 could increase the percentage of apoptosis cells (85–87). Therefore, three methods were applied to assess the effect of MDM2-HDAC dual inhibitor 11b on the apoptosis of MCF-7 cells. Firstly, as shown in **Figure 9A**, a significantly higher number of apoptotic positive cells were determined by TUNEL staining after treating MCF-7 cells with 0.5 μM 11b, compared with SAHA and Nutlin-3. This result was in accordance with previous studies. Subsequently, the annexin V-FITC/propidium iodide (PI) double staining method was carried out to quantitatively detect the effect of compound 11b on cell apoptosis (**Figure 9B**). According to the flow cytometry analysis, we found that the apoptotic cells reached 37.36% at a dose of 5.0 μM 11b, which was higher than that of reference compound SAHA (7.56%) and Nutlin-3 (28.23%), these results indicate that the simultaneously inhibit MDM2 and HDAC could increase the percentage of apoptosis cells.

Western blot analysis was conducted to further confirm the dual-action targeting of MDM2 and HDAC compared to the MDM2 inhibitor Nutlin-3 and the HDAC inhibitor SAHA as control. As shown in **Figure 9C** (left), treatment with compound 11b in MCF-7 cells dose-dependently increased the protein level of MDM2 as well as p53 after 24 h incubation, which was much stronger than Nutlin-3 at the same concentration. And exposure MCF-7 cells to compound 11b for 24 h, the increased acetylation level of H4 in MCF-7 cells was also observed in a dose-dependent manner (**Figure 9C**, right), similar to SAHA on the acetylation-induced capacity at the same concentration. These results demonstrated that inhibition of intracellular MDM2 and HDAC were the main mechanisms of bioactivity of compound 11b.

## 3 Materials and methods

### 3.1 Chemistry

#### 3.1.1 General information

Nuclear magnetic resonance (NMR) spectra were recorded in CDCl_3_ or DMSO-*d_6_* on Bruker 400 MHz NMR instrument (at 400 MHz for ^1^H, and at 100 for ^13^C). Protonchemical shifts are reported in parts per million (δ scale). The ^1^H NMR chemical shifts are reported in ppm with the internal TMS signal at 0.0 ppm as standard. The ^13^C NMR chemical shifts were given using CDCl_3_ or DMSO-*d_6_* as the internal standard (CDCl_3_: *δ* = 77.00 ppm; DMSO-*d_6_*: *δ* = 39.51 ppm). Data are reported as follows: chemical shift [multiplicity (s = singlet, d = doublet, t = triplet, q = quartet, m = multiplet, br = broad), coupling constant(s) (Hz), integration]. High-resolution mass spectra (HRMS) were obtained using Agilent P/N G1969-90010. High-resolution mass spectra were reported for the molecular ion [M+Na]^+^. X-ray diffraction experiment was carried out on Agilent Gemini or Agilent D8 QUEST and the data obtained were deposited at the Cambridge Crystallographic Data Centre. UV detection was performed at 254 nm. Column UV detection was performed at 254 nm. Column chromatography was performed on silica gel (200–300 mesh) using an eluent of ethyl acetate, petroleum ether, methanol, and dichloromethane. TLC was performed on glass-backed silica plates; products were visualized using UV light. All reagents and solvents were obtained from commercial sources and used without further purification. Oil baths were used as the heat source. Melting points were recorded on BUCHI Melting Point M-565 instrument.

#### 3.1.2 General procedure for the preparation of 5a-5v

A mixture of compounds 1 (5.0 mmol), 4-nitrochalcone derivatives 2 (5.0 mmol), and amino acids 3 (5.0 mmol) were stirred in MeOH at 65°C. When the reaction was complete (based on TLC monitoring), the reaction mixture was poured into cold water and stirred for 20 min. The precipitate obtained was collected and washed with cold water. The solid was recrystal with MeOH dried at 50°C to constant weight affording the products 4a-4v. To a solution of compounds 4a-4v (1.0 mmol) in MeOH/H_2_O (2:1) was added iron powder (10.0 mmol), NH_4_Cl (5.0 mmol). The mixture was stirred at 60°C. When the reaction was complete (based on TLC monitoring), the reaction mixture was cooled to ambient and filtered through a pad of celite. The filtrate was washed with saturated saltwater and extracted with DCM (15 mL × 2). The combined organic layers were dried over anhydrous MgSO_4_, filtered, and concentrated under reduced pressure, the corresponding products 5a-5v were directly applied in the next step without further purification. The spectroscopic data are given in the supporting information.

#### 3.1.3 General Procedure for the Preparation of Target Compounds 7a-7y, 9a-9e, 11a-11b, 13a, 15a-15c

To a solution of 16-20 (1.2 mmol) in DCM was added HATU (1.2 mmol),DIEA (2.0 mmol), the mixture was stirred at room temperature for 30 min. After that, compounds 5a-5v were added, and the mixture was stirred at room temperature until it was completed (based on TLC monitoring). Then the reaction mixture was concentrated and the residue was purified by flash chromatography on silica gel (DCM/MeOH = 50:1) to give intermediate 6a-6v, 8a-8e, 10a-10b, 12a, 14a-14c. The obtained intermediates (0.5 mmol) were then added TFA and stirred in DCM (TFA/DCM = 1:3) at room temperature. The reaction was monitored by TLC until it was completed, then saturated aqueous NaHCO_3_ solution was added to quench the reaction at room temperature. The mixture was extracted with EtOAc (15 mL × 2). The combined organic layers were dried over anhydrous MgSO_4_, filtered, and concentrated under reduced pressure. Then the residue was purified by flash chromatography on silica gel (DCM/MeOH = 20:1) to give the target products 7a-7y, 9a-9e, 11a-11b, 13a, 15a-15c.

***N^1^-(4-((3R,6'S,7'R,7a'S)-6'-benzoyl-2-oxo-1',6',7',7a'-tetrahydro-3'H-spiro[indoline-3,5'-pyrrolo[1,2-c]thiazol]-7'-yl)phenyl)-N^8^-hydroxyoctanediamide (7a)***

Orange solid, 50% yield, dr > 99:1, m.p. 144.7-146.1 °C. ^1^H NMR (400 MHz, DMSO-*d_6_*): δ = 10.32 (s, 2H), 9.86 (s, 1H), 8.70 (s, 1H), 7.57–7.55 (m, 2H), 7.48 (t, *J* = 7.2 Hz, 1H), 7.44–7.38 (m, 3H), 7.35–7.26 (m, 4H), 7.11 (t, *J* = 7.6 Hz, 1H), 6.93 (t, *J* = 7.2 Hz, 1H), 6.49 (d, *J* = 8.0 Hz, 1H), 4.75 (d, *J* = 11.6 Hz, 1H), 4.12–4.08 (m, 1H), 3.82 (dd, *J* = 11.6, 9.6 Hz, 1H), 3.71 (d, *J* = 10.0 Hz, 1H), 3.33 (d, *J* = 10.4 Hz, 1H), 3.05–2.97 (m, 2H), 2.28–2.24 (m, 2H), 1.95–1.91 (m, 2H), 1.56–1.46 (m, 4H), 1.26–1.24 (m, 4H) ppm; ^13^C NMR (100 MHz, DMSO-*d_6_*): δ = 196.67, 178.79, 171.61, 169.53, 142.49, 138.79, 136.97, 134.13, 133.67, 130.18, 130.17, 128.78, 128.67, 128.39, 127.91, 123.44, 121.39, 119.87, 109.93, 74.82, 73.67, 61.78, 53.92, 50.94, 36.77, 36.22, 28.83, 25.52, 25.47 ppm; HRMS (ESI-TOF) *m/z*: [M + Na]^+^ Calculated for C_34_H_36_N_4_NaO_5_S^+^ 635.2299, found 635.2305.

***N^1^-(4-((3R,6'S,7'R,7a'S)-6'-benzoyl-5-methyl-2-oxo-1',6',7',7a'-tetrahydro-3'H-spiro[indoline-3,5'-pyrrolo[1,2-c]thiazol]-7'-yl)phenyl)-N^8^-hydroxyoctanediamide (7b)***

Orange solid, 41% yield, dr > 99:1, m.p. 144.2-145.6 °C. ^1^H NMR (400 MHz, DMSO-*d*_6_): δ = 10.29 (s, 2H), 10.03 (s, 1H), 8.79 (br, 1H), 7.59–7.57 (m, 2H), 7.47 (t, *J* = 7.6 Hz, 1H), 7.447–7.34 (m, 4H), 7.30–7.21 (m, 3H), 6.91 (d, *J* = 7.6 Hz, 1H), 6.40 (d, *J* = 8.0 Hz, 1H), 4.75 (d, *J* = 11.6 Hz, 1H), 4.12–4.07 (m, 1H), 3.81 (dd, *J* = 11.2 Hz, 9.2 Hz, 1H), 3.69 (d, *J* = 10.0 Hz, 1H), 3.32 (d, *J* = 10.0 Hz, 1H), 3.04–2.96 (m, 2H), 2.30–2.26 (m, 2H), 2.25 (s, 3H), 1.96–1.92 (m, 2H), 1.58–1.53 (m, 2H), 1.49–1.44 (m, 2H), 1.30–1.23 (m, 4H) ppm; ^13^C NMR (100 MHz, DMSO-*d*_6_): δ = 196.67, 178.78, 171.69, 169.62, 140.09, 138.84, 137.02, 134.09, 133.64, 130.41, 130.01, 128.89, 128.79, 128.60, 127.93, 123.59, 119.89, 109.66, 74.77, 73.60, 61.83, 53.69, 50.88, 36.75, 36.07, 32.68, 28.82, 25.55, 25.49, 21.30 ppm; HRMS (ESI-TOF) *m/z*: [M + Na]^+^ Calculated for C_35_H_38_N_4_NaO_5_S^+^ 649.2455, found 649.2461.

***N^1^-(4-((3R,6'S,7'R,7a'S)-6'-benzoyl-5-fluoro-2-oxo-1',6',7',7a'-tetrahydro-3'H-spiro[indoline-3,5'-pyrrolo[1,2-c]thiazol]-7'-yl)phenyl)-N^8^-hydroxyoctanediamide (7c)***

Orange solid, 43% yield, dr > 99:1, m.p. 144.3-145.7 °C. ^1^H NMR (400 MHz, DMSO-*d*_6_): δ = 10.43 (s, 2H), 10.01 (s, 1H), 8.87 (br, 1H), 7.60–7.58 (m, 2H), 7.51 (t, *J* = 7.6 Hz, 1H), 7.46–7.40 (m, 4H), 7.34–7.26 (m, 3H), 7.00 (td, *J* = 8.8 Hz, 2.4 Hz, 1H), 6.53 (dd, *J* = 8.4 Hz, 4.4 Hz, 1H), 4.79 (d, *J* = 11.6 Hz, 1H), 4.13–4.09 (m, 1H), 3.81 (t, *J* = 11.2 Hz, 1H), 3.74 (d, *J* = 10.4 Hz, 1H), 3.36 (d, *J* = 10.4 Hz, 1H), 3.07–3.00 (m, 2H), 2.30–2.26 (m, 2H), 2.25 (s, 3H), 1.97–1.93 (m, 2H), 1.55–1.52 (m, 2H), 1.50–1.46 (m, 2H), 1.31–1.23 (m, 4H) ppm; ^13^C NMR (100 MHz, DMSO-*d*_6_): δ = 196.53, 178.63, 171.70, 169.63, 158.67, 156.32, 138.90, 138.79, 136.80, 133.95, 133.79, 128.93, 128.66, 128.01, 125.15, 125.07, 119.89, 116.87, 116.64, 116.10, 115.85, 110.78, 74.82, 74.24, 61.55, 54.15, 51.04, 36.76, 36.29, 32.69, 28.82, 25.55, 25.49 ppm; HRMS (ESI-TOF) *m/z*: [M + Na]^+^ Calculated for C_34_H_35_FN_4_NaO_5_S^+^ 653.2204, found 653.2208.

***N^1^-(4-((3R,6'S,7'R,7a'S)-6'-benzoyl-5-chloro-2-oxo-1',6',7',7a'-tetrahydro-3'H-spiro[indoline-3,5'-pyrrolo[1,2-c]thiazol]-7'-yl)phenyl)-N^8^-hydroxyoctanediamide (7d)***

Orange solid, 39% yield, dr > 99:1, m.p. 154.6-156.2 °C. ^1^H NMR (400 MHz, DMSO-*d*_6_): δ = 9.90 (s, 1H), 7.57–7.55 (m, 2H), 7.02 (t, *J* = 6.8 Hz, 1H), 7.46–7.44 (m, 3H), 7.40–7.39 (m, 2H), 7.34–7.30 (m, 2H), 7.19 (d, *J* = 7.6 Hz, 1H), 6.53 (d, *J* = 8.0 Hz, 1H), 4.79 (d, *J* = 11.6 Hz, 1H), 4.12–4.08 (m, 1H), 3.77 (t, *J* = 10.4 Hz, 1H), 3.73 (d, *J* = 10.4 Hz, 1H), 3.35 (d, *J* = 10.4 Hz, 1H), 3.06–2.99 (m, 2H), 2.28–2.25 (m, 2H), 1.96–1.92 (m, 2H), 1.57–1.46 (m, 4H), 1.29–1.23 (m, 4H) ppm; ^13^C NMR (100 MHz, DMSO-*d*_6_): δ = 196.58, 178.42, 171.63, 169.53, 141.51, 138.85, 136.79, 133.94, 133.71, 130.11, 128.92, 128.69,128.33, 127.98, 125.39, 119.86, 111.38, 74.78, 74.02, 61.51, 54.24, 51.10, 36.78, 36.28, 32.69, 28.81, 25.52, 25.48 ppm; HRMS (ESI-TOF) *m/z*: [M + Na]^+^ Calculated for C_34_H_35_ClN_4_NaO_5_S^+^ 669.1909, found 669.1916.

***N^1^-(4-((3R,6'S,7'R,7a'S)-6'-benzoyl-5-bromo-2-oxo-1',6',7',7a'-tetrahydro-3'H-spiro[indoline-3,5'-pyrrolo[1,2-c]thiazol]-7'-yl)phenyl)-N^8^-hydroxyoctanediamide (7e)***

Orange solid, 30% yield, dr > 99:1, m.p. 146.8-148.0 °C. ^1^H NMR (400 MHz, DMSO-*d*_6_): δ = 9.90 (s, 1H), 7.57–7.55 (m, 3H), 7.50 (t, *J* = 7.2 Hz, 1H), 7.47–7.45 (m, 2H), 7.41–7.39 (m, 2H), 7.34–7.30 (m, 3H), 6.49 (d, *J* = 8.0 Hz, 1H), 4.79 (d, *J* = 11.6 Hz, 1H), 4.12–4.08 (m, 1H), 3.79–3.77 (m, 1H), 3.72 (d, *J* = 10.4 Hz, 1H), 3.36 (d, *J* = 10.4 Hz, 1H), 3.07–2.99 (m, 2H), 2.29–2.25 (m, 2H), 1.96–1.92 (m, 2H), 1.57–1.46 (m, 4H), 1.30–1.23 (m, 4H) ppm; ^13^C NMR (100 MHz, DMSO-*d*_6_): δ = 196.61, 178.43, 171.64, 169.52, 142.19, 138.86, 136.82, 133.93, 133.73, 132.91, 131.01, 130.10, 128.93, 128.70, 127.99, 128.88, 119.87, 113.07, 111.93, 74.77, 73.96, 61.57, 54.24, 51.11, 36.79, 36.26, 32.71, 28.83, 25.53, 25.50 ppm; HRMS (ESI-TOF) *m/z*: [M + Na]^+^ Calculated for C_34_H_35_BrN_4_NaO_5_S^+^ 713.1404, found 713.1404.

***N^1^-(4-((3R,6'S,7'R,7a'S)-6'-benzoyl-6-chloro-2-oxo-1',6',7',7a'-tetrahydro-3'H-spiro[indoline-3,5'-pyrrolo[1,2-c]thiazol]-7'-yl)phenyl)-N^8^-hydroxyoctanediamide (7f)***

Orange solid, 39% yield, dr > 12:1, m.p. 150.2-151.2 °C. ^1^H NMR (400 MHz, DMSO-*d*_6_): δ = 10.48 (s, 2H), 10.04 (s, 1H), 8.77 (s, 1H), 7.60–7.58 (m, 2H), 7.52 (t, *J* = 7.2 Hz, 1H), 7.43–7.39 (m, 5H), 7.35–7.31 (m, 2H), 7.02 (dd, *J* = 8.0, 1.6 Hz, 1H), 6.56 (d, *J* = 2.0 Hz, 1H), 4.77 (d, *J* = 11.6 Hz, 1H), 4.12–4.08 (m, 1H), 3.82–3.77 (m, 1H), 3.72 (d, *J* = 10.4 Hz, 1H), 3.35 (d, *J* = 10.4 Hz, 1H), 3.06–2.98 (m, 2H), 2.30–2.26 (m, 2H), 1.97–1.93 (m, 2H), 1.59–1.51 (m, 2H), 1.49–1.44 (m, 2H), 1.30–1.24 (m, 4H) ppm; ^13^C NMR (100 MHz, DMSO-*d*_6_): δ = 196.55, 178.68, 171.71, 169.63, 144.16, 138.93, 136.82, 134.44, 133.96, 133.83, 129.94, 128.95, 128.64, 127.97, 122.32, 121.12, 119.91, 110.09, 74.82, 73.55, 61.54, 54.17, 51.17, 36.78, 36.36, 32.70, 28.85, 28.83, 25.56, 25.51 ppm; HRMS (ESI-TOF) *m/z*: [M + Na]^+^ Calculated for C_34_H_35_ClN_4_NaO_5_S^+^ 669.1909, found 669.1908.

***N^1^-(4-((3R,6'S,7'R,7a'S)-6'-benzoyl-6-bromo-2-oxo-1',6',7',7a'-tetrahydro-3'H-spiro[indoline-3,5'-pyrrolo[1,2-c]thiazol]-7'-yl)phenyl)-N^8^-hydroxyoctanediamide (7g)***

Orange solid, 34% yield, dr > 20:1, m.p. 149.8-152.1 °C. ^1^H NMR (400 MHz, DMSO-*d*_6_): δ = 10.39 (s, 1H), 9.96 (s, 1H), 8.76 (br, 1H), 7.58–7.56 (m, 2H), 7.52 (t, *J* = 7.2 Hz, 1H), 7.46–7.37 (m, 4H), 7.37–7.30 (m, 3H), 7.15 (dd, *J* = 8.0 Hz, 1.6 Hz, 1H), 6.67 (d, *J* = 2.0 Hz, 1H), 4.77 (d, *J* = 12.0 Hz, 1H), 4.11–4.07 (m, 1H), 3.78 (dd, *J* = 11.6 Hz, 9.2 Hz, 1H), 3.71 (d, *J* = 10.4 Hz, 1H), 3.36 (s, 1H), 3.05–2.97 (m, 2H), 2.29–2.25 (m, 2H), 1.95–1.91 (t, *J* = 7.3 Hz, 2H), 1.56–1.45 (tm, 4H), 1.28–1.24 (m, 4H) ppm; ^13^C NMR (100 MHz, DMSO-*d*_6_): δ = 196.51, 178.68, 171.63, 169.56, 144.36, 138.82, 136.79, 133.91, 133.86, 130.23, 128.90, 128.66, 127.96, 123.98, 122.94, 122.77, 119.88, 112.82, 74.80, 73.64, 61.52, 54.17, 51.15, 36.78, 36.35, 32.69, 28.83, 28.81, 25.52, 25.48 ppm; HRMS (ESI-TOF) *m/z*: [M + Na]^+^ Calculated for C_34_H_35_BrN_4_NaO_5_S^+^ 713.1404, found 713.1400.

***N^1^-(4-((3R,6'S,7'R,7a'S)-6'-benzoyl-7-methyl-2-oxo-1',6',7',7a'-tetrahydro-3'H-spiro[indoline-3,5'-pyrrolo[1,2-c]thiazol]-7'-yl)phenyl)-N^8^-hydroxyoctanediamide (7h)***

Orange solid, 37% yield, dr > 20:1, m.p. 142.7-143.9 °C. ^1^H NMR (400 MHz, DMSO-*d*_6_): δ = 10.31 (s, 1H), 10.09 (s, 1H), 8.97 (br, 1H), 7.62–7.59 (m, 2H), 7.49–7.45 (m, 1H), 7.43–7.41 (m, 2H), 7.29–7.23 (m, 4H), 7.20 (d, *J* = 7.6 Hz, 1H), 6.93 (d, *J* = 7.6 Hz, 1H), 6.84 (t, *J* = 7.6 Hz, 1H), 4.73 (d, *J* = 11.6 Hz, 1H), 4.12–4.07 (m, 1H), 3.78 (dd, *J* = 11.2 Hz, 9.2 Hz, 1H), 3.71 (d, *J* = 10.0 Hz, 1H), 3.35 (d, *J* = 10.0 Hz, 1H), 3.05–2.96 (m, 2H), 2.31–2.27 (m, 2H), 1.97–1.93 (m, 2H), 1.86 (s, 3H), 1.59–1.52 (m, 2H), 1.50–1.44 (m, 2H), 1.29–1.23 (m, 4H) ppm; ^13^C NMR (100 MHz, DMSO-*d*_6_): δ = 197.09, 179.42, 171.72, 169.63, 141.13, 138.85, 137.23, 134.17, 133.34, 131.28, 128.62, 128.58, 127.71, 125.51, 123.22, 121.34, 119.90, 119.27, 74.93, 73.84, 62.46, 53.89, 50.71, 36.76, 36.19, 32.68, 28.82, 25.56, 25.50, 16.47 ppm; HRMS (ESI-TOF) *m/z*: [M + Na]^+^ Calculated for C_35_H_38_N_4_NaO_5_S^+^ 649.2455, found 649.2462.

***N^1^-(4-((3R,6'S,7'R,7a'S)-6'-benzoyl-7-bromo-2-oxo-1',6',7',7a'-tetrahydro-3'H-spiro[indoline-3,5'-pyrrolo[1,2-c]thiazol]-7'-yl)phenyl)-N^8^-hydroxyoctanediamide (7i)***

Orange solid, 40% yield, dr > 17:1, m.p. 137.5-138.9 °C. ^1^H NMR (400 MHz, DMSO-*d*_6_): δ = 10.10 (s, 1H), 7.62–7.60 (m, 2H), 7.53-7.48 (m, 1H), 7.44–7.42 (m, 2H), 7.39 (d, *J* = 7.6 Hz, 1H), 7.33–7.28 (m, 5H), 6.92 (t, *J* = 8.0 Hz, 1H), 4.77 (d, *J* = 11.6 Hz, 1H), 4.13–4.09 (m, 1H), 3.79 (dd, *J* = 11.6 Hz, 9.2 Hz, 1H), 3.73 (d, *J* = 10.4 Hz, 1H), 3.38 (d, *J* = 10.4 Hz, 1H), 3.06–2.98 (m, 2H), 2.31–2.27 (m, 2H), 1.97–1.93 (m, 2H), 1.57–1.52 (m, 2H), 1.50–1.46 (m, 2H), 1.29–1.23 (m, 4H) ppm; ^13^C NMR (100 MHz, DMSO-*d*_6_): δ = 196.88, 178.87, 171.72, 169.61, 141.96, 138.93, 137.00, 133.82, 133.68, 133.03, 128.80, 128.63, 127.71, 127.27, 125.59, 123.00, 119.90, 102.74, 74.88, 74.53, 62.49, 53.91, 50.78, 36.75, 36.17, 32.68, 28.82, 25.55, 25.49 ppm; HRMS (ESI-TOF) *m/z*: [M + Na]^+^ Calculated for C_34_H_35_BrN_4_NaO_5_S^+^ 713.1404, found 713.1403.

***N^1^-(4-((3R,6'S,7'R,7a'S)-6'-benzoyl-5-methoxy-2-oxo-1',6',7',7a'-tetrahydro-3'H-spiro[indoline-3,5'-pyrrolo[1,2-c]thiazol]-7'-yl)phenyl)-N^8^-hydroxyoctanediamide (7j)***

Orange solid, 35% yield, dr > 20:1, m.p. 134.9-136.2 °C. ^1^H NMR (400 MHz, DMSO-*d*_6_): δ = 10.28 (s, 2H), 10.04 (s, 1H), 8.86 (br, 1H), 7.60–7.58 (m, 2H), 7.49 (t, *J* = 7.2 Hz, 1H), 7.44–7.39 (m, 4H), 7.32–7.29 (m, 2H), 7.04 (d, *J* = 2.4 Hz, 1H), 6.71 (dd, *J* = 8.4 Hz, 2.8 Hz, 1H), 6.44 (d, *J* = 8.4 Hz, 1H), 4.76 (d, *J* = 11.6 Hz, 1H), 4.14–4.09 (m, 1H), 3.79 (dd, *J* = 11.6 Hz, 9.6 Hz, 1H), 3.74–3.71 (m, 4H), 3.36 (d, *J* = 10.0 Hz, 1H), 3.05 –3.00 (m, 2H), 2.29 (t, *J* = 7.2 Hz, 2H), 1.95 (t, *J* = 7.2 Hz, 2H), 1.57–1.52 (m, 2H), 1.50–1.45 (m, 2H), 1.31–1.24 (m, 4H) ppm; ^13^C NMR (100 MHz, DMSO-*d*_6_): δ = 196.50, 178.67, 171.68, 169.62, 154.38, 138.86, 136.92, 135.76, 133.97, 133.73, 128.82, 128.63, 127.96, 124.66, 119.88, 115.60, 114.71, 110.18, 74.76, 74.13, 61.66, 55.98, 54.05, 50.98, 36.74, 36.17, 32.67, 28.81, 25.51, 25.48 ppm; HRMS (ESI-TOF) *m/z*: [M + Na]^+^ Calculated for C_35_H_38_N_4_NaO_6_S^+^ 665.2404, found 665.2407.

***N^1^-(4-((3R,6'S,7'R,7a'S)-6'-benzoyl-6-methoxy-2-oxo-1',6',7',7a'-tetrahydro-3'H-spiro[indoline-3,5'-pyrrolo[1,2-c]thiazol]-7'-yl)phenyl)-N^8^-hydroxyoctanediamide (7k)***

Orange solid, 29% yield, dr > 20:1, m.p. 139.4-136.2 °C. ^1^H NMR (400 MHz, DMSO-*d*_6_): δ = 10.03 (s, 1H), 7.60–7.58 (m, 2H), 7.50 (t, *J* = 7.6 Hz, 1H), 7.42–7.38 (m, 4H), 7.33–7.29 (m, 3H), 6.50 (dd, *J* = 8.4 Hz, 2.0 Hz, 1H), 6.07 (d, *J* = 2.0 Hz, 1H), 4.74 (d, *J* = 11.6 Hz, 1H), 4.10–4.05 (m, 1H), 3.81-3.75 (m, 1H), 3.70 (d, *J* = 10.0 Hz, 1H), 3.68 (s, 3H), 3.36 (d, *J* = 10.0 Hz, 1H), 3.05–2.96 (m, 2H), 2.28 (t, *J* = 7.2 Hz, 2H), 1.95 (t, *J* = 7.2 Hz, 2H), 1.59–1.52 (m, 2H), 1.50–1.44 (m, 2H), 1.31–1.23 (m, 4H) ppm; ^13^C NMR (100 MHz, DMSO-*d*_6_): δ = 196.68, 179.36, 171.67, 169.60, 160.91, 143.98, 138.83, 137.02, 134.15, 133.67, 129.47, 128.81, 128.60, 127.94, 119.88, 114.97, 106.42, 96.57, 74.80, 73.65, 61.35, 55.59, 54.15, 51.10, 36.75, 36.42, 32.68, 28.82, 25.54, 25.48 ppm; HRMS (ESI-TOF) *m/z*: [M + Na]^+^ Calculated for C_35_H_38_N_4_NaO_6_S^+^ 665.2404, found 665.2411.

***N^1^-hydroxy-N^8^-(4-((3R,6'S,7'R,7a'S)-6'-(3-methoxybenzoyl)-2-oxo-1',6',7',7a'-tetrahydro-3'H-spiro[indoline-3,5'-pyrrolo[1,2-c]thiazol]-7'-yl)phenyl)octanediamide (7l)***

Orange solid, 31% yield, dr > 20:1, m.p. 121.6-122.8 °C. ^1^H NMR (400 MHz, DMSO-*d*_6_): δ = 10.38 (s, 1H), 9.97 (s, 1H), 8.81 (br, 1H), 7.59–7.57 (m, 2H), 7.44–7.42 (m, 2H), 7.38 (d, *J* = 7.2 Hz, 1H), 7.19 (t, *J* = 8.0 Hz, 1H), 7.12 (t, *J* = 7.6 Hz, 1H), 7.04 (dd, *J* = 8.4, 2.8 Hz, 1H), 6.95–6.92 (m, 2H), 6.85–6.84 (m, 1H), 6.53 (d, *J* = 7.6 Hz, 1H), 4.73 (d, *J* = 11.6 Hz, 1H), 4.12–4.07 (m, 1H), 3.81 (dd, *J* = 11.6 Hz, 9.2 Hz, 1H), 3.70 (d, *J* = 10.0 Hz, 1H), 3.69 (s, 3H), 3.33 (d, *J* = 10.0 Hz, 1H), 3.05–2.97 (m, 2H), 2.28 (t, *J* = 7.2 Hz, 2H), 1.94 (t, *J* = 7.6 Hz, 2H), 1.57–1.51(m, 2H), 1.50–1.45 (m, 2H), 1.30–1.23 (m, 4H) ppm; ^13^C NMR (100 MHz, DMSO-*d*_6_): δ = 196.42, 178.87, 171.67, 169.59, 159.35, 142.59, 138.84, 138.32, 134.09, 130.17, 129.91, 128.68, 128.36, 123.50, 121.41, 120.26, 120.08, 119.90, 112.29, 109.98, 74.78, 73.71, 61.91, 55.57, 54.00, 51.04, 36.78, 36.27, 32.70, 28.85, 25.56, 25.50 ppm; HRMS (ESI-TOF) *m/z*: [M + Na]^+^ Calculated for C_35_H_38_N_4_NaO_6_S^+^ 665.2404, found 665.2402.

***N^1^-(4-((3R,6'S,7'R,7a'S)-6'-(4-(dimethylamino)benzoyl)-2-oxo-1',6',7',7a'-tetrahydro-3'H-spiro[indoline-3,5'-pyrrolo[1,2-c]thiazol]-7'-yl)phenyl)-N^8^-hydroxyoctanediamide (7m)***

Orange solid, 33% yield, dr > 16:1, m.p. 148.7-150.0 °C. ^1^H NMR (400 MHz, DMSO-*d*_6_): δ = 10.24 (s, 1H), 9.91 (s, 1H), 7.55–7.53 (m, 2H), 7.44 (d, *J* = 7.6 Hz, 1H), 7.39–7.34 (m, 4H), 7.10 (t, *J* = 7.6 Hz, 1H), 6.91 (t, *J* = 7.2 Hz, 1H), 6.58 (d, *J* = 7.2 Hz, 1H), 6.50 (d, *J* = 8.4 Hz, 2H), 4.64 (d, *J* = 11.6 Hz, 1H), 4.12–4.08 (m, 1H), 3.84 (dd, *J* = 11.6 Hz, 9.2 Hz, 1H), 3.71 (d, *J* = 10.0 Hz, 1H), 3.32 (d, *J* = 10.0 Hz, 1H), 3.03–2.96 (m, 2H), 2.92 (s, 6H), 2.25 (t, *J* = 7.6 Hz, 2H), 1.93 (t, *J* = 7.2 Hz, 2H), 1.57–1.51 (m, 2H), 1.49–1.44 (m, 2H), 1.29–1.23 (m, 4H) ppm; ^13^C NMR (100 MHz, DMSO-*d*_6_): δ = 192.37, 179.12, 171.61, 169.50, 153.64, 142.44, 138.72, 130.32, 129.85, 128.86, 128.53, 124.51, 123.85, 121.18, 119.84, 110.82, 109.83, 74.77, 74.10, 60.36, 53.86, 51.46, 36.78, 36.26, 32.71, 28.83, 25.54, 25.50 ppm; HRMS (ESI-TOF) *m/z*: [M + Na]^+^ Calculated for C_36_H_41_N_5_NaO_5_S^+^ 678.2721, found 678.2722.

***N^1^-(4-((3R,6'S,7'R,7a'S)-6'-(3-bromobenzoyl)-2-oxo-1',6',7',7a'-tetrahydro-3'H-spiro[indoline-3,5'-pyrrolo[1,2-c]thiazol]-7'-yl)phenyl)-N^8^-hydroxyoctanediamide (7n)***

Orange solid, 27% yield, dr > 10:1, m.p. 120.2-121.5 °C. ^1^H NMR (400 MHz, DMSO-*d*_6_): δ = 10.37 (s, 1H), 9.98 (s, 1H), 8.81 (br, 1H), 7.68 (d, *J* = 7.6 Hz, 1H), 7.60–7.58 (m, 2H), 7.47–7.45 (m, 2H), 7.38 (d, *J* = 7.6 Hz, 1H), 7.32–7.31 (m, 2H), 7.25 (t, *J* = 8.0 Hz, 1H), 7.15 (t, *J* = 7.6 Hz, 1H), 6.95 (t, *J* = 7.6 Hz, 1H), 6.52 (d, *J* = 7.6 Hz, 1H), 4.73 (d, *J* = 11.6 Hz, 1H), 4.11–4.07 (m, 1H), 3.78 (dd, *J* = 11.6 Hz, 9.6 Hz, 1H), 3.72 (d, *J* = 10.4 Hz, 1H), 3.34 (d, *J* = 10.4 Hz, 1H), 3.05–2.98 (m, 2H), 2.29 (t, *J* = 7.6 Hz, 2H), 1.95 (t, *J* = 7.2 Hz, 2H), 1.60–1.52 (m, 2H), 1.50–1.45 (m, 2H), 1.31–1.23 (m, 4H) ppm; ^13^C NMR (100 MHz, DMSO-*d*_6_): δ = 196.09, 178.61, 171.66, 169.58, 142.60, 138.97, 138.86, 136.15, 133.88, 130.91, 130.37, 130.30, 128.74, 128.24, 126.81, 123.27, 122.18, 121.48, 119.87, 110.03, 74.88, 73.64, 62.14, 54.06, 50.75, 36.79, 36.23, 32.70, 28.84, 25.55, 25.50 ppm; HRMS (ESI-TOF) *m/z*: [M + Na]^+^ Calculated for C_34_H_35_BrN_4_NaO_5_S^+^ 713.1404, found 713.1404.

***N^1^-(4-((3R,6'S,7'R,7a'S)-6'-benzoyl-2-oxo-1',6',7',7a'-tetrahydro-3'H-spiro[indoline-3,5'-pyrrolo[1,2-c]thiazol]-7'-yl)phenyl)-N^4^-hydroxysuccinamide (7o)***

Orange solid, 20% yield, dr > 16:1, m.p. 145.1-146.3 °C. ^1^H NMR (400 MHz, DMSO-*d*_6_): δ = 10.38 (s, 1H), 10.10 (s, 1H), 8.79 (br, 1H), 7.58–7.56 (m, 2H), 7.48 (d, *J* = 7.2 Hz, 2H), 7.43–7.38 (m, 3H), 7.35–7.33 (m, 2H), 7.30–7.26 (m, 2H), 7.11 (d, *J* = 8.0 Hz, 1H), 6.93 (t, *J* = 7.2 Hz, 1H), 6.51 (d, *J* = 7.6 Hz, 1H), 4.76 (d, *J* = 11.6 Hz, 1H), 4.13–4.08 (m, 1H), 3.82 (dd, *J* = 11.6 Hz, 9.2 Hz, 1H), 3.71 (d, *J* = 10.0 Hz, 1H), 3.33 (d, *J* = 10.0 Hz, 1H), 3.05–2.97 (m, 2H), 2.56–2.51 (m, 2H), 2.28 (t, *J* = 7.6 Hz, 2H) ppm; ^13^C NMR (100 MHz, DMSO-*d*_6_): δ = 196.67, 178.77, 170.62, 168.82, 142.53, 138.79, 136.98, 134.12, 133.69, 130.17, 128.79, 128.64, 128.38, 127.91, 123.45, 121.38, 119.80, 109.98, 74.82, 73.67, 61.79, 53.90, 50.96, 36.35, 36.23, 32.03, 28.05 ppm; HRMS (ESI-TOF) *m/z*: [M + Na]^+^ Calculated for C_30_H_28_N_4_NaO_5_S^+^ 579.1673, found 579.1676.

***N^1^-(4-((3R,6'S,7'R,7a'S)-6'-benzoyl-2-oxo-1',6',7',7a'-tetrahydro-3'H-spiro[indoline-3,5'-pyrrolo[1,2-c]thiazol]-7'-yl)phenyl)-N^6^-hydroxyadipamide (7p)***

Orange solid, 26% yield, dr > 99:1, m.p. 140.3-141.7 °C. ^1^H NMR (400 MHz, DMSO-*d*_6_): δ = 10.37 (s, 2H), 10.00 (s, 1H), 8.75 (br, 1H), 7.60–7.58 (m, 2H), 7.50–7.39 (m, 3H), 7.36–7.34 (m, 2H), 7.31–7.27 (m, 2H), 7.11 (t, *J* = 7.6 Hz, 1H), 6.94 (t, *J* = 7.6 Hz, 1H), 6.51 (d, *J* = 7.6 Hz, 1H), 4.77 (d, *J* = 11.6 Hz, 1H), 4.13–4.09 (m, 1H), 3.83 (dd, *J* = 11.6 Hz, 9.2 Hz, 1H), 3.71 (d, *J* = 10.0 Hz, 1H), 3.33 (d, *J* = 10.0 Hz, 1H), 3.06–2.98 (m, 2H), 2.29 (t, *J* = 6.8 Hz, 2H), 1.99 (t, *J* = 6.8 Hz, 2H), 1.59–1.49 (m, 4H) ppm; ^13^C NMR (100 MHz, DMSO-*d*_6_): δ = 196.67, 178.79, 171.51, 169.44, 142.51, 138.80, 136.97, 134.14, 133.67, 130.17, 128.79, 128.65, 128.39, 127.91, 123.45, 121.39, 119.89, 109.96, 74.83, 73.69, 61.78, 53.94, 50.97, 36.56, 36.24, 32.57, 25.31, 25.26 ppm; HRMS (ESI-TOF) *m/z*: [M + Na]^+^ Calculated for C_32_H_32_N_4_NaO_5_S^+^ 607.1986, found 607.1986.

***N^1^-(4-((3R,6'S,7'R,7a'S)-6'-benzoyl-2-oxo-1',6',7',7a'-tetrahydro-3'H-spiro[indoline-3,5'-pyrrolo[1,2-c]thiazol]-7'-yl)phenyl)-N^10^-hydroxydecanediamide (7q)***

Orange solid, 35% yield, dr > 16:1, m.p. 131.9-132.1 °C. ^1^H NMR (400 MHz, DMSO-*d*_6_): δ = 10.39 (s, 2H), 10.01 (s, 1H), 8.80 (s, 1H), 7.60–7.58 (m, 2H), 7.48 (t, *J* = 7.2 Hz, 1H), 7.44–7.39 (m, 3H), 7.36–7.34 (m, 2H), 7.31–7.27 (m, 2H), 7.11 (t, *J* = 7.6 Hz, 1H), 6.93 (t, *J* = 7.6 Hz, 1H), 6.51 (d, *J* = 7.6 Hz, 1H), 4.76 (d, *J* = 11.6 Hz, 1H), 4.13–4.09 (m, 1H), 3.83 (dd, *J* = 11.6 Hz, 9.2 Hz, 1H), 3.71 (d, *J* = 10.0 Hz, 1H), 3.33 (d, *J* = 10.0 Hz, 1H), 3.05–2.97 (m, 2H), 2.28 (t, *J* = 7.6 Hz, 2H), 1.94 (t, *J* = 7.2 Hz, 2H), 1.58–1.52 (m, 2H), 1.49–1.44 (m, 2H), 1.28–1.20 (m, 8H) ppm; ^13^C NMR (100 MHz, DMSO-*d*_6_): δ = 196.65, 17.8.78, 171.71, 169.61, 142.53, 138.86, 136.97, 134.08, 133.67, 130.16, 128.78, 128.62, 128.38, 127.91, 123.45, 121.37, 119.88, 109.97, 74.82, 73.68, 61.78, 53.92, 50.98, 36.81, 36.23, 32.70, 29.18, 29.10, 29.07, 29.01, 25.65, 25.57 ppm; HRMS (ESI-TOF) *m/z*: [M + Na]^+^ Calculated for C_36_H_40_N_4_NaO_5_S^+^ 663.2612, found 663.2613.

***N^1^-(4-((3R,6'S,7'R,7a'S)-6'-benzoyl-6-chloro-2-oxo-1',6',7',7a'-tetrahydro-3'H-spiro[indoline-3,5'-pyrrolo[1,2-c]thiazol]-7'-yl)phenyl)-N^6^-hydroxyadipamide (7r)***

Orange solid, 30% yield, dr > 13:1, m.p. 160.6-161.9 °C. ^1^H NMR (400 MHz, DMSO-*d*_6_): δ = 10.47 (s, 2H), 9.99 (s, 1H), 8.74 (br, 1H), 7.59–7.57 (m, 2H), 7.52 (t, *J* = 7.2 Hz, 1H), 7.44–7.39 (m, 5H), 7.35–7.31 (m, 2H), 7.02 (dd, *J* = 8.0 Hz, 2.0 Hz, 1H), 6.54 (d, *J* = 2.0 Hz, 1H), 4.78 (d, *J* = 11.6 Hz, 1H), 4.13–4.08 (m, 1H), 3.80 (dd, *J* = 11.6 Hz, 9.6 Hz, 1H), 3.72 (d, *J* = 10.4 Hz, 1H), 3.36 (d, *J* = 10.4 Hz, 1H), 3.06–2.98 (m, 2H), 2.29 (t, *J* = 6.4 Hz, 2H), 1.98 (t, *J* = 6.4 Hz, 2H), 1.59–1.49 (m, 4H) ppm; ^13^C NMR (100 MHz, DMSO-*d*_6_): δ = 196.56, 178.7, 171.53, 169.45, 144.12, 138.87, 136.84, 134.46, 133.94, 133.89, 129.94, 128.93, 128.67, 127.97, 122.33, 121.13, 119.90, 110.04, 74.83, 73.56, 61.57, 54.18, 51.14, 36.58, 36.36, 32.58, 25.32, 25.27 ppm; HRMS (ESI-TOF) *m/z*: [M + Na]^+^ Calculated for C_32_H_31_ClN_4_NaO_5_S^+^ 641.1596, found 641.1596.

***N^1^-(4-((3R,6'S,7'R,7a'S)-6'-benzoyl-6-chloro-2-oxo-1',6',7',7a'-tetrahydro-3'H-spiro[indoline-3,5'-pyrrolo[1,2-c]thiazol]-7'-yl)phenyl)-N^10^-hydroxydecanediamide (7s)***

Orange solid, 37% yeld, dr > 99:1, m.p. 137.7-139.0 °C. ^1^H NMR (400 MHz, DMSO-*d*_6_): δ = 10.38 (s, 2H), 9.88 (s, 1H), 8.87 (br, 1H), 7.58–7.56 (m, 2H), 7.52 (t, *J* = 7.2 Hz, 1H), 7.44–7.39 (m, 5H), 7.35–7.31 (m, 3H), 7.02 (d, *J* = 8.0 Hz, 1H), 6.53 (s, 1H), 4.78 (d, *J* = 11.6 Hz, 1H), 4.13–4.08 (m, 1H), 3.80 (dd, *J* = 11.6 Hz, 9.2 Hz, 1H), 3.72 (d, *J* = 10.4 Hz, 1H), 3.36 (d, *J* = 10.4 Hz, 1H), 3.06–2.98 (m, 2H), 2.27 (t, *J* = 7.2 Hz, 2H), 1.93 (t, *J* = 7.2 Hz, 2H), 1.59–1.55 (m, 2H), 1.49–1.46 (m, 2H), 1.28–1.20 (m, 8H) ppm; ^13^C NMR (100 MHz, DMSO-*d*_6_): δ = 196.54, 178.72, 171.66, 169.57, 144.10, 138.85, 136.83, 134.46, 133.91, 133.87, 129.94, 128.91, 128.67, 127.96, 122.32, 121.13, 119.87, 110.01, 74.82, 73.54, 61.59, 54.16, 51.11, 36.83, 36.35, 32.71, 29.18, 29.08, 29.02, 25.63, 25.56 ppm; HRMS (ESI-TOF) *m/z*: [M + Na]^+^ Calculated for C_36_H_39_ClN_4_NaO_5_S^+^ 697.2222, found 697.2227.

***N^1^-(4-((1'R,2'S,3R,7a'R)-2'-benzoyl-6-chloro-2-oxo-1',2',5',6',7',7a'-hexahydrospiro[indoline-3,3'-pyrrolizin]-1'-yl)phenyl)-N^8^-hydroxyoctanediamide (7t)***

Orange solid, 50% yield, dr > 25:1, m.p. 143.4-144.5 °C. ^1^H NMR (400 MHz, DMSO-*d*_6_): δ = 10.41 (s, 2H), 9.92 (s, 1H), 8.73 (br, 1H), 7.56–7.54 (m, 2H), 7.50 (d, *J* = 7.6 Hz, 1H), 7.43–7.31 (m, 6H), 7.26 (d, *J* = 8.4 Hz, 1H), 6.99 (d, *J* = 7.6 Hz, 1H), 6.57 (s, 1H), 4.85 (d, *J* = 11.6 Hz, 1H), 3.93-3.88 (m, 1H), 3.81 (t, *J* = 10.8 Hz, 1H), 2.55 (d, *J* = 8.0 Hz, 1H), 2.38-2.33 (m, 1H), 2.27 (t, *J* = 7.2 Hz, 2H), 1.95 (t, *J* = 7.6 Hz, 2H), 1.89-1.82 (m, 2H), 1.77–1.68 (m, 2H), 1.57–1.52 (m, 2H), 1.50–1.45 (m, 2H), 1.31–1.23 (m, 4H) ppm; 13C NMR (100 MHz, DMSO-*d*_6_): δ = 197.15, 179.76, 171.59, 169.58, 143.93, 138.52, 137.03, 134.55, 133.98, 133.77, 128.23, 128.90, 128.26, 127.92, 124.16, 121.23, 119.83, 110.08, 72.55, 71.83, 63.30, 52.27, 47.96, 36.76, 32.69, 29.95, 28.83, 27.22, 25.54, 25.48 ppm; HRMS (ESI-TOF) *m/z*: [M + Na]^+^ Calculated for C_35_H_37_ClN_4_ ${\text{NaO}}_{5}^{+}$ 651.2345, found 651.2347.

***N-(4-((3R,6'S,7'R,7a'S)-6'-benzoyl-6-chloro-2-oxo-1',6',7',7a'-tetrahydro-3'H-spiro[indoline-3,5'-pyrrolo[1,2-c]thiazol]-7'-yl)phenyl)-2-(2-(hydroxyamino)-2-oxoethoxy)acetamide (7u)***

Orange solid, 36% yield, dr > 10:1, m.p. 167.1-168.5 °C. ^1^H NMR (400 MHz, DMSO-*d*_6_): δ = 10.55 (s, 2H), 9.95 (s, 1H), 9.07 (br, 1H), 7.61–7.58 (m, 2H), 7.53–7.46 (m, 3H), 7.43–7.36 (m, 3H), 7.33–7.29 (m, 2H), 7.01 (dd, *J* = 8.0, 2.0 Hz, 1H), 6.50 (d, *J* = 2.0 Hz, 1H), 4.78 (d, *J* = 11.6 Hz, 1H), 4.15–4.07 (m, 3H), 4.02 (s, 2H), 3.84–3.78 (m, 1H), 3.70 (d, *J* = 10.4 Hz, 1H), 3.33 (s, 1H), 3.05–2.99 (m, 2H) ppm; ^13^C NMR (100 MHz, DMSO-*d*_6_): δ = 196.59, 178.70, 168.05, 165.77, 144.10, 137.66, 136.84, 134.86, 133.93, 129.96, 128.92, 128.75, 127.98, 122.31, 121.15, 120.91, 110.02, 74.81, 73.57, 71.07, 69.81, 61.60, 54.18, 51.13, 36.36 ppm; HRMS (ESI-TOF) *m/z*: [M + Na]^+^ Calculated for C_30_H_27_ClN_4_NaO_6_S^+^ 629.1232, found 629.1238.

***N^1^-(4-((4-((3R,6'S,7'R,7a'S)-6'-benzoyl-6-chloro-2-oxo-1',6',7',7a'-tetrahydro-3'H-spiro[indoline-3,5'-pyrrolo[1,2-c]thiazol]-7'-yl)phenyl)carbamoyl)phenyl)-N^6^-hydroxyadipamide (7v)***

Orange solid, 17% yield, dr > 16:1, m.p. 175.5-176.6 °C. ^1^H NMR (400 MHz, DMSO-*d*_6_): δ = 10.42 (s, 2H), 10.19 (s, 1H), 10.10 (s, 1H), 8.71 (br, 1H), 7.92–7.90 (m, 2H), 7.75–7.71 (m, 4H), 7.54–7.48 (m, 3H), 7.45–7.39 (m, 3H), 7.35–7.31 (m, 2H), 7.03 (dd, *J* = 8.0, 2.0 Hz, 1H), 6.52 (d, *J* = 2.0 Hz, 1H), 4.81 (d, *J* = 11.6 Hz, 1H), 4.15–4.11 (m, 1H), 3.83 (dd, *J* = 11.6 Hz, 9.2 Hz, 1H), 3.73 (d, *J* = 10.4 Hz, 1H), 3.08–3.02 (m, 2H), 2.35 (t, *J* = 6.4 Hz, 2H), 1.99 (t, *J* = 6.4 Hz, 2H), 1.62–1.52 (m, 4H) ppm; ^13^C NMR (100 MHz, DMSO-*d*_6_): δ = 196.59, 178.71, 172.02, 169.39, 165.26, 144.08, 142.73, 138.80, 136.85, 134.47, 133.93, 129.95, 129.34, 129.02, 128.93, 128.61, 127.97, 122.33, 121.10, 118.62, 110.00, 74.83, 73.56, 61.62, 54.16, 51.15, 36.71, 36.38, 32.61, 25.32, 25.14 ppm; HRMS (ESI-TOF) *m/z*: [M + Na]^+^ Calculated for C_39_H_36_ClN_5_NaO_6_S^+^ 760.1967, found 760.1968.

***N^1^-(4-((3R,6'S,7'R,7a'S)-6-chloro-6'-(3-methoxybenzoyl)-2-oxo-1',6',7',7a'-tetrahydro-3'H-spiro[indoline-3,5'-pyrrolo[1,2-c]thiazol]-7'-yl)phenyl)-N^6^-hydroxyadipamide (7w)***

Orange solid, 40% yield, dr > 99:1, m.p. 141.8-143.1 °C. ^1^H NMR (400 MHz, DMSO-*d*_6_): δ = 9.91 (s, 1H), 7.57–7.55 (m, 2H), 7.45–7.40 (m, 3H), 7.22 (t, *J* = 7.6 Hz, 1H), 7.06 (d, *J* = 8.0 Hz, 1H), 7.03–6.97 (m, 2H), 6.85 (m, 1H), 6.54 (m, 1H), 4.74 (d, *J* = 11.6 Hz, 1H), 4.11–4.07 (m, 1H), 3.80–3.75 (m, 2H), 3.71 (s, 3H), 3.37 (d, *J* = 10.4 Hz, 1H), 3.05–2.98 (m, 2H), 2.28 (t, *J* = 6.8 Hz, 2H), 1.98 (t, *J* = 6.8 Hz, 2H), 1.57–1.49 (m, 4H) ppm; ^13^C NMR (100 MHz, DMSO-*d*_6_): δ = 196.44, 178.96, 171.51, 169.43, 159.42, 144.40, 138.79, 138.21, 134.48, 133.95, 130.02, 129.84, 128.72, 122.42, 121.09, 120.36, 120.30, 119.89, 112.15, 110.06, 74.79, 73.58, 61.82, 55.55, 54.25, 51.14, 36.62, 36.39, 32.61, 25.33, 25.29 ppm; HRMS (ESI-TOF) *m/z*: [M + Na]^+^ Calculated for C_33_H_33_ClN_4_NaO_6_S^+^ 671.1702, found 671.1694.

***N^1^-(4-((3R,6'S,7'R,7a'S)-6-chloro-6'-(4-fluorobenzoyl)-2-oxo-1',6',7',7a'-tetrahydro-3'H-spiro[indoline-3,5'-pyrrolo[1,2-c]thiazol]-7'-yl)phenyl)-N^6^-hydroxyadipamide (7x)***

Orange solid, 34% yield, dr > 13:1, m.p. 218.2-219.6°C. ^1^H NMR (400 MHz, DMSO-*d*_6_): δ = 10.43 (s, 2H), 9.90 (s, 1H), 8.73 (, 1H), 7.57–7.55 (m, 2H), 7.48–7.42 (m, 5H), 7.19–7.15 (m, 2H), 7.02 (d, *J* = 8.0 Hz, 1H), 6.55 (s, 1H), 4.76 (d, *J* = 11.6 Hz, 1H), 4.11–4.06 (m, 1H), 3.77 (t, *J* = 10.8 Hz, 1H), 3.72 (d, *J* = 10.4 Hz, 1H), 3.34 (d, *J* = 10.4 Hz, 1H), 3.05–2.99 (m, 2H), 2.28 (t, *J* = 6.8 Hz, 2H), 1.98 (t, *J* = 6.8 Hz, 2H), 1.57–1.49 (m, 4H) ppm; ^13^C NMR (100 MHz, DMSO-*d*_6_): δ = 195.24, 178.67, 171.48, 169.38, 164.22, 144.09, 138.81, 134.52, 133.79, 133.61, 131.05, 130.96, 129.92, 128.71, 122.22, 121.15, 119.86, 116.11, 115.89, 110.03, 74.85, 73.64, 61.62, 54.26, 51.02, 36.61, 36.35, 32.59, 25.31, 25.28 ppm; HRMS (ESI-TOF) *m/z*: [M + H]^+^ Calculated for C_32_H_31_ClFN_4_O_5_S^+^ 637.1682, found 637.1682.

***N^1^-(4-((3R,6'S,7'R,7a'S)-6-chloro-6'-(4-(dimethylamino)benzoyl)-2-oxo-1',6',7',7a'-tetrahydro-3'H-spiro[indoline-3,5'-pyrrolo[1,2-c]thiazol]-7'-yl)phenyl)-N^6^-hydroxyadipamide (7y)***

Orange solid, 37% yield, dr > 25:1, m.p. 178.2-179.9°C. ^1^H NMR (400 MHz, DMSO-*d*_6_): δ = 9.87 (s, 1H), 7.53–7.47 (m, 3H), 7.41–7.34 (m, 4H), 6.99 (dd, *J* = 8.0 Hz, 2.0 Hz, 1H), 6.62 (d, *J* = 2.0 Hz, 1H), 6.54–6.52 (m, 2H), 4.66 (d, *J* = 12.0 Hz, 1H), 4.11–4.06 (m, 1H), 3.82 (dd, *J* = 12.0 Hz, 9.2 Hz, 1H), 3.72 (d, *J* = 10.4 Hz, 1H), 3.35 (d, *J* = 10.4 Hz, 1H), 3.05–2.99 (m, 2H), 2.94 (s, 6H), 2.26 (t, *J* = 7.2 Hz, 2H), 1.96 (t, *J* = 6.8 Hz, 2H), 1.58–1.48 (m, 4H) ppm; ^13^C NMR (100 MHz, DMSO-*d*_6_): δ = 192.16, 179.05, 171.46, 169.40, 153.78, 144.02, 138.71, 134.19, 134.10, 130.37, 128.55, 124.31, 122.71, 120.93, 119.83, 110.90, 109.89, 74.77, 73.99, 60.12, 54.15, 51.60, 36.60, 36.39, 32.60, 25.32, 25.28 ppm; HRMS (ESI-TOF) *m/z*: [M + Na]^+^ Calculated for C_34_H_36_ClN_5_NaO_5_S^+^ 684.2018, found 684.2008.

***N^1^-(2-aminophenyl)-N^4^-(4-((3R,6'S,7'R,7a'S)-6'-benzoyl-2-oxo-1',6',7',7a'-tetrahydro-3'H-spiro[indoline-3,5'-pyrrolo[1,2-c]thiazol]-7'-yl)phenyl)succinamide (9a)***

White sold, 19% yield, dr > 99:1, m.p. 149.1-150.2 °C. ^1^H NMR (400 MHz, DMSO-*d*_6_): δ = 10.34 (s, 1H), 10.05 (s, 1H), 9.25 (s, 1H), 7.59–7.57 (m, 2H), 7.49–7.39 (m, 4H), 7.36–7.34 (m, 2H), 7.30–7.26 (m, 2H), 7.15-7.09 (m, 2H), 6.93 (t, *J* = 7.6 Hz, 1H), 6.88 (t, *J* = 7.6 Hz, 1H), 6.70 (d, *J* = 7.6 Hz, 1H), 6.53–6.49 (m, 2H), 4.90–4.88 (m, 2H), 4.77 (d, *J* = 11.6 Hz, 1H), 4.14–4.09 (m, 1H), 3.84 (dd, *J* = 11.6 Hz, 9.2 Hz, 1H), 3.71 (d, *J* = 10.0 Hz, 1H), 3.33 (d, *J* = 10.0 Hz, 1H), 3.06–2.98 (m, 2H), 2.65 (s, 4H) ppm; 13C NMR (100 MHz, DMSO-*d*_6_): δ = 196.69, 178.81, 170.97, 170.84, 142.70, 142.52, 138.76, 136.99, 134.18, 133.67, 130.18, 128.79, 128.68, 128.39, 127.92, 126.28, 126.02, 123.74, 123.47, 121.40, 119.87, 116.38, 116.04, 109.96, 74.84, 73.69, 61.82, 53.91, 50.95, 36.25, 32.16, 31.32 ppm; HRMS (ESI-TOF) *m/z*: [M + Na]^+^ Calculated for C_36_H_33_N_5_NaO_4_S^+^ 654.2145, found 654.2150.

***N^1^-(2-aminophenyl)-N^8^-(4-((3R,6'S,7'R,7a'S)-6'-benzoyl-2-oxo-1',6',7',7a'-tetrahydro-3'H-spiro[indoline-3,5'-pyrrolo[1,2-c]thiazol]-7'-yl)phenyl)octanediamide (9b)***

White yield, 45% yield, dr > 99:1, m.p. 131.7-132.9 °C. ^1^H NMR (400 MHz, DMSO-*d*_6_): δ = 10.35 (s, 1H), 9.95 (s, 1H), 9.20 (s, 1H), 7.60–7.58 (m, 2H), 7.48 (t, *J* = 7.2 Hz, 1H), 7.46–7.39 (m, 3H), 7.36–7.33 (m, 2H), 7.30–7.26 (m, 2H), 7.17 (d, *J* = 7.6 Hz, 1H), 7.11 (t, *J* = 7.6 Hz, 1H), 6.93 (t, *J* = 7.6 Hz, 1H), 6.88 (t, *J* = 7.6 Hz, 1H), 6.71 (d, *J* = 8.0 Hz, 1H), 6.55–6.49 (m, 2H), 4.85 (s, 2H), 4.77 (d, *J* = 11.6 Hz, 1H), 4.13–4.09 (m, 1H), 3.83 (dd, *J* = 11.2 Hz, 9.2 Hz, 1H), 3.71 (d, *J* = 10.4 Hz, 1H), 3.33 (d, *J* = 10.0 Hz, 1H), 3.05–3.97 (m, 2H), 2.34–2.28 (m, 4H), 1.63–1.56 (m, 4H), 1.35–1.32 (m, 4H) ppm; ^13^C NMR (100 MHz, DMSO-*d*_6_): δ = 196.67, 178.80, 171.67, 171.64, 142.52, 142.28, 138.84, 136.99, 134.11, 133.67, 130.17, 128.79, 128.65, 128.40, 127.91, 126.04, 125.69, 124.10, 123.45, 121.39, 119.91, 116.58, 116.33, 109.96, 74.84, 73.70, 61.80, 53.94, 50.98, 36.82, 36.25, 36.22, 28.95, 28.91, 25.71, 25.59 ppm; HRMS (ESI-TOF) *m/z*: [M + Na]^+^ Calculated for C_40_H_41_N_5_NaO_4_S^+^ 710.2771, found 710.2772.

***N^1^-(2-aminophenyl)-N^6^-(4-((3R,6'S,7'R,7a'S)-6'-benzoyl-6-chloro-2-oxo-1',6',7',7a'-tetrahydro-3'H-spiro[indoline-3,5'-pyrrolo[1,2-c]thiazol]-7'-yl)phenyl)adipamide (9c)***

White yield, 24% yield, dr > 20:1, m.p. 148.9-150.0 °C. ^1^H NMR (400 MHz, DMSO-*d*_6_): δ = 10.53 (s, 1H), 9.99 (s, 1H), 9.21 (s, 1H), 7.60–7.58 (m, 2H), 7.52 (t, *J* = 7.2 Hz, 1H), 7.45–7.39 (m, 5H), 7.34–7.31 (m, 2H), 7.17 (d, *J* = 8.0 Hz, 1H), 7.02 (dd, *J* = 8.0, 2.0 Hz, 1H), 6.88 (t, *J* = 7.6 Hz, 1H), 6.72 (d, *J* = 8.0 Hz, 1H), 6.55–6.51 (m, 2H), 4.86 (s, 2H), 4.78 (d, *J* = 11.6 Hz, 1H), 4.13–4.09 (m, 1H), 3.80 (dd, *J* = 11.6 Hz, 9.2 Hz, 1H), 3.72 (d, *J* = 10.4 Hz, 1H), 3.36 (d, *J* = 10.4 Hz, 1H), 3.06–2.98 (m, 2H), 2.38–2.33 (m, 4H), 1.66-1.60 (m, 4H) ppm; ^13^C NMR (100 MHz, DMSO-*d*_6_): δ = 196.56, 178.72, 171.57, 171.49, 144.12, 142.31, 138.86, 136.84, 134.46, 133.91, 129.94, 128.92, 128.67, 127.97, 126.09, 125.73, 124.02, 122.33, 121.13, 119.92, 116.57, 116.32, 110.03, 74.82, 73.56, 61.59, 54.17, 51.13, 36.66, 36.36, 36.03, 25.46, 25.35 ppm; HRMS (ESI-TOF) *m/z*: [M + Na]^+^ Calculated for C_38_H_36_ClN_5_NaO_4_S^+^ 716.2069, found 716.2070.

***N^1^-(2-aminophenyl)-N^8^-(4-((3R,6'S,7'R,7a'S)-6'-benzoyl-6-chloro-2-oxo-1',6',7',7a'-tetrahydro-3'H-spiro[indoline-3,5'-pyrrolo[1,2-c]thiazol]-7'-yl)phenyl)octanediamide (9d)***

White yield, 30% yield, dr > 99:1, m.p. 145.2-146.4 °C. ^1^H NMR (400 MHz, DMSO-*d*_6_): δ = 10.53 (s, 1H), 9.92 (s, 1H), 9.16 (s, 1H), 7.59–7.57 (m, 2H), 7.52 (t, *J* = 7.6 Hz, 1H), 7.44–7.39 (m, 5H), 7.34–7.31 (m, 2H), 7.17 (d, *J* = 7.6 Hz, 1H), 7.02 (dd, *J* = 8.0 Hz, 2.0 Hz, 1H), 6.88 (t, *J* = 7.6 Hz, 1H), 6.72 (d, *J* = 7.6 Hz, 1H), 6.55-6.51 (m, 2H), 4.84 (s, 2H), 4.78 (d, *J* = 11.6 Hz, 1H), 4.13–4.08 (m, 1H), 3.80 (dd, *J* = 11.2 Hz, 9.2 Hz, 1H), 3.72 (d, *J* = 10.4 Hz, 1H), 3.36 (d, *J* = 10.4 Hz, 1H), 3.06–2.98 (m, 2H), 2.34–2.27 (m, 4H), 1.63–1.56 (m, 4H), 1.35–1.32 (m, 4H) ppm; ^13^C NMR (100 MHz, DMSO-*d*_6_): δ = 196.54, 178.70, 171.65, 171.62, 144.08, 142.29, 138.87, 136.83, 134.46, 133.91, 133.85, 129.93, 128.91, 128.66, 127.95, 126.07, 125.70, 124.07, 122.31, 121.13, 119.89, 116.59, 116.33, 110.01, 74.81, 73.54, 61.58, 54.17, 51.12, 36.82, 36.35, 36.21, 28.94, 28.91, 25.70, 25.58 ppm; HRMS (ESI-TOF) *m/z*: [M + Na]^+^ Calculated for C_40_H_40_ClN_5_NaO_4_S^+^ 744.2382, found 744.2386.

***N^1^-(2-aminophenyl)-N^10^-(4-((3R,6'S,7'R,7a'S)-6'-benzoyl-6-chloro-2-oxo-1',6',7',7a'-tetrahydro-3'H-spiro[indoline-3,5'-pyrrolo[1,2-c]thiazol]-7'-yl)phenyl)decanediamide (9e)***

White solid, 36% yield, dr > 99:1, m.p. 132.4-133.9 °C. ^1^H NMR (400 MHz, DMSO-*d*_6_): δ = 10.52 (s, 1H), 9.91 (s, 1H), 9.16 (s, 1H), 7.58–7.56 (m, 2H), 7.52 (t, *J* = 7.6 Hz, 1H), 7.44–7.39 (m, 5H), 7.34–7.30 (m, 2H), 7.16 (d, *J* = 7.2 Hz, 1H), 7.02 (dd, *J* = 8.0 Hz, 2.0 Hz, 1H), 6.88 (t, *J* = 7.2 Hz, 1H), 6.71 (d, *J* = 7.6 Hz, 1H), 6.55–6.51 (m, 2H), 4.84 (s, 2H), 4.77 (d, *J* = 11.6 Hz, 1H), 4.13–4.08 (m, 1H), 3.79 (dd, *J* = 11.6 Hz, 9.2 Hz, 1H), 3.72 (d, *J* = 10.4 Hz, 1H), 3.36 (d, *J* = 10.8 Hz, 1H), 3.06–2.98 (m, 2H), 2.33–2.26 (m, 4H), 1.60-1.56 (m, 4H), 1.29 (s, 8H) ppm; 13C NMR (100 MHz, DMSO-*d*_6_): δ = 196.54, 178.71, 171.68, 171.66, 144.09, 142.29, 138.87, 136.83, 134.46, 133.91, 133.85, 129.94, 128.91, 128.66, 127.96, 126.07, 125.68, 122.32, 121.14, 119.88, 116.61, 116.35, 110.01, 74.82, 73.55, 61.59, 54.16, 51.12, 36.84, 36.36, 36.24, 29.20, 29.18, 29.12, 29.09, 25.78, 25.65 ppm; HRMS (ESI-TOF) *m/z*: [M + Na]^+^ Calculated for C_42_H_44_ClN_5_NaO_4_S^+^ 772.2695, found 772.2700.

***N-(4-((3R,6'S,7'R,7a'S)-6'-benzoyl-6-chloro-2-oxo-1',6',7',7a'-tetrahydro-3'H-spiro[indoline-3,5'-pyrrolo[1,2-c]thiazol]-7'-yl)phenyl)-1-(6-(hydroxyamino)-6-oxohexanoyl)piperidine-4-carboxamide (11a)***

Orange solid, 24% yield, dr > 16:1, m.p. 170.3-171.5 °C. ^1^H NMR (400 MHz, DMSO-*d*_6_): δ = 10.43 (s, 2H), 9.92 (s, 1H), 8.71 (s, 1H), 7.58–7.56 (m, 2H), 7.52 (t, *J* = 7.2 Hz, 1H), 7.45–7.38 (m, 5H), 7.34–7.31 (m, 2H), 7.02 (d, *J* = 8.0 Hz, 1H), 6.52 (s, 1H), 4.78 (d, *J* = 11.6 Hz, 1H), 4.41 (d, *J* = 12.4 Hz, 1H), 4.12–4.08 (m, 1H), 3.91 (d, *J* = 13.2 Hz, 1H), 3.80 (dd, *J* = 11.6 Hz, 9.2 Hz, 1H), 3.72 (d, *J* = 10.4 Hz, 1H), 3.35 (s, 1H), 3.05-2.98 (m, 3H), 2.33–2.54 (m, 2H), 2.33–2.26 (m, 2H), 1.99–1.95 (m, 2H), 1.81–1.75 (m, 2H), 1.58–1.42 (m, 6H) ppm; ^13^C NMR (100 MHz, DMSO-*d*_6_): δ = 196.53, 178.71, 173.35, 170.74, 169.46, 144.07, 138.78, 136.83, 134.47, 134.05, 133.93, 129.95, 128.92, 128.71, 127.96, 122.31, 121.15, 119.98, 110.00, 74.81, 73.55, 61.61, 54.17, 51.12, 44.90, 43.14, 40.97, 36.34, 32.59, 32.50, 29.34, 28.65, 25.37, 24.91 ppm; HRMS (ESI-TOF) *m/z*: [M + Na]^+^ Calculated for C_38_H_40_ClN_5_NaO_6_S^+^ 752.2280, found 752.2284.

***N-(4-((3R,6'S,7'R,7a'S)-6'-benzoyl-6-chloro-2-oxo-1',6',7',7a'-tetrahydro-3'H-spiro[indoline-3,5'-pyrrolo[1,2-c]thiazol]-7'-yl)phenyl)-1-(8-(hydroxyamino)-8-oxooctanoyl)piperidine-4-carboxamide (11b)***

Orange solid, 31% yield, dr > 17:1, m.p. 137.3-138.6 °C. ^1^H NMR (400 MHz, DMSO-*d*_6_): δ = 10.47 (s, 1H), 10.32 (s, 1H), 9.91 (s, 1H), 8.64 (s, 1H), 7.56–7.54 (m, 2H), 7.50 (t, *J* = 7.2 Hz, 1H), 7.43–7.36 (m, 5H), 7.33–7.29 (m, 2H), 7.00 (dd, *J* = 8.0, 1.6 Hz, 1H), 6.50 (d, *J* = 2.0 Hz, 1H), 4.75 (d, *J* = 12.0 Hz, 1H), 4.39 (d, *J* = 12.8 Hz, 1H), 4.10–4.05 (m, 1H), 3.89 (d, *J* = 13.2 Hz, 1H), 3.78 (dd, *J* = 11.6 Hz, 9.2 Hz, 1H), 3.70 (d, *J* = 10.4 Hz, 1H), 3.04–3.99 (m, 2H), 2.55 (t, *J* = 12.8 Hz, 2H), 2.29–2.25 (m, 2H), 1.94–1.90 (m, 2H), 1.79–1.73 (m, 2H), 1.50–1.42 (m, 4H), 1.29–1.20 (m, 8H) ppm; ^13^C NMR (100 MHz, DMSO-*d*_6_): δ = 196.51, 178.68, 173.33, 170.85, 169.58, 144.05, 138.76, 136.81, 134.44, 134.01, 133.91, 129.92, 128.90, 128.67, 127.93, 122.29, 121.12, 119.95, 109.98, 74.79, 73.52, 61.58, 54.13, 51.09, 44.89, 43.10, 40.92, 36.30, 32.75, 32.69, 29.32, 28.95, 28.89, 28.64, 25.49, 25.23 ppm; HRMS (ESI-TOF) *m/z*: [M + Na]^+^ Calculated for C_40_H_44_ClN_5_NaO_6_S^+^ 780.2593, found 780.2596.

***N^1^-(4-((3R,3'S,4'R)-3'-benzoyl-6-chloro-1'-methyl-2-oxospiro[indoline-3,2'-pyrrolidin]-4'-yl)phenyl)-N^8^-hydroxyoctanediamide (13a)***

Orange solid, 50% yield, dr > 20:1, m.p. 126.7-127.9 °C. ^1^H NMR (400 MHz, DMSO-*d*_6_): δ = 10.47 (s, 2H), 9.96 (s, 1H), 8.76 (s, 1H), 7.57–7.55 (m, 2H), 7.47 (t, *J* = 7.6 Hz, 1H), 7.40–7.38 (m, 2H), 7.34–7.28 (m, 4H), 6.95–6.88 (m, 2H), 6.51 (s, 1H), 4.42 (d, *J* = 9.2 Hz, 1H), 4.29 (dd, *J* = 16.8 Hz, 9.2 Hz, 1H), 3.42 (d, *J* = 8.8 Hz, 1H), 3.35 (d, *J* = 8.0 Hz, 1H), 2.28 (t, *J* = 7.2 Hz, 2H), 2.07 (s, 3H), 1.95 (t, *J* = 7.2 Hz, 2H), 1.59–1.54 (m, 2H), 1.50–1.45 (m, 2H), 1.31–1.23 (m, 4H) ppm; ^13^C NMR (100 MHz, DMSO-*d*_6_): δ = 197.54, 179.33, 171.61, 169.59, 144.05, 138.56, 136.97, 135.81, 133.72, 128.93, 128.25, 127.91, 127.65, 126.23, 121.91, 119.79, 109.71, 72.75, 62.07, 60.18, 44.07, 36.75, 34.91, 32.69, 28.84, 25.50 ppm; HRMS (ESI-TOF) *m/z*: [M + Na]^+^ Calculated for C_33_H_35_ClN_4_ ${\text{NaO}}_{5}^{+}$ 625.2188, found 625.2188.

***N^1^-(4-((3R,3'S,4'R,5'S)-3'-benzoyl-6-chloro-2-oxo-5'-phenylspiro[indoline-3,2'-pyrrolidin]-4'-yl)phenyl)-N^8^-hydroxyoctanediamide (15a)***

Orange solid, 15% yield, dr > 10:1, m.p. 146.5-147.6 °C. ^1^H NMR (400 MHz, DMSO-*d*_6_): δ = 10.38 (s, 2H), 9.81 (s, 1H), 8.86 (br, 1H), 7.47–7.45 (m, 2H), 7.40–7.38 (m, 2H), 7.34–7.16 (m, 11H), 6.94 (d, *J* = 8.4 Hz, 1H), 6.45 (s, 1H), 4.94 (dd, *J* = 10.4 Hz, 4.4 Hz, 1H), 4.57 (d, *J* = 10.4 Hz, 1H), 4.08 (d, *J* = 4.8 Hz, 1H), 3.94 (t, *J* = 10.4 Hz, 1H), 2.23 (t, *J* = 7.2 Hz, 2H), 1.92 (t, *J* = 7.2 Hz, 2H), 1.53 (t, *J* = 6.8 Hz, 2H), 1.47 (t, *J* = 6.8 Hz, 2H), 1.26–1.23 (m, 4H) ppm; ^13^C NMR (100 MHz, DMSO-*d*_6_): δ = 197.47, 182.21, 171.55, 169.52, 143.46, 141.87, 138.50, 137.05, 133.95, 133.72, 133.32, 129.70, 128.91, 128.80, 128.50, 127.92, 127.79, 127.63, 127.59, 121.73, 119.63, 109.47, 67.56, 67.27, 62.31, 55.25, 36.76, 32.69, 28.84, 25.49 ppm; HRMS (ESI-TOF) *m/z*: [M + Na]^+^ Calculated for C_38_H_37_ClN_4_ ${\text{NaO}}_{5}^{+}$ 687.2345, found 687.2342.

***N^1^-(4-((3R,6'S,7'R,7a'S)-6-chloro-6'-(furan-2-carbonyl)-2-oxo-1',6',7',7a'-tetrahydro-3'H-spiro[indoline-3,5'-pyrrolo[1,2-c]thiazol]-7'-yl)phenyl)-N^6^-hydroxyadipamide (15b)***

Orange solid, 34% yield, dr > 25:1, m.p. 151.3-152.4 °C. ^1^H NMR (400 MHz, DMSO-*d*_6_): δ = 10.38 (s, 1H), 9.88 (s, 1H), 8.86 (br, 1H), 7.83 (s, 1H), 7.55–7.53 (m, 2H), 7.46 (d, *J* = 8.0 Hz, 1H), 7.39–7.37 (m, 2H), 7.16 (d, *J* = 3.2 Hz, 1H), 7.01 (d, *J* = 8.0 Hz, 1H), 6.70 (s, 1H), 6.59 (s, 1H), 4.45 (d, *J* = 11.6 Hz, 1H), 4.11–4.06 (m, 1H), 3.78 (dd, *J* = 11.6 Hz, 9.2 Hz, 1H), 3.72 (d, *J* = 10.4 Hz, 1H), 3.38 (d, *J* = 10.4 Hz, 1H), 3.05–2.96 (m, 2H), 2.29–2.26 (m, 2H), 1.99–1.96 (m, 2H), 1.57–1.50 (m, 4H) ppm; ^13^C NMR (100 MHz, DMSO-*d*_6_): δ = 182.78, 178.86, 171.49, 169.43, 151.78, 149.24, 144.06, 138.89, 134.51, 133.54, 130.12, 128.65, 122.29, 121.20, 120.19, 119.90, 113.12, 110.17, 74.64, 73.83, 61.91, 53.90, 50.60, 36.63, 36.16, 32.61, 25.31, 25.30 ppm; HRMS (ESI-TOF) *m/z*: [M + Na]^+^ Calculated for C_30_H_29_ClN_4_NaO_6_S^+^ 631.1389, found 631.1383.

***N^1^-(4-((3R,6'S,7'R,7a'S)-6-chloro-2-oxo-6'-(thiophene-2-carbonyl)-1',6',7',7a'-tetrahydro-3'H-spiro[indoline-3,5'-pyrrolo[1,2-c]thiazol]-7'-yl)phenyl)-N^6^-hydroxyadipamide (15c)***

Orange solid, 36% yield, dr > 20:1, m.p. 151.6-153.8 °C. ^1^H NMR (400 MHz, DMSO-*d*_6_): δ = 10.49 (s, 2H), 9.86 (s, 1H), 8.67 (s, 1H), 7.89 (d, *J* = 4.8 Hz, 1H), 7.63 (d, *J* = 3.6 Hz, 1H), 7.54–7.52 (m, 2H), 7.47 (d, *J* = 8.4 Hz, 1H), 7.40–7.38 (m, 2H), 7.12 (t, *J* = 4.4 Hz, 1H), 7.03 (dd, *J* = 8.0 Hz, 1H), 6.63 (d, *J* = 2.0 Hz, 1H), 4.60 (d, *J* = 12.0 Hz, 1H), 4.11–4.06 (m, 1H), 3.77 (dd, *J* = 11.6 Hz, 9.2 Hz, 1H), 3.72 (d, *J* = 10.8 Hz, 1H), 3.38 (d, *J* = 10.8 Hz, 1H), 3.05–2.97 (m, 2H), 2.26 (t, *J* = 6.4 Hz, 2H), 1.95 (t, *J* = 6.4 Hz, 2H), 1.52–1.49 (m, 4H) ppm; ^13^C NMR (100 MHz, DMSO-*d*_6_): δ = 188.07, 179.08, 171.55, 169.49, 144.25, 143.85, 138.89, 136.78, 133.53, 130.19, 129.10, 128.67, 122.33, 121.18, 119.93, 110.22, 74.72, 74.20, 62.36, 54.25, 51.13, 36.61, 36.32, 32.61, 25.32, 25.30 ppm; HRMS (ESI-TOF) *m/z*: [M + Na]^+^ Calculated for C_30_H_29_ClN_4_NaO_5_ ${\text{S}}_{2}^{+}$ 647.1160, found 647.1161.

### 3.2 Biological evaluation

#### 3.2.1 Enzyme inhibition assay

The enzymatic assay of test compounds is using the HTRF-based method provided by Cisbio Co. Ltd. In brief, the target compounds to be tested were prepared into samples with a concentration of 1 μM, which was added to GST-tagged MDM2 and HDAC1 proteins, respectively, then biotinylated substrate p53 peptide and HTRF detection reagents were added. The data obtained were processed by the ratio method, and the inhibition rates of MDM2 and HDAC1 were calculated. The detailed experimental procedures are according to the manufacturer’s protocols.

#### 3.2.2 Cell culture and cell viability assay

The human epidermal cancer cells A431, human breast cancer cells MCF-7, and human colorectal cancer cells HCT116 were all derived from Chinese Center for Type Culture Collection (Wuhan, China) and cultured in our laboratory, cells were incubated with Dulbecco’s modified Eagle’s medium (DMEM) containing 10% fetal bovine serum (FBS) in a humidified atmosphere including 5% CO_2_ under a sterile condition at 37°C (v/v) (GIBCO, Waltham, MA, USA). The cytotoxicity assay was performed with MTT method as previously described (73, 88, 89).

#### 3.2.3 Cell apoptosis analysis using TUNEL staining assay

TUNEL assay was performed using *in situ* apoptosis detection kit (Promega, USA), according to the manufacturer’s protocols. Briefly, MCF-7 cells were seeded in 6-well plates for 12 h, and then treated with compound SAHA, Nutlin-3, 11b (0.5 μM, respectively) or vehicle (DMSO). After 24 h incubation, cells were plated onto the slides, fixed in 4% paraformaldehyde in PBS for 25 min, and then washed with PBS twice. After that, the cells were incubated in TUNEL reaction mixture at 37°C for 60 min. Then nuclei were counterstained with DAPI, and cells were checked under an immunofluorescent confocal microscopy (Olympus FV1000).

#### 3.2.3 Cell apoptosis analysis using annexin V-FITC/PI double staining

MCF-7 cells were seeded in 6-well plates for 12 h, and then treated with compound SAHA, Nutlin-3, 11b (5 μM, respectively) or vehicle (DMSO) for 24 h. Cells were collected after incubation, then fixed with 75% ice-cold ethanol and stored at -20°C for 1 h. The cells were then harvested with 0.25% trypsin (without EDTA) and centrifuged at 2000 rpm for 5min. Then, the harvested cells were washed with ice-cold PBS twice and resuspended in 500 μL of 1× binding buffer, which was then added to 5 μL of annexin V-FITC and incubated at room temperature in the dark for 15 min. After adding 10 μL of propidium iodide (PI), the cells were incubated at room temperature in the dark. After another 15 min incubation, the cells were collected and analyzed by flow cytometry.

#### 3.2.5 Molecular docking study

The crystal structures of MDM2 and HDAC1 were obtained from PDB (Protein Data Bank, http://www.pdb.org) database. Water molecules and cocrystallized ligands were removed from the proteins and the polar hydrogens were added to the protein structure. The molecular docking study of 11b was carried out *via* Discovery Studio 3.5 according to the previously reported protocol (90, 91).

#### 3.2.6 Western blot assay

MCF-7 cells were seeded in 6-well plates for 24 h and treated with compounds SAHA, Nutlin-3, 11b (5 μM, respectively) or DMSO for 24 h. The cells were collected and washed with cold PBS twice, then added the protease and phosphatase inhibitors contained lysis buffer. Total cell lysates were prepared in lysis RIPA buffer (Invitrogen, CA, USA) on ice for 30 min, followed by centrifugation at 12000 rpm for 10 min at 4°C. After collecting supernatant, protein concentration was determined by a BCA protein assay. The total proteins were loaded into the 10%-15% SDS-PAGE gel and separated by sodium dodecyl sulfate polyacrylamide gel electrophoresis (PAGE). After electrophoresis, the protein was transferred to the PVDF membranes and rinsed three times in PBST buffer for 10 min each time, then sealed with 5% BSA Buffer for 1h. The membranes were incubated at 4°C for 24 h with primary antibodies and washed with TBST three times. Then incubated at room temperature for 2 h with secondary antibodies (1:10,000). The incubated PVDF membranes were taken out and rinsed with TBST three times. The protein bands were visualized through enhanced chemiluminescence (ECL) WB substrate (Millipore, MA, USA) and analyzed by ChemiScope. The antibodies including p53, MDM2, H4, and GAPDH were purchased from Abcam (Cambridge, MA, USA).

## 4 Conclusions

In summary, a series of MDM2-HDAC dual small-molecule inhibitors derivatives were designed and synthesized on the basis of the pharmacophore hybrid strategy. Among the target compounds, one spirooxindole-based hydroxamic acid 11b exhibited comparable inhibitory activities among MDM2 and HDAC, it also possessed good *in vitro* activity toward the tested three cancer cells, especially MCF-7, reaching the IC_50_ value of 1.37 ± 0.45 μM. Moreover, compound 11b exhibited remarkable selectivity for HDAC1 and HDAC2, with nanomolar IC_50_ of 58 nM and 64 nM, respectively. Further antitumor mechanism studies indicated that compound 11b could significantly induce the apoptosis of MCF-7 cells, and exerted potent anticancer effects. According to the western blot analysis, we demonstrated the most active compound 11b effectively and simultaneously inhibited MDM2 and HDAC through up-regulating the expression of p53 and Ac-H4 in MCF-7 cancer cells in a dose-dependent manner. These results highlighted that the simultaneous blockade of MDM2 and HDAC proteins with a single small-molecule compound represents a promising method for the discovery of new anticancer drugs.

## Data availability statement

The original contributions presented in the study are included in the article/**Supplementary Material**. Further inquiries can be directed to the corresponding authors.

## Author contributions

GH and BH conceived and designed the experiments, QZ, S-SX, and CC performed the experiments, S-SX, H-PZ, QZ, and XX analyzed the data, CP, QZ, and BH wrote the manuscript. All authors have read and agreed to the published version of the manuscript.

## Funding

This research was funded by the National Natural Science Foundation of China (82073998, 22107015, 22177084 and 82104376), Sichuan Science and Technology Program (2022JDRC0045 and 2022JDRC0125), and the China Postdoctoral Science Foundation.

## Conflict of interest

The authors declare that the research was conducted in the absence of any commercial or financial relationships that could be construed as a potential conflict of interest.

## Publisher’s note

All claims expressed in this article are solely those of the authors and do not necessarily represent those of their affiliated organizations, or those of the publisher, the editors and the reviewers. Any product that may be evaluated in this article, or claim that may be made by its manufacturer, is not guaranteed or endorsed by the publisher.
