# Corrigendum: Discovery of spirooxindole-derived small-molecule compounds as novel HDAC/MDM2 dual inhibitors and investigation of their anticancer activity

**DOI:** 10.3389/fonc.2023.1259550

**Published:** 2023-08-25

**Authors:** Qian Zhao, Shan-Shan Xiong, Can Chen, Hong-Ping Zhu, Xin Xie, Cheng Peng, Gu He, Bo Han

**Affiliations:** ^1^ State Key Laboratory of Southwestern Chinese Medicine Resources, Hospital of Chengdu University of Traditional Chinese Medicine, School of Basic Medical Sciences, Chengdu University of Traditional Chinese Medicine, Chengdu, China; ^2^ Department of Dermatology and State Key Laboratory of Biotherapy, West China Hospital, Sichuan University, Chengdu, China; ^3^ School of Pharmacy, Chengdu Medical College, Chengdu, China; ^4^ The First Affiliated Hospital, Chengdu Medical College, Chengdu, China; ^5^ Antibiotics Research and Re-evaluation Key Laboratory of Sichuan Province, Sichuan Industrial Institute of Antibiotics, Chengdu University, Chengdu, China

**Keywords:** multitarget drugs, histone deacetylase inhibitors, MDM2 inhibitors, spirooxindole, dual inhibitors, anticancer

In the published article, there was an error in **Table 1** **(Effects of substituted group R1 and R2 on enzyme inhibition)** as published. The figures A-C insert in **Table 1** were found to be incorrect. The corrected **Table 1** and its caption appear below.

**Table 1:** Effects of substituted group R^1^ and R^2^ on enzyme inhibition.

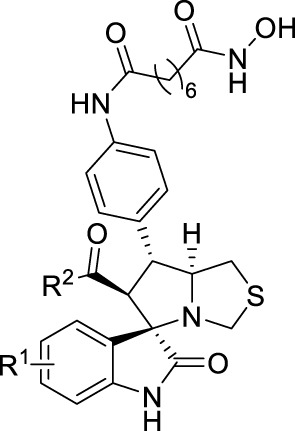				
compound	R^1^	R^2^	% enzyme inhibition	
			MDM2	HDAC
**7a**	H	Ph	39	69
**7b**	5-Me	Ph	45	71
**7c**	5-F	Ph	51	68
**7d**	5-Cl	Ph	49	67
**7e**	5-Br	Ph	48	72
**7f**	6-Cl	Ph	65	64
**7g**	6-Br	Ph	59	68
**7h**	7-Me	Ph	23	71
**7i**	7-Br	Ph	30	67
**7j**	5-OMe	Ph	50	70
**7k**	6-OMe	Ph	54	70
**7l**	H	3-OMe-C_6_H_4_	44	62
**7m**	H	4-NMe2-C_6_H_4_	40	68
**7n**	H	3-Br-C_6_H_4_	43	69
**7w**	6-Cl	3-OMe-C_6_H_4_	60	69
**7x**	6-Cl	4-F-C_6_H_4_	62	73
**7y**	6-Cl	4-NMe_2_-C_6_H_4_	54	71
**15b**	6-Cl	2-furyl	48	70
**15c**	6-Cl	2-thienyl	49	70

In addition, there was an error in **Table 2** **(Effects of amino acids 3 on enzyme inhibition)** as published. The figures A-C insert in **Table 2** were found to be incorrect. The corrected **Table 2** and its caption appear below.

**Table 2:** Effects of amino acids 3 on enzyme inhibition.

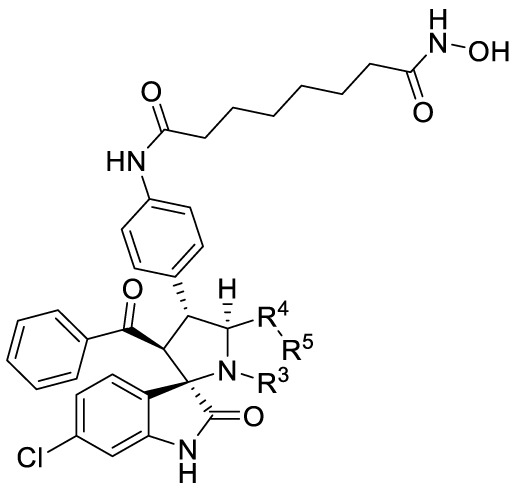					
compound	R^3^	R^4^	R^5^	% enzyme inhibition	
				MDM2	HDAC
**7f**	C	C	S	65	64
**7t**	C	C	C	61	61
**13a**	C	H	/	52	65
**15a**	H	Ph	/	67	61

The authors apologize for this error and state that this does not change the scientific conclusions of the article in any way. The original article has been updated.

