# Supplementary material for: Discovery of spirooxindole-derived small-molecule compounds as novel HDAC/MDM2 dual inhibitors and investigation of their anticancer activity
Source: Front Oncol. 2022 Aug 4;12:972372. doi: 10.3389/fonc.2022.972372 (PMC9386376; doi:10.3389/fonc.2022.972372)

Supplementary Information for

Discovery of spirooxindole-derived small-molecule compounds as novel HDAC/MDM2 dual inhibitors and investigation of their anticancer activity

Qian Zhao^1,†^, Shan-Shan Xiong^2,†^, Can Chen^3,4,†^, Hong-Ping Zhu^1,5^, Xin Xie^1^, Cheng Peng^1^*, Gu He^2^* and Bo Han^1^*

^1^State Key Laboratory of Southwestern Chinese Medicine Resources, Hospital of Chengdu University of Traditional Chinese Medicine, School of Basic Medical Sciences, Chengdu University of Traditional Chinese Medicine, Chengdu 611137, China

^2^Department of Dermatology and State Key Laboratory of Biotherapy, West China Hospital, Sichuan University, Chengdu, Sichuan 610041, China

^3^School of Pharmacy, Chengdu Medical College, Chengdu 610500, China

^4^The First Affiliated Hospital, Chengdu Medical College, Chengdu 610500, China

^5^Antibiotics Research and Re-evaluation Key Laboratory of Sichuan Province, Sichuan Industrial Institute of Antibiotics, Chengdu University, Chengdu 610106, China

^†^These authors contributed equally to this work

**Table of Contents**

1. General methods…………………………………………………………………….1

2. Spectroscopic data of compounds **4a**-**4v**……………………………………………1

3. Spectroscopic data of compounds **5a**-**5v**……………………………………………6

4. General Procedure for the Preparation of intermediates **16-20**…………...……...…10

5. Crystal data of compound **4o**……………………………………………………….11

6. NMR Spectra for Target Compounds **7a-7y**, **9a-9e**, **11a-11b**, **13a**, **15a-15c**…….….13

**1. General methods**

Nuclear magnetic resonance (NMR) spectra were recorded in CDCl_3_ or DMSO-*d_6_* on Bruker 400 MHz NMR instrument (at 400 MHz for ^1^H, and at 100 for ^13^C). Protonchemical shifts are reported in parts per million (δ scale). The ^1^H NMR chemical shifts are reported in ppm with the internal TMS signal at 0.0 ppm as standard. The ^13^C NMR chemical shifts were given using CDCl_3_ or DMSO-*d_6_* as the internal standard (CDCl_3_: *δ* = 77.00 ppm; DMSO-*d_6_*: *δ* = 39.51 ppm). Data are reported as follows: chemical shift [multiplicity (s = singlet, d = doublet, t = triplet, q = quartet, m = multiplet, br = broad), coupling constant(s) (Hz), integration]. High-resolution mass spectra (HRMS) were obtained using Agilent P/N G1969-90010. High-resolution mass spectra were reported for the molecular ion [M+Na]^+^. X-ray diffraction experiment was carried out on Agilent Gemini or Agilent D8 QUEST and the data obtained were deposited at the Cambridge Crystallographic Data Centre. UV detection was performed at 254 nm. Column UV detection was performed at 254 nm. Column chromatography was performed on silica gel (200–300 mesh) using an eluent of ethyl acetate, petroleum ether, methanol, and dichloromethane. TLC was performed on glass-backed silica plates; products were visualized using UV light. All reagents and solvents were obtained from commercial sources and used without further purification. Oil baths were used as the heat source. Melting points were recorded on BUCHI Melting Point M-565 instrument.

**2. Spectroscopic data of compounds 4a-4v**

***(3R,6'S,7'R,7a'S)-6'-benzoyl-7'-(4-nitrophenyl)-1',6',7',7a'-tetrahydro-3'H-spiro[indoline-3,5'-pyrrolo[1,2-c]thiazol]-2-one (4a).*** ^1^H NMR (400 MHz, CDCl_3_): δ = 8.24–8.21 (m, 2H), 7.77–7.74 (m, 2H), 7.55 (d, *J =* 7.6 Hz, 1H), 7.43 (s, 1H), 7.40–7.35 (m, 1H), 7.33–7.30 (m, 2H), 7.20-7.16 (m, 2H), 7.13 (dd, *J =* 8.0 Hz, 1.2 Hz, 1H), 7.04 (td, *J =* 7.6 Hz, 1.2 Hz, 1H), 6.48 (d, *J =* 8.0 Hz, 1H), 4.77 (d, *J =* 11.6 Hz, 1H), 4.44–4.40 (m, 1H), 4.08 (dd, *J =* 11.6 Hz, 10.0 Hz, 1H), 3.90 (d, *J =* 10.4 Hz, 1H), 3.54 (d, *J =* 10.4 Hz, 1H), 3.10 (dd, *J =* 12.0 Hz, 6.4 Hz, 1H), 2.99 (dd, *J =* 11.6, 2.0 Hz, 1H) ppm.

***(3R,6'S,7'R,7a'S)-6'-benzoyl-5-methyl-7'-(4-nitrophenyl)-1',6',7',7a'-tetrahydro-3'H-spiro[indoline-3,5'-pyrrolo[1,2-c]thiazol]-2-one (4b).*** ^1^H NMR (400 MHz, CDCl_3_): δ = 8.24–8.21 (m, 2H), 7.81–7.75 (m, 2H), 7.39–7.35 (m, 3H), 7.33–7.31 (m, 2H), 7.20–7.16 (m, 2H), 6.93 (d, *J =* 7.6 Hz, 1H), 6.37 (d, *J =* 8.0 Hz, 1H), 4.76 (d, *J =* 11.6 Hz, 1H), 4.43–4.38 m, 1H), 4.07 (dd, *J =* 11.6 Hz, 9.6 Hz, 1H), 3.89 (d, *J =* 10.4 Hz, 1H), 3.54 (d, *J =* 10.4 Hz, 1H), 3.09 (dd, *J =* 11.6 Hz, 6.2 Hz, 1H), 2.99 (dd, *J =* 11.6 Hz, 2.0 Hz, 1H), 2.35 (s, 3H) ppm.

***(3R,6'S,7'R,7a'S)-6'-benzoyl-5-fluoro-7'-(4-nitrophenyl)-1',6',7',7a'-tetrahydro-3'H-spiro[indoline-3,5'-pyrrolo[1,2-c]thiazol]-2-one (4c).*** ^1^H NMR (400 MHz, CDCl_3_): δ = 8.24–8.21 (m, 2H), 7.99 (s, 1H), 7.77–7.74 (m, 2H), 7.59 (d, *J =* 2.0 Hz, 1H), 7.41–7.35 (m, 3H), 7.22–7.18 (m, 2H), 6.89 (td, *J =* 8.8 Hz, 2.8 Hz, 1H), 6.49 (dd, *J =* 8.4 Hz, 4.0 Hz, 1H), 4.78 (d, *J =* 11.6 Hz, 1H), 4.43–4.38 (m, 1H), 4.04 (dd, *J =* 11.6 Hz, 9.6 Hz, 1H), 3.90 (d, *J =* 10.4 Hz, 1H), 3.52 (d, *J =* 10.4 Hz, 1H), 3.10 (dd, *J =* 12.0 Hz, 6.4 Hz, 1H), 3.00 (dd, *J =* 12.0 Hz, 2.4 Hz, 1H) ppm.

***(3R,6'S,7'R,7a'S)-6'-benzoyl-5-chloro-7'-(4-nitrophenyl)-1',6',7',7a'-tetrahydro-3'H-spiro[indoline-3,5'-pyrrolo[1,2-c]thiazol]-2-one (4d).*** ^1^H NMR (400 MHz, CDCl_3_): δ = 8.27–8.19 (m, 2H), 7.94 (s, 1H), 7.79 - 7.73 (m, 2H), 7.59 (d, *J =* 2.0 Hz, 1H), 7.42–7.34 (m, 3H), 7.23–7.17 (m, 2H), 7.15 (dd, *J =* 8.4 Hz, 2.0 Hz, 1H), 6.48 (d, *J =* 8.4 Hz, 1H), 4.77 (d, *J =* 11.6 Hz, 1H), 4.43–4.38 (m, 1H), 4.05 (dd, *J =* 11.6 Hz, 9.7 Hz, 1H), 3.89 (d, *J =* 10.4 Hz, 1H), 3.49 (d, *J =* 10.4 Hz, 1H), 3.09 (dd, *J =* 11.6 Hz, 6.4 Hz, 1H), 3.00 (dd, *J =* 12.0 Hz, 2.4 Hz, 1H) ppm.

***(3R,6'S,7'R,7a'S)-6'-benzoyl-5-bromo-7'-(4-nitrophenyl)-1',6',7',7a'-tetrahydro-3'H-spiro[indoline-3,5'-pyrrolo[1,2-c]thiazol]-2-one (4e).*** ^1^H NMR (400 MHz, CDCl_3_): δ = 8.24–8.21 (m, 2H), 7.77–7.74 (m, 2H), 7.71 (d, *J =* 2.0 Hz, 1H), 7.64 (s, 1H), 7.41–7.35 (m, 3H), 7.29 (dd, *J =* 8.0 Hz, 1.6 Hz, 1H), 7.23–7.19 (m, 2H), 6.41 (d, *J =* 8.4 Hz, 1H), 4.77 (d, *J =* 11.2 Hz, 1H), 4.43–4.38 (m, 1H), 4.04 (dd, *J =* 11.6 Hz, 9.6 Hz, 1H), 3.88 (d, *J =* 10.4 Hz, 1H), 3.49 (d, *J =* 10.4 Hz, 1H), 3.09 (dd, *J =* 12.0 Hz, 6.4 Hz, 1H), 3.00 (dd, *J =* 11.6 Hz, 2.4 Hz, 1H) ppm.

***(3R,6'S,7'R,7a'S)-6'-benzoyl-6-chloro-7'-(4-nitrophenyl)-1',6',7',7a'-tetrahydro-3'H-spiro[indoline-3,5'-pyrrolo[1,2-c]thiazol]-2-one (4f).*** ^1^H NMR (400 MHz, CDCl_3_): δ = 8.22 (d, *J =* 8.4 Hz, 2H), 7.73 (d, *J =* 8.4 Hz, 2H), 7.54 (s, 1H), 7.51 (d, *J =* 8.4 Hz, 1H), 7.43–7.39 (m, 1H), 7.37–7.35 (m, 2H), 7.25–7.21 (m, 2H), 7.03 (d, *J =* 8.0 Hz, 1H), 6.54 (d, *J =* 2.0 Hz, 1H), 4.77 (d, *J =* 11.6 Hz, 1H), 4.42–4.38 (m, 1H), 4.04 (dd, *J =* 11.6 Hz, 9.6 Hz, 1H), 3.88 (d, *J =* 10.4 Hz, 1H), 3.49 (d, *J =* 10.4 Hz, 1H), 3.09 (dd, *J =* 12.0 Hz, 6.4 Hz, 1H), 2.99 (dd, *J =* 12.0 Hz, 2.0 Hz, 1H) ppm.

***(3R,6'S,7'R,7a'S)-6'-benzoyl-6-bromo-7'-(4-nitrophenyl)-1',6',7',7a'-tetrahydro-3'H-spiro[indoline-3,5'-pyrrolo[1,2-c]thiazol]-2-one (4g).*** ^1^H NMR (400 MHz, CDCl_3_): δ = 8.24–8.20 (m, 2H), 7.91 (s, 1H), 7.75–7.72 (m, 2H), 7.45 (d, *J =* 8.0 Hz, 1H), 7.41 (t, *J =* 7.6 Hz, 1H), 7.38–7.34 (m, 2H), 7.25–7.18 (m, 3H), 6.73 (d, *J =* 1.6 Hz, 1H), 4.76 (d, *J =* 11.6 Hz, 1H), 4.42–4.38 (m, 1H), 4.04 (dd, *J =* 11.6 Hz, 9.6 Hz, 1H), 3.88 (d, *J =* 10.8 Hz, 1H), 3.49 (d, *J =* 10.4 Hz, 1H), 3.10 (dd, *J =* 12.0 Hz, 6.4 Hz, 1H), 2.99 (dd, *J =* 12.0 Hz, 2.0 Hz, 1H) ppm.

***(3R,6'S,7'R,7a'S)-6'-benzoyl-7-methyl-7'-(4-nitrophenyl)-1',6',7',7a'-tetrahydro-3'H-spiro[indoline-3,5'-pyrrolo[1,2-c]thiazol]-2-one (4h).*** ^1^H NMR (400 MHz, CDCl_3_): δ = 8.24–8.21 (m, 2H), 7.77–7.74 (m, 3H), 7.39–7.33 (m, 2H), 7.26–7.23 (m, 2H), 7.18–7.14 (m, 2H), 6.97–6.95 (m, 2H), 4.74 (d, *J =* 11.6 Hz, 1H), 4.43–4.38 (m, 1H), 4.06 (dd, *J =* 11.6 Hz, 9.6 Hz, 1H), 3.90 (d, *J =* 10.4 Hz, 1H), 3.58 (d, *J =* 10.4 Hz, 1H), 3.10 (dd, *J =* 11.6 Hz, 6.4 Hz, 1H), 2.99 (dd, *J =* 12.0 Hz, 2.4 Hz, 1H), 1.92 (s, 3H) ppm.

***(3R,6'S,7'R,7a'S)-6'-benzoyl-7-bromo-7'-(4-nitrophenyl)-1',6',7',7a'-tetrahydro-3'H-spiro[indoline-3,5'-pyrrolo[1,2-c]thiazol]-2-one (4i).*** ^1^H NMR (400 MHz, CDCl_3_): δ = 8.24–8.21 (m, 2H), 7.76–7.73 (m, 2H), 7.50 (d, *J =* 7.6 Hz, 1H), 7.44–7.40 (m, 1H), 7.29–7.26 (m, 2H), 7.24–7.21 (m, 3H), 6.95 (t, *J =* 8.0 Hz, 1H), 4.75 (d, *J =* 11.6 Hz, 1H), 4.43–4.39 (m, 1H), 4.02 (dd, *J =* 11.6, Hz, 9.6 Hz, 1H), 3.91 (d, *J =* 10.8 Hz, 1H), 3.53 (d, *J =* 10.8 Hz, 1H), 3.09 (dd, *J =* 12.0 Hz, 6.4 Hz, 1H), 2.98 (dd, *J =* 12.0 Hz, 2.4 Hz, 1H) ppm.

***(3R,6'S,7'R,7a'S)-6'-benzoyl-5-methoxy-7'-(4-nitrophenyl)-1',6',7',7a'-tetrahydro-3'H-spiro[indoline-3,5'-pyrrolo[1,2-c]thiazol]-2-one (4j).*** ^1^H NMR (400 MHz, CDCl_3_): δ = 8.25–8.18 (m, 2H), 8.12 (s, 1H), 7.80–7.72 (m, 2H), 7.40–7.33 (m, 3H), 7.22–7.14 (m, 3H), 6.70 (dd, *J =* 8.4 Hz, 2.4 Hz, 1H), 6.45 (d, *J =* 8.4 Hz, 1H), 4.77 (d, *J =* 11.6 Hz, 1H), 4.44–4.39 (m, 1H), 4.07 (dd, *J =* 11.6 Hz, 9.6 Hz, 1H), 3.90 (d, *J =* 10.4 Hz, 1H), 3.84 (s, 3H), 3.57 (d, *J =* 10.4 Hz, 1H), 3.10 (dd, *J =* 11.6 Hz, 6.4 Hz, 1H), 3.00 (dd, *J =* 11.6 Hz, 2.0 Hz, 1H) ppm.

***(3R,6'S,7'R,7a'S)-6'-benzoyl-6-methoxy-7'-(4-nitrophenyl)-1',6',7',7a'-tetrahydro-3'H-spiro[indoline-3,5'-pyrrolo[1,2-c]thiazol]-2-one (4k).*** ^1^H NMR (400 MHz, CDCl_3_): δ = 8.25–8.22 (m, 2 H), 8.15 (s, 1H), 7.79–7.77 (m, 2H), 7.39–7.36 (m, 3H), 7.21–7.18 (m, 3H), 6.72 (dd, *J =* 8.4 Hz, 2.4 Hz, 1H), 6.48 (d, *J =* 8.4 Hz, 1H), 4.79 (d, *J =* 11.6 Hz, 1H), 4.46–4.42 (m, 1H), 4.09 (dd, *J =* 11.6 Hz, 10.0 Hz, 1H), 3.92 (d, *J =* 10.4 Hz, 1H), 3.86 (s, 3H), 3.59 (d, *J =* 10.8 Hz, 1H), 3.14–3.10 (m, 1H), 3.03 (dd, *J =* 12.0 Hz, 2.0 Hz, 1H) ppm.

***(3R,6'S,7'R,7a'S)-6'-(3-methoxybenzoyl)-7'-(4-nitrophenyl)-1',6',7',7a'-tetrahydro-3'H-spiro[indoline-3,5'-pyrrolo[1,2-c]thiazol]-2-one (4l).*** ^1^H NMR (400 MHz, CDCl_3_): δ = 8.35 (s, 1H), 8.23–8.21 (m, 2H), 7.76–7.74 (m, 2H), 7.57 (d, *J =* 7.6 Hz, 1H), 7.19–7.15 (m, 1H), 7.10–7.04 (m, 2H), 7.00 (d, *J =* 7.6 Hz, 1H), 6.89 (dd, *J =* 8.0 Hz, 2.4 Hz, 1H), 6.73 (t, *J =* 2.0 Hz, 1H), 6.59 (d, *J =* 8.0 Hz, 1H), 4.75 (d, *J =* 11.6 Hz, 1H), 4.43–4.39 (m, 1H), 4.08 (dd, *J =* 11.6 Hz, 9.6 Hz, 1H), 3.89 (d, *J =* 10.8 Hz, 1H), 3.61 (s, 3H), 3.54 (d, *J =* 10.8 Hz, 1H), 3.10 (dd, *J =* 11.6 Hz, 6.4 Hz, 1H), 3.00 (dd, *J =* 12.0 Hz, 2.0 Hz, 1H) ppm.

***(3R,6'S,7'R,7a'S)-6'-(4-(dimethylamino)benzoyl)-7'-(4-nitrophenyl)-1',6',7',7a'-tetrahydro-3'H-spiro[indoline-3,5'-pyrrolo[1,2-c]thiazol]-2-one (4m).*** ^1^H NMR (400 MHz, CDCl_3_): δ = 8.19–8.16 (m, 2H), 7.72–7.68 (m, 2H), 7.67–7.65 (m, 2H), 7.40–7.36 (m, 2H), 7.15 (td, *J =* 8.0 Hz, 1.6 Hz, 1H), 7.04 (td, *J =* 7.6 Hz, 0.8 Hz, 1H), 6.60 (d, *J =* 7.2 Hz, 1H), 6.36–6.33 (m, 2H), 4.73 (d, *J =* 11.6 Hz, 1H), 4.43–4.38 (m, 1H), 4.14 (dd, *J =* 11.6 Hz, 9.6 Hz, 1H), 3.91 (d, *J =* 10.4 Hz, 1H), 3.53 (d, *J =* 10.4 Hz, 1H), 3.10 (dd, *J =* 11.6 Hz, 6.4 Hz, 1H), 2.99 (dd, *J =* 12.0 Hz, 2.4 Hz, 1H), 2.91 (s, 6H) ppm.

***(3R,6'S,7'R,7a'S)-6'-(3-bromobenzoyl)-7'-(4-nitrophenyl)-1',6',7',7a'-tetrahydro-3'H-spiro[indoline-3,5'-pyrrolo[1,2-c]thiazol]-2-one (4n).*** ^1^H NMR (400 MHz, CDCl_3_): δ = 8.25–8.22 (m, 2H), 7.82 (s, 1H), 7.76–7.73 (m, 2H), 7.53 (d, *J =* 7.6 Hz, 1H), 7.50–7.47 (m, 1H), 7.35 (t, *J =* 2.0 Hz, 1H), 7.29–7.28 (m, 1H), 7.19 (td, *J =* 7.6 Hz, 1.2 Hz, 1H), 7.10–7.04 (m, 2H), 6.56 (d, *J =* 7.6 Hz, 1H), 4.69 (d, *J =* 11.6 Hz, 1H), 4.43–4.37 (m, 1H), 4.05 (dd, *J =* 11.6 Hz, 9.6 Hz, 1H), 3.90 (d, *J =* 10.8 Hz, 1H), 3.54 (d, *J =* 10.8 Hz, 1H), 3.09 (dd, *J =* 11.6 Hz, 6.4 Hz, 1H), 2.98 (dd, *J =* 12.0 Hz, 2.0 Hz, 1H) ppm.

***(1'R,2'S,3R,7a'R)-2'-benzoyl-6-chloro-1'-(4-nitrophenyl)-1',2',5',6',7',7a'-hexahydrospiro[indoline-3,3'-pyrrolizin]-2-one (4o).*** ^1^H NMR (400 MHz, CDCl_3_): δ = 8.52 (s, 1H), 8.20–8.17 (m, 2H), 7.69–7.67 (m, 2H), 7.43–7.40 (m, 2H), 7.38–7.36 (m, 1H), 7.23–7.19 (m, 2H), 7.15 (d, *J =* 8.0 Hz, 1H), 7.01 (dd, *J =* 8.0 Hz, 2.0 Hz, 1H), 6.65 (d, *J =* 2.0 Hz, 1H), 4.91 (d, *J =* 11.2 Hz, 1H), 4.29–4.24 (m, 1H), 4.03–3.97 (m, 1H), 2.74–2.67 (m, 1H), 2.65–2.59 (m, 1H), 2.09–2.01 (m, 1H), 1.98–1.89 (m, 2H), 1.77–1.68 (m, 1H) ppm.

***(3R,6'S,7'R,7a'S)-6-chloro-6'-(3-methoxybenzoyl)-7'-(4-nitrophenyl)-1',6',7',7a'-tetrahydro-3'H-spiro[indoline-3,5'-pyrrolo[1,2-c]thiazol]-2-one (4p).*** ^1^H NMR (400 MHz, CDCl_3_): δ = 8.24–8.25 (m, 2H), 7.87 (s, 1H), 7.74–7.72 (m, 2H), 7.50 (d, *J =* 8.0 Hz, 1H), 7.14 (t, *J =* 8.0 Hz, 1H), 7.06–7.01 (m, 2H), 6.94 (dd, *J =* 8.4 Hz, 2.8 Hz, 1H), 6.74–6.73 (m, 1H), 6.58 (d, *J =* 2.0 Hz, 1H), 4.73 (d, *J =* 11.6 Hz, 1H), 4.42–4.37 (m, 1H), 4.02 (dd, *J =* 11.6 Hz, 9.6 Hz, 1H), 3.88 (d, *J =* 10.8 Hz, 1H), 3.67 (s, 3H), 3.50 (d, *J =* 10.8 Hz, 1H), 3.10 (dd, *J =* 12.0 Hz, 6.4 Hz, 1H), 3.00–2.96 (m, 1H) ppm.

***(3R,6'S,7'R,7a'S)-6-chloro-6'-(4-fluorobenzoyl)-7'-(4-nitrophenyl)-1',6',7',7a'-tetrahydro-3'H-spiro[indoline-3,5'-pyrrolo[1,2-c]thiazol]-2-one (4q).*** ^1^H NMR (400 MHz, CDCl_3_): δ = 8.24–8.21 (m, 2H), 7.73–7.71 (m, 2H), 7.51 (d, *J =* 8.0 Hz, 1H), 7.45–7.41 (m, 2H), 7.28 (s, 1H), 7.04 (dd, *J =* 8.4 Hz, 2.0 Hz, 1H), 6.95–6.90 (t, *J =* 8.5 Hz, 2H), 6.57 (d, *J =* 1.6 Hz, 1H), 4.72 (d, *J =* 11.6 Hz, 1H), 4.42–4.37 (m, 1H), 4.03 (dd, *J =* 11.6 Hz, 9.6 Hz, 1H), 3.89 (d, *J =* 10.8 Hz, 1H), 3.48 (d, *J =* 10.8 Hz, 1H), 3.09 (dd, *J =* 12.0 Hz, 6.4 Hz, 1H), 2.98 (dd, *J =* 12.0 Hz, 2.0 Hz, 1H) ppm.

***(3R,6'S,7'R,7a'S)-6-chloro-6'-(4-(dimethylamino)benzoyl)-7'-(4-nitrophenyl)-1',6',7',7a'-tetrahydro-3'H-spiro[indoline-3,5'-pyrrolo[1,2-c]thiazol]-2-one (4r).*** ^1^H NMR (400 MHz, CDCl_3_): δ = 8.23–8.21 (m, 1H), 8.18–8.16 (m, 2H), 7.85–7.82 (m, 2H), 7.69–7.67 (m, 2H), 7.63–7.59 (m, 1H), 7.42–7.39 (m, 2H), 7.01 (d, *J =* 8.4 Hz, 1H), 6.65–6.62 (m, 2H), 4.72 (d, *J =* 11.6 Hz, 1H), 4.41–4.37 (m, 1H), 4.09 (dd, *J =* 11.6 Hz, 9.6 Hz, 1H), 3.89 (d, *J =* 10.4 Hz, 1H), 3.50–3.48 (m, 1H), 3.22–3.10 (m, 1H), 2.92 (s, 6H) ppm.

***(3R,3'S,4'R)-3'-benzoyl-6-chloro-1'-methyl-4'-(4-nitrophenyl)spiro[indoline-3,2'-pyrrolidin]-2-one (4s).*** ^1^H NMR (400 MHz, CDCl_3_): δ = 8.20–8.16 (m, 2H), 7.81 (s, 1H), 7.72–7.68 (m, 2H), 7.42–7.35 (m, 3H), 7.22–7.18 (m, 2H), 7.03 (d, *J =* 8.0 Hz, 1H), 6.92 (dd, *J =* 8.0 Hz, 2.0 Hz, 1H), 6.50 (d, *J =* 2.0 Hz, 1H), 4.61 (td, *J =* 9.2 Hz, 6.8 Hz, 1H), 4.48 (d, *J =* 9.6 Hz, 1H), 3.66 (t, *J =* 9.2 Hz, 1H), 3.49 (dd, *J =* 8.8 Hz, 7.2 Hz, 1H), 2.22 (s, 3H) ppm.

***(3R,3'S,4'R,5'S)-3'-benzoyl-6-chloro-4'-(4-nitrophenyl)-5'-phenylspiro[indoline-3,2'-pyrrolidin]-2-one (4t).*** ^1^H NMR (400 MHz, CDCl_3_): δ = 8.12–8.10 (m, 2H), 7.72–7.67 (m, 1H), 7.58–7.56 (m, 2H), 7.45–7.42 (m, 2H), 7.39 (d, *J =* 7.6 Hz, 1H), 7.36–7.32 (m, 2H), 7.30–7.26 (m, 3H), 7.25–7.21 (m, 4H), 6.98 (dd, *J =* 8.0 Hz, 1.6 Hz, 1H), 6.49 (d, *J =* 1.6 Hz, 1H), 5.14 (d, *J =* 10.4 Hz, 1H), 4.73 (d, *J =* 10.8 Hz, 1H), 4.33 (t, *J =* 10.8 Hz, 1H) ppm.

***(3R,6'S,7'R,7a'S)-6-chloro-6'-(furan-2-carbonyl)-7'-(4-nitrophenyl)-1',6',7',7a'-tetrahydro-3'H-spiro[indoline-3,5'-pyrrolo[1,2-c]thiazol]-2-one (4u).*** ^1^H NMR (400 MHz, CDCl_3_): δ = 8.46 (s, 1H), 8.22–8.19 (m, 2H), 7.71–7.68 (m, 2H), 7.52 (d, *J =* 8.0 Hz, 1H), 7.40–7.39 (m, 1H), 7.02 (dt, *J =* 8.4 Hz, 1.6 Hz, 1H), 6.98 (d, *J =* 3.6 Hz, 1H), 6.77 (d, *J =* 1.6 Hz, 1H), 6.35–6.34 (m, 1H), 4.57 (dd, *J =* 12.0 Hz, 1.2 Hz, 1H), 4.41–4.37 (m, 1H), 4.05 (dd, *J =* 11.2 Hz, 9.2 Hz, 1H), 3.88 (d, *J =* 10.4 Hz, 1H), 3.52 (d, *J =* 9.6 Hz, 1H),3.08 (dd, *J =* 11.6 Hz, 6.4 Hz, 1H), 2.97 (dd, *J =* 11.6 Hz, 2.4 Hz, 1H) ppm.

***(3R,6'S,7'R,7a'S)-6-chloro-7'-(4-nitrophenyl)-6'-(thiophene-2-carbonyl)-1',6',7',7a'-tetrahydro-3'H-spiro[indoline-3,5'-pyrrolo[1,2-c]thiazol]-2-one (4v).*** ^1^H NMR (400 MHz, CDCl_3_): δ = 8.34 (s, 1H), 8.22–8.19 (m, 2H), 7.70–7.68 (m, 2H), 7.58 (d, *J =* 8.4 Hz, 1H), 7.54–7.52 (m, 1H), 7.50 (d, *J =* 5.2 Hz, 1H) 7.05 (dd, *J =* 8.0 Hz, 1.6 Hz, 1H), 6.94–6.92 (m, 1H), 6.71 (d, *J =* 1.6 Hz, 1H), 4.62 (d, *J =* 12.0 Hz, 1H), 4.42–4.38 (m, 1H), 4.05 (dd, *J =* 11.6 Hz, 9.6 Hz, 1H), 3.88 (d, *J =* 10.4 Hz, 1H), 3.51 (d, *J =* 10.8 Hz, 1H), 3.10 (dd, *J =* 12.0 Hz, 6.4 Hz, 1H), 2.98 (dd, *J =* 12.0, 2.0 Hz, 1H) ppm.

**3. Spectroscopic data of compounds 5a-5v**

***(3R,6'S,7'R,7a'S)-7'-(4-aminophenyl)-6'-benzoyl-1',6',7',7a'-tetrahydro-3'H-spiro[indoline-3,5'-pyrrolo[1,2-c]thiazol]-2-one (5a).*** ^1^H NMR (400 MHz, CDCl_3_): δ = 8.12 (s, 1H), 7.61 (d, *J =* 7.5 Hz, 1H), 7.36–7.30 (m, 5H), 7.16–7.09 (m, 3H), 7.02 (t, *J =* 7.6 Hz, 1H), 6.67–6.62 (m, 2H), 6.51 (d, *J =* 7.6 Hz, 1H), 4.72 (d, *J =* 11.7 Hz, 1H), 4.36–4.30 (m, 1H), 3.89 (d, *J =* 10.5 Hz, 1H), 3.84 (dd, *J =* 11.6, 9.6 Hz, 1H), 3.62 (s, 2H), 3.50 (d, *J =* 10.8 Hz, 1H), 3.10–3.01 (m, 2H) ppm.

***(3R,6'R,7'R,7a'S)-7'-(4-aminophenyl)-5-methyl-6'-(1-phenylvinyl)-1',6',7',7a'-tetrahydro-3'H-spiro[indoline-3,5'-pyrrolo[1,2-c]thiazol]-2-one (5b).*** ^1^H NMR (400 MHz, CDCl_3_): δ = 7.41 (d, *J =* 1.6 Hz, 1H), 7.39 (s, 1H), 7.36–7.33 (m, 5H), 7.18–7.14 (m, 2H), 6.92–6.90 (m, 1H), 6.67–6.65 (m, 2H), 6.35 (d, *J =* 8.0 Hz, 1H), 4.71 (d, *J =* 11.6 Hz, 1H), 4.35–4.30 (m, 1H), 3.89 (d, *J =* 10.4 Hz, 1H), 3.83 (dd, *J =* 11.6 Hz, 9.6 Hz, 1H), 3.63 (s, 2H), 3.50 (d, *J =* 10.4 Hz, 1H), 3.09–3.00 (m, 2H), 2.34 (s, 3H) ppm.

***(3R,6'S,7'R,7a'S)-7'-(4-aminophenyl)-6'-benzoyl-5-fluoro-1',6',7',7a'-tetrahydro-3'H-spiro[indoline-3,5'-pyrrolo[1,2-c]thiazol]-2-one (5c).*** ^1^H NMR (400 MHz, CDCl_3_): δ = 8.25 (s, 1H), 7.43 (dd, *J =* 8.4 Hz, 2.8 Hz, 1H), 7.40–7.38 (m, 2H), 7.37–7.32 (m, 1H), 7.31–7.28 (m, 2H), 7.18–7.14 (m, 2H), 6.85 (td, *J =* 8.8 Hz, 2.8 Hz, 1H), 6.66–6.63 (m, 2H), 6.46 (dd, *J =* 8.4 Hz, 4.4 Hz, 1H), 4.72 (d, *J =* 11.6 Hz, 1H), 4.34–4.29 (m, 1H), 3.89 (d, *J =* 10.8 Hz, 1H), 3.79 (dd, *J =* 11.6 Hz, 10.0 Hz Hz, 1H), 3.64 (s, 2H), 3.47 (d, *J =* 10.4 Hz, 1H), 3.09–3.01 (m, 2H) ppm.

***(3R,6'S,7'R,7a'S)-7'-(4-aminophenyl)-6'-benzoyl-5-chloro-1',6',7',7a'-tetrahydro-3'H-spiro[indoline-3,5'-pyrrolo[1,2-c]thiazol]-2-one (5d).*** ^1^H NMR (400 MHz, CDCl_3_): δ = 8.24–8.21 (m, 2H), 7.94 (s, 1H), 7.38 (d, *J =* 8.0 Hz, 3H), 7.32 (d, *J =* 7.5 Hz, 3H), 7.20 (t, *J =* 7.6 Hz, 2H), 7.14–7.08 (m, 1H), 6.66 (d, *J =* 8.0 Hz, 2H), 6.41 (d, *J =* 8.2 Hz, 1H), 4.72 (d, *J =* 11.7 Hz, 1H), 4.35–4.29 (m, 1H), 3.89 (d, *J =* 10.6 Hz, 1H), 3.80 (t, *J =* 10.8 Hz, 1H), 3.45 (d, *J =* 10.6 Hz, 1H), 3.09–3.02 (m, 2H) ppm.

***(3R,6'S,7'R,7a'S)-7'-(4-aminophenyl)-6'-benzoyl-5-bromo-1',6',7',7a'-tetrahydro-3'H-spiro[indoline-3,5'-pyrrolo[1,2-c]thiazol]-2-one (5e).*** ^1^H NMR (400 MHz, CDCl_3_): δ = 7.76 (d, *J =* 2.0 Hz, 1H), 7.41–7.31 (m, 5H), 7.25–7.18 (m, 3H), 6.72–6.62 (m, 2H), 6.35 (d, *J =* 8.0 Hz, 1H), 4.72 (d, *J =* 11.7 Hz, 1H), 4.35–4.30 (m, 1H), 3.88 (d, *J =* 10.6 Hz, 1H), 3.81–6.70 (m, 2H), 3.62 (s, 2H), 3.45 (d, *J =* 10.6 Hz, 1H), 3.09–3.01 m, 2H) ppm.

***(3R,6'S,7'R,7a'S)-7'-(4-aminophenyl)-6'-benzoyl-6-chloro-1',6',7',7a'-tetrahydro-3'H-spiro[indoline-3,5'-pyrrolo[1,2-c]thiazol]-2-one (5f).*** ^1^H NMR (400 MHz, CDCl_3_): δ = 8.21 (s, 1H), 7.56 (d, *J =* 8.0 Hz, 1H), 7.39–7.34 (m, 3H), 7.31–7.28 (m, 2H), 7.20–7.16 (m, 2H), 7.01 (dd, *J =* 8.0 Hz, 2.0 Hz, 1H), 6.66–6.63 (m, 2H), 6.56 (d, *J =* 2.0 Hz, 1H), 4.71 (d, *J =* 11.6 Hz, 1H), 4.34–4.29 (m, 1H), 3.87 (d, *J =* 10.8 Hz, 1H), 3.79 (dd, *J =* 12.0 Hz, 10.0 Hz, 1H), 3.63 (s, 2H), 3.44 (d, *J =* 10.8 Hz, 1H), 3.09–3.00 (m, 2H) ppm.

***(3R,6'S,7'R,7a'S)-7'-(4-aminophenyl)-6'-benzoyl-6-bromo-1',6',7',7a'-tetrahydro-3'H-spiro[indoline-3,5'-pyrrolo[1,2-c]thiazol]-2-one (5g).*** ^1^H NMR (400 MHz, CDCl_3_): δ = 8.28 (s, 1H), 7.51 (d, *J =* 8.4 Hz, 1H), 7.39–7.33 (m, 3H), 7.31–7.27 (m, 2H), 7.20–7.16 (m, 3H), 6.72 (d, *J =* 1.6 Hz, 1H), 6.66–6.63 (m, 2H), 4.71 (d, *J =* 11.6 Hz, 1H), 4.33–4.29 (m, 1H), 3.87 (d, *J =* 10.0 Hz, 1H), 3.79 (dd, *J =* 12.0 Hz, 10.0 Hz, 1H), 3.63 (s, 2H), 3.44 (d, *J =* 10.8 Hz, 1H), 3.08–3.00 (m, 2H) ppm.

***(3R,6'S,7'R,7a'S)-7'-(4-aminophenyl)-6'-benzoyl-7-methyl-1',6',7',7a'-tetrahydro-3'H-spiro[indoline-3,5'-pyrrolo[1,2-c]thiazol]-2-one (5h).*** ^1^H NMR (400 MHz, CDCl_3_): δ = 8.10 (s, 1H), 7.44 –7.40 (m, 1H), 7.33–7.28 (m, 4H), 7.25 (d, *J =* 1.6 Hz, 1H), 7.14–7.10 (m, 2H), 6.94–6.93 (m, 2H), 6.66–6.64 (m, 2H), 4.68 (d, *J =* 11.6 Hz, 1H), 4.35–4.30 (m, 1H), 3.88 (d, *J =* 10.4 Hz, 1H), 3.81 (dd, *J =* 11.6 Hz, 9.6 Hz, 1H), 3.63 (s, 2H), 3.54 (d, *J =* 10.4 Hz, 1H), 3.09–3.00 (m, 2H), 1.93 (s, 3H) ppm.

***(3R,6'S,7'R,7a'S)-7'-(4-aminophenyl)-6'-benzoyl-7-bromo-1',6',7',7a'-tetrahydro-3'H-spiro[indoline-3,5'-pyrrolo[1,2-c]thiazol]-2-one (5i).*** ^1^H NMR (400 MHz, CDCl_3_): δ = 7.55 (d, *J =* 7.6 Hz, 1H), 7.42–7.37 (m, 1H), 7.33–7.30 (m, 2H), 7.27 (d, *J =* 1.6 Hz, 1H), 7.25–7.24 (m, 2H), 7.23–7.19 (m, 2H), 7.14 (s, 1H), 6.93 (t, *J =* 8.0 Hz, 1H), 6.68–6.65 (m, 2H), 4.70 (d, *J =* 11.6 Hz, 1H), 4.36–4.31 (m, 1H), 3.91 (d, *J =* 10.8 Hz, 1H), 3.77 (dd, *J =* 12.0 Hz, 10.0 Hz, 1H), 3.63 (s, 2H), 3.49 (d, *J =* 10.4 Hz, 1H), 3.09–3.00 (m, 2H) ppm.

***(3R,6'S,7'R,7a'S)-7'-(4-aminophenyl)-6'-benzoyl-5-methoxy-1',6',7',7a'-tetrahydro-3'H-spiro[indoline-3,5'-pyrrolo[1,2-c]thiazol]-2-one (5j).*** ^1^H NMR (400 MHz, CDCl_3_): δ = 7.58 (s, 1H), 7.38–7.36 (m, 2H), 7.34–7.32 (m, 3H), 7.24 (d, *J =* 2.4 Hz, 1H), 7.19–7.15 (m, 2H), 6.68–6.64 (m, 3H), 6.39 (d, *J =* 8.4 Hz, 1H), 4.71 (d, *J =* 12.0 Hz, 1H), 4.36–4.31 (m, 1H), 3.90 (d, *J =* 10.4 Hz, 1H), 3.84 (s, 3H), 3.80 (d, *J =* 11.2 Hz, 1H), 3.52 (d, *J =* 10.8 Hz, 1H), 3.49 (s, 1H), 3.09–3.02 (t, *J =* 4.0 Hz, 2H) ppm.

***(3R,6'S,7'R,7a'S)-7'-(4-aminophenyl)-6'-benzoyl-6-methoxy-1',6',7',7a'-tetrahydro-3'H-spiro[indoline-3,5'-pyrrolo[1,2-c]thiazol]-2-one (5k).*** ^1^H NMR (400 MHz, CDCl_3_): δ = 7.91 (s, 1H), 7.51 (d, *J =* 8.4 Hz, 1H), 7.39–7.36 (m, 2H), 7.34–7.30 (m, 3H), 7.19–7.15 (m, 2H), 6.66–6.64 (m, 2H), 6.54 (dd, *J =* 8.4 Hz, 2.4 Hz, 1H), 6.09 (d, *J =* 2.4 Hz, 1H), 4.70 (d, *J =* 11.6 Hz, 1H), 4.32–4.28 (m, 1H), 3.88 (d, *J =* 10.6 Hz, 1H), 3.80 (dd, *J =* 11.6 Hz, 9.6 Hz, 1H), 3.74 (s, 3H), 3.62 (s, 2H), 3.54–3.48 (m, 1H), 3.11–3.00 (m, 2H) ppm.

***(3R,6'S,7'R,7a'S)-7'-(4-aminophenyl)-6'-(3-methoxybenzoyl)-1',6',7',7a'-tetrahydro-3'H-spiro[indoline-3,5'-pyrrolo[1,2-c]thiazol]-2-one (5l).*** ^1^H NMR (400 MHz, CDCl_3_): δ = 8.04 (s, 1H), 7.61 (d, *J =* 7.6 Hz, 1H), 7.33–7.30 (m, 2H), 7.13 (td, *J =* 7.6 Hz, 1.2 Hz, 1H), 7.07 (t, *J =* 7.6 Hz, 1H), 7.05–7.01 (m, 2H), 6.89–6.86 (m, 1H), 6.74 (dd, *J =* 2.8 Hz, 1.6 Hz, 1H), 6.67–6.64 (m, 2H), 6.53 (d, *J =* 7.6 Hz, 1H), 4.69 (d, *J =* 12.0 Hz, 1H), 4.35–4.30 (m, 1H), 3.89 (d, *J =* 10.4 Hz, 1H), 3.83 (dd, *J =* 11.6 Hz, 9.6 Hz, 1H), 3.63–3.61 (m, 5H), 3.49 (d, *J =* 10.4 Hz, 1H), 3.09–3.01 (m, 2H) ppm.

***(3R,6'S,7'R,7a'S)-7'-(4-aminophenyl)-6'-(4-(dimethylamino)benzoyl)-1',6',7',7a'-tetrahydro-3'H-spiro[indoline-3,5'-pyrrolo[1,2-c]thiazol]-2-one (5m).*** ^1^H NMR (400 MHz, CDCl_3_): δ = 8.37 (s, 1H), 7.70 (d, *J =* 7.6 Hz, 1H), 7.40–7.38 (m, 2H), 7.28–7.25 (m, 2H), 7.12 (td, *J =* 7.6 Hz, 1.2 Hz, 1H), 7.01 (td, *J =* 7.6 Hz, 1.2 Hz, 1H), 6.63–6.58 (m, 3H), 6.28–6.26 (m, 2H), 4.69 (d, *J =* 12.0 Hz, 1H), 4.34–4.29 (m, 1H), 3.92–3.86 (m, 2H), 3.58 (s, 2H), 3.48 (d, *J =* 10.4 Hz, 1H), 3.09–3.01 (m, 2H), 2.82 (s, 6H) ppm.

***(3R,6'S,7'R,7a'S)-7'-(4-aminophenyl)-6'-(3-bromobenzoyl)-1',6',7',7a'-tetrahydro-3'H-spiro[indoline-3,5'-pyrrolo[1,2-c]thiazol]-2-one (5n).*** ^1^H NMR (400 MHz, CDCl_3_): δ = 8.53 (s, 1H), 7.59 (d, *J =* 7.2 Hz, 1H), 7.46–7.44 (m, 1H), 7.42–7.41 (m, 1H), 7.33–7.29 (m, 2H), 7.29 (dt, *J =* 7.6 Hz, 1.2 Hz, 1H), 7.17 (td, *J =* 7.6 Hz, 1.2 Hz, 1H), 7.06–7.02 (m, 2H), 6.68–6.64 (m, 2H), 6.60 (d, *J =* 8.0 Hz, 1H), 4.64 (d, *J =* 11.6 Hz, 1H), 4.36–4.31 (m, 1H), 3.91 (d, *J =* 10.4 Hz, 1H), 3.81 (dd, *J =* 12.0 Hz, 10.0 Hz, 1H), 3.65 (s, 2H), 3.51 (d, *J =* 10.8 Hz, 1H), 3.10–3.01 (m, 2H) ppm.

***(1'R,2'S,3R,7a'R)-1'-(4-aminophenyl)-2'-benzoyl-6-chloro-1',2',5',6',7',7a'-hexahydrospiro[indoline-3,3'-pyrrolizin]-2-one (5o).*** ^1^H NMR (400 MHz, CDCl_3_): δ = 8.84 (s, 1H), 7.43–7.41 (m, 2H), 7.34–7.30 (m, 1H), 7.27 (s, 1H), 7.25–7.24 (m, 1H), 7.19–7.14 (m, 3H), 6.99 (dd, *J =* 8.4 Hz, 2.0 Hz, 1H), 6.64–6.60 (m, 3H), 4.85 (d, *J =* 11.6 Hz, 1H), 4.19–4.13 (m, 1H), 3.77 (dd, *J =* 11.6 Hz, 9.6 Hz, 1H), 3.60 (s, 2H), 2.66–2.55 (m, 2H), 2.05–1.97 (m, 1H), 1.91–1.82 (m, 2H), 1.74–1.66 (m, 1H) ppm.

***(3R,6'S,7'R,7a'S)-7'-(4-aminophenyl)-6-chloro-6'-(3-methoxybenzoyl)-1',6',7',7a'-tetrahydro-3'H-spiro[indoline-3,5'-pyrrolo[1,2-c]thiazol]-2-one (5p).*** ^1^H NMR (400 MHz, CDCl3): δ = 8.55 (s, 1H), 7.56 (d, *J =* 8.0 Hz, 1H), 7.30–7.28 (m, 3H), 7.10–7.01 (m, 3H), 6.90–6.87 (m, 1H), 6.76 (dd, *J =* 2.8 Hz, 1.2 Hz, 1H), 6.65–6.61 (m, 2H), 6.60 (t, *J =* 1.6 Hz, 1H), 4.67 (d, *J =* 11.6 Hz, 1H), 4.32–4.29 (m, 1H), 3.86 (d, *J =* 10.8 Hz, 1H), 3.78 (dd, *J =* 11.6 Hz, 9.6 Hz, 1H), 3.62 (s, 3H), 3.452–3.43 (m, 2H), 3.07–3.03 (m, 2H) ppm.

***(3R,6'S,7'R,7a'S)-7'-(4-aminophenyl)-6-chloro-6'-(4-fluorobenzoyl)-1',6',7',7a'-tetrahydro-3'H-spiro[indoline-3,5'-pyrrolo[1,2-c]thiazol]-2-one (5q).*** ^1^H NMR (400 MHz, CDCl_3_): δ = 8.39 (s, 1H), 7.57 (d, *J =* 8.4 Hz, 1H), 7.45–7.42 (m, 2H), 7.29–7.27 (m, 2H), 7.02 (dd, *J =* 8.0 Hz, 2.0 Hz, 1H), 6.89–6.84 (m, 2H), 6.66–6.61 (m, 3H), 4.66 (d, *J =* 12.0 Hz, 1H), 4.33–4.28 (m, 1H), 3.87 (d, *J =* 11.2 Hz, 1H), 3.78 (dd, *J =* 12.0 Hz, 10.0 Hz, 1H), 3.47 (s, 1H), 3.44 (d, *J =* 11.2 Hz, 1H), 3.07–3.03 (m, 2H) ppm.

***(3R,6'S,7'R,7a'S)-7'-(4-aminophenyl)-6-chloro-6'-(4-(dimethylamino)benzoyl)-1',6',7',7a'-tetrahydro-3'H-spiro[indoline-3,5'-pyrrolo[1,2-c]thiazol]-2-one (5r).*** ^1^H NMR (400 MHz, CDCl_3_): δ = 8.15 (s, 1H), 7.65 (d, *J =* 8.4 Hz, 1H), 7.43–7.40 (m, 2H), 7.27–7.25 (m, 2H), 6.99 (dt, *J =* 8.0 Hz, 1.6 Hz, 1H), 6.63–6.60 (m, 3H), 6.34–6.31 (m, 2H), 4.68 (d, *J =* 12.0 Hz, 1H), 4.32–4.28 (m, 1H), 3.88 (d, *J =* 10.8 Hz, 1H), 3.83 (dd, *J =* 12.0 Hz, 9.6 Hz, 1H), 3.44 (d, *J =* 10.8 Hz, 1H), 3.08–3.05 (m, 1H), 3.04–3.02 (m, 1H), 2.87 (s, 6H) ppm.

***(3R,3'S,4'R)-4'-(4-aminophenyl)-3'-benzoyl-6-chloro-1'-methylspiro[indoline-3,2'-pyrrolidin]-2-one (5s).*** ^1^H NMR (400 MHz, CDCl_3_): δ = 8.07 (s, 1H), 7.42–7.39 (m, 2H), 7.34–7.28 (m, 3H), 7.18–7.14 (m, 2H), 7.05 (d, *J =* 8.0 Hz, 1H), 6.90 (dd, *J =* 8.0 Hz, 2.0 Hz, 1H), 6.65–6.62 (m, 2H), 6.47 (d, *J =* 2.0 Hz, 1H), 4.48 (d, *J =* 9.6 Hz, 1H), 4.42–4.36 (m, 1H), 3.61–3.56 (m, 3H), 3.39 (dd, J =8.8 Hz, 6.8 Hz, 1H), 2.20 (s, 3H) ppm.

***(3R,3'S,4'R,5'S)-4'-(4-aminophenyl)-3'-benzoyl-6-chloro-5'-phenylspiro[indoline-3,2'-pyrrolidin]-2-one (5t).*** ^1^H NMR (400 MHz, CDCl_3_): δ = 8.42 (s, 1H), 7.42–7.40 (m, 2H), 7.35–7.29 (m, 4H), 7.25–7.24 (m, 2H), 7.22–7.20 (m, 2H), 7.17–7.13 (m, 4H), 6.95 (dd, *J =* 8.0 Hz, 2.0 Hz, 1H), 6.55–6.53 (m, 2H), 6.46 (d, *J =* 1.6 Hz, 1H), 5.00 (d, *J =* 10.8 Hz, 1H), 4.70 (d, *J =* 10.8 Hz, 1H), 4.09 (t, *J =* 10.8 Hz, 1H), 3.07 (s, 2H) ppm.

***(3R,6'S,7'R,7a'S)-7'-(4-aminophenyl)-6-chloro-6'-(furan-2-carbonyl)-1',6',7',7a'-tetrahydro-3'H-spiro[indoline-3,5'-pyrrolo[1,2-c]thiazol]-2-one (5u).*** ^1^H NMR (400 MHz, CDCl_3_): δ = 8.99 (s, 1H), 7.59 (d, *J =* 8.4 Hz, 1H), 7.33 (d, *J =* 1.6 Hz, 1H), 7.27–7.23 (m, 3H), 6.99 (dd, *J =* 8.0 Hz, 1.6 Hz, 1H), 6.96 (d, *J =* 3.6 Hz, 1H), 6.77 (d, *J =* 2.0 Hz, 1H), 6.64–6.62 (m, 2H), 6.25 (dd, *J =* 3.6 Hz, 1.6 Hz, 1H), 4.49 (d, *J =* 12.0 Hz, 1H), 4.31–4.26 (m, 1H), 3.86 (d, *J =* 10.4 Hz, 1H), 3.79 (dd, *J =* 12.0 Hz, 9.6 Hz, 1H), 3.48–3.45 (m, 2H), 3.07–2.98 (m, 2H) ppm.

***(3R,6'S,7'R,7a'S)-7'-(4-aminophenyl)-6-chloro-6'-(thiophene-2-carbonyl)-1',6',7',7a'-tetrahydro-3'H-spiro[indoline-3,5'-pyrrolo[1,2-c]thiazol]-2-one (5v).*** ^1^H NMR (400 MHz, CDCl_3_): δ = 8.60 (s, 1H), 7.63 (d, *J =* 8.0 Hz, 1H), 7.52 (d, *J =* 3.6 Hz, 1H), 7.42 (d, *J =* 5.2 Hz, 1H), 7.26–7.24 (m, 3H), 7.03 (dd, *J =* 8.0 Hz, 2.0 Hz, 1H), 6.85 (t, *J =* 4.4 Hz, 1H), 6.70 (d, *J =* 1.6 Hz, 1H), 6.64–6.62 (m, 2H), 4.56 (d, *J =* 12.0 Hz, 1H), 4.32–4.28 (m, 1H), 3.87 (d, *J =* 10.8 Hz, 1H), 3.79 (dd, *J =* 12.4 Hz, 10.0 Hz, 1H), 3.47–3.45 (m, 2H), 3.08–2.99 (m, 2H) ppm.

**4. General Procedure for the Preparation of intermediates 16-20**

Scheme 1 Synthesis of intermediates **16**

To a solution of **A2** (6.0 mmol) in DCM (25 mL) was added HATU (6.0 mmol), DIEA (10.0 mmol), and the mixture was stirred at room temperature. After 30 min, O-triphenylmethylhydroxylamine **A1** (5.0 mmol) was added, and the reaction mixture was stirred at room temperature until it was completed. Then the mixture was washed with 1M hydrochloric acid and concentrated under reduced pressure to obtain coarse intermediate **A3**. Then THF/MeOH/2 N NaOH = 2:1:1 was added to dissolve **A3**, the mixture was stirred at room temperature overnight. When the reaction was complete (based on TLC monitoring), using diluted hydrochloric acid to adjust the pH to 3-5, The precipitate obtained was collected and washed with cold water to generate the corresponding intermediates **16**.

Compounds **17**-**20** were synthesized according to **16**.

Scheme 2 Synthesis of intermediates **17-20**.

5. Single crystal X-ray diffraction analysis crystal data of 4o

To a 5 mL tube containing **4o** (20 mg) was added a 3:1 mixture of ethyl acetate and petroleum ether (4 mL). A clear solution was obtained through ultrasound treatment and was kept at room temperature and **4o** crystals were obtained after the solvent evaporated, which were characterized by single crystal X-ray diffraction. X-ray diffraction experiment was carried out on an Agilent Gemini and the data obtained were deposited at the Cambridge Crystallographic Data Centre. The crystal structure was solved by Olex2 with the SHELXT structure solution program using Intrinsic Phasing and refined with the SHELXL refinement package using Least Squares minimisation. CCDC 2095868 (**4o**) contains the supplementary crystallographic data for this paper. X-ray crystal structure of **4o** with the ellipsoid contour at 50% probability levels.

| Identification code | twin_twin1_hklf4 |
| --- | --- |
| Empirical formula | C_27_H_22_ClN_3_O_4_ |
| Formula weight | 487.92 |
| Temperature/K | 293.15 |
| Crystal system | monoclinic |
| Space group | P2_1_/c |
| a/Å | 8.9610(19) |
| b/Å | 34.403(7) |
| c/Å | 8.252(2) |
| α/° | 90 |
| β/° | 107.48(3) |
| γ/° | 90 |
| Volume/Å^3^ | 2426.4(10) |
| Z | 4 |
| ρ_calc_g/cm^3^ | 1.336 |
| μ/mm^‑1^ | 0.196 |
| F(000) | 1016.0 |
| Crystal size/mm^3^ | 0.35 × 0.3 × 0.25 |
| Radiaiition | MoKα (λ = 0.71073) |
| 2Θ range for data collection/° | 5.692 to 52.746 |
| Index ranges | -11 ≤ h ≤ 11, -42 ≤ k ≤ 41, -10 ≤ l ≤ 10 |
| Reflections collected | 14348 |
| Independent reflections | 4824 [R_int_ = 0.0960, R_sigma_ = 0.0945] |
| Data/restraints/parameters | 4824/0/320 |
| Goodness-of-fit on F^2^ | 0.915 |
| Final R indexes [I>=2σ (I)] | R_1_ = 0.0597, wR_2_ = 0.1149 |
| Final R indexes [all data] | R_1_ = 0.1104, wR_2_ = 0.1263 |
| Largest diff. peak and hole / e Å^-3^ | 0.22/-0.20 |

6. NMR Spectra for Target Compounds 7a-7y, 9a-9e, 11a-11b, 13a, 15a-15c


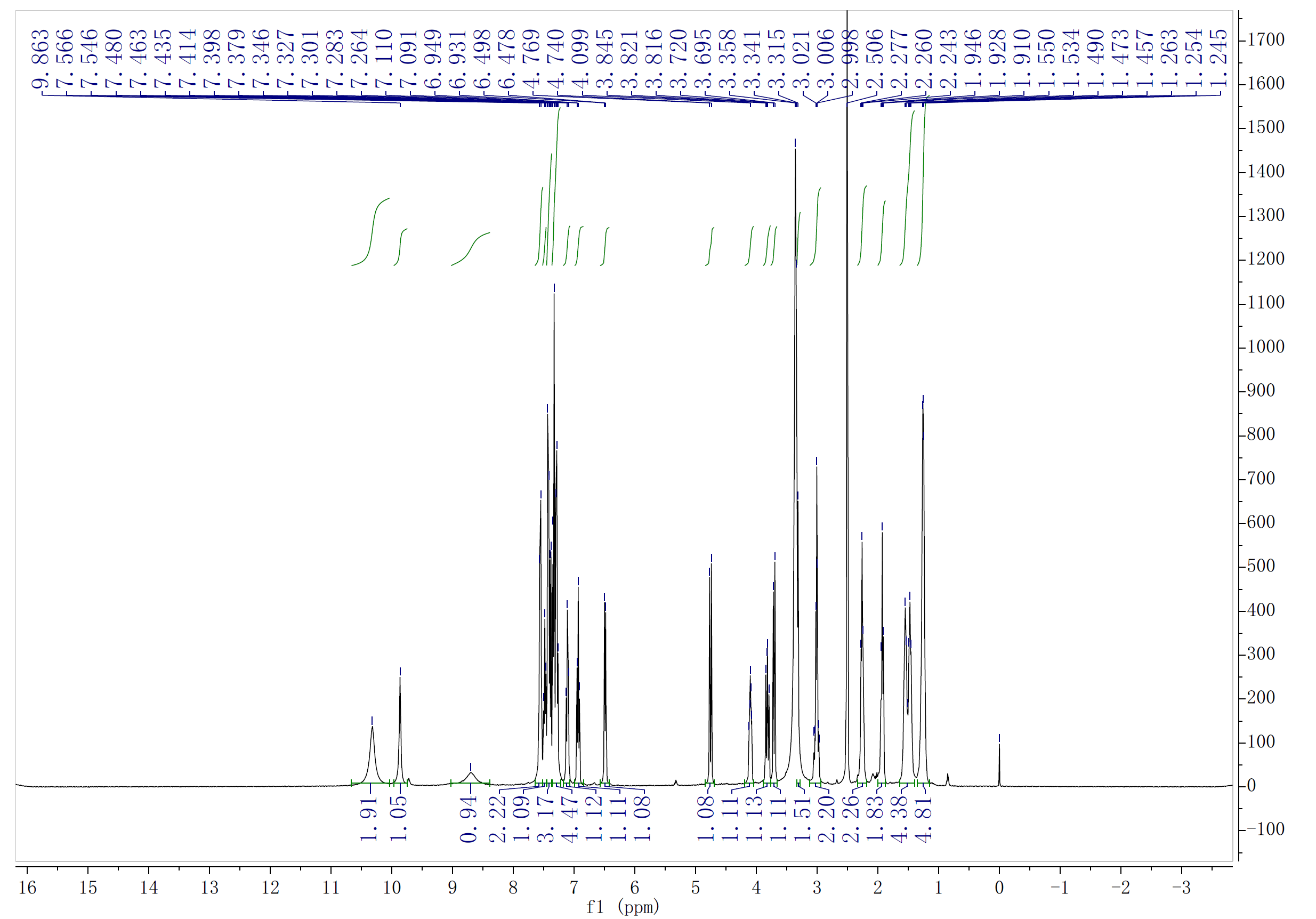


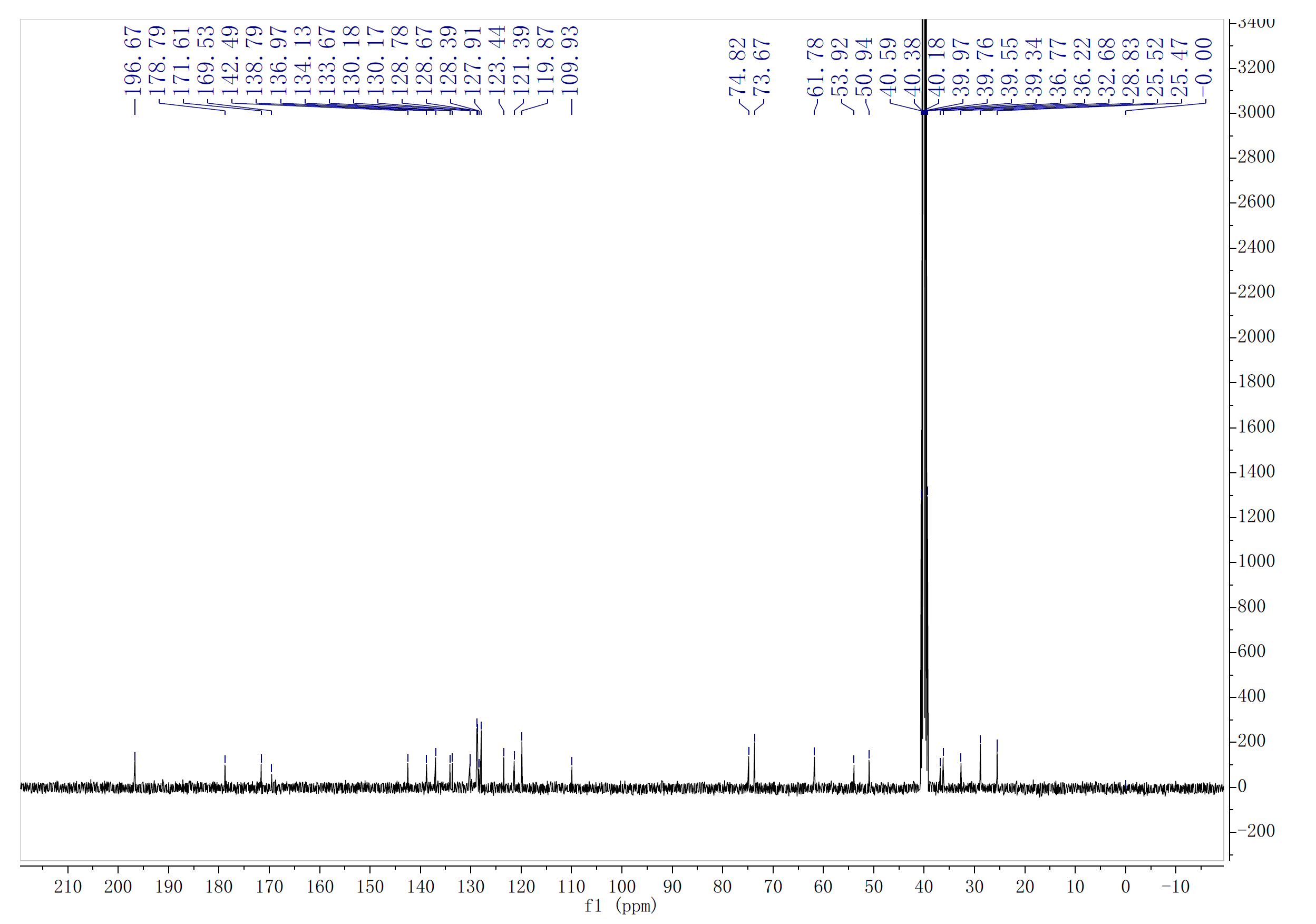


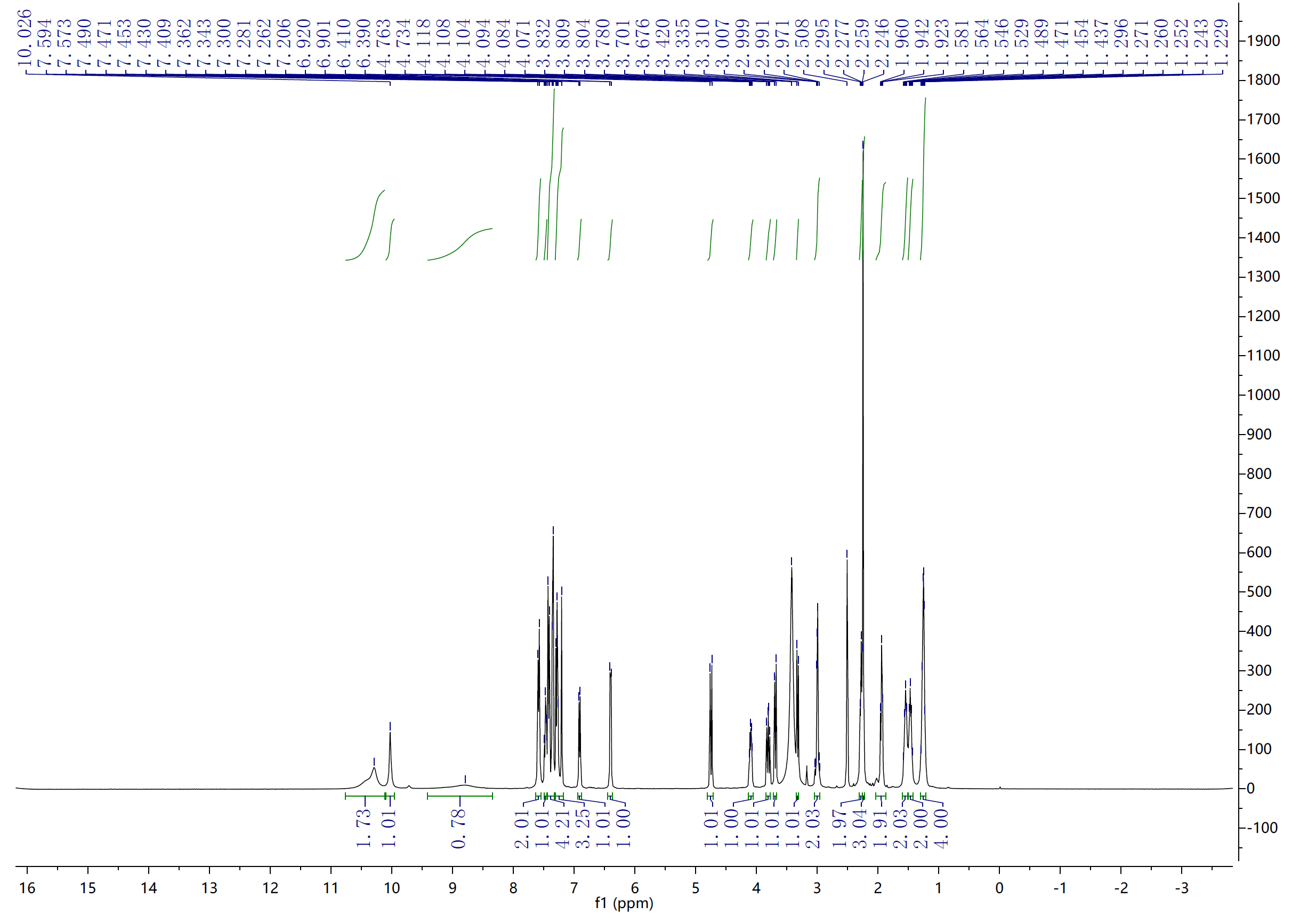


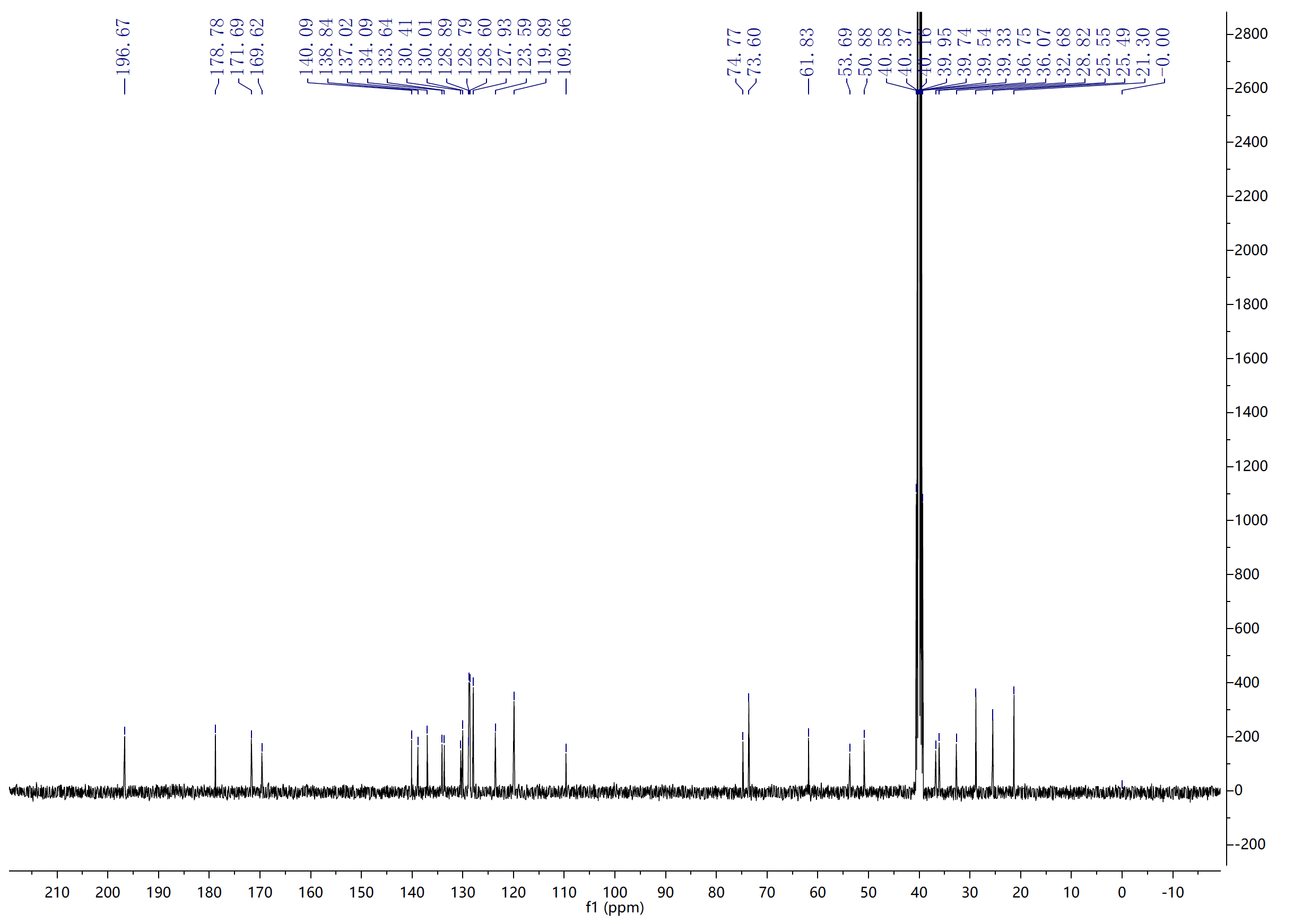


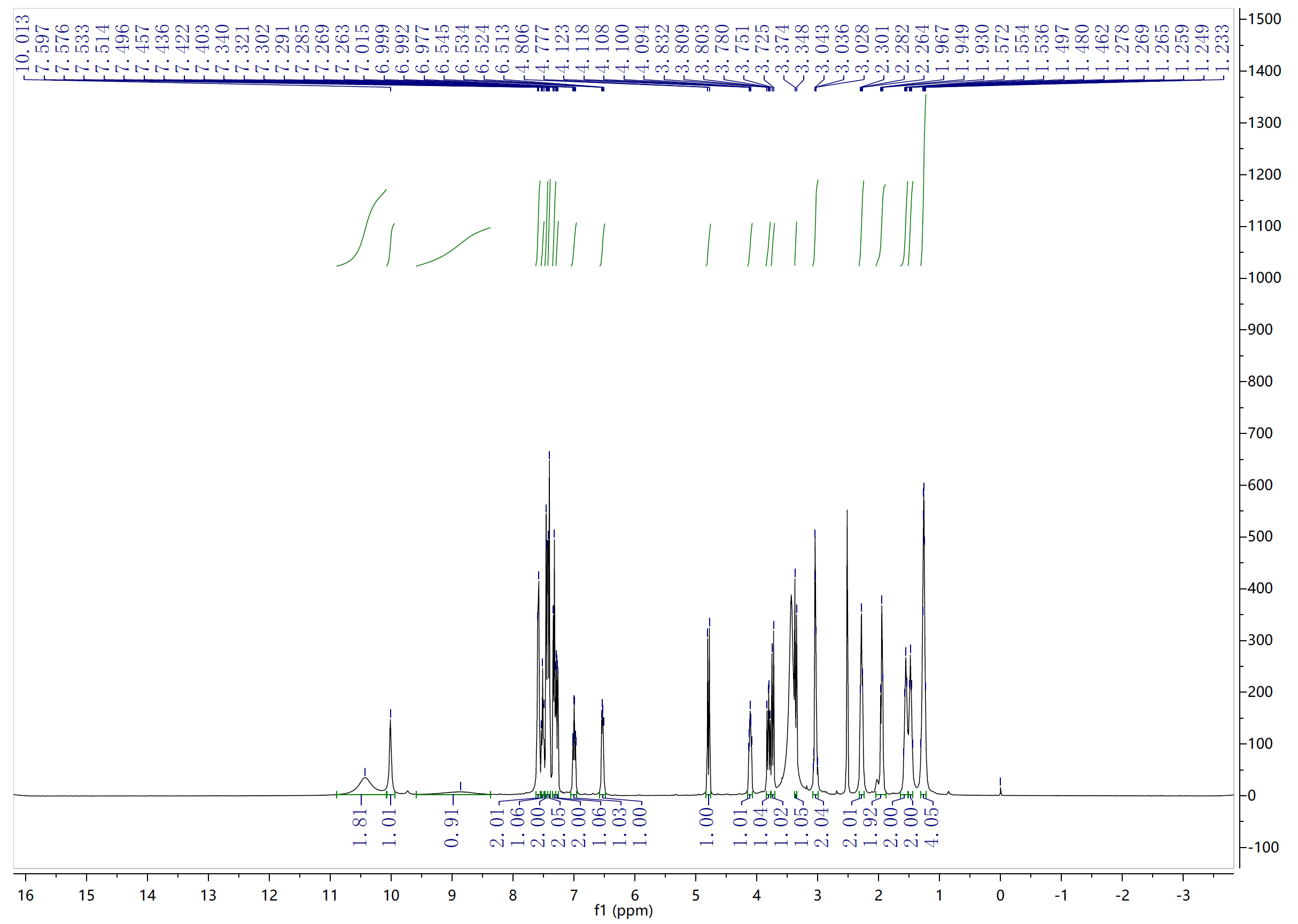


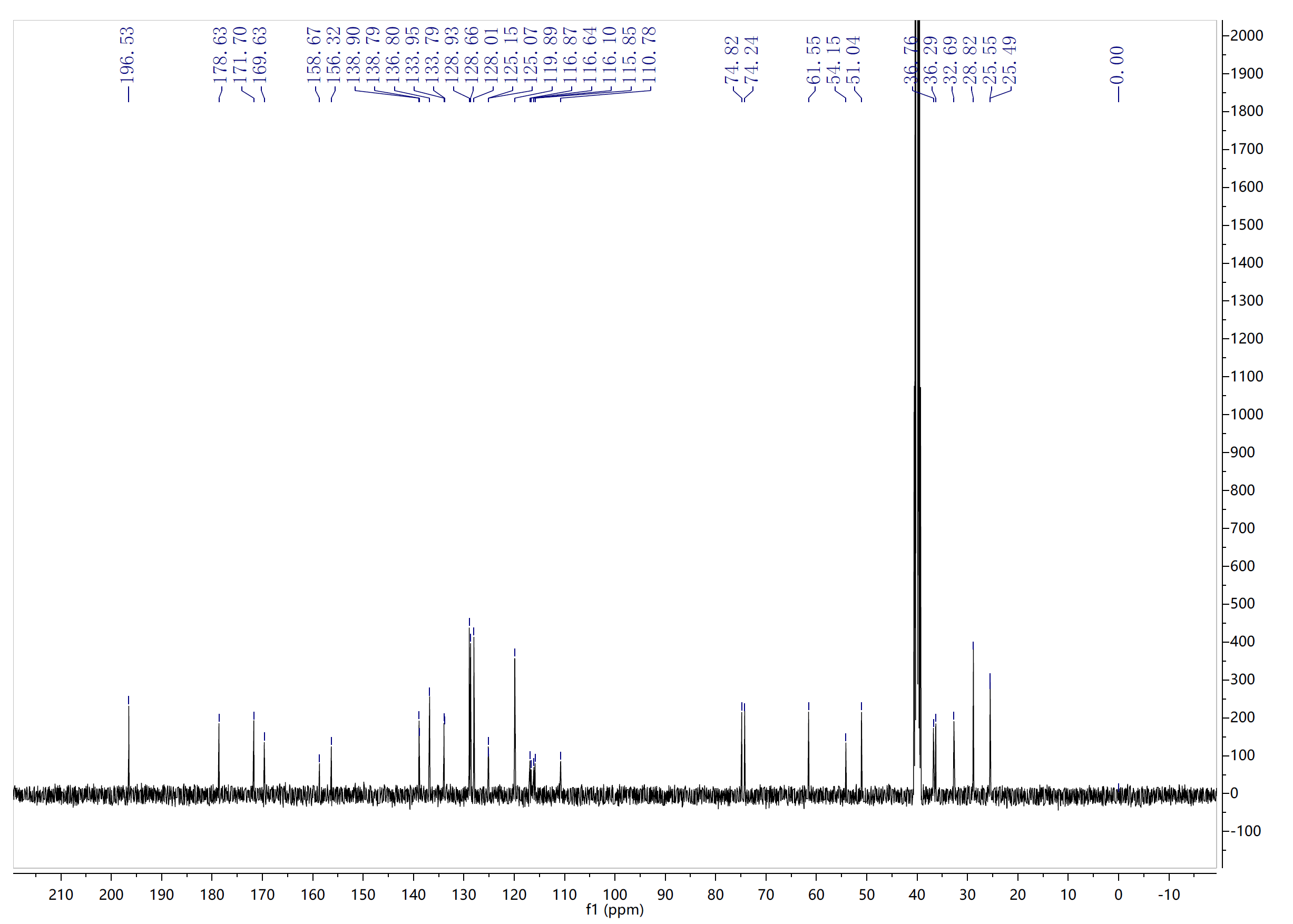


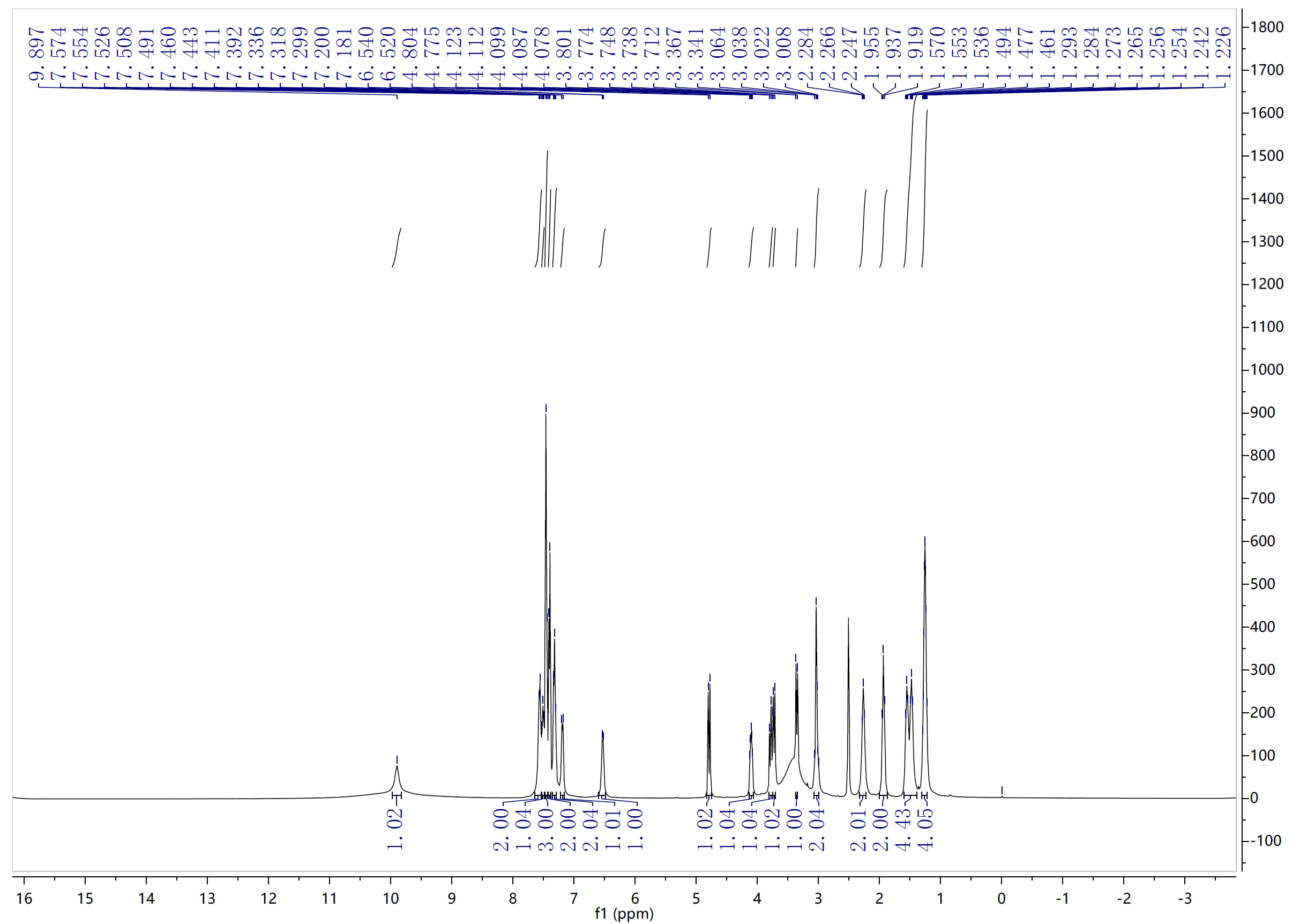


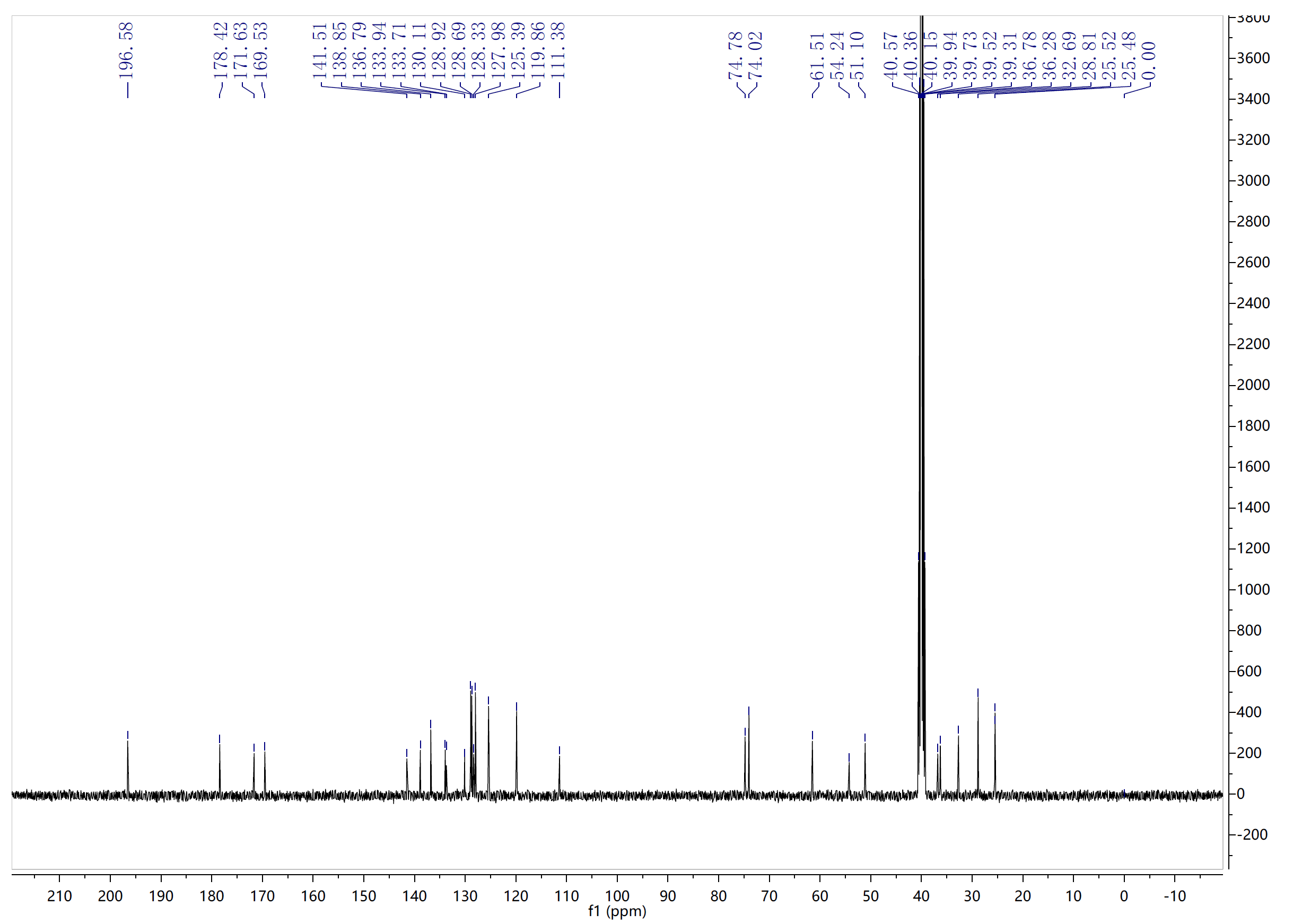


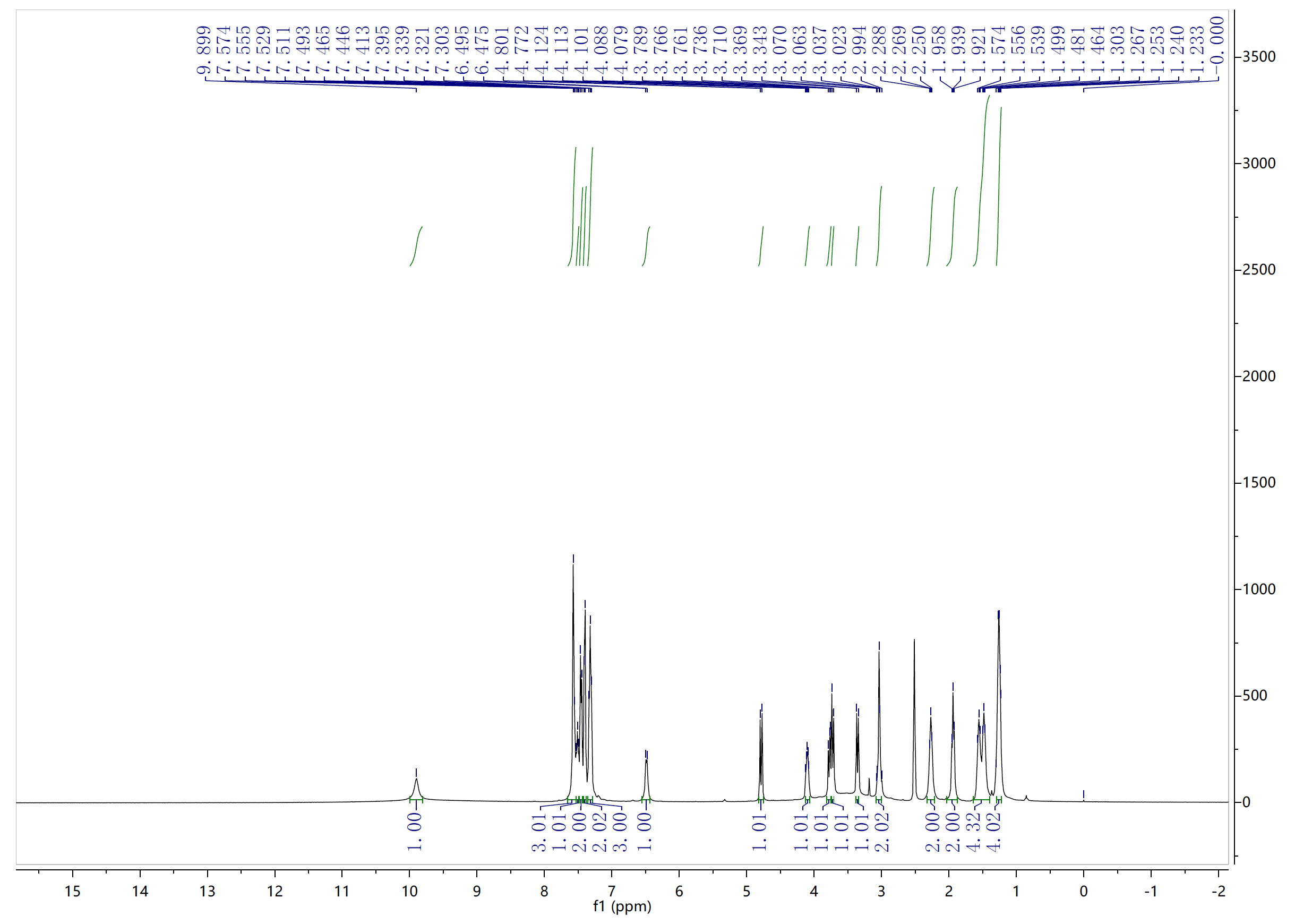


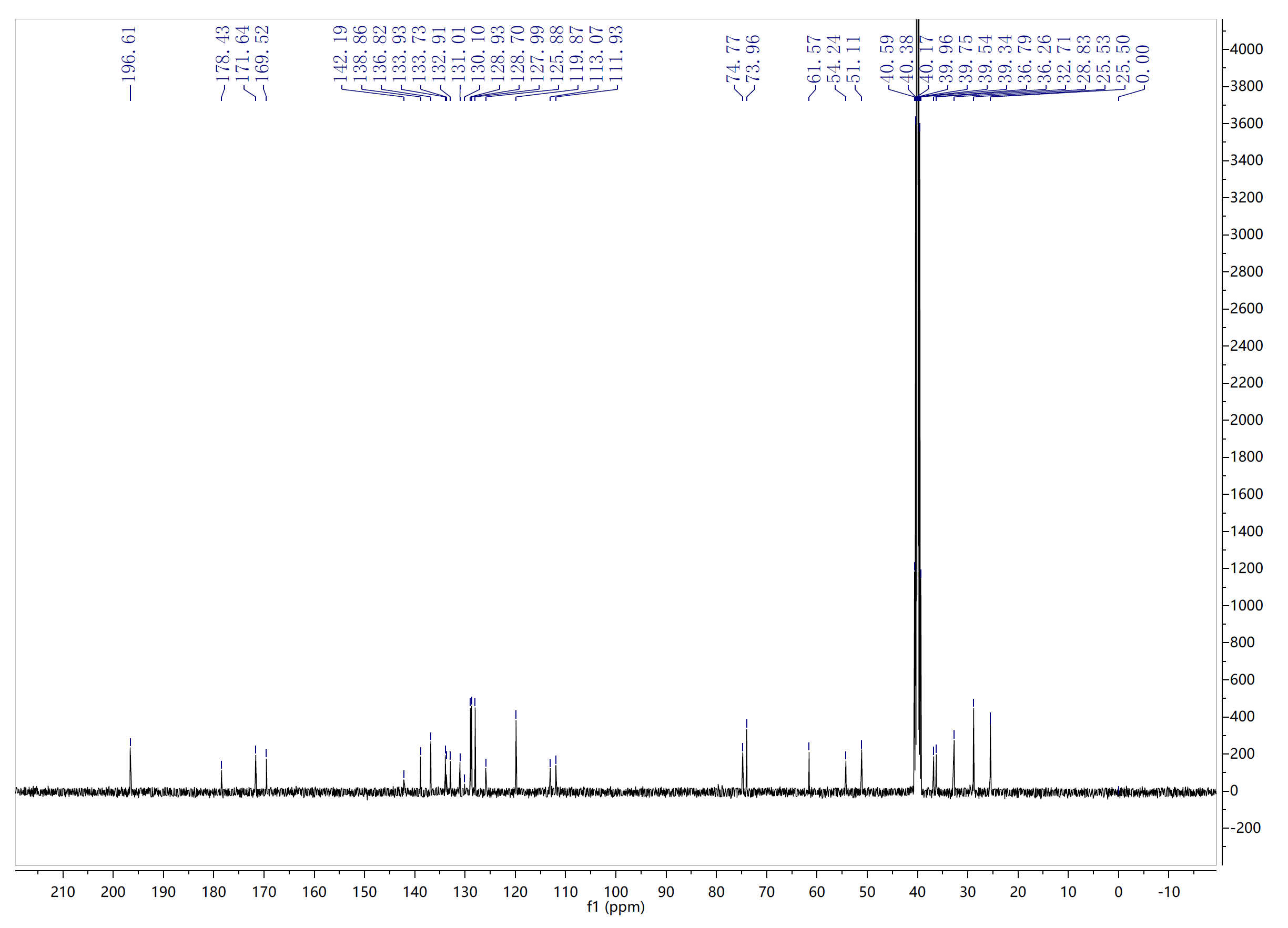


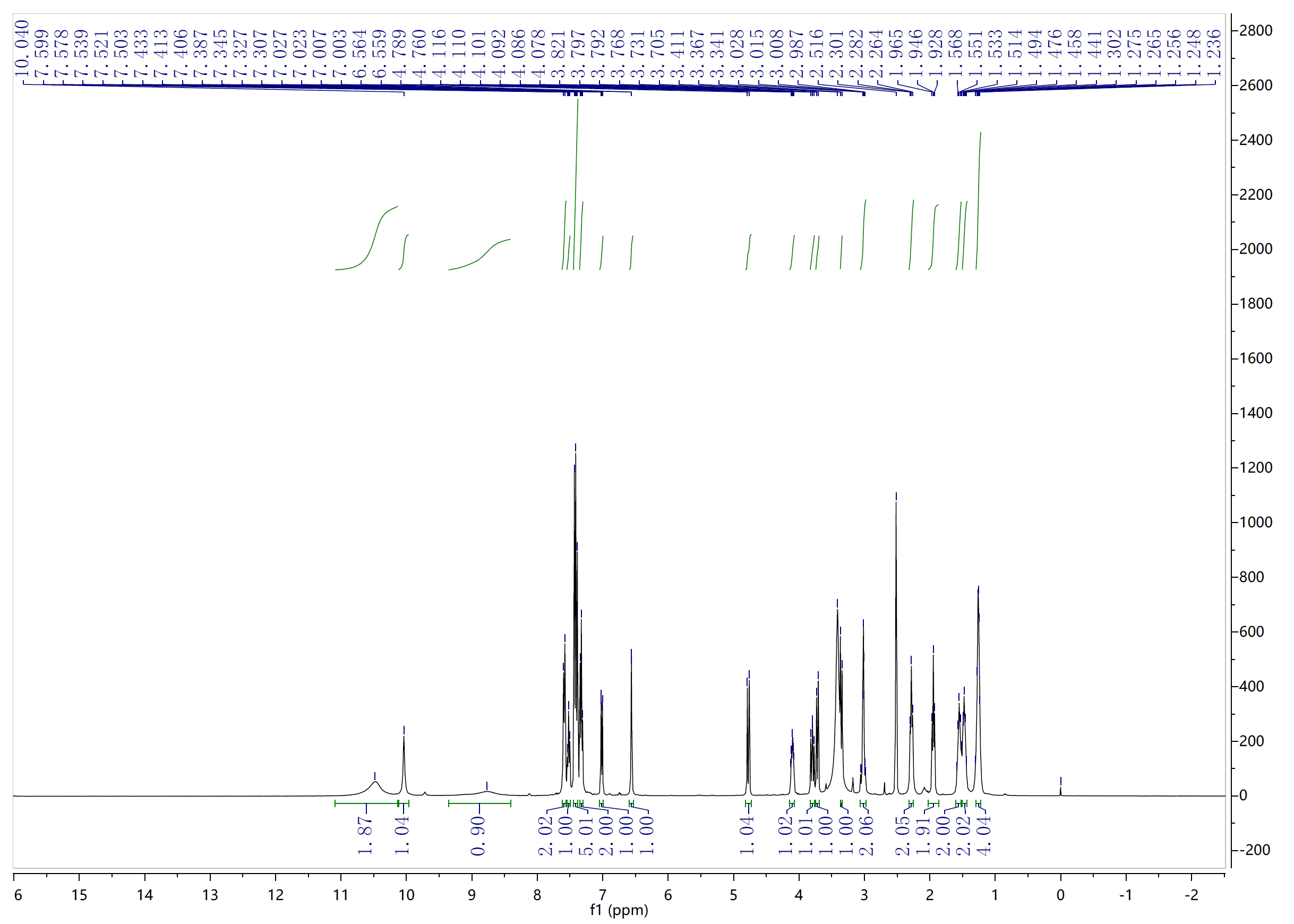


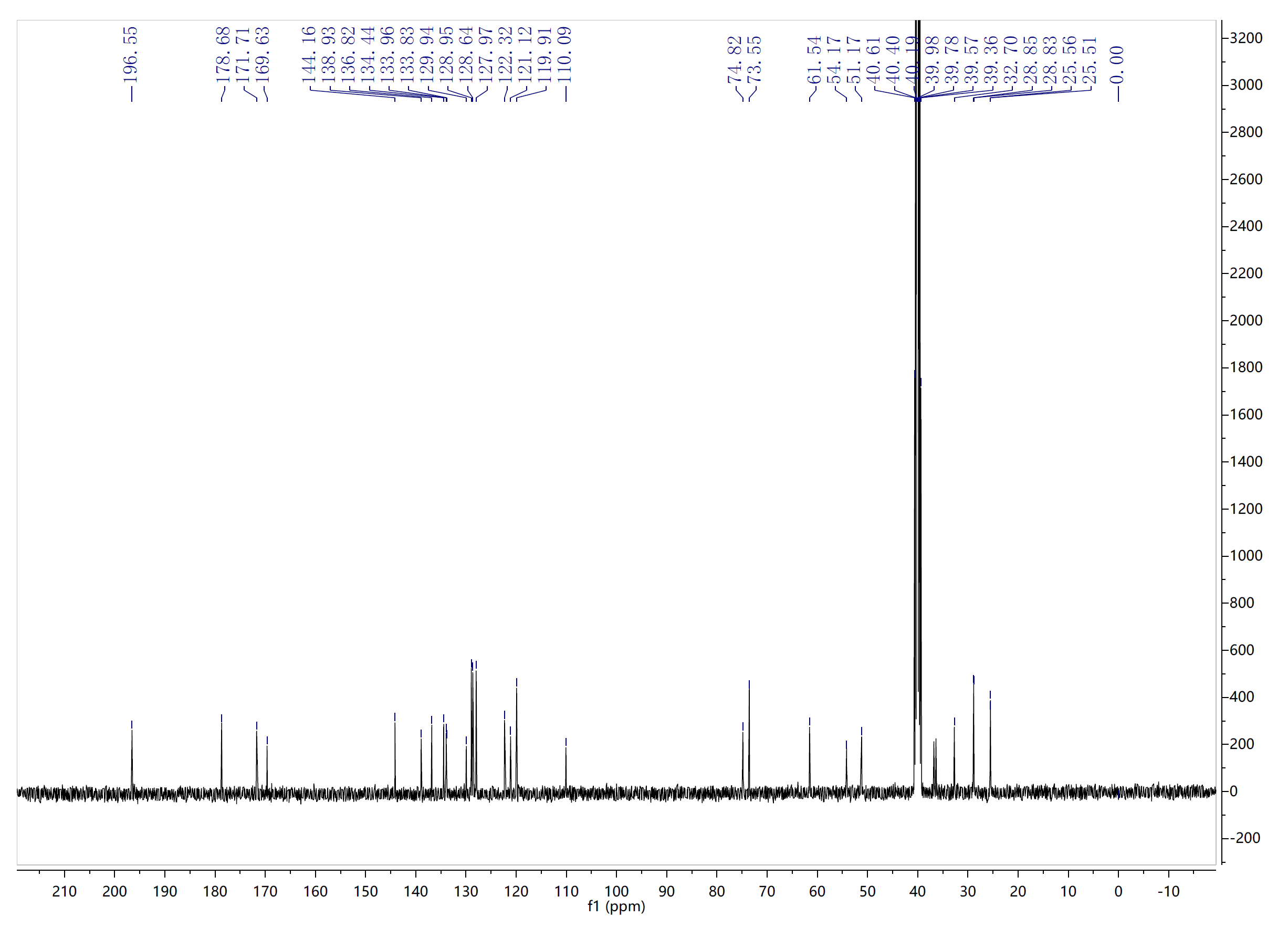


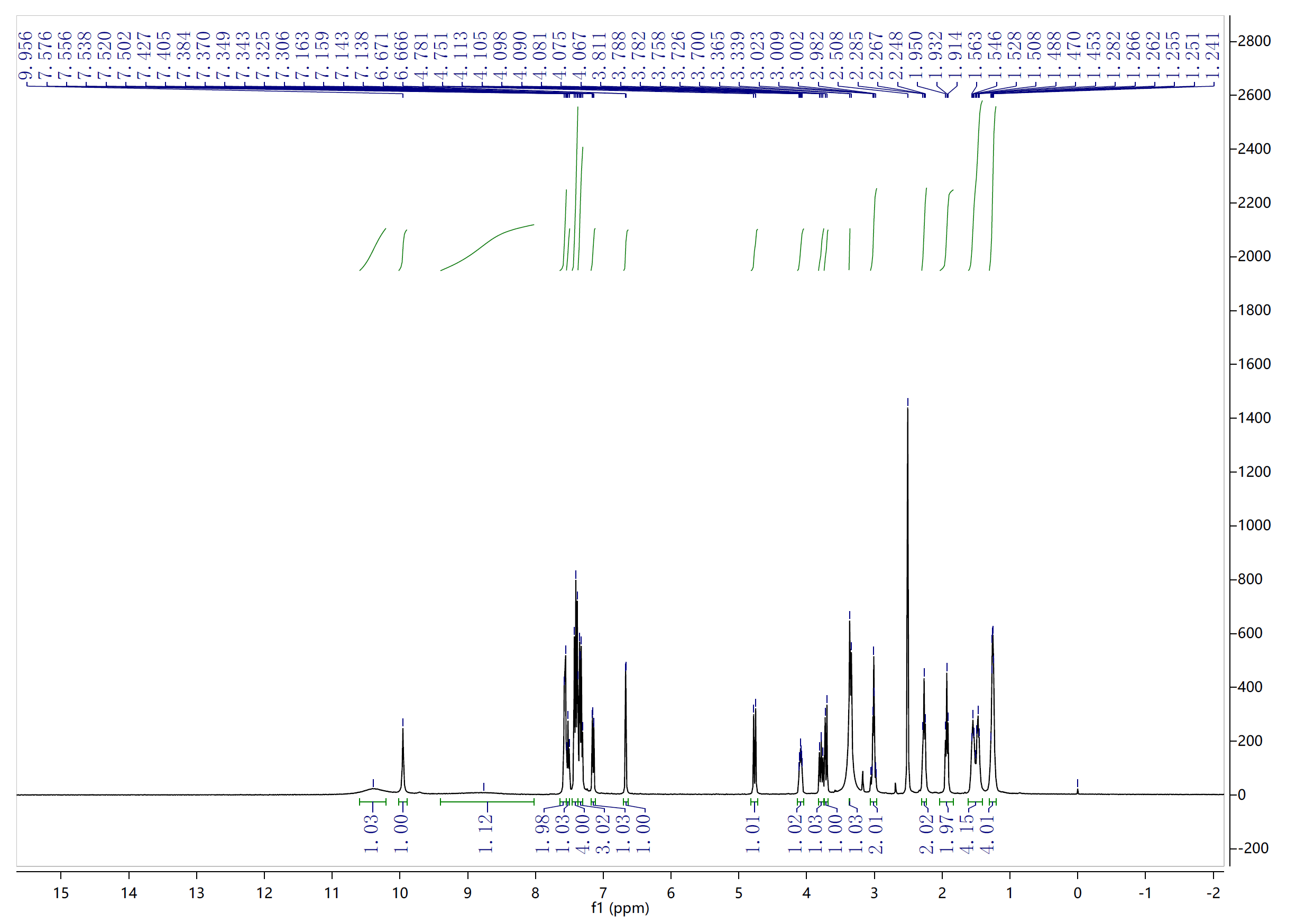


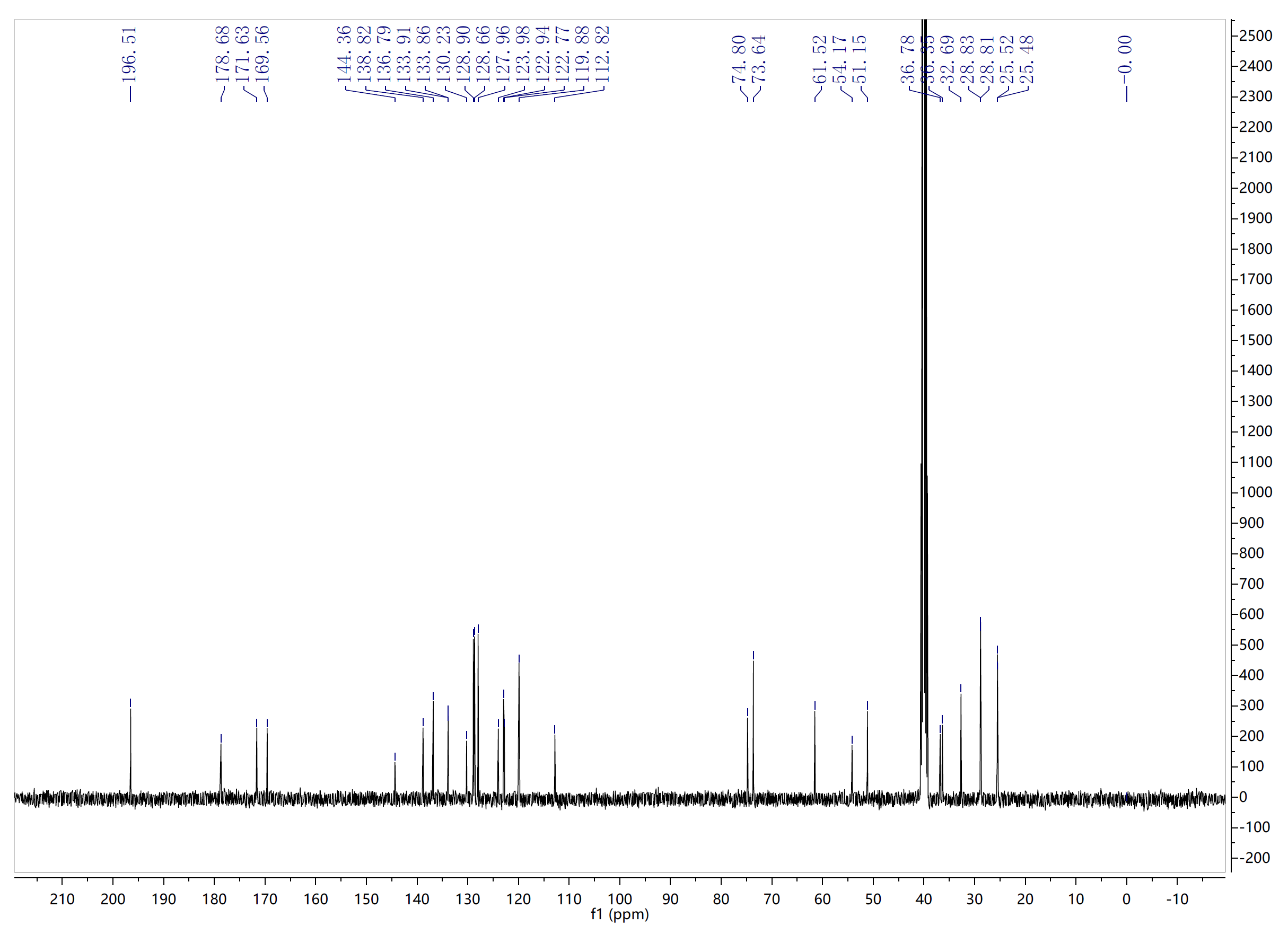


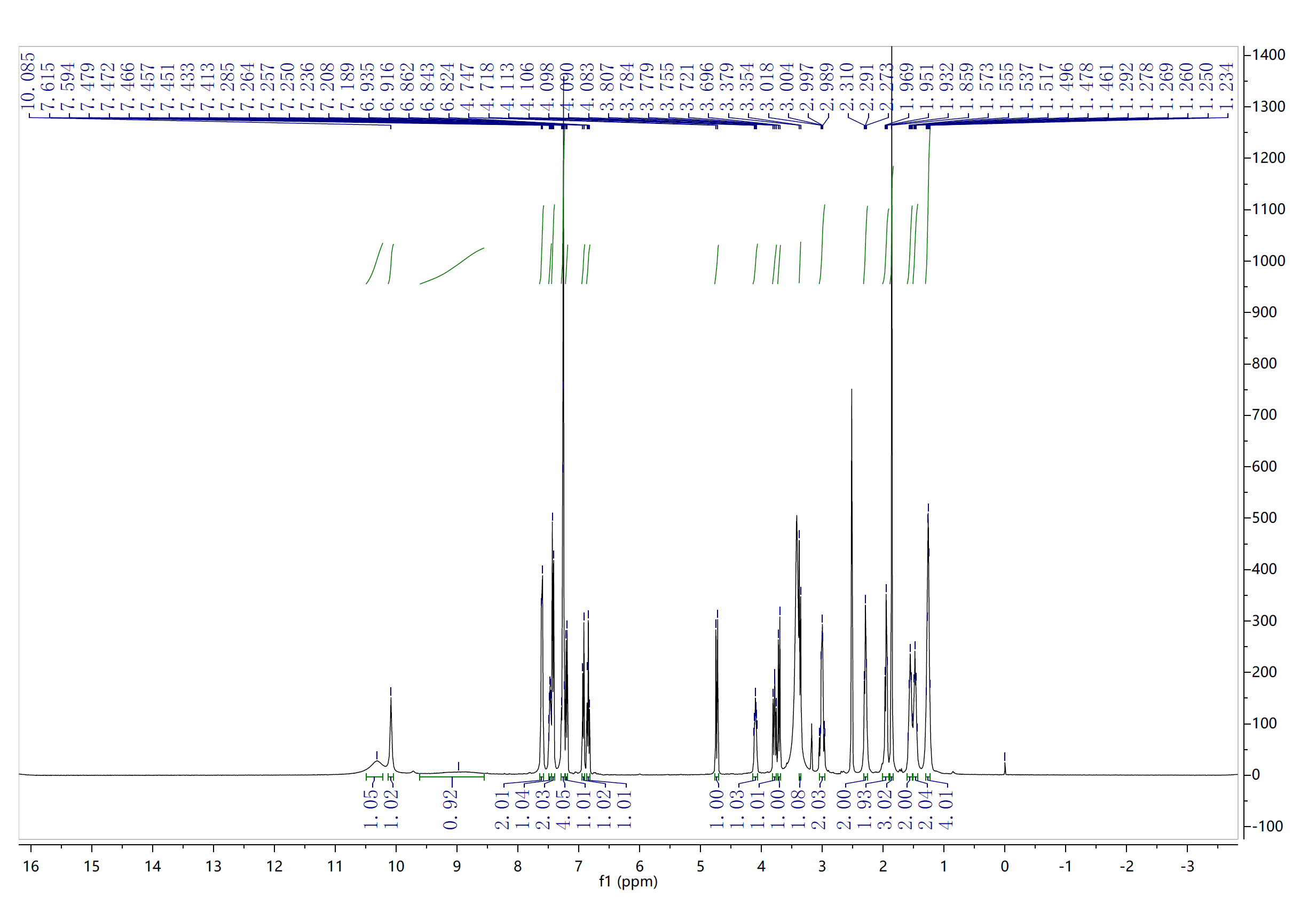


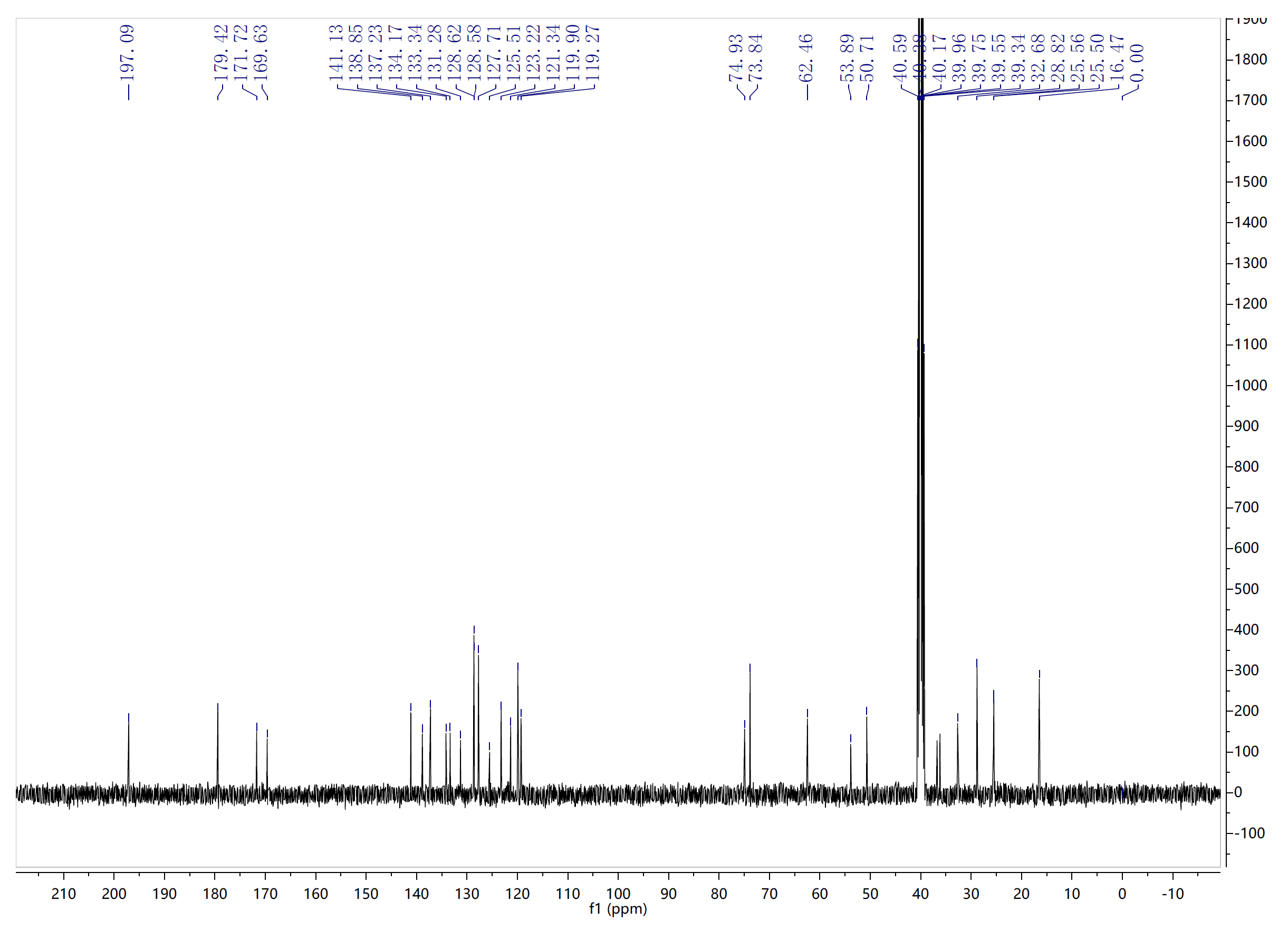


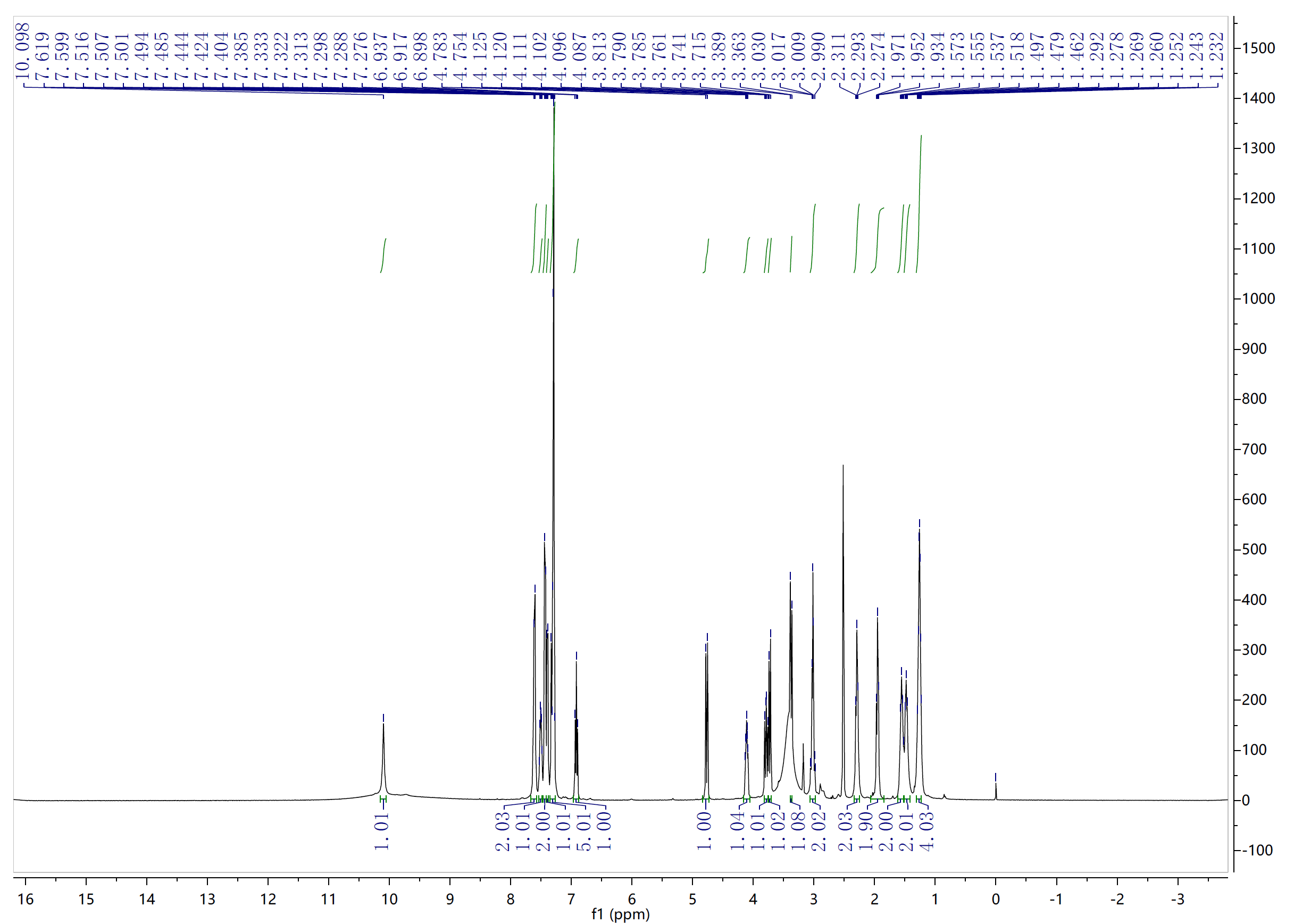


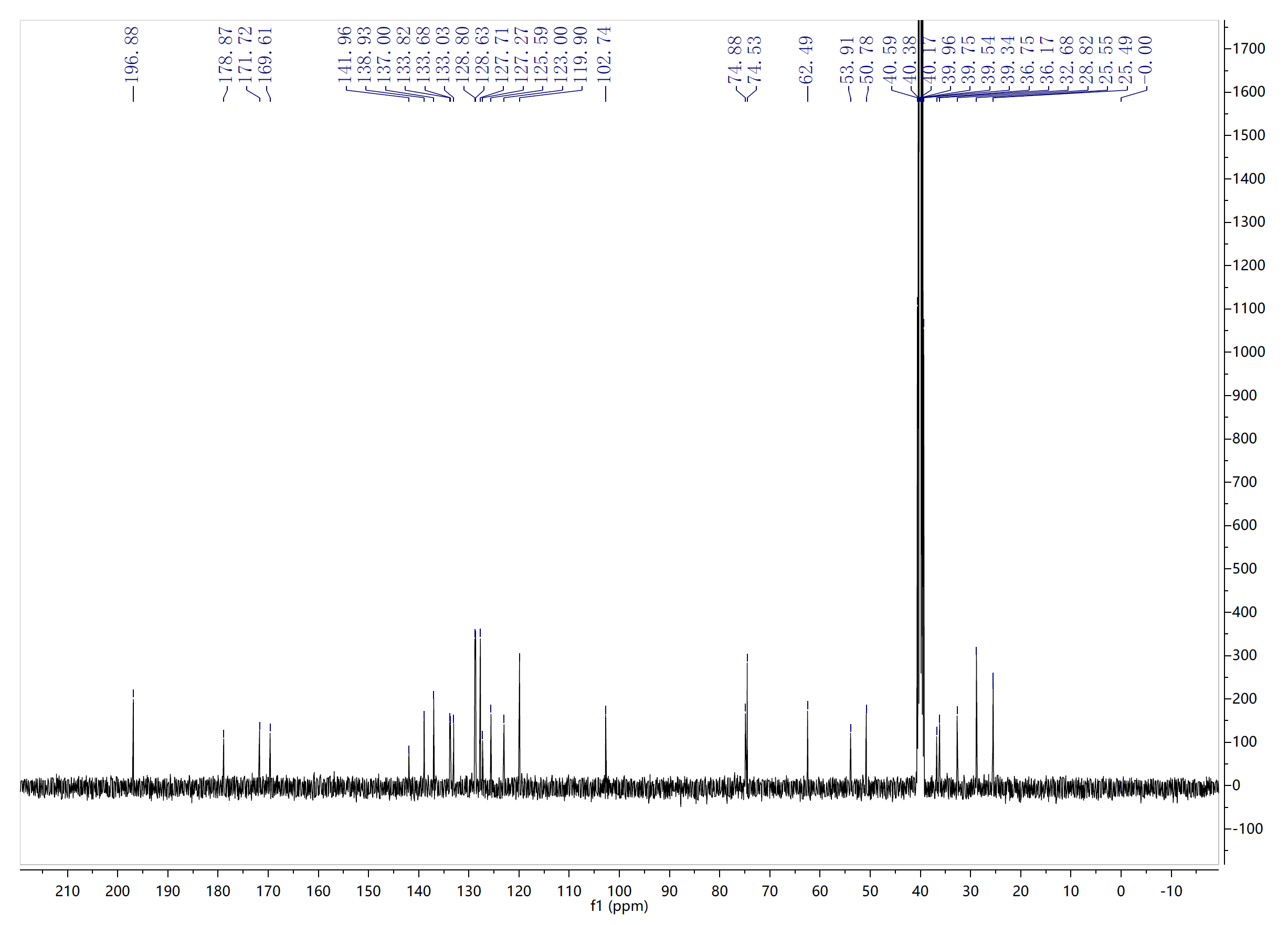


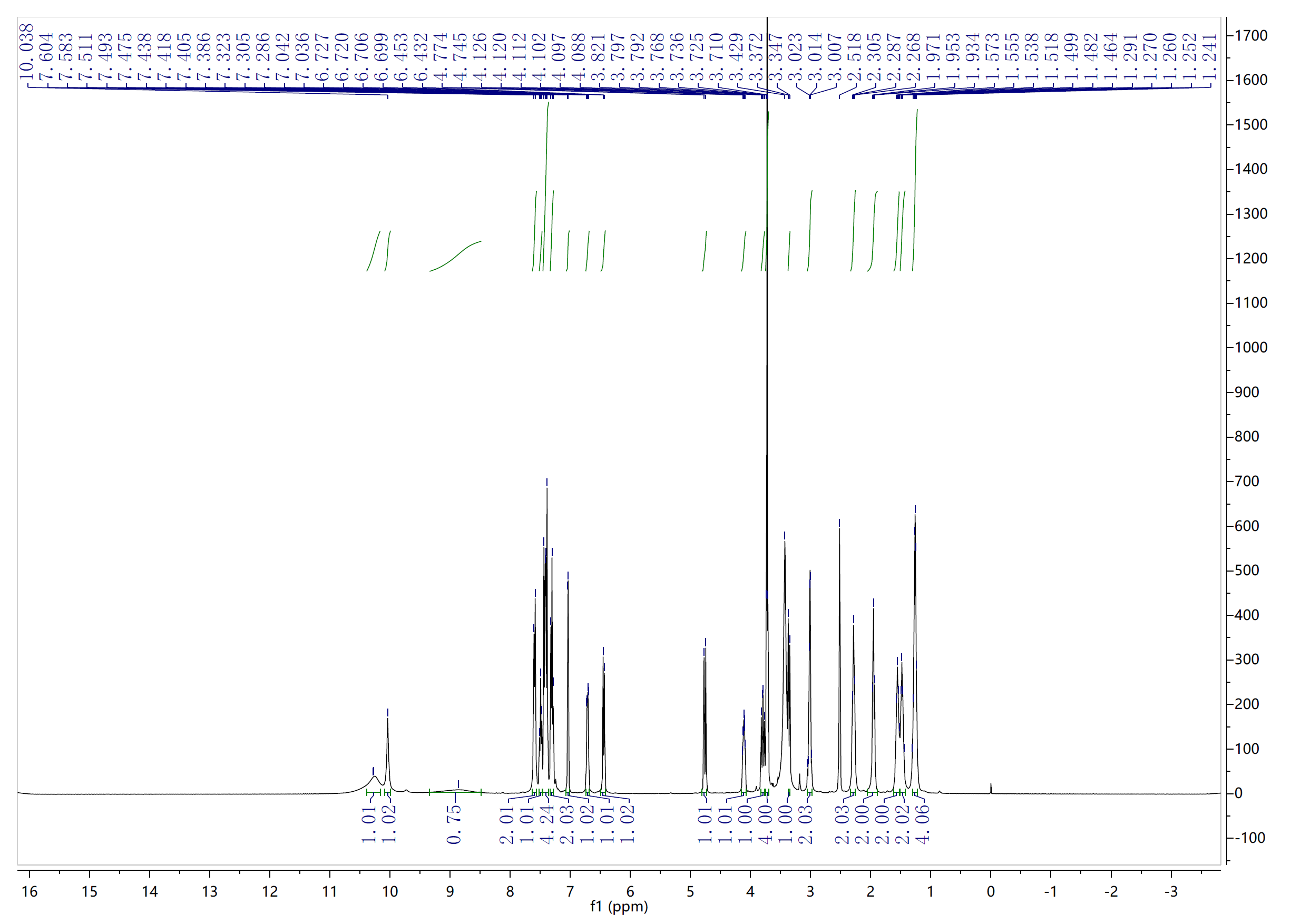


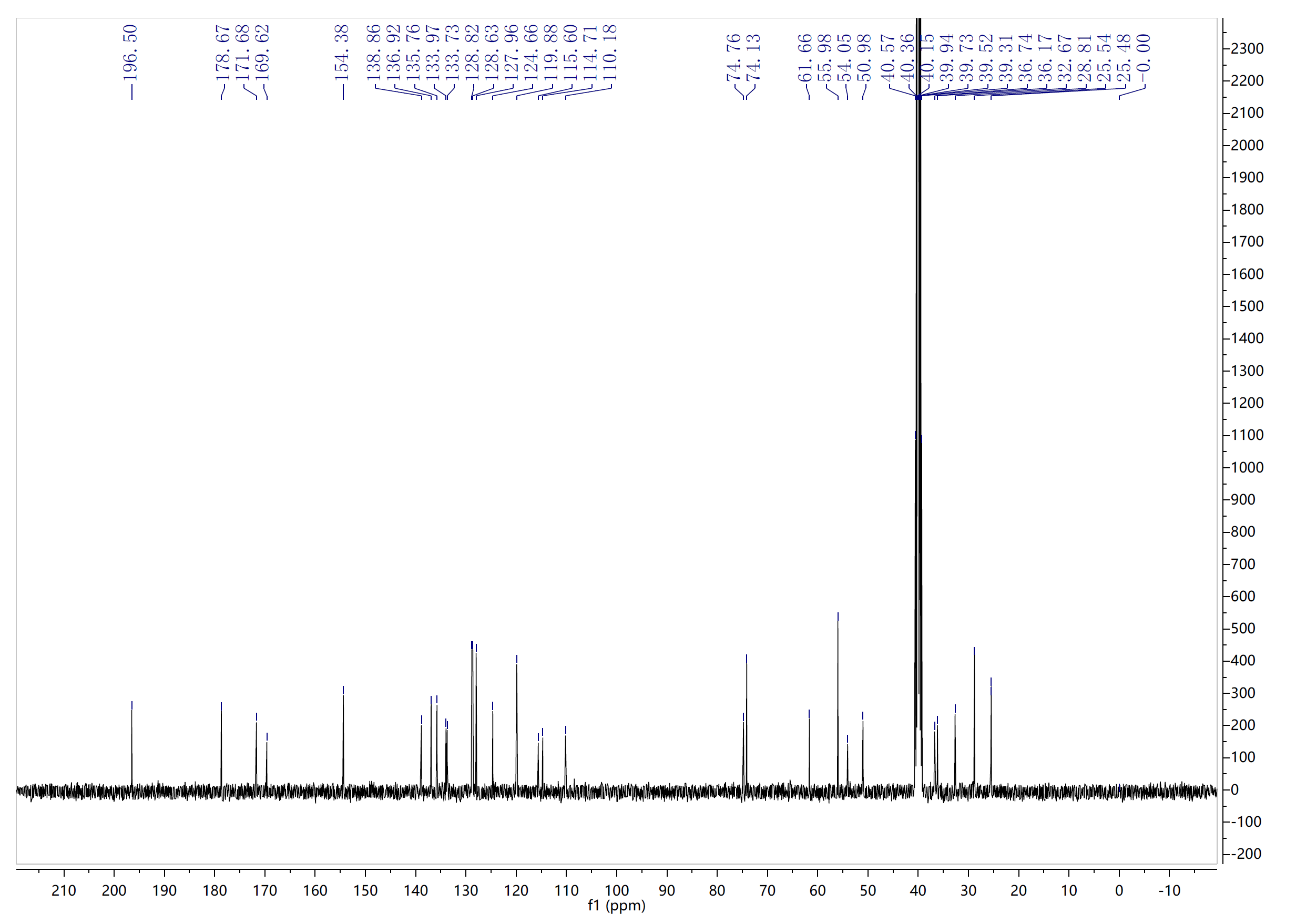


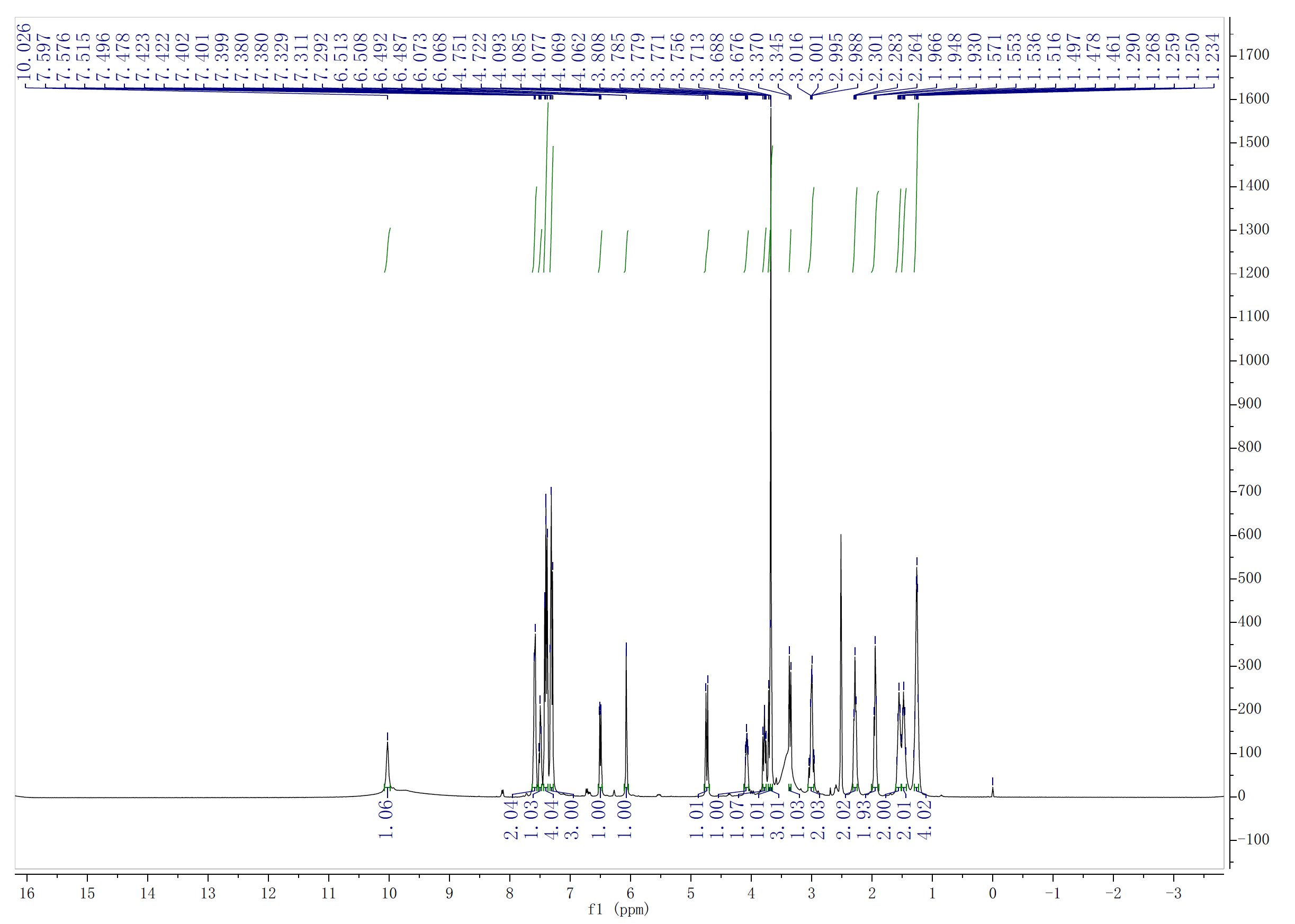


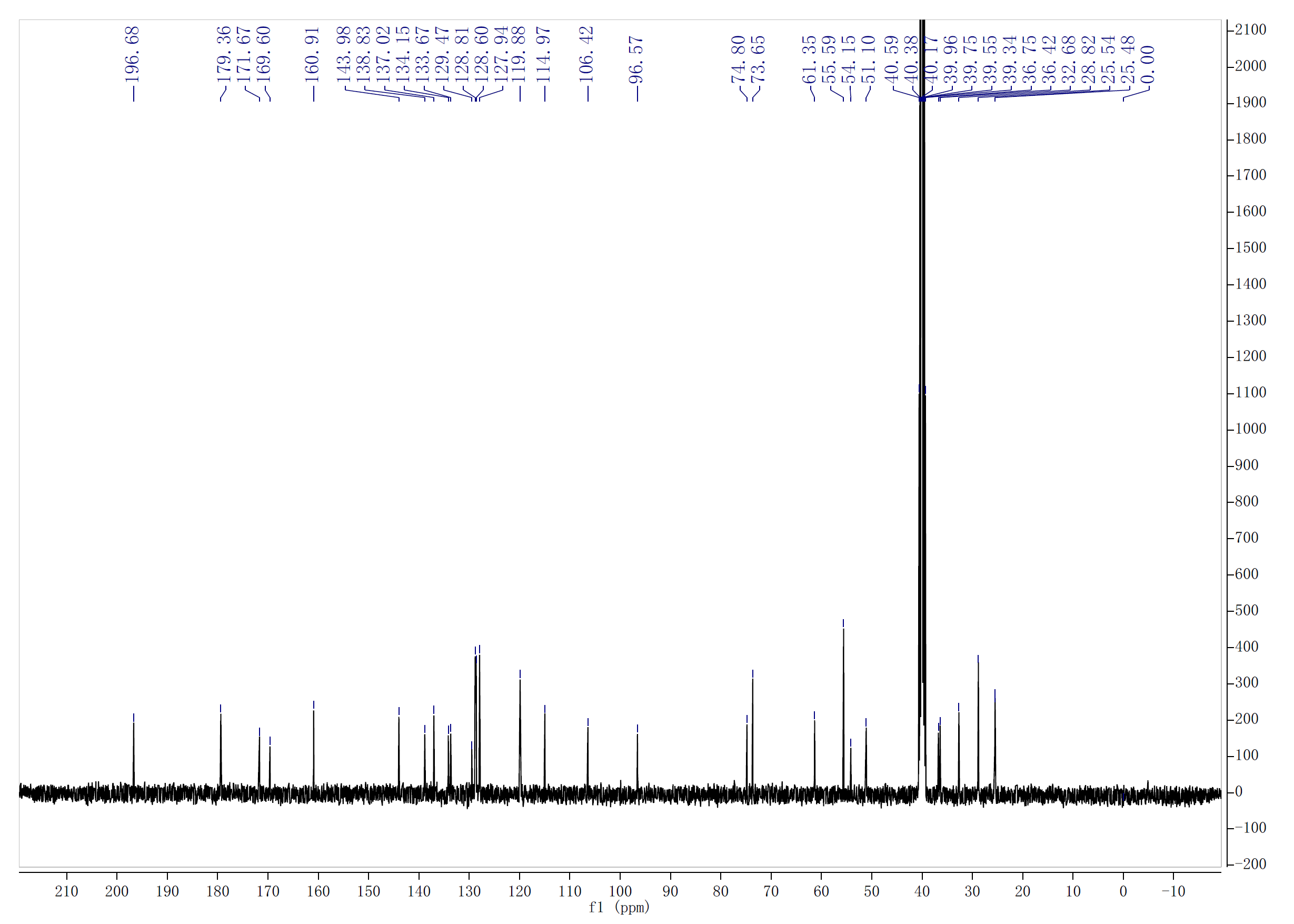


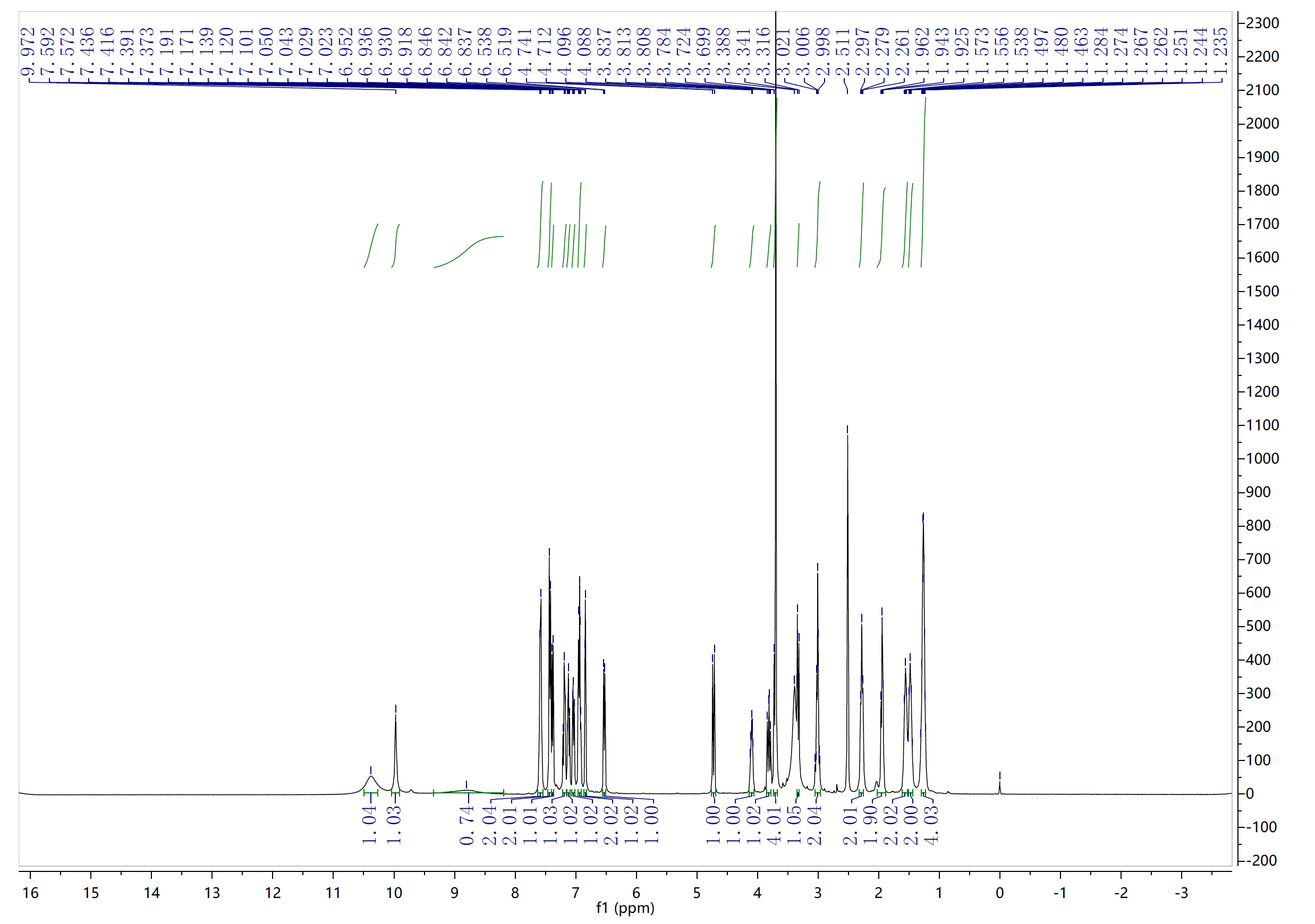


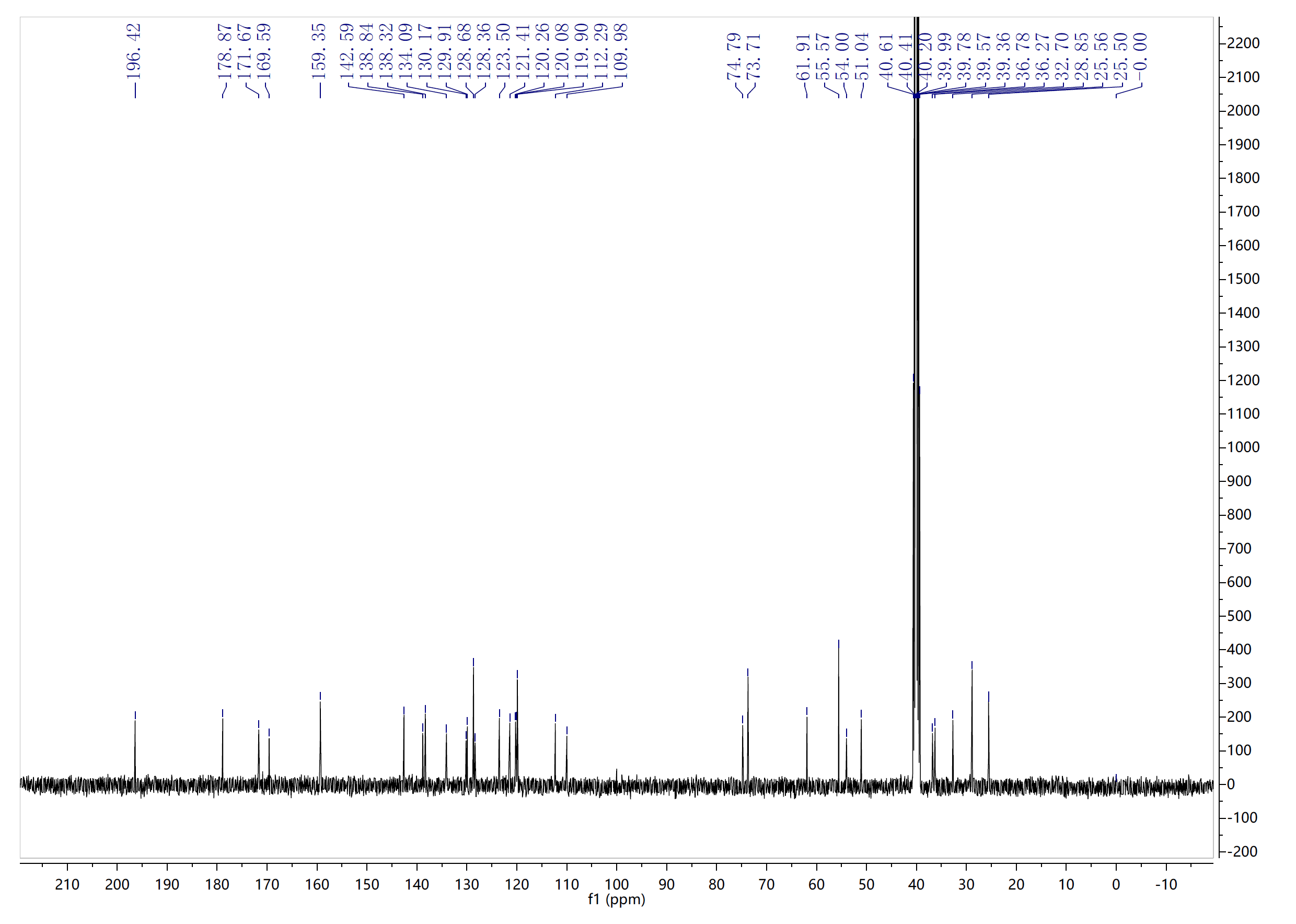


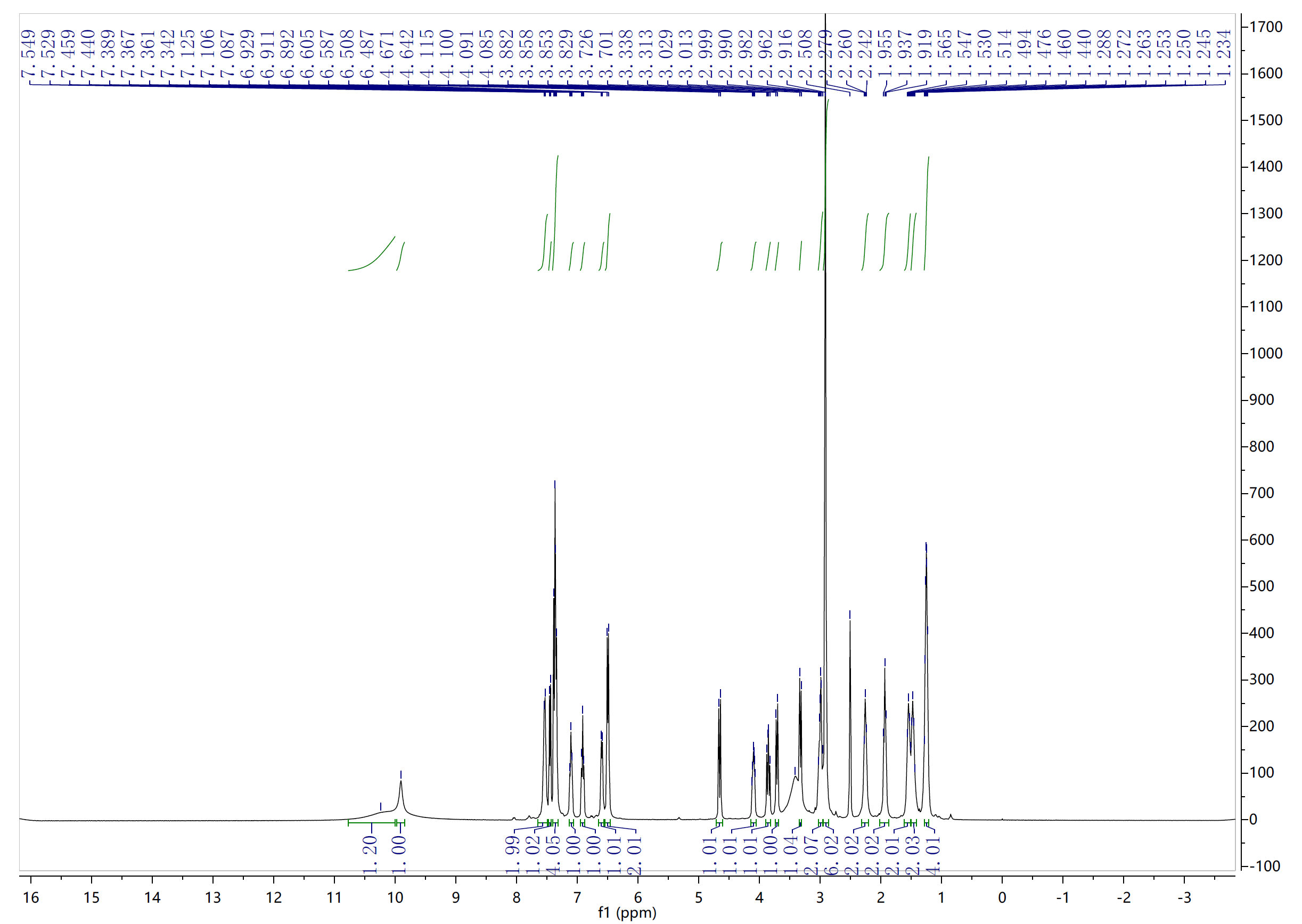


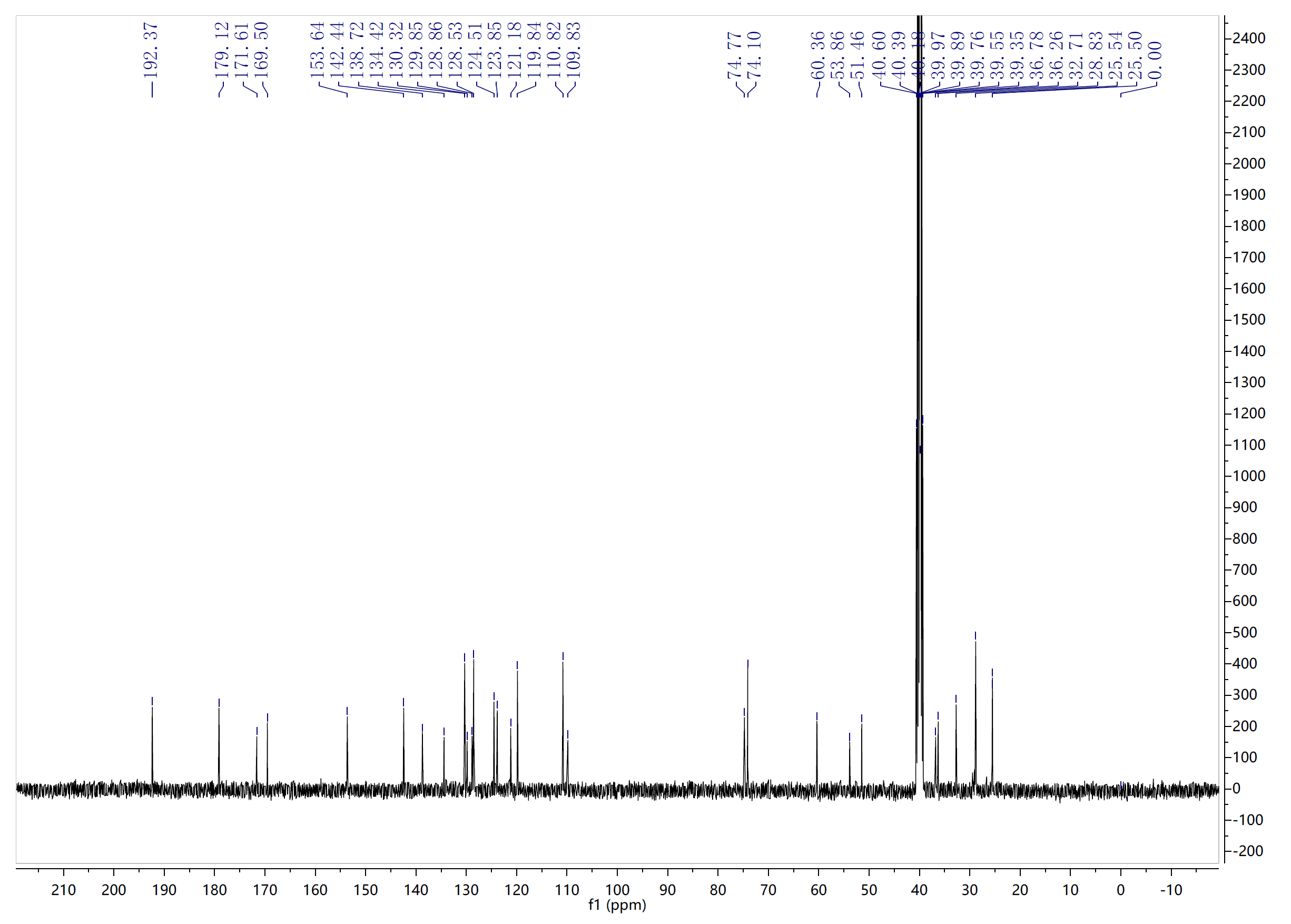


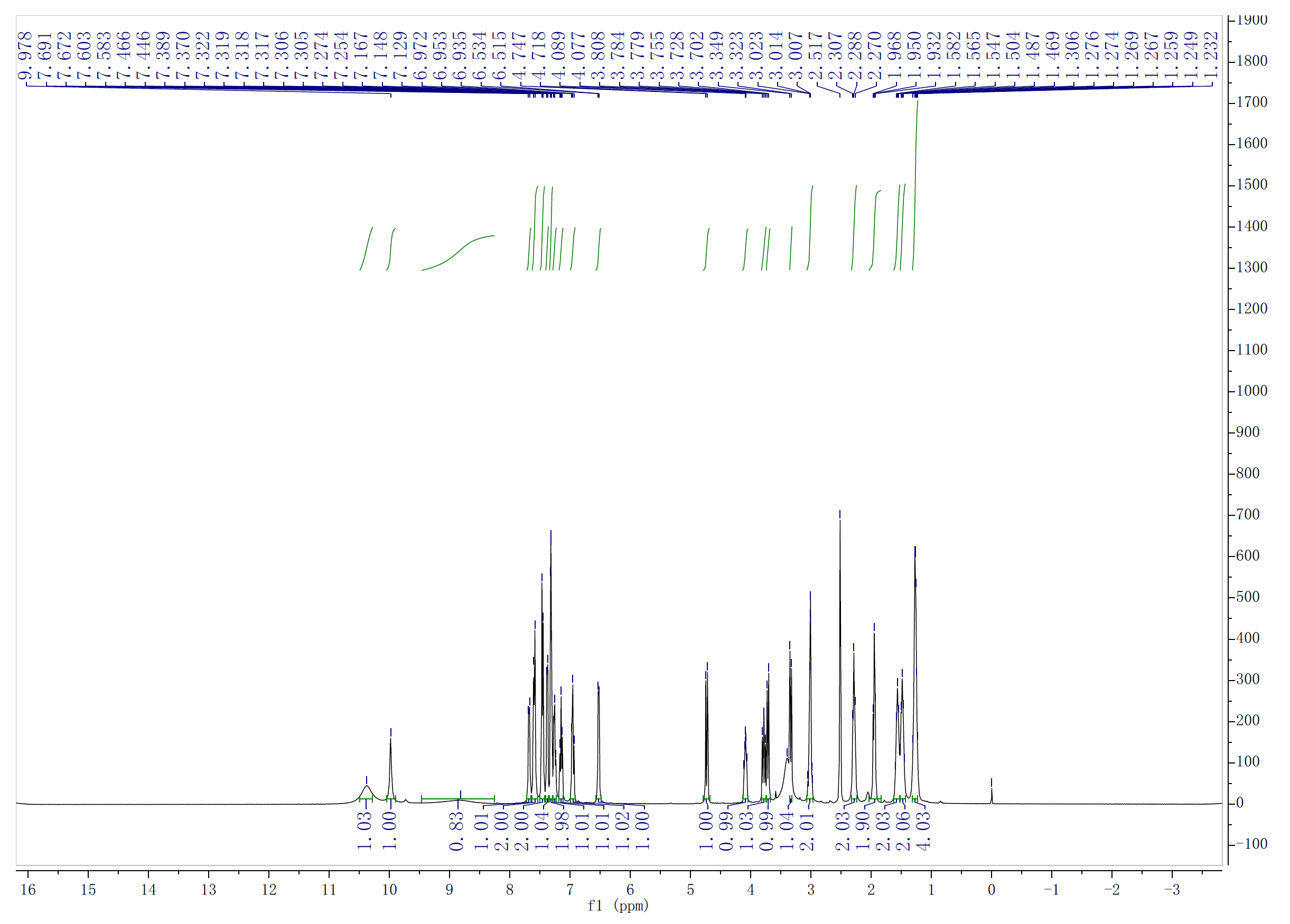


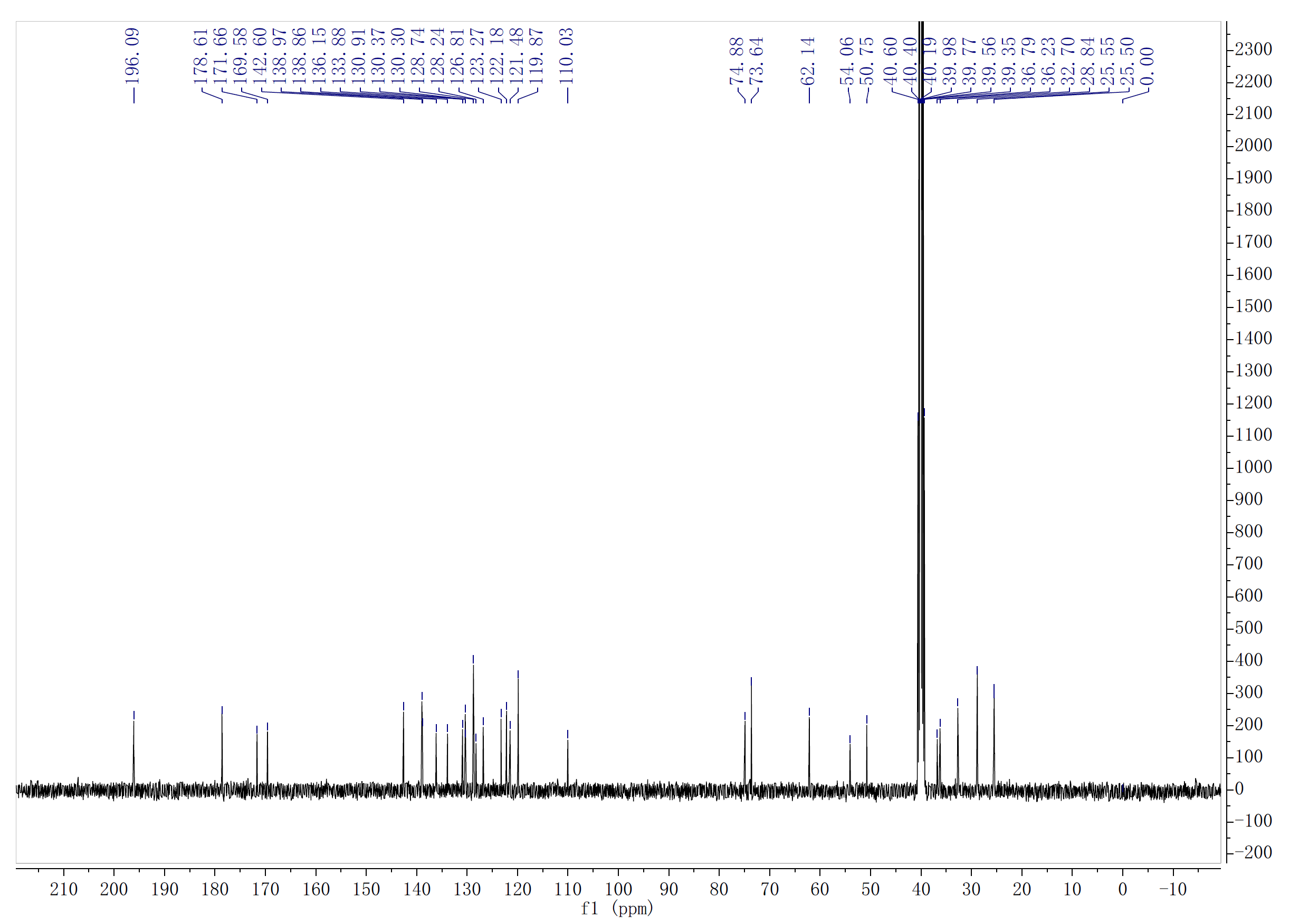


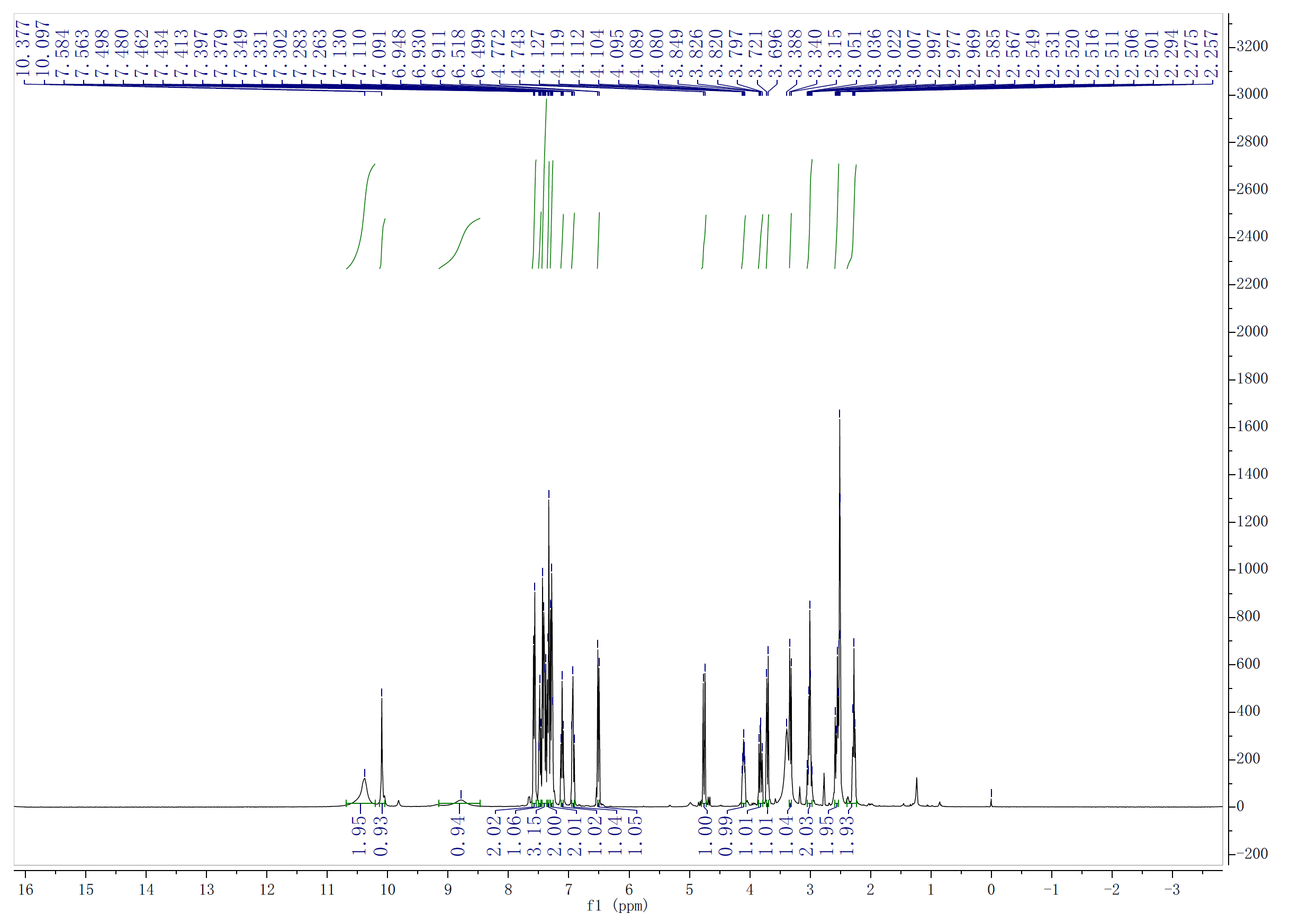


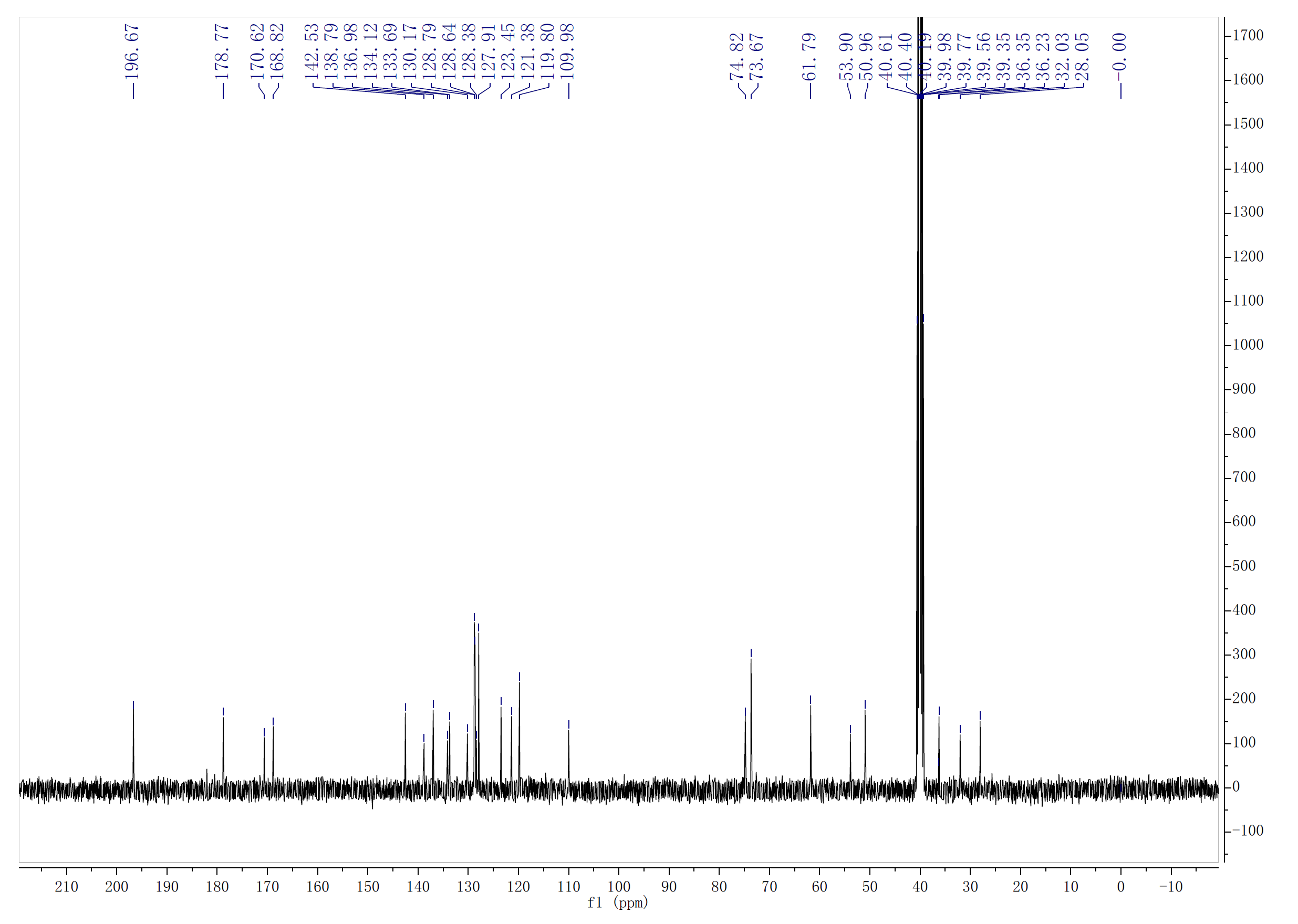


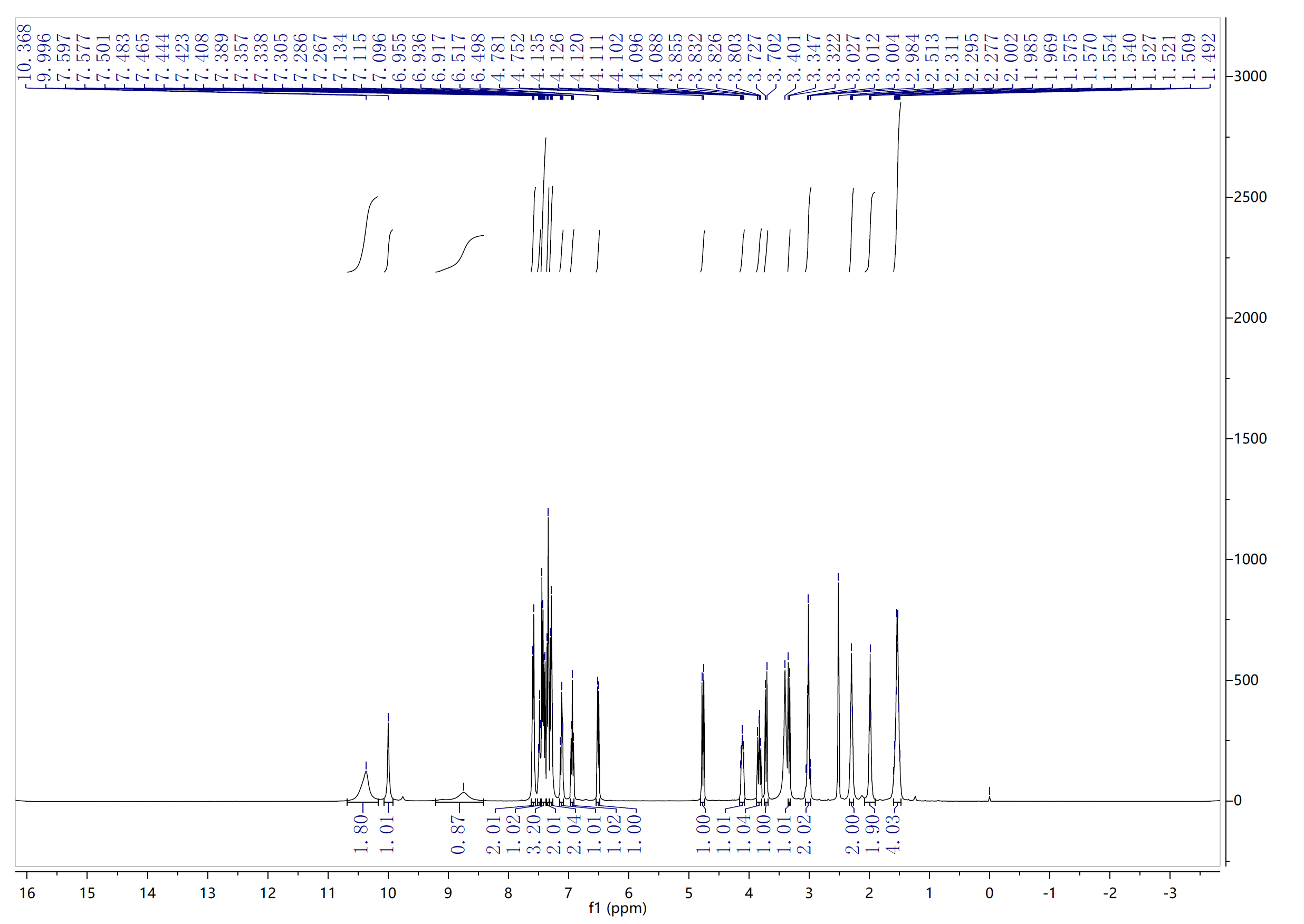


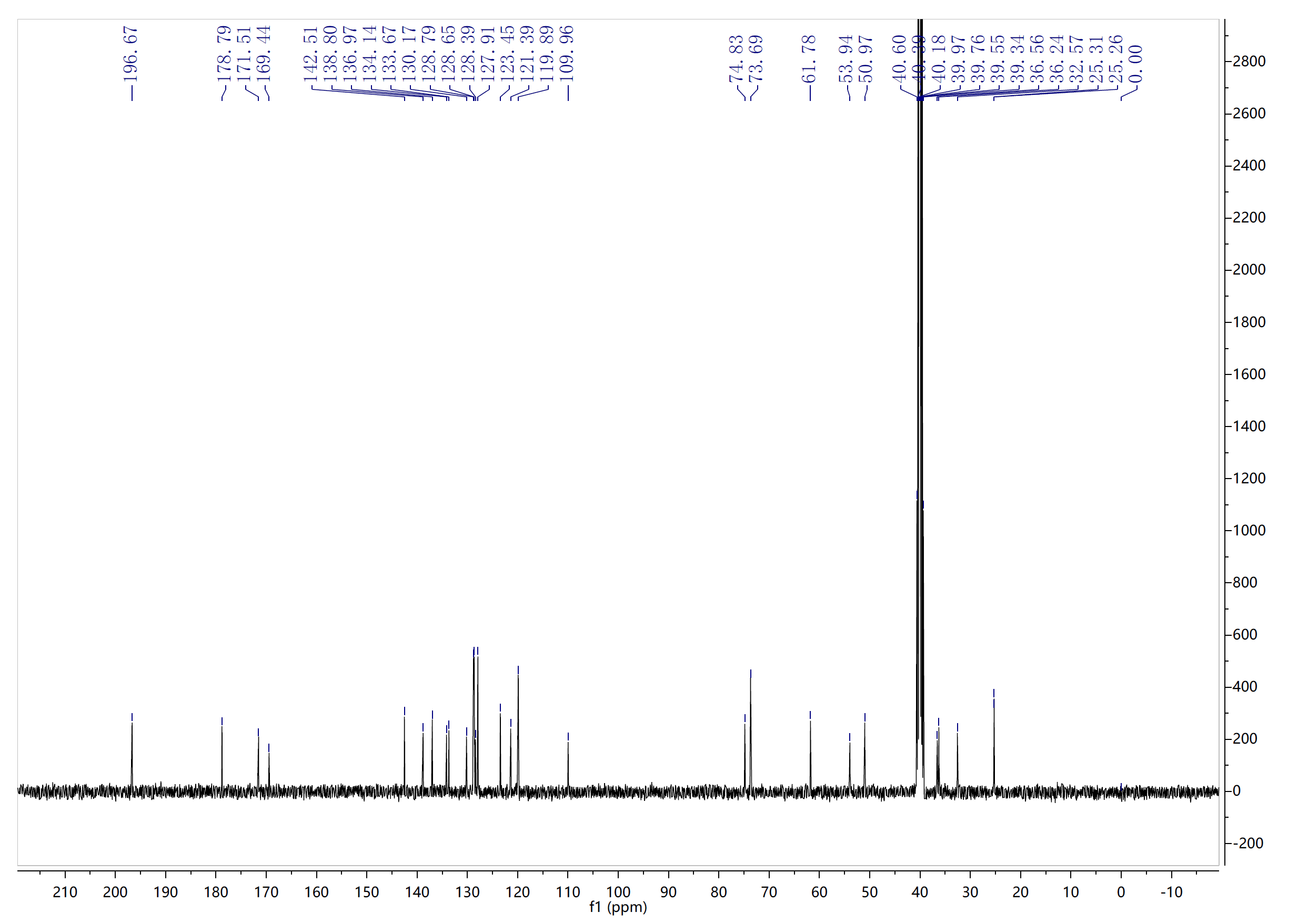


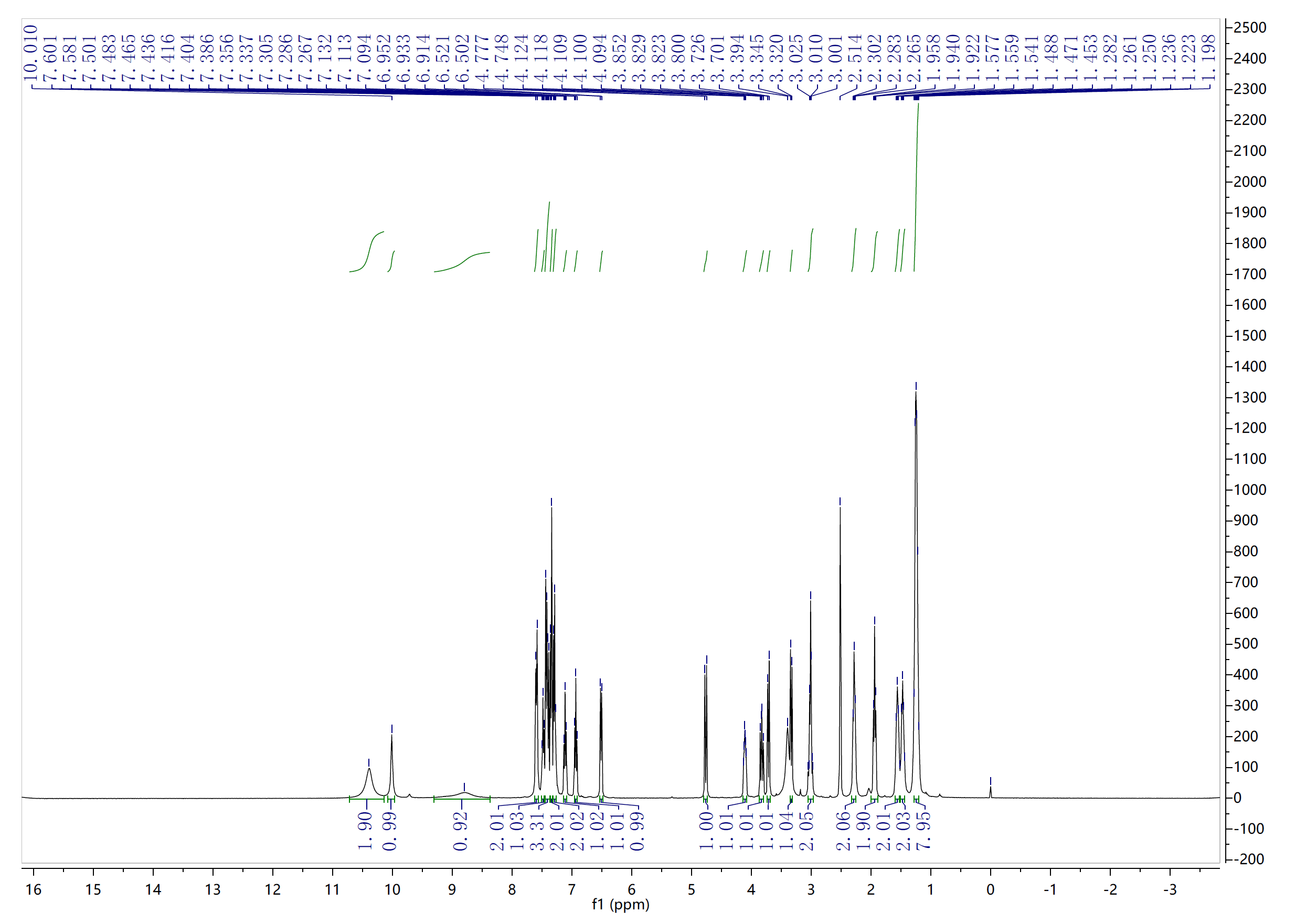


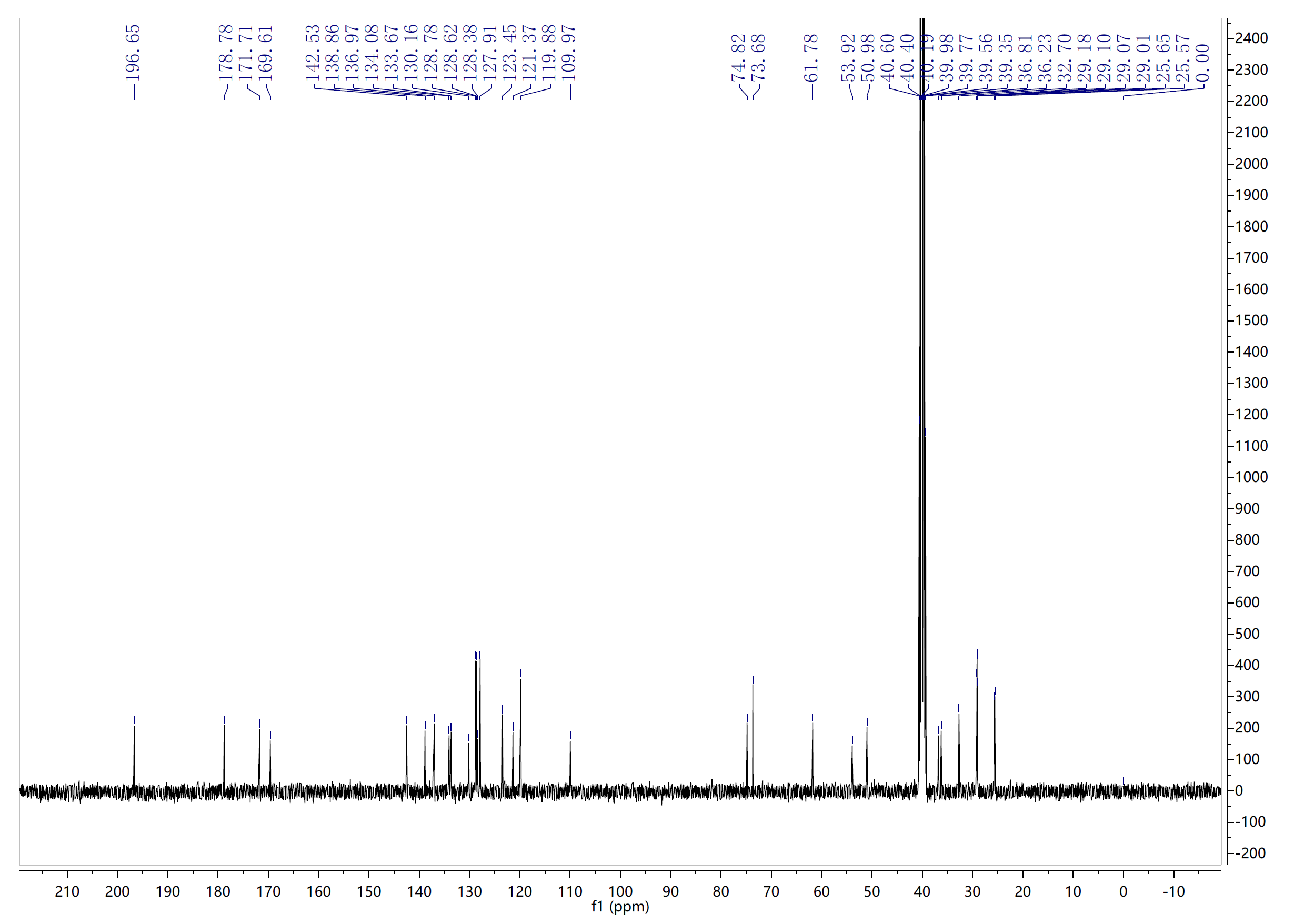


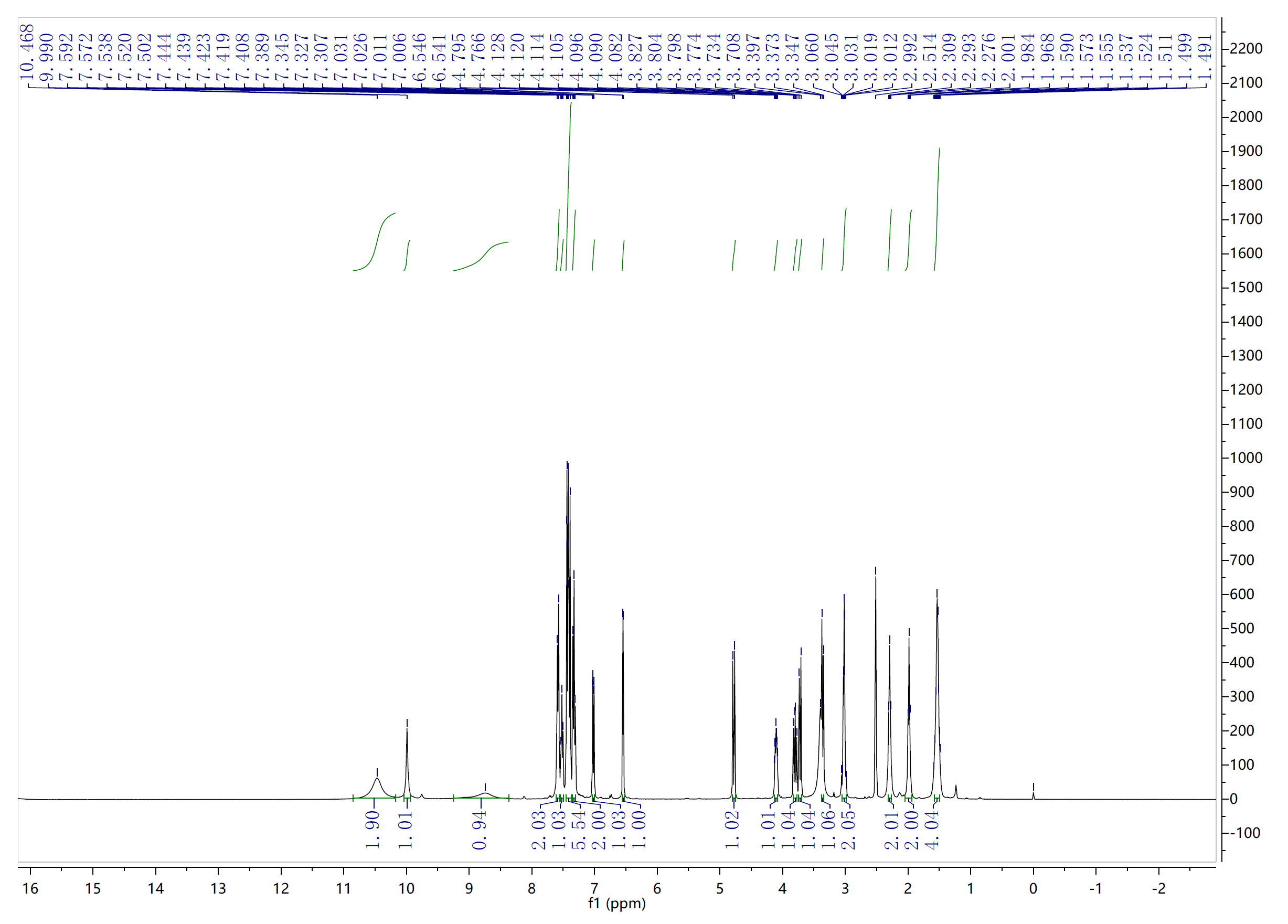


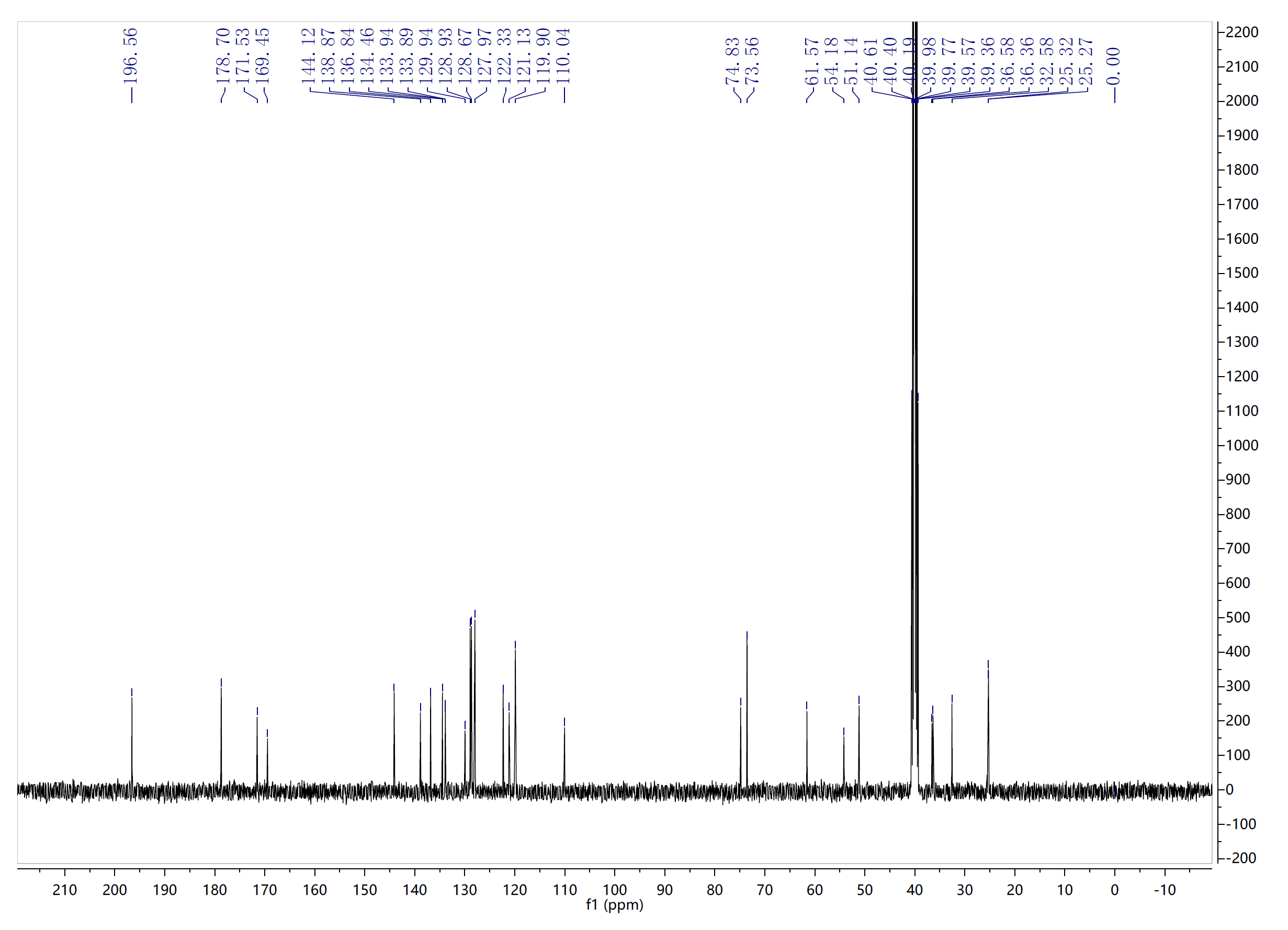


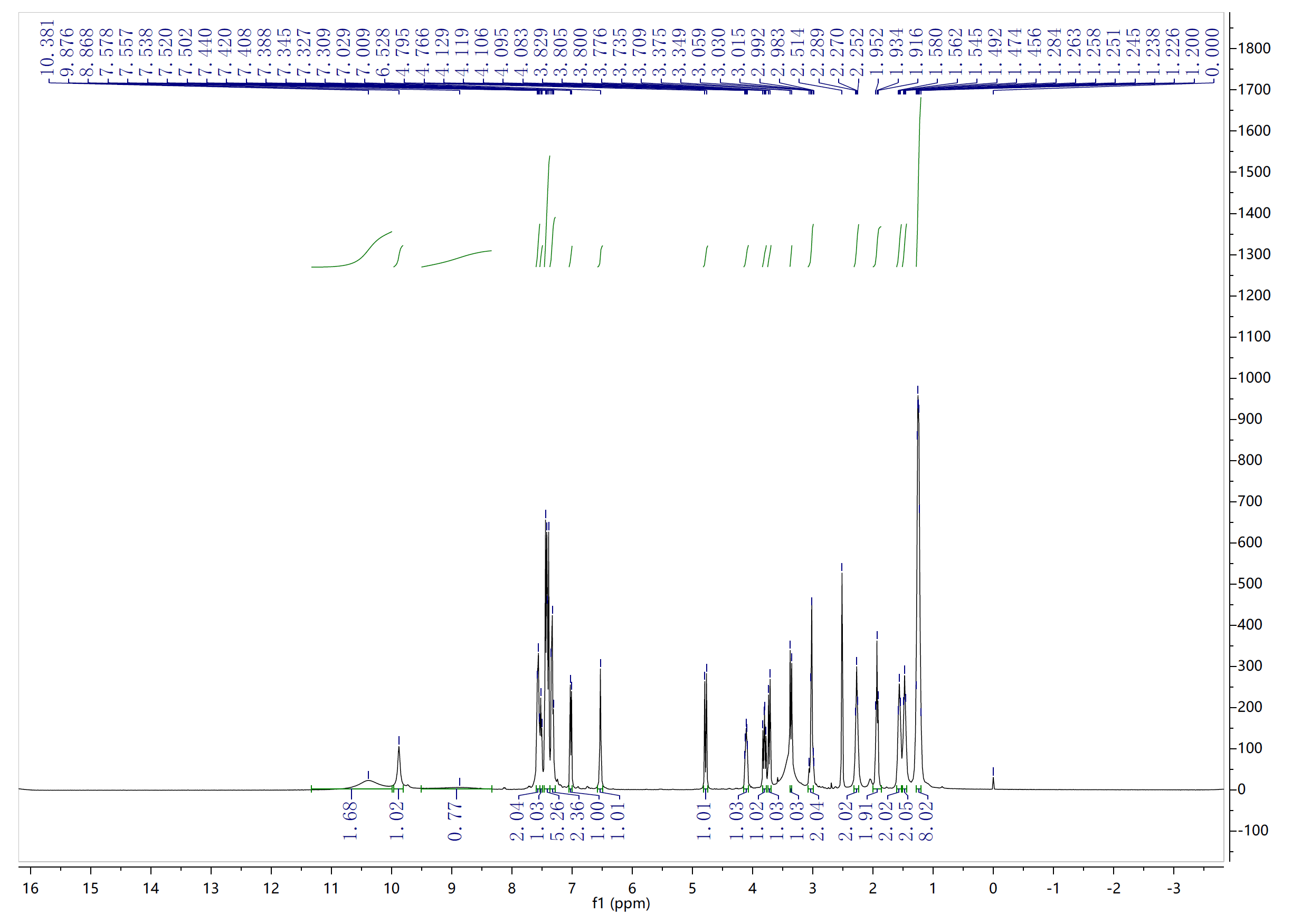


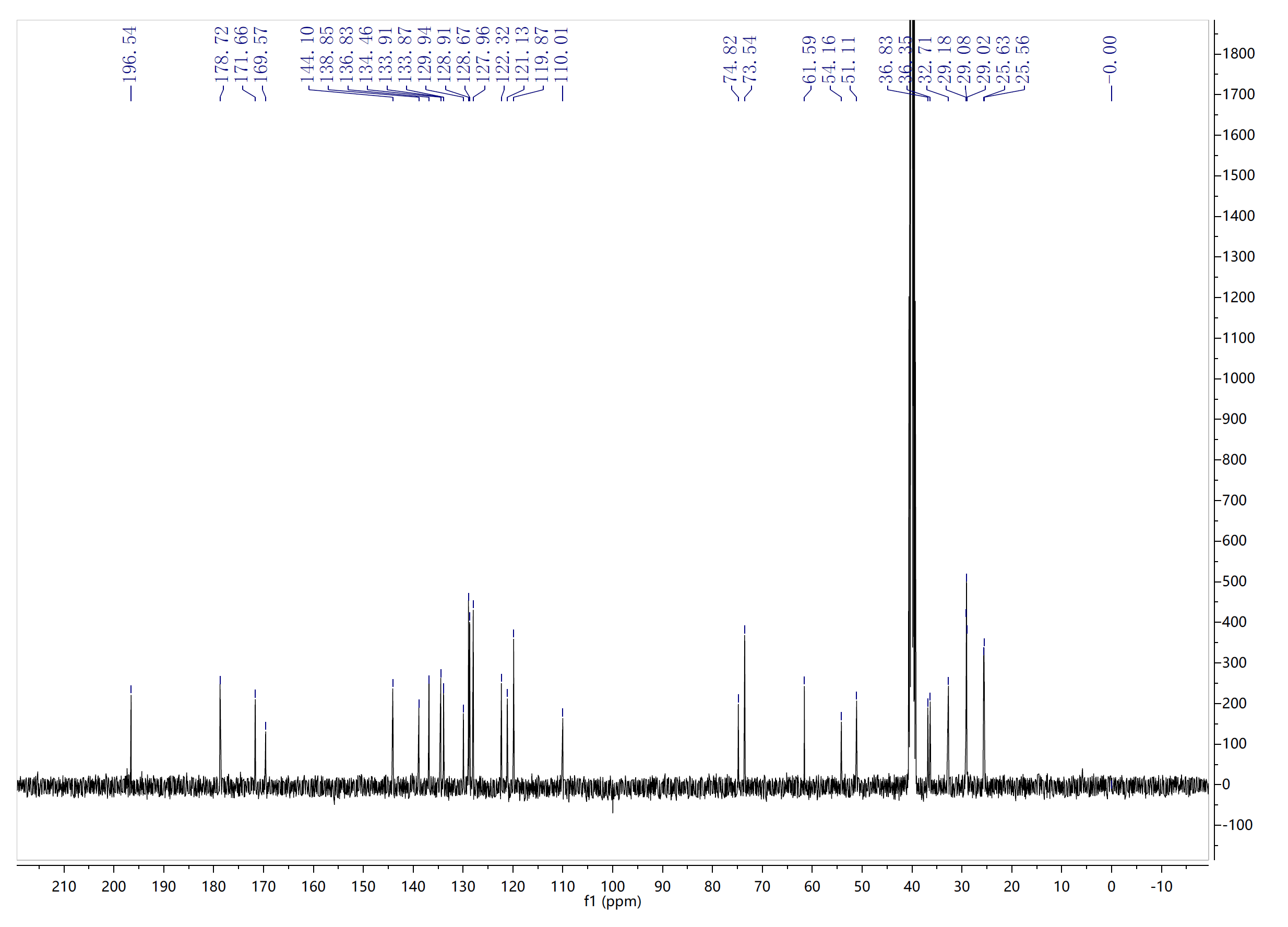


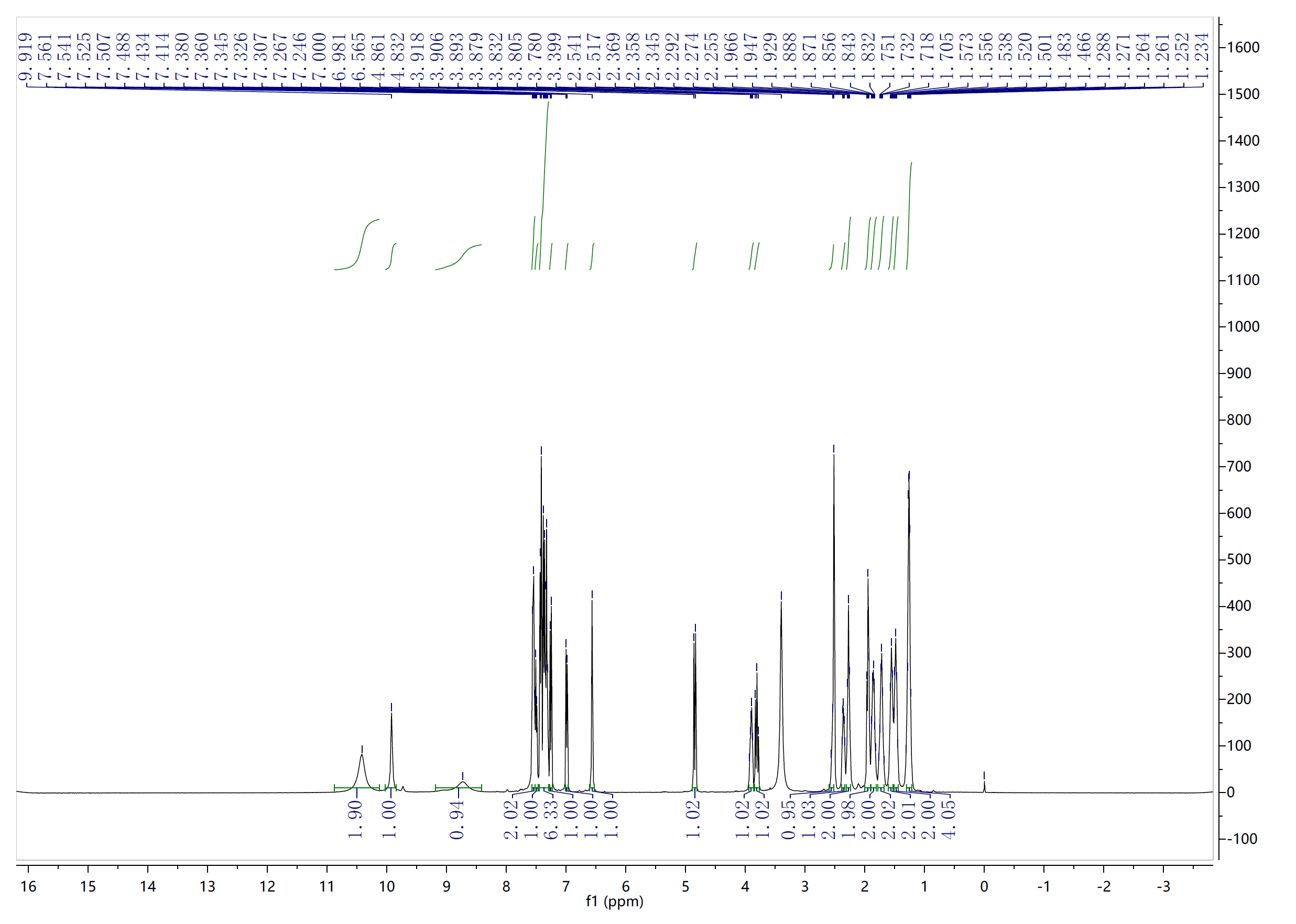


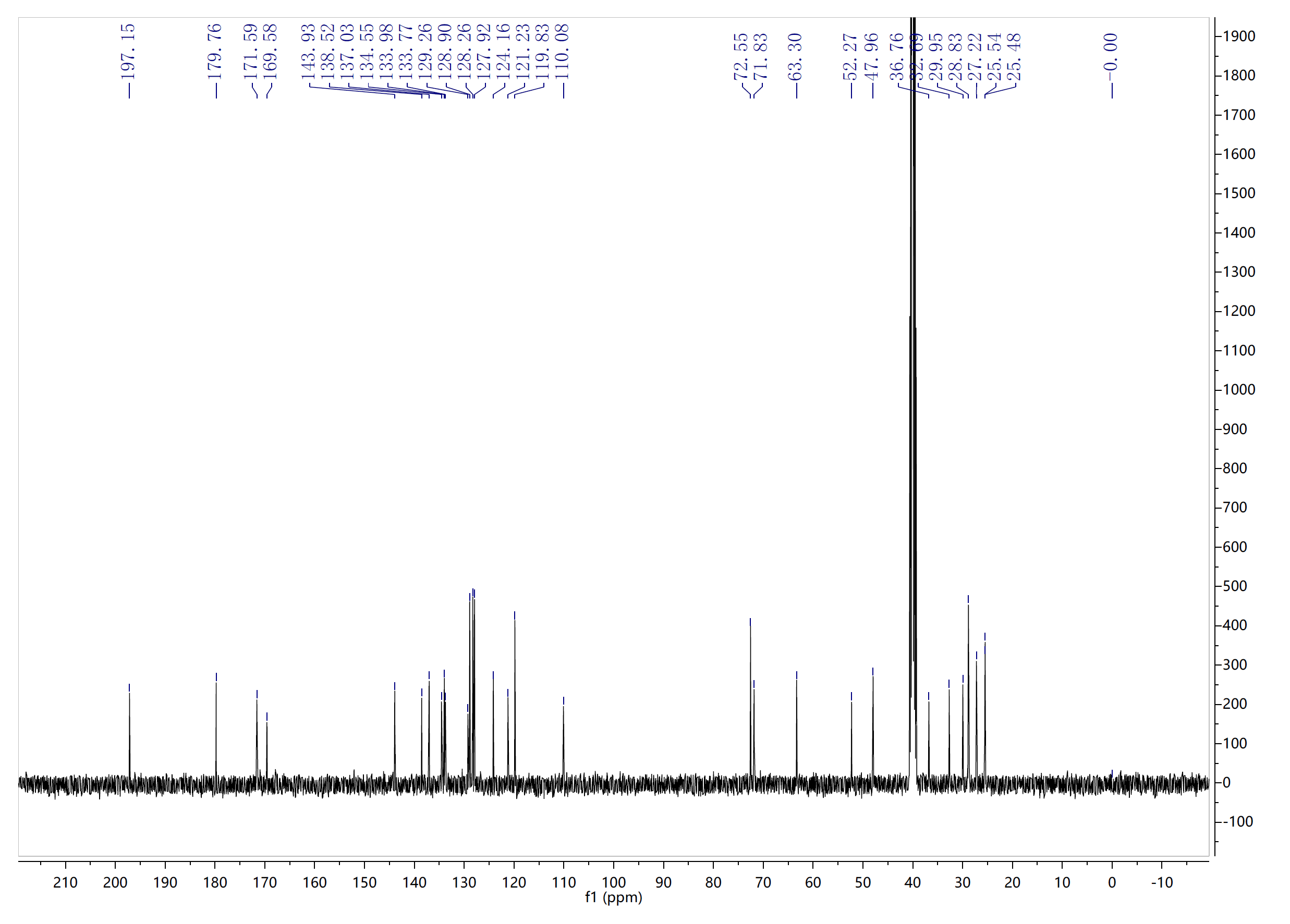


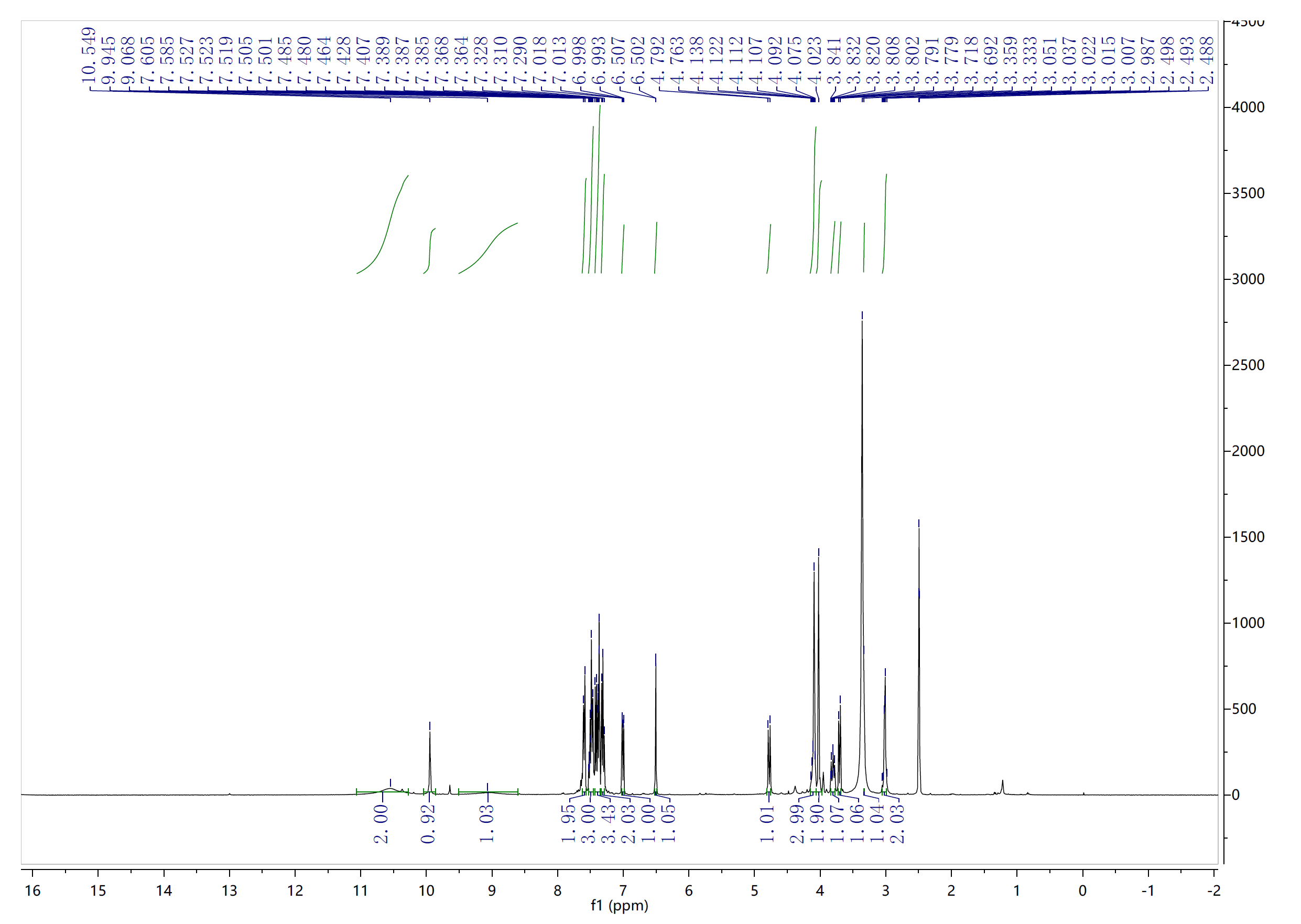


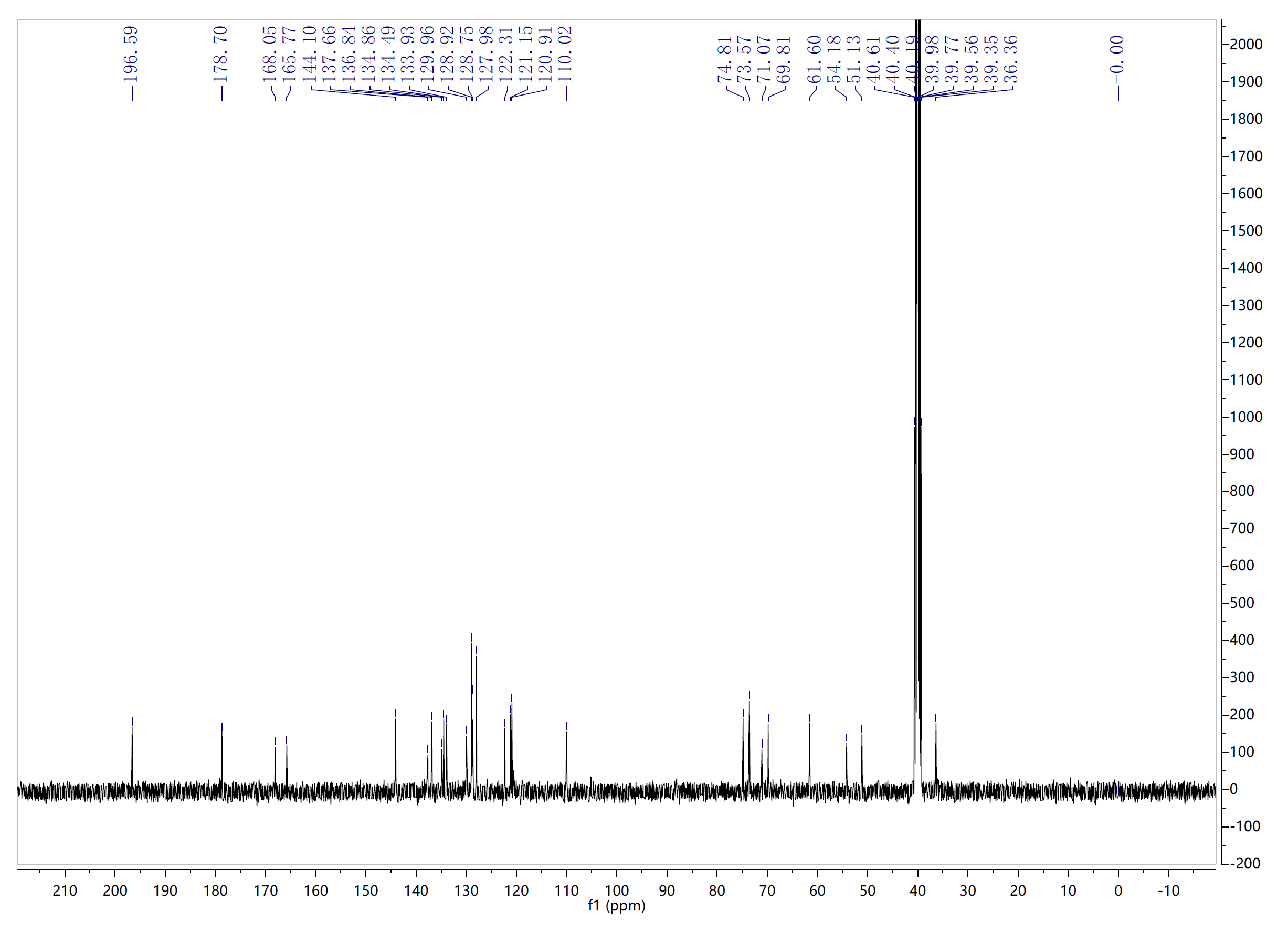


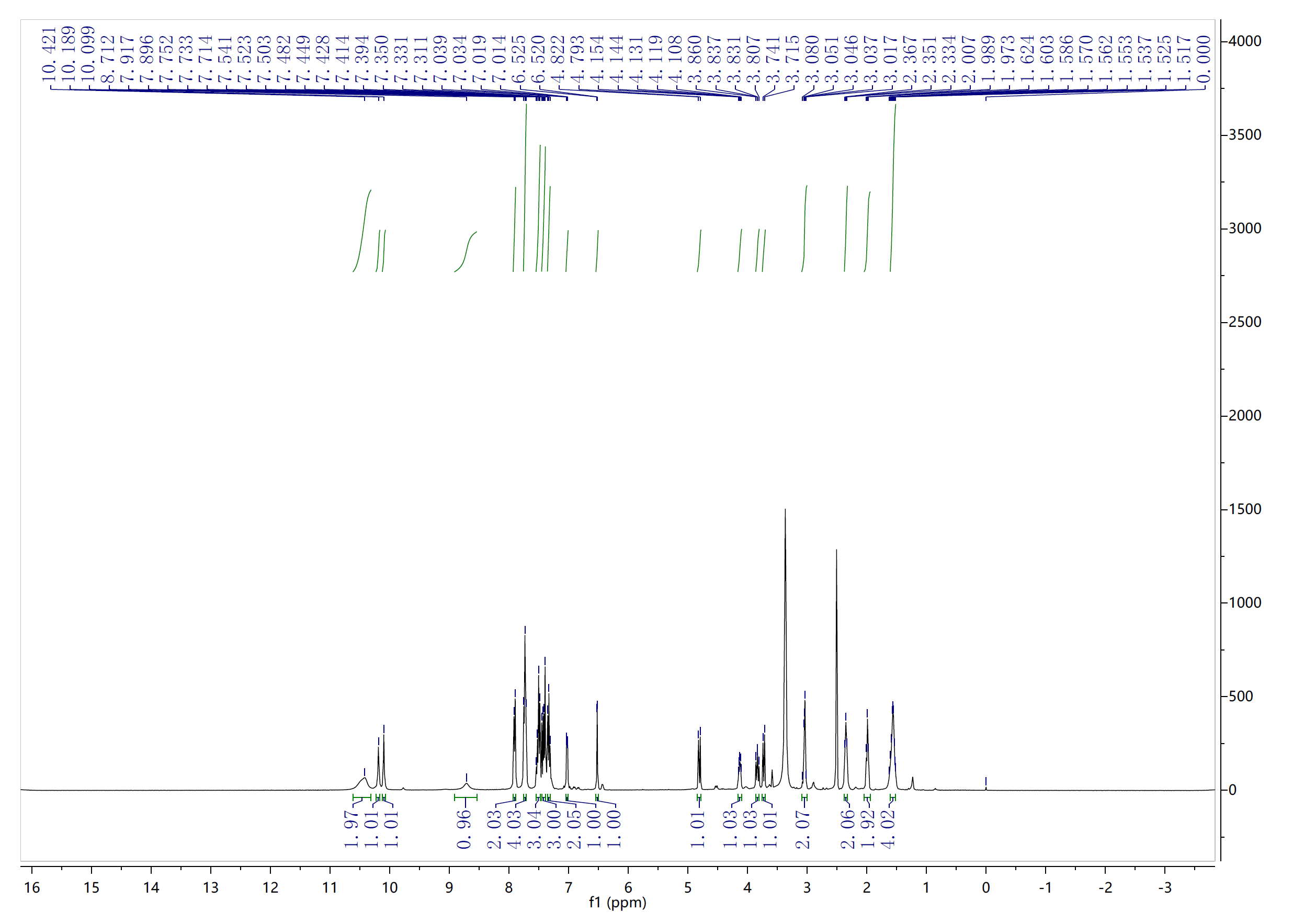


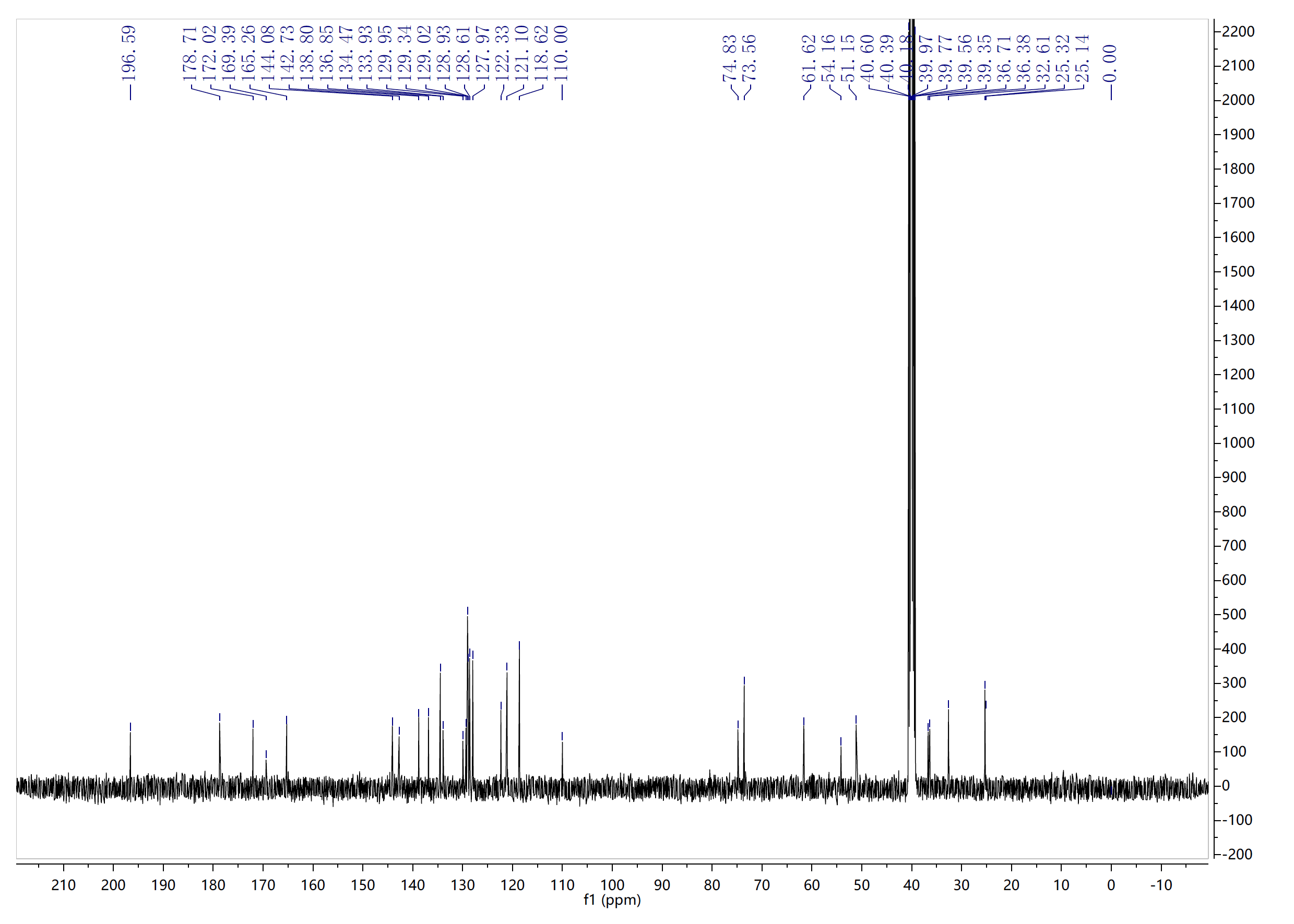


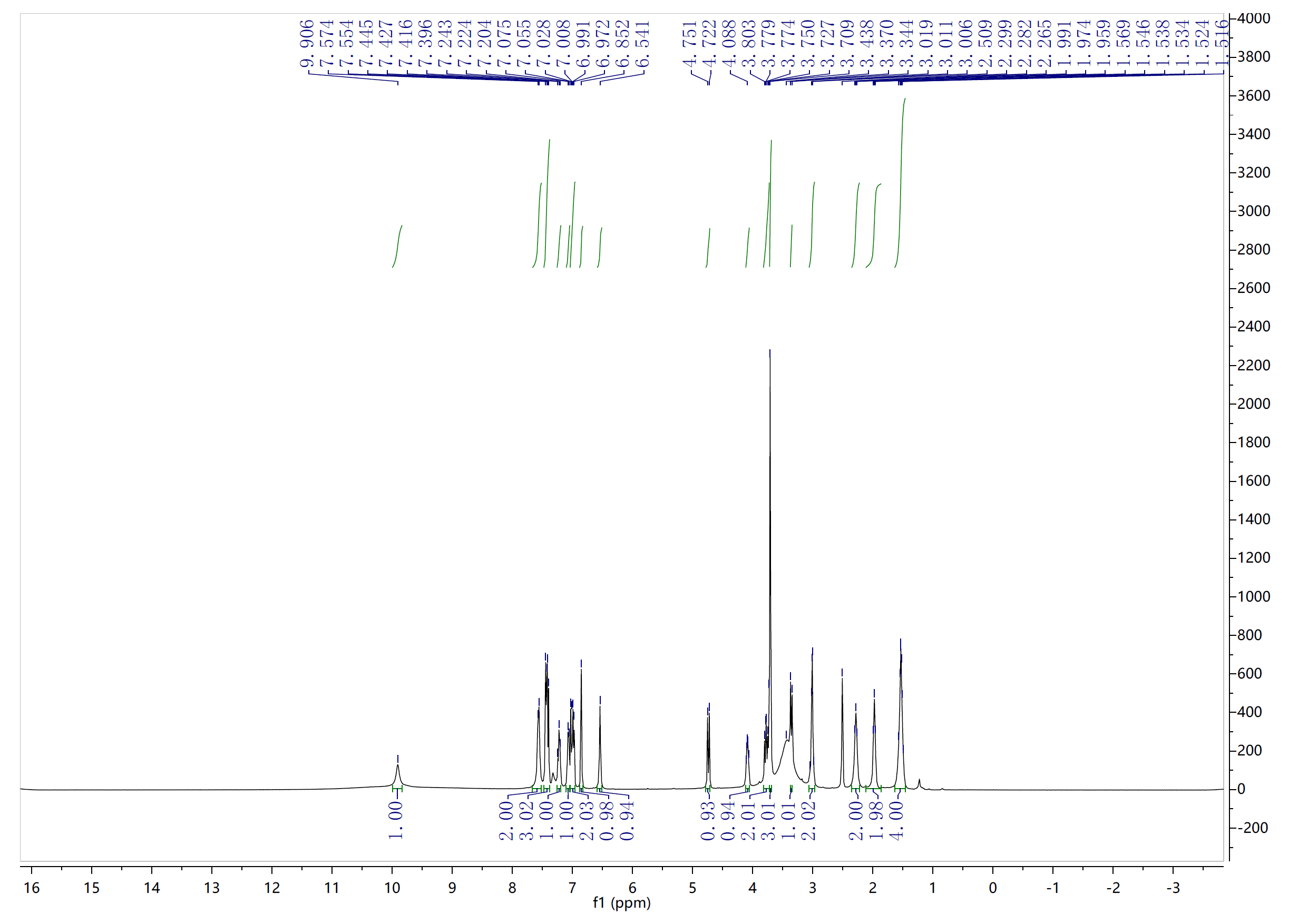


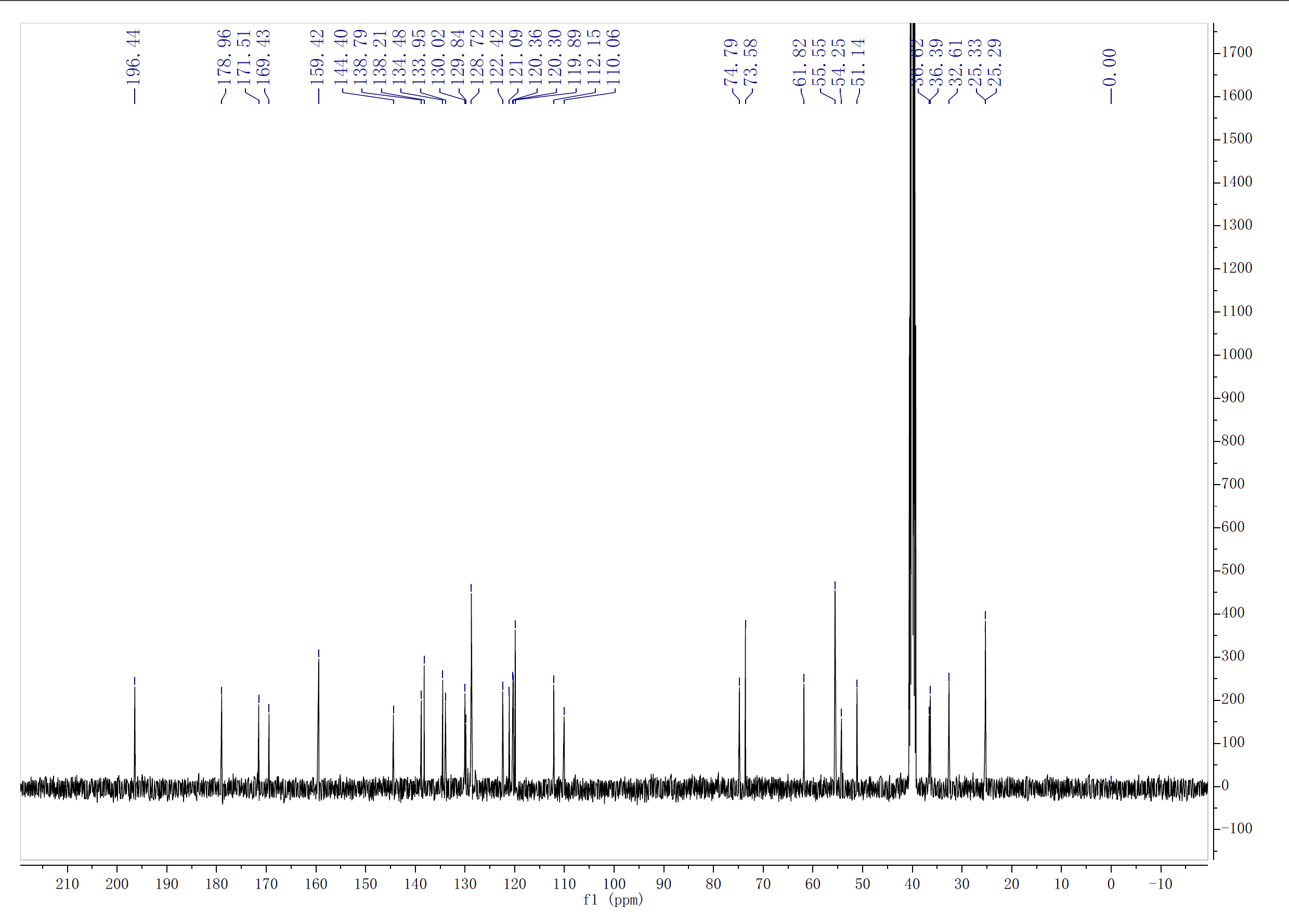


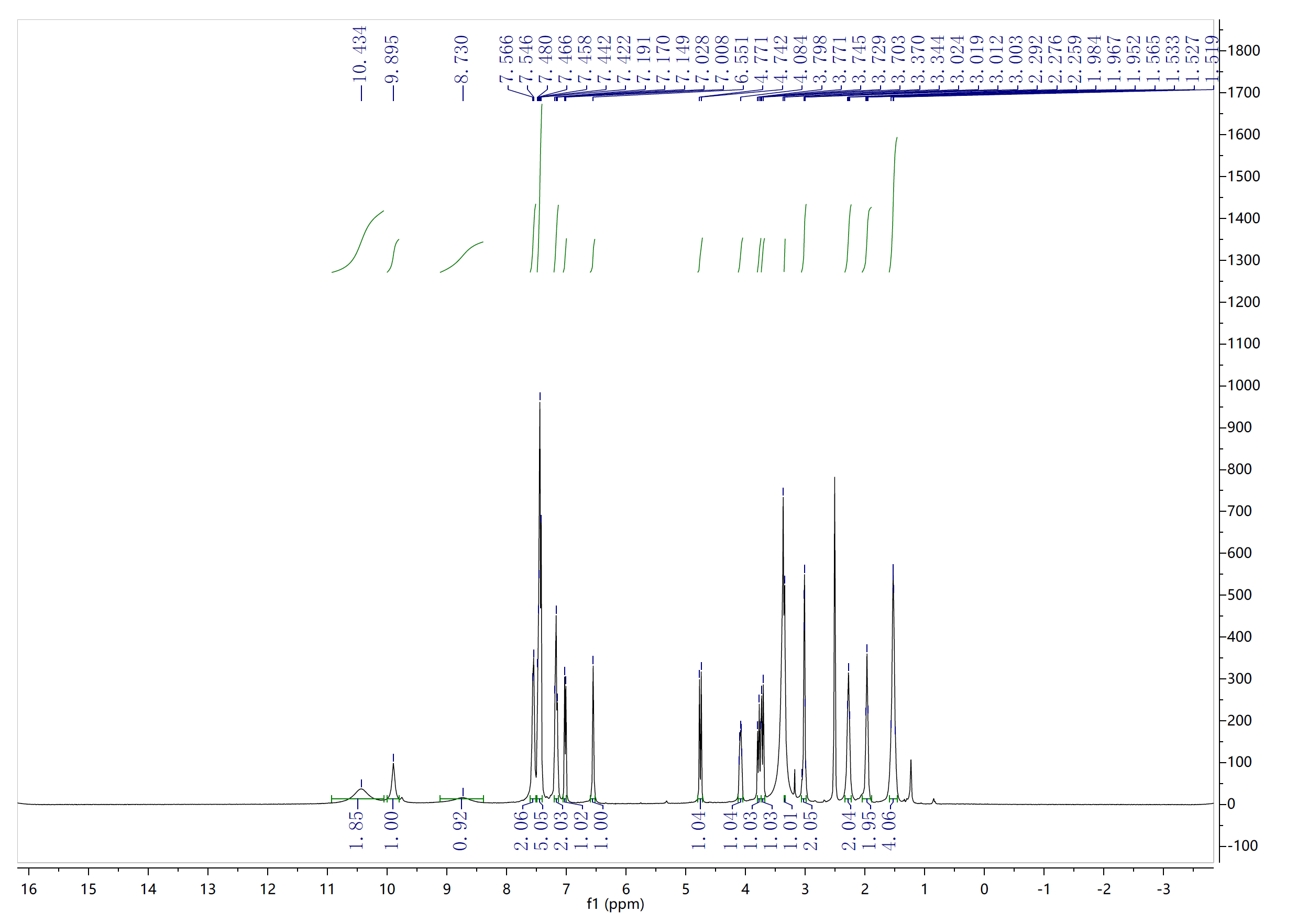


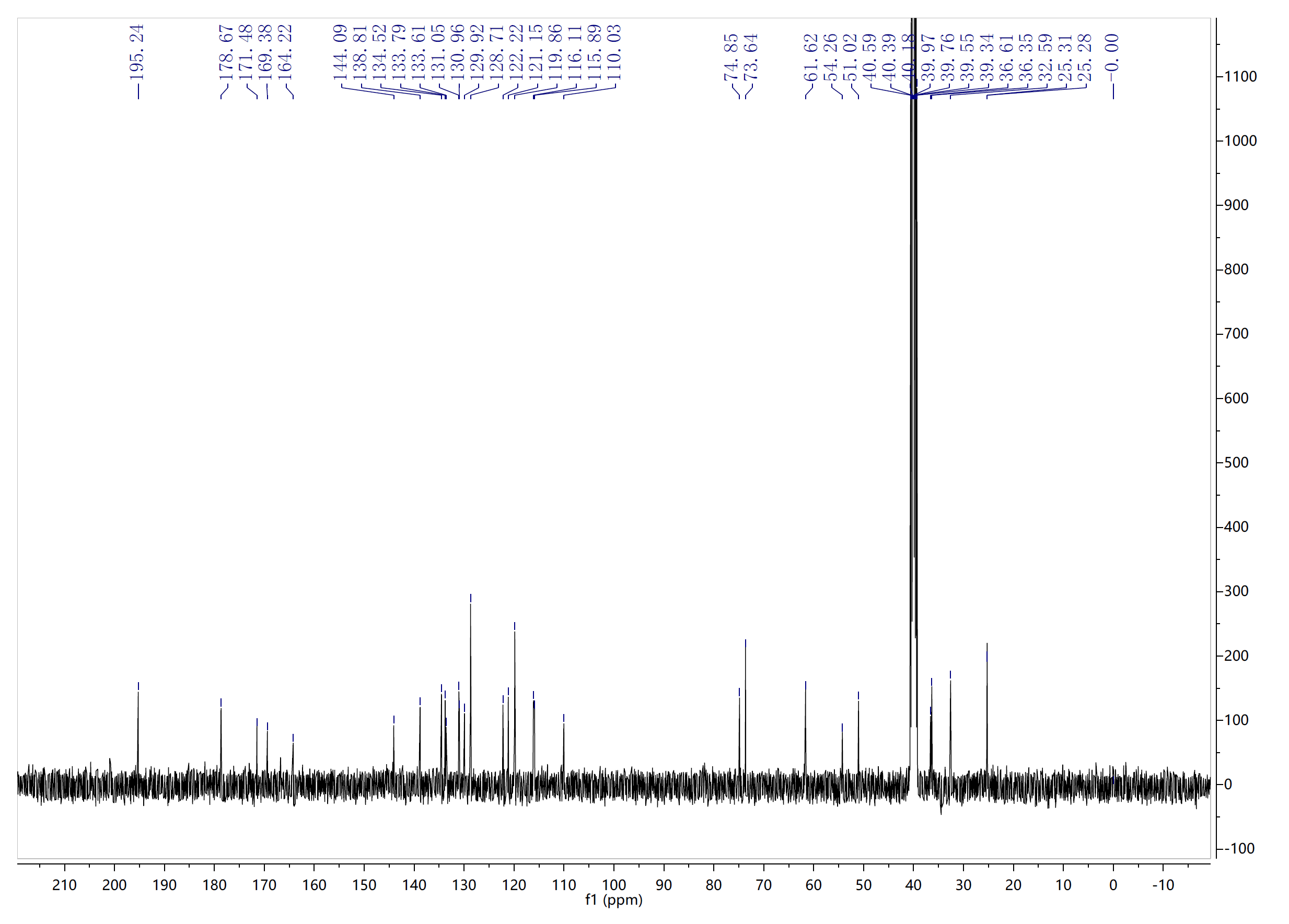


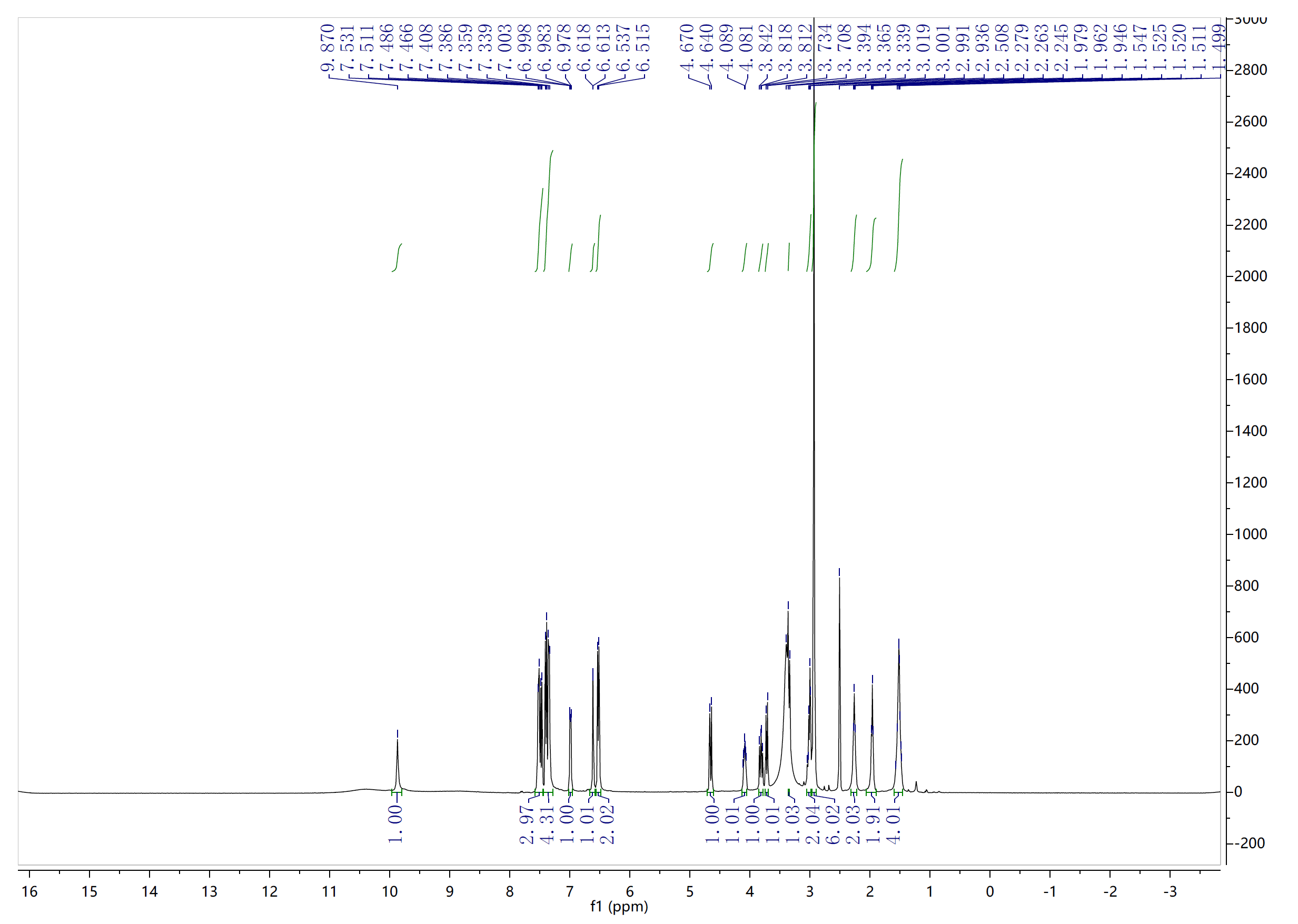


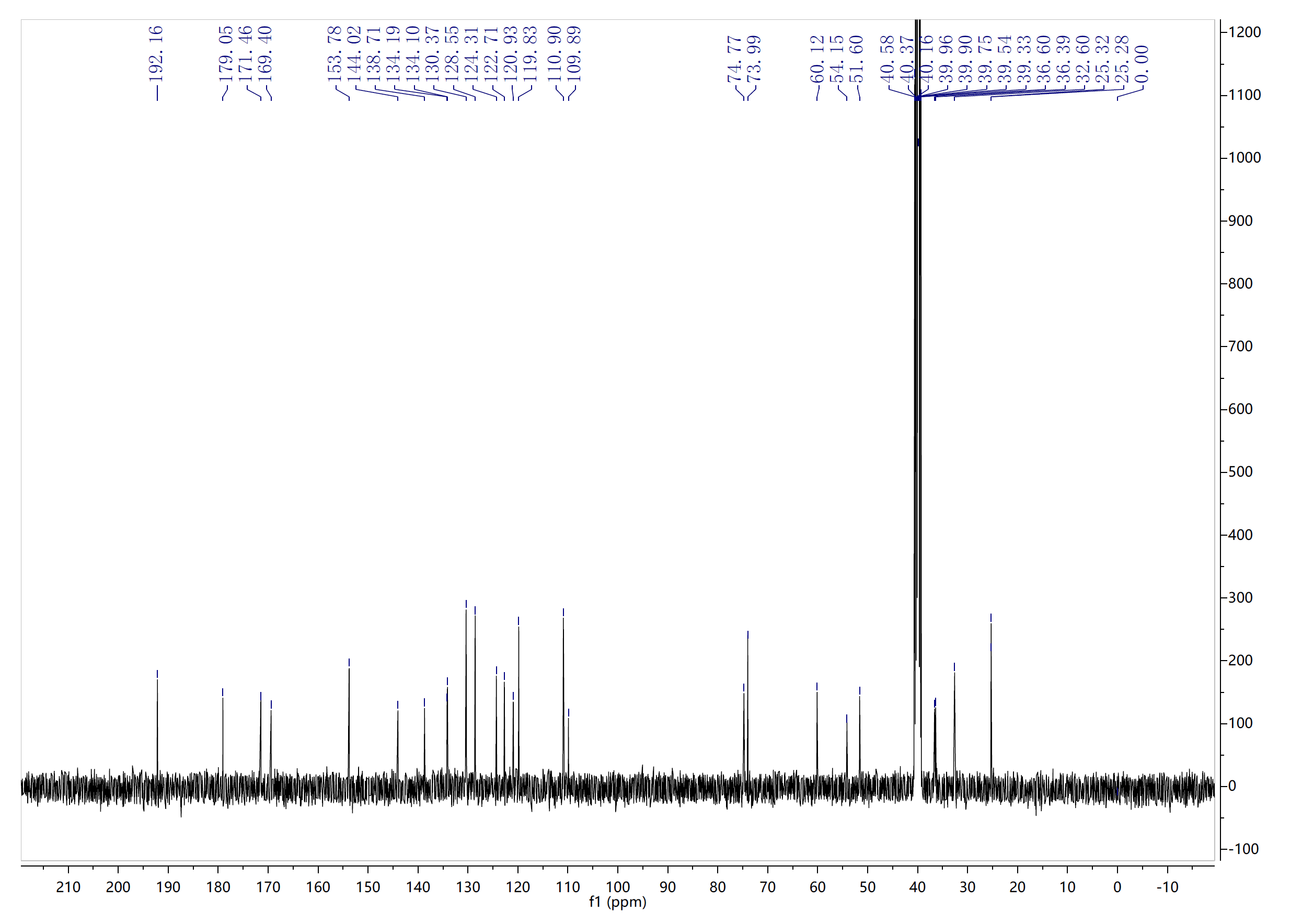


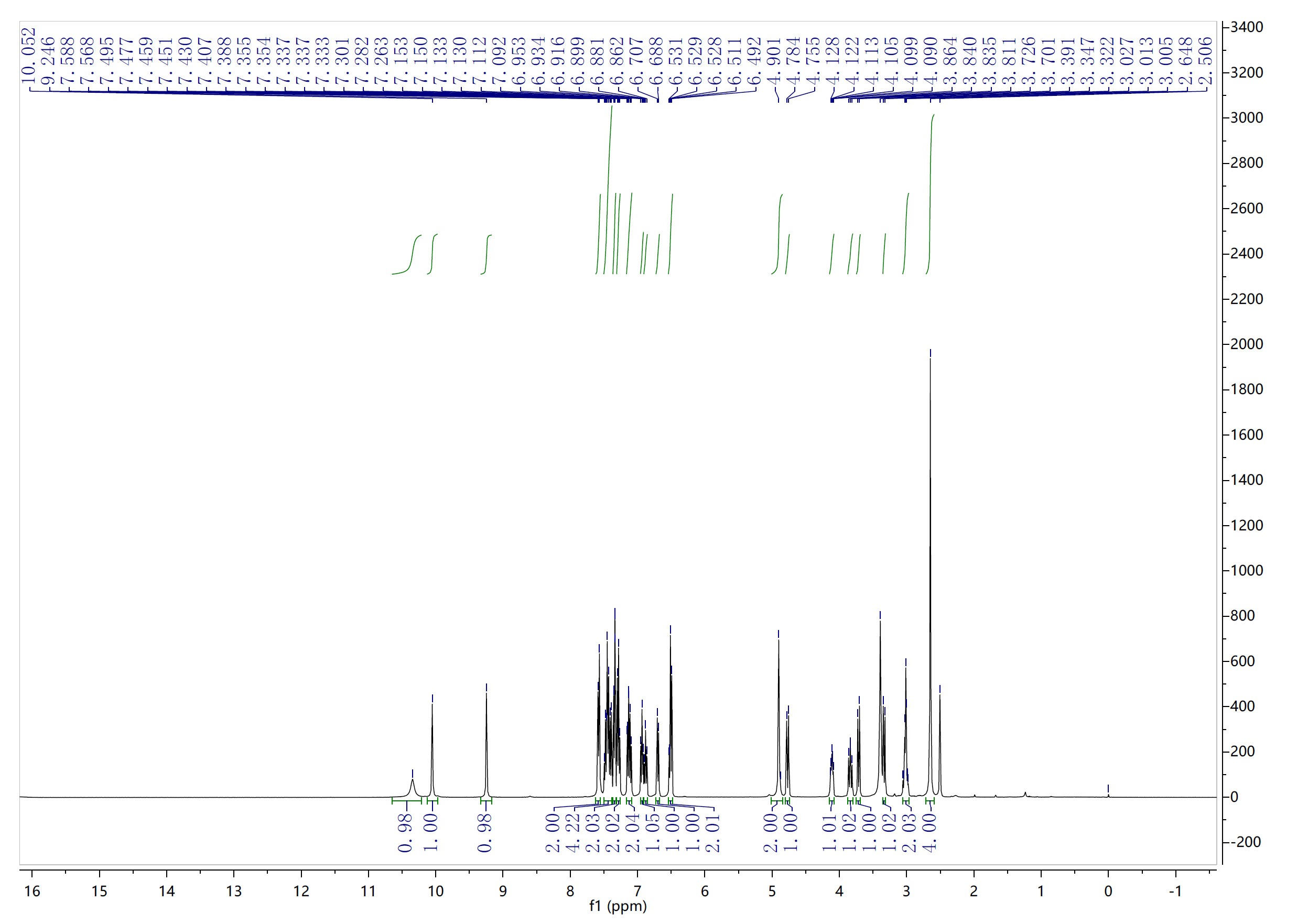


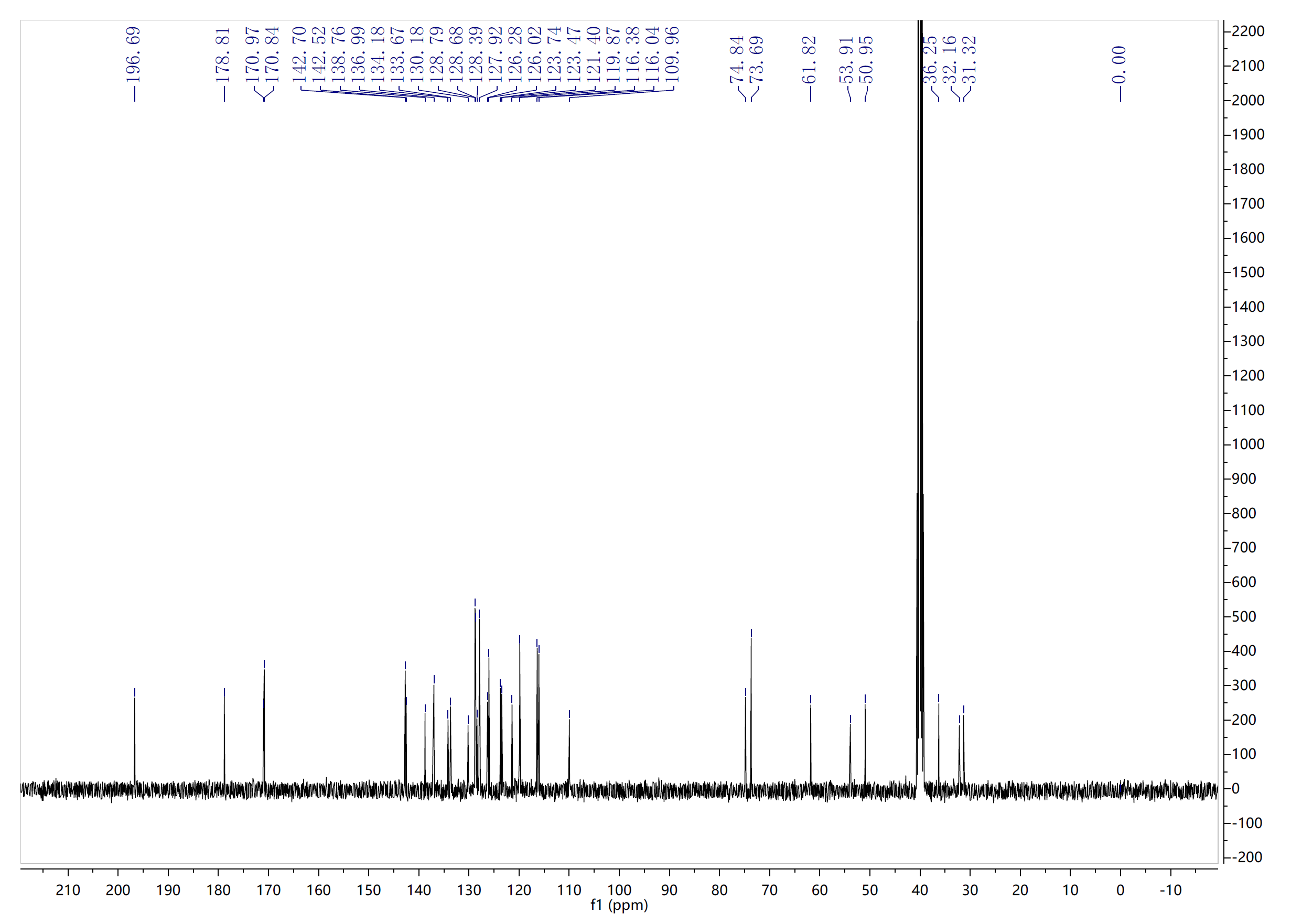


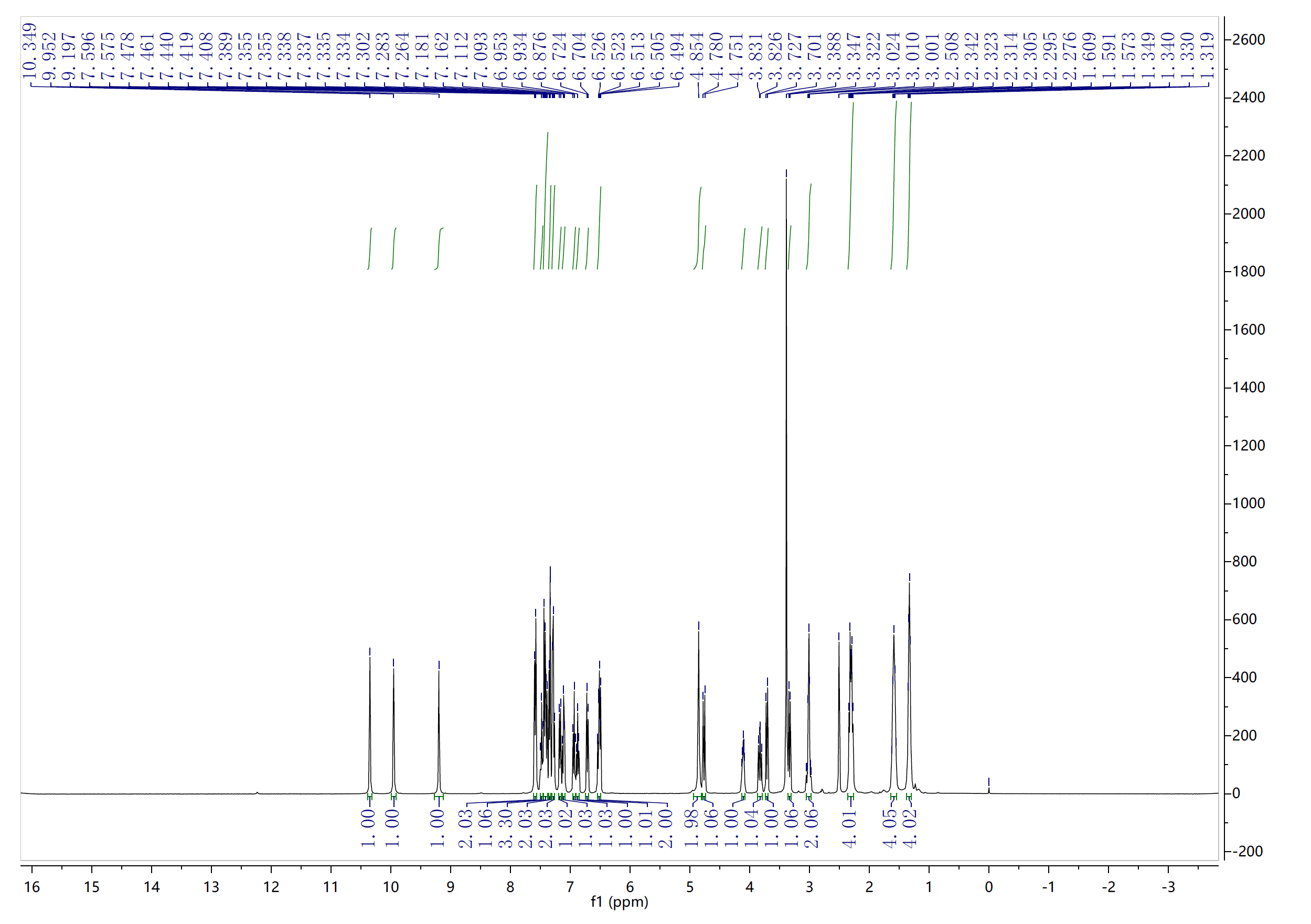


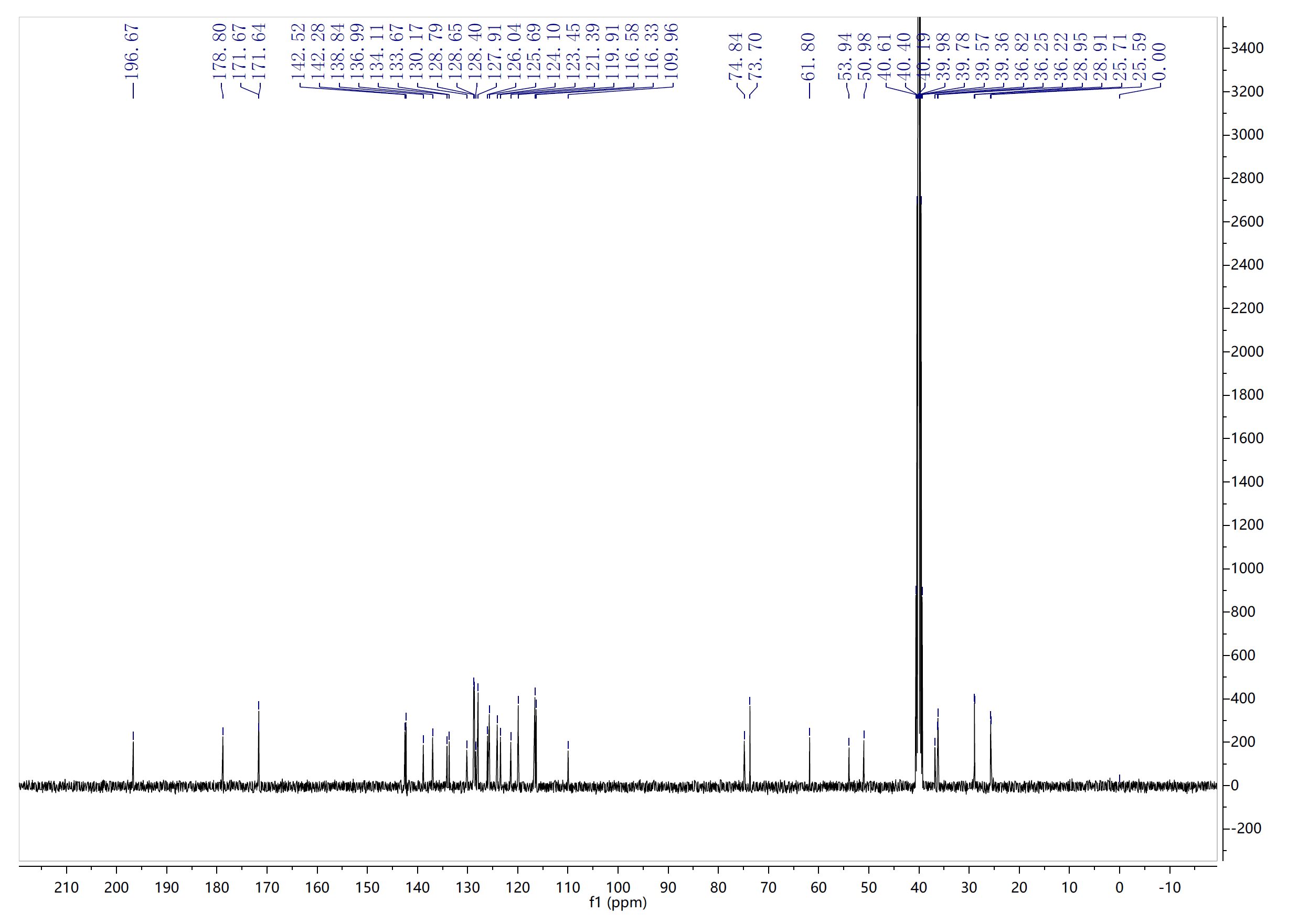


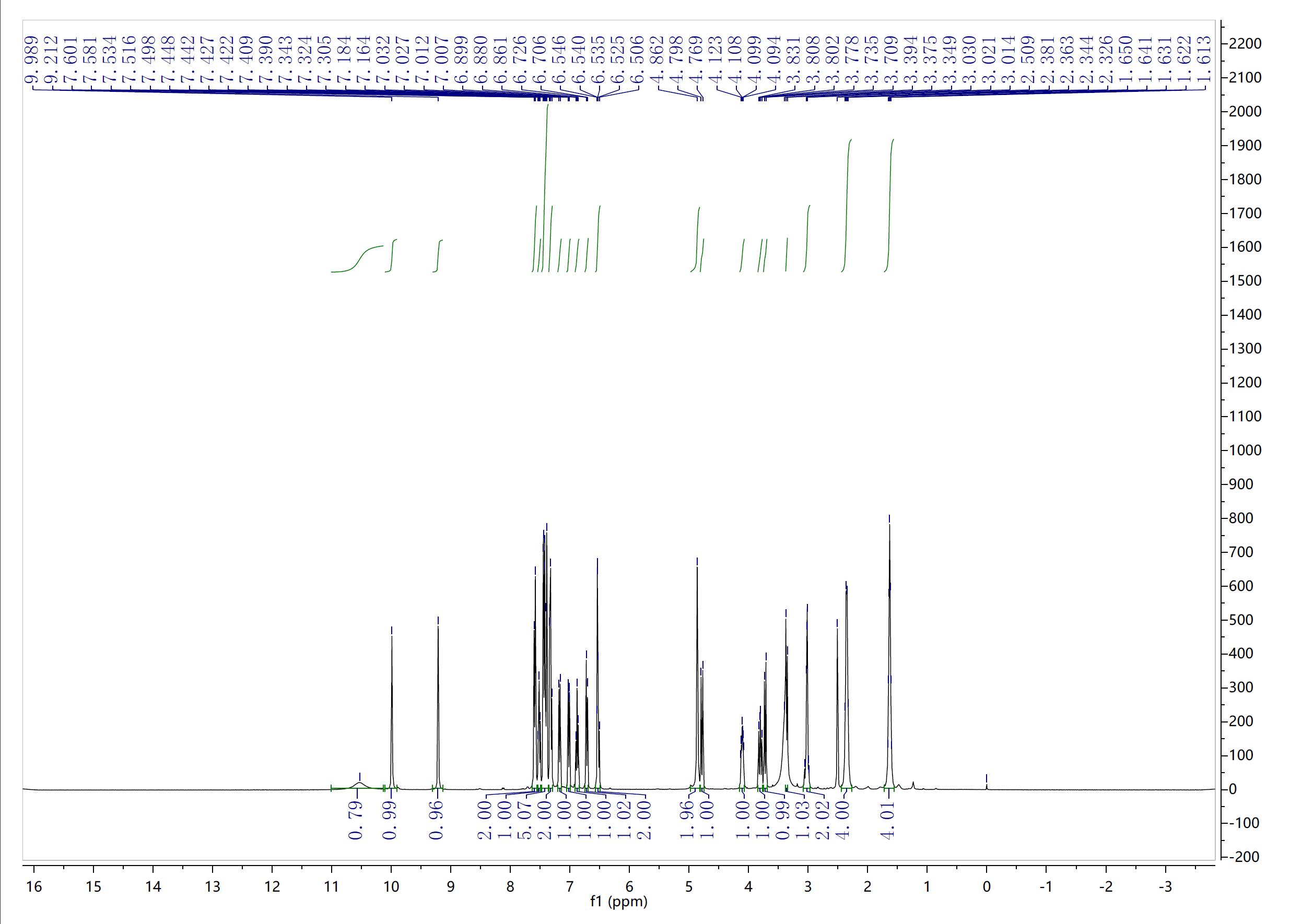


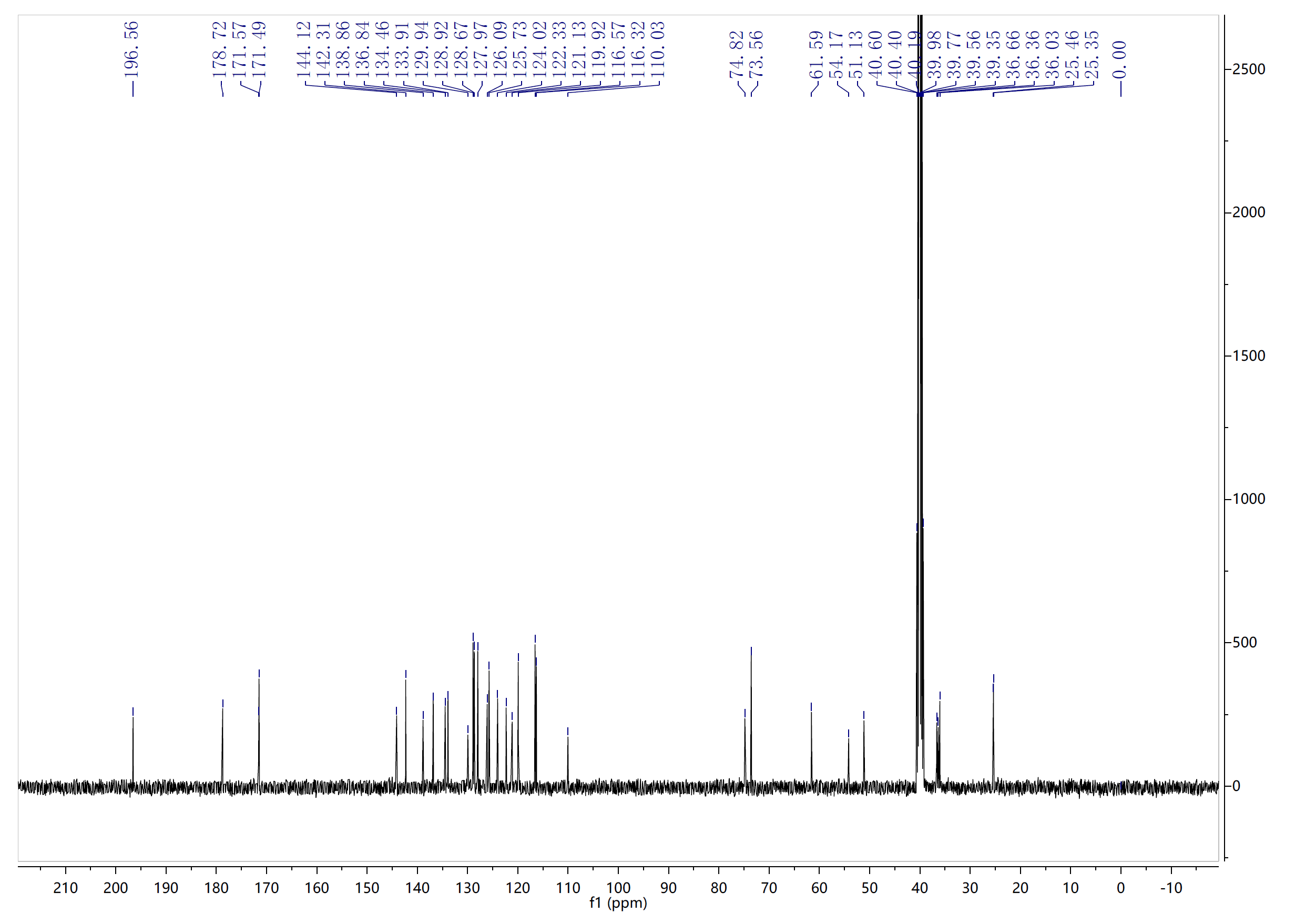


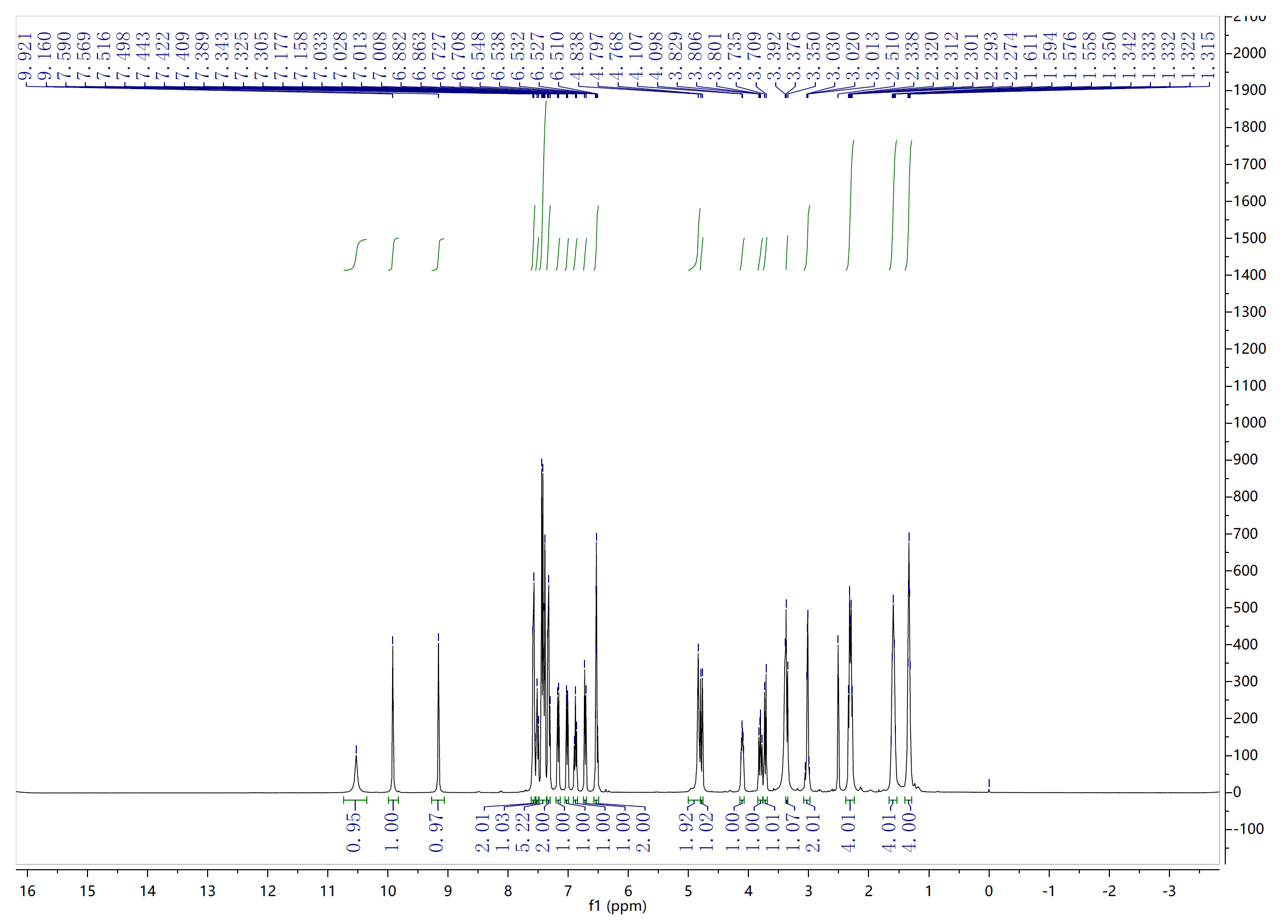


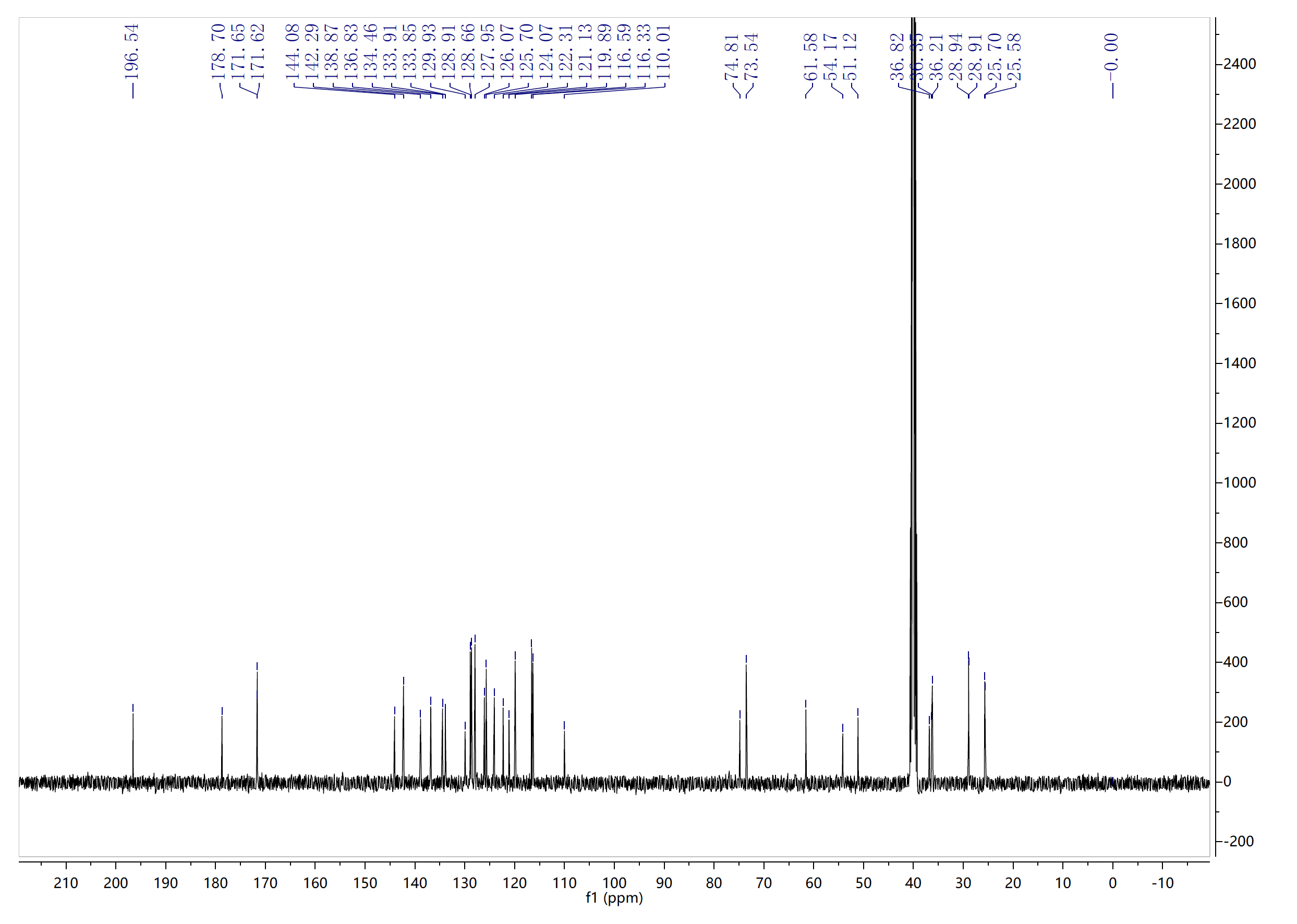


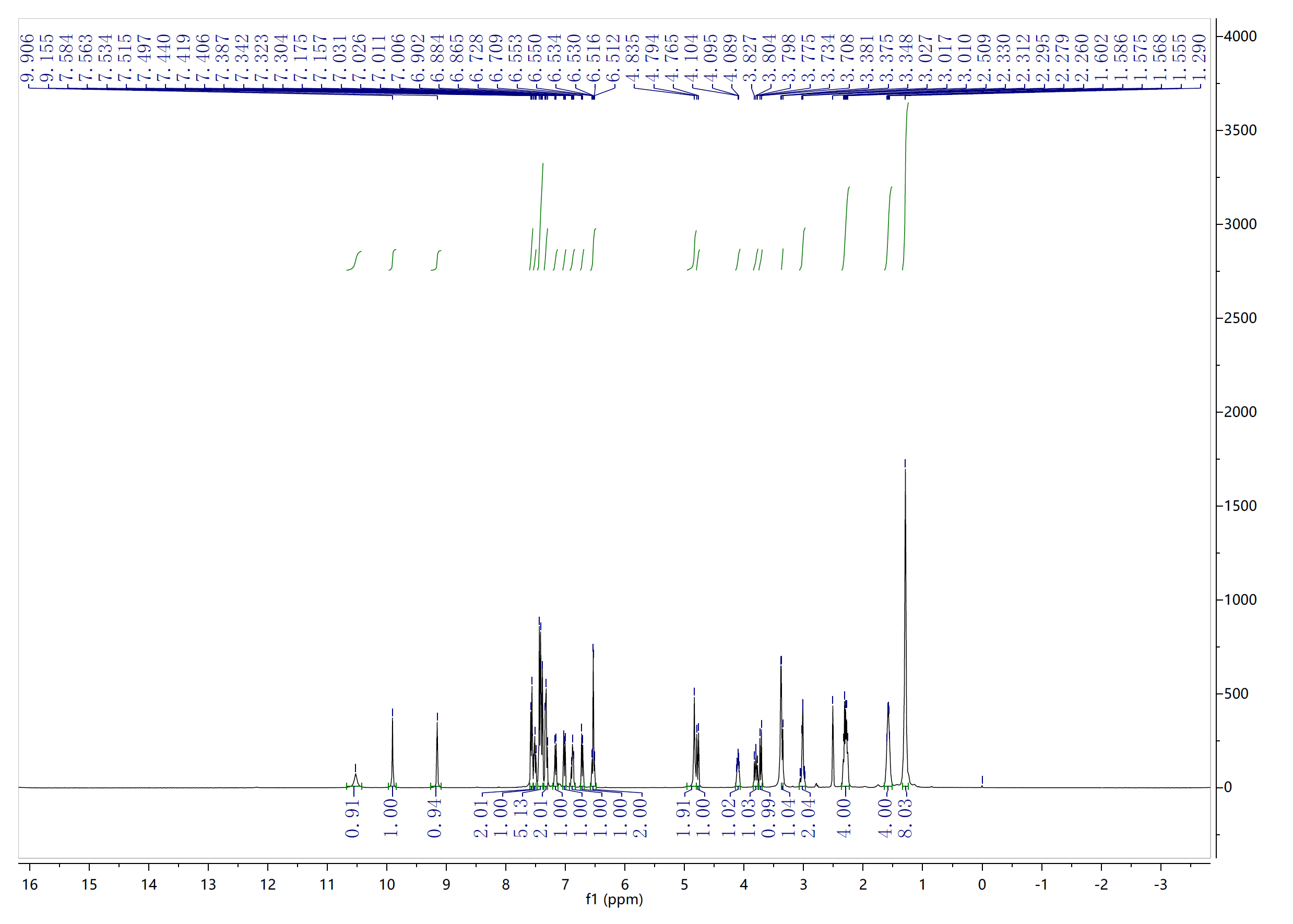


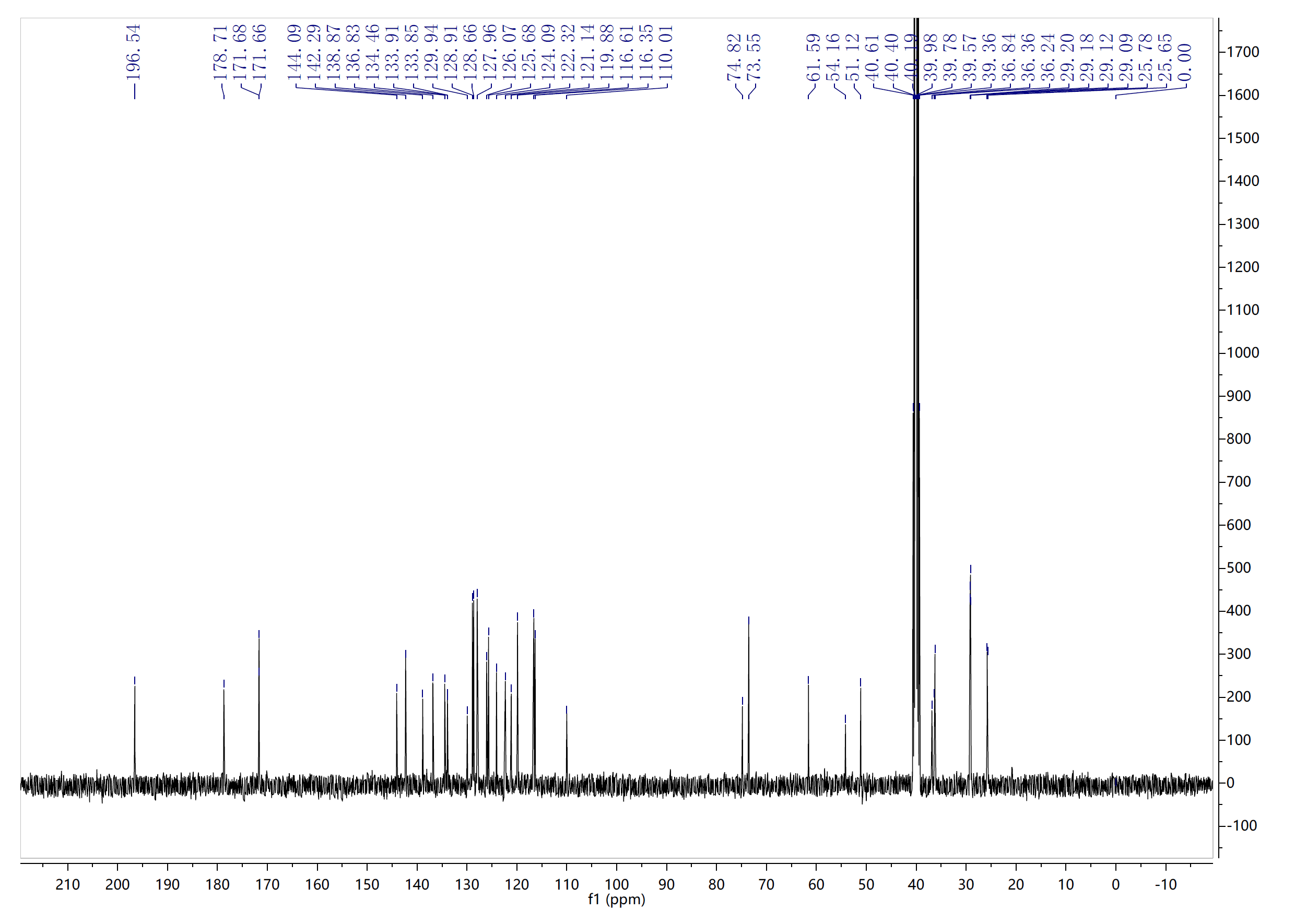


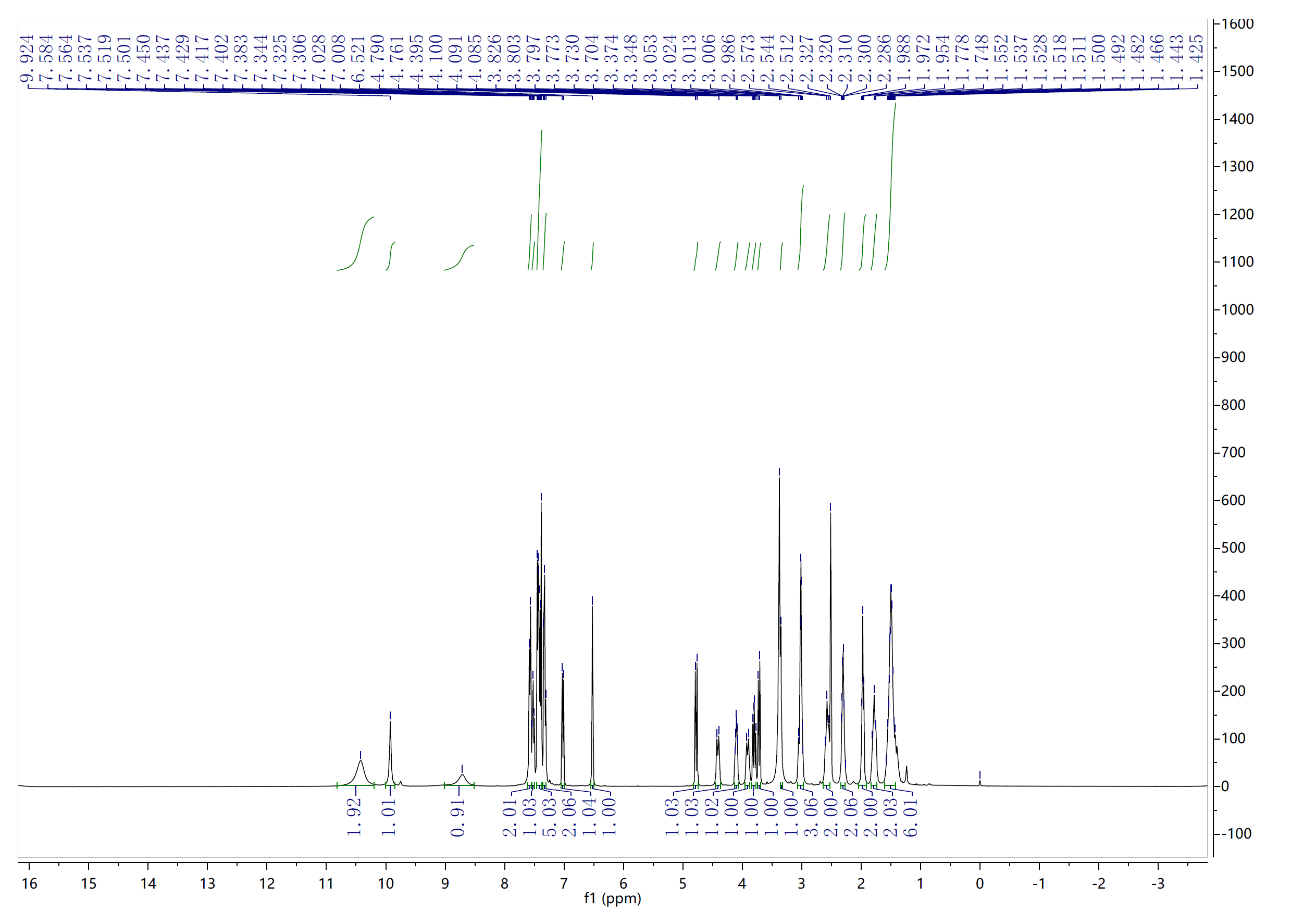


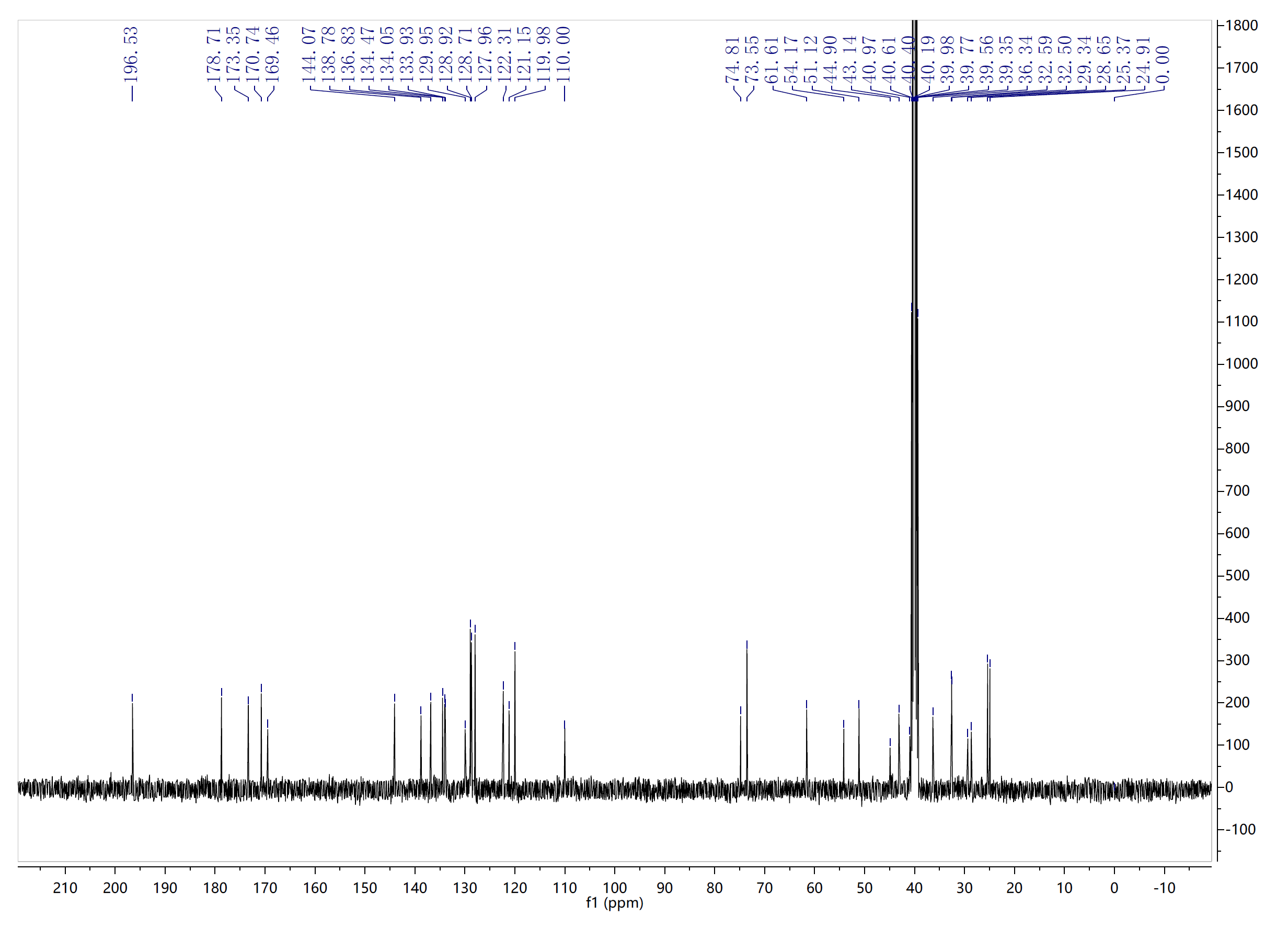


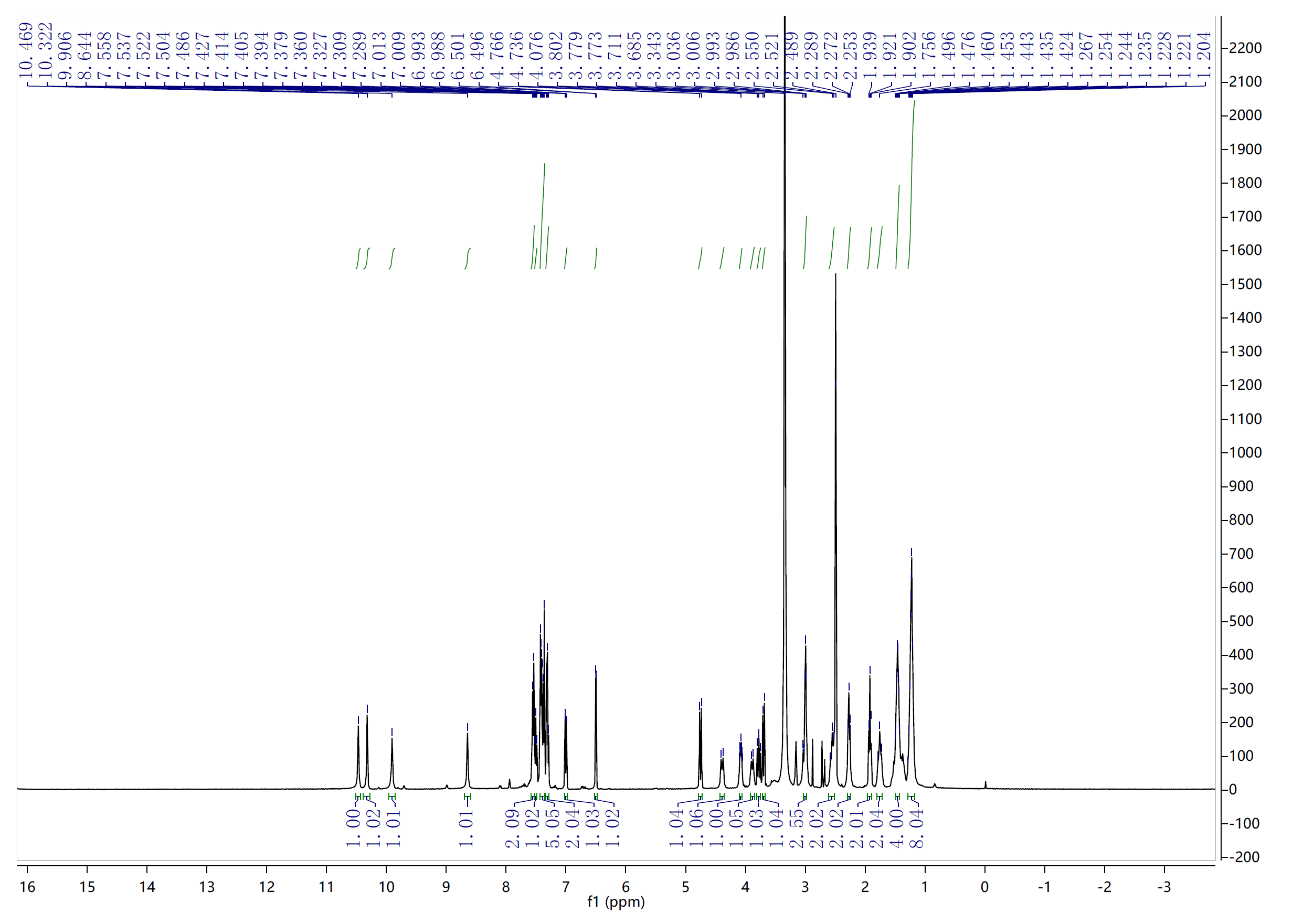


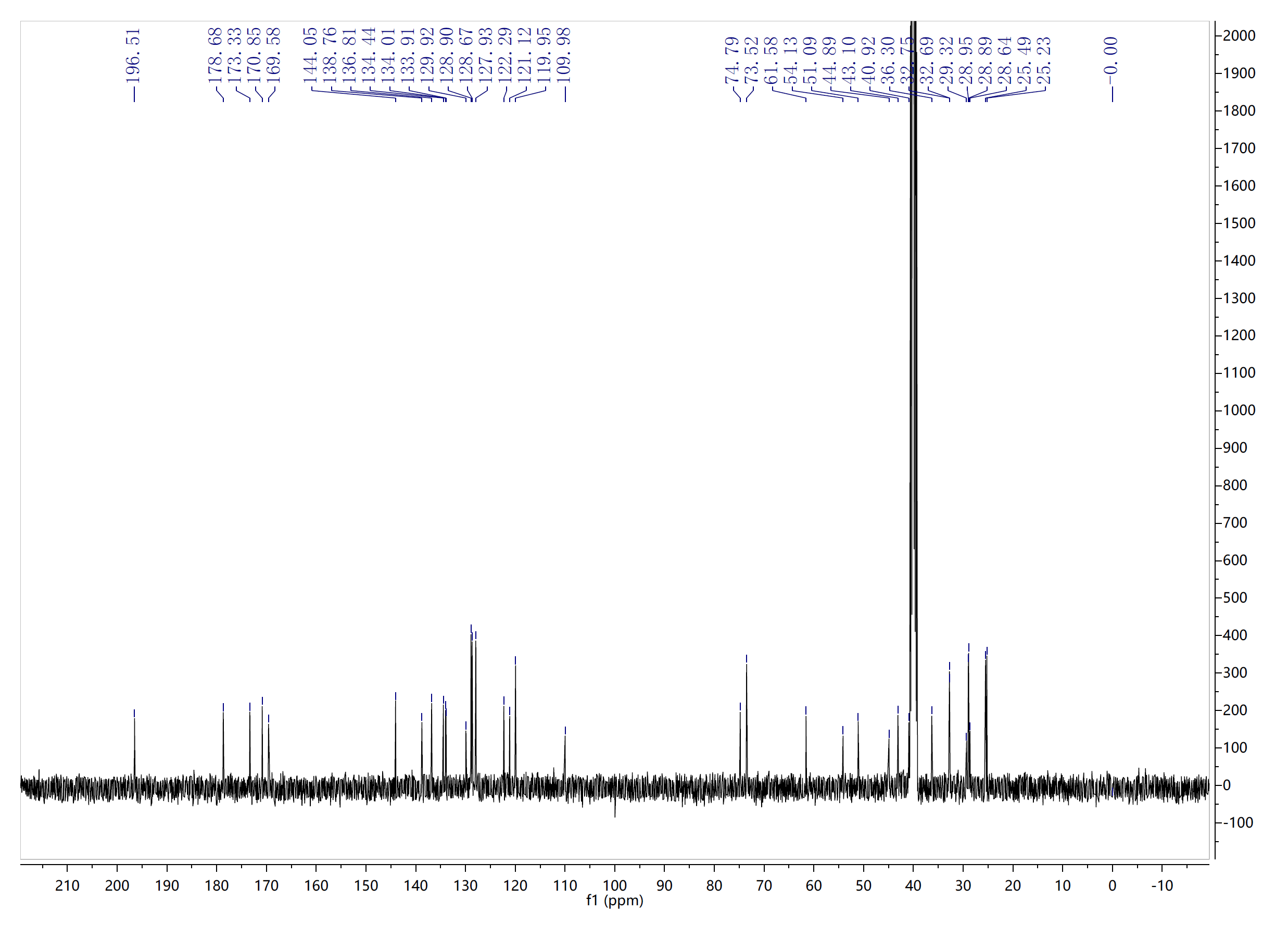


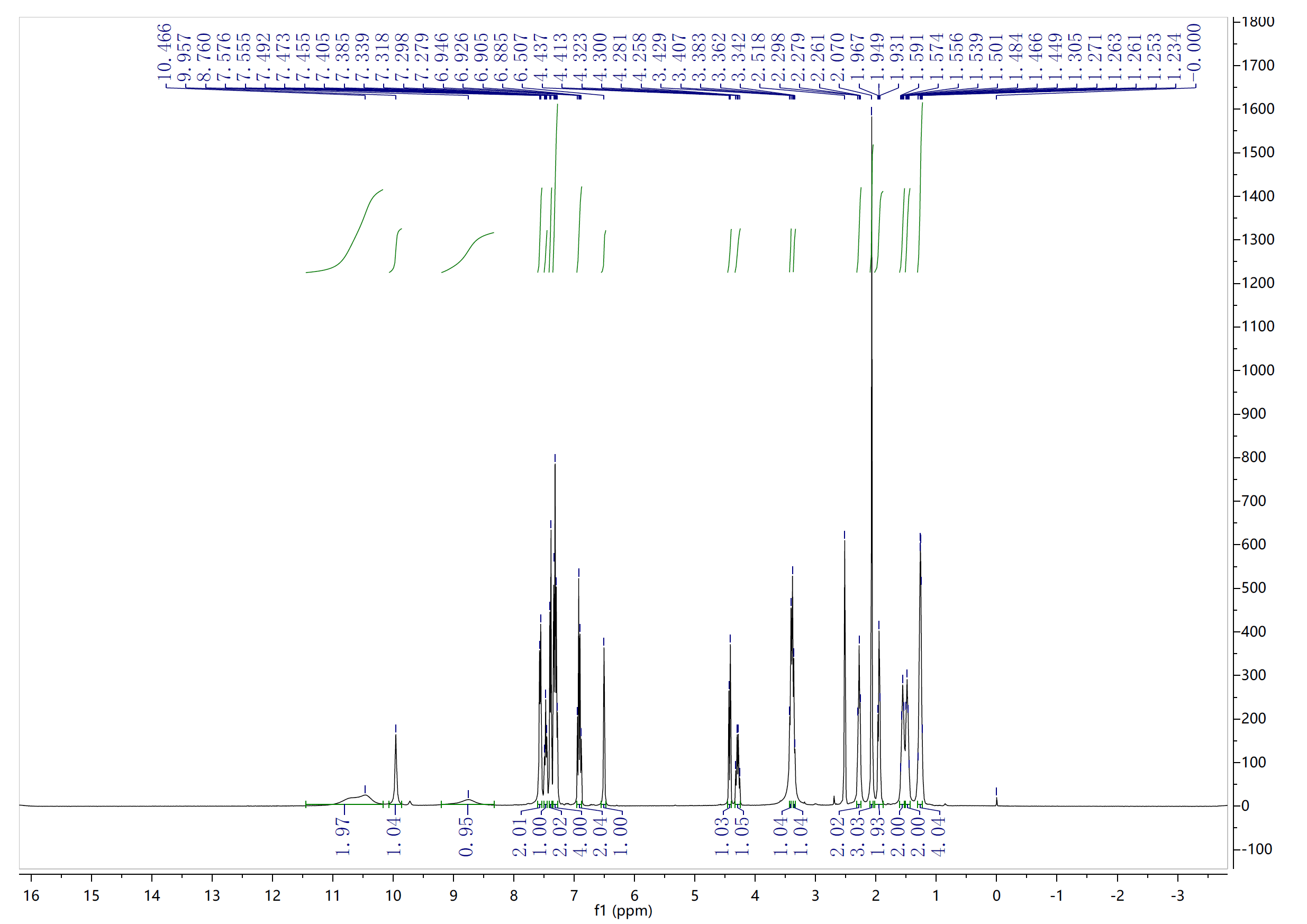


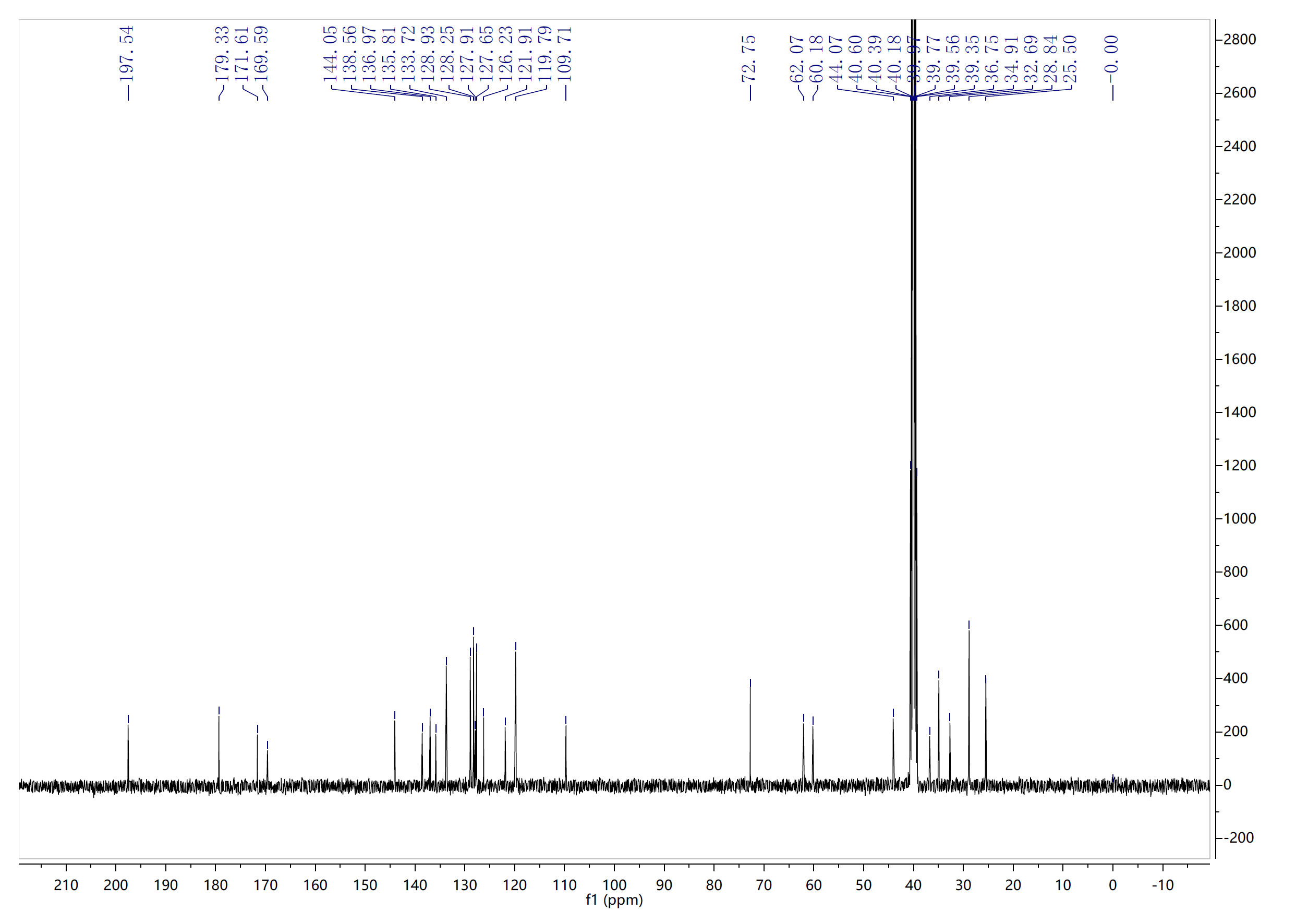


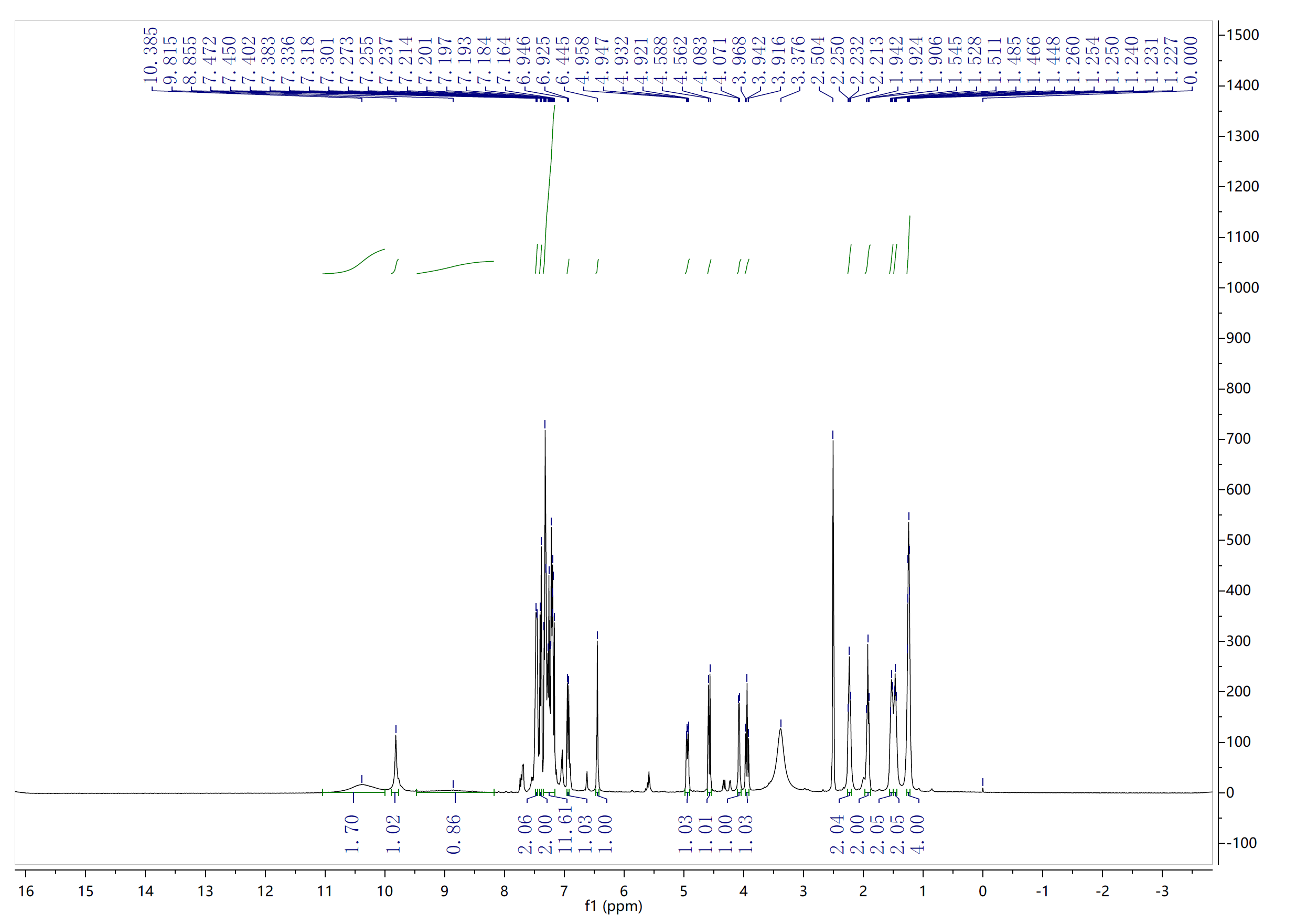


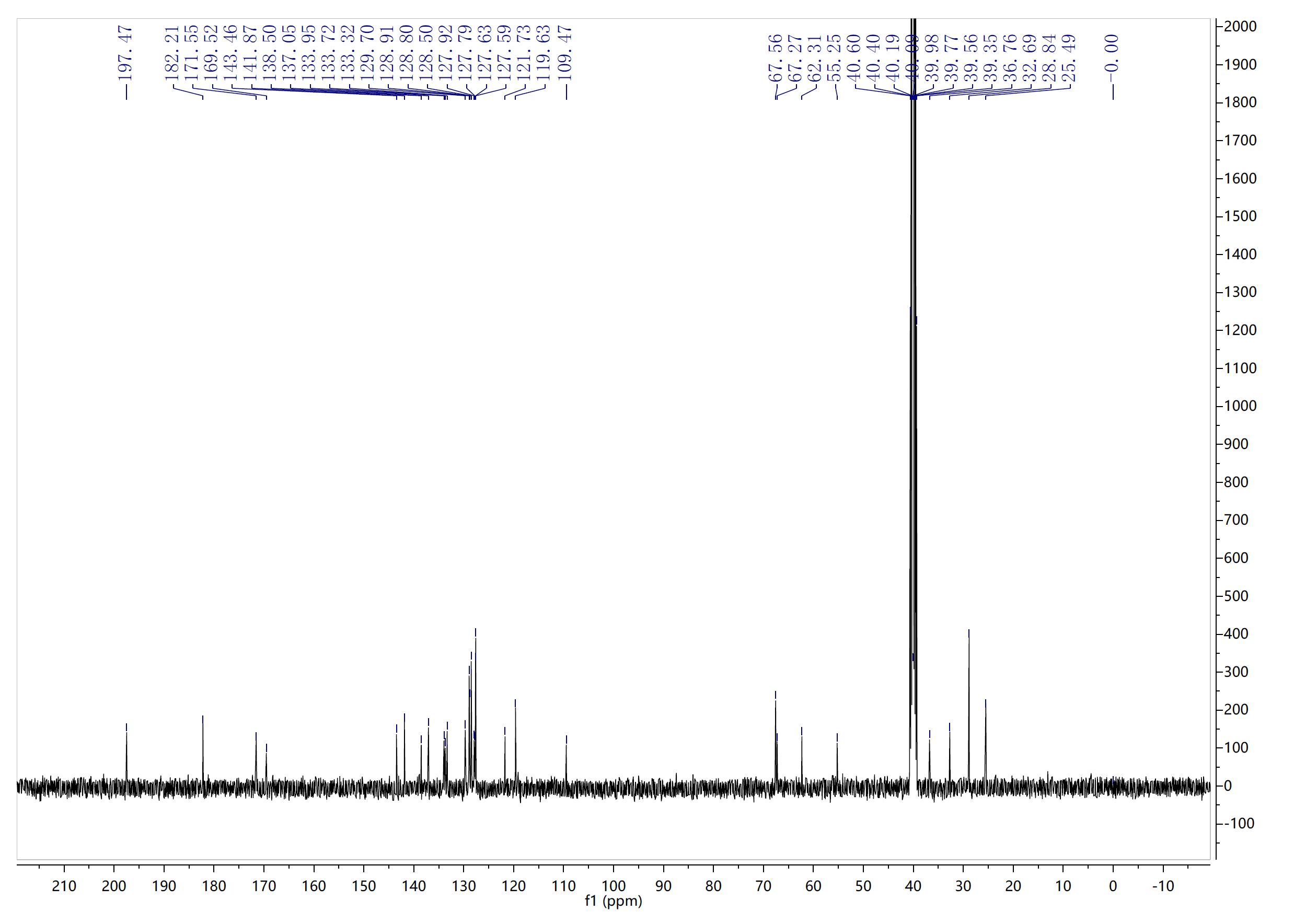


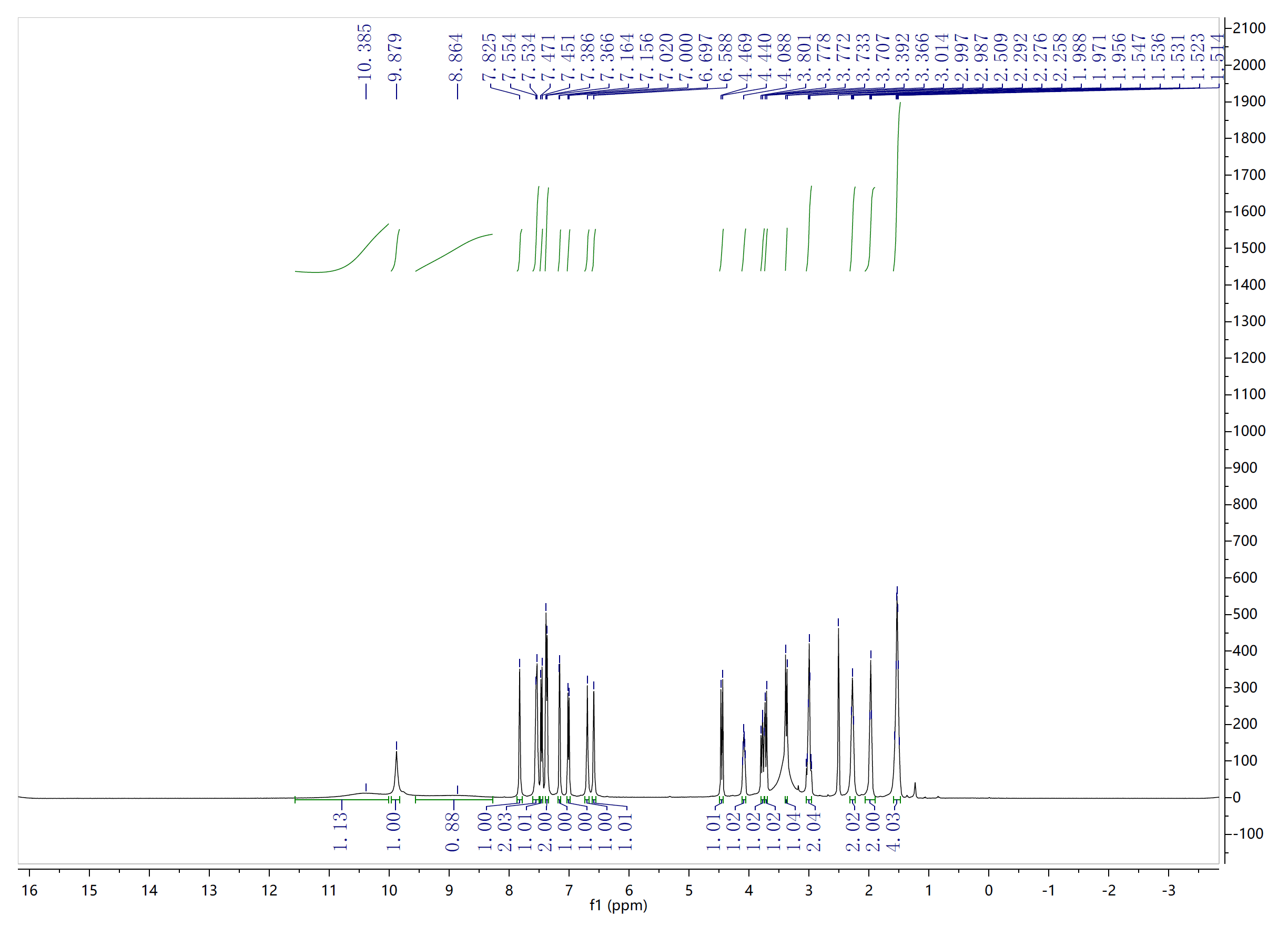


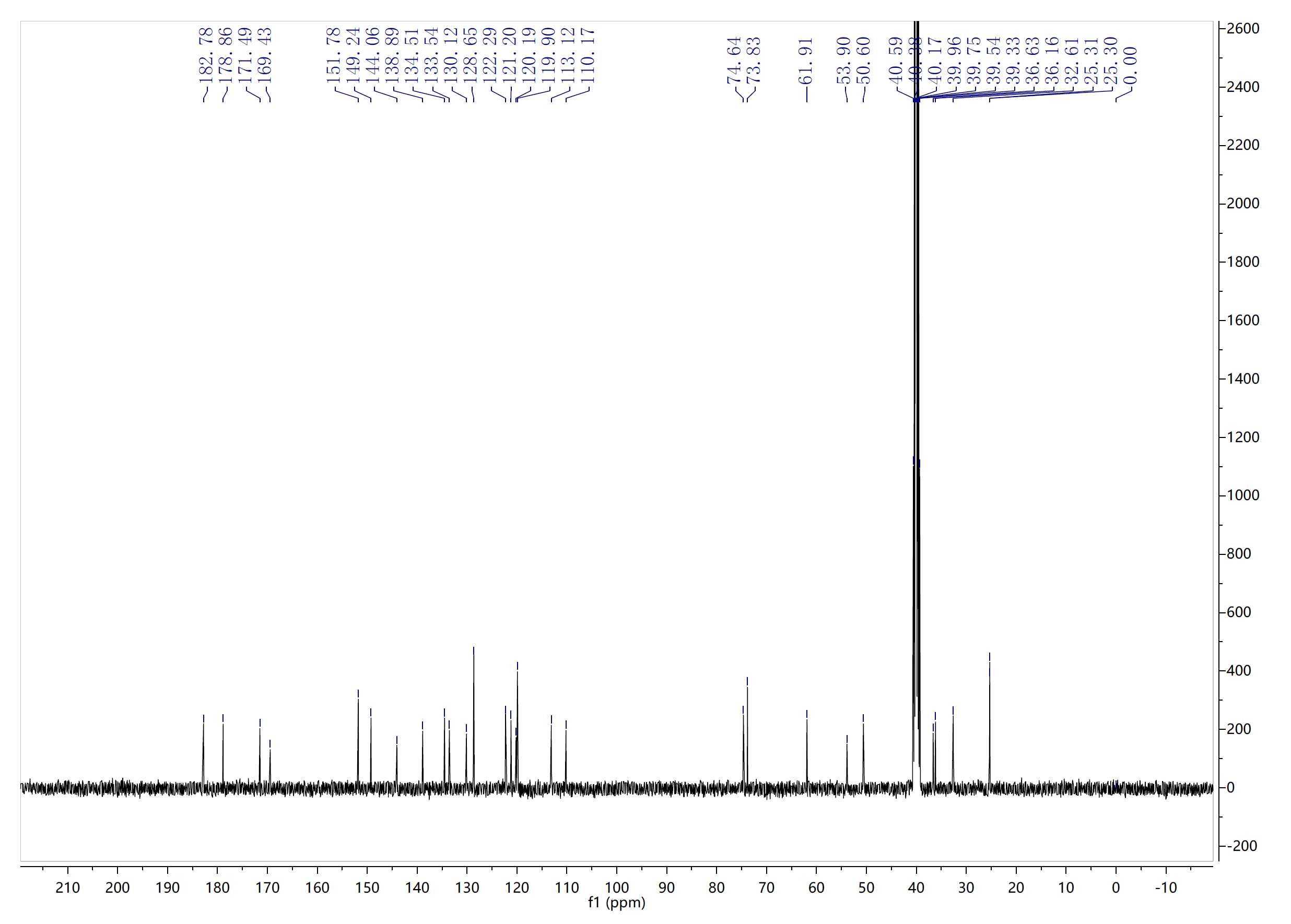


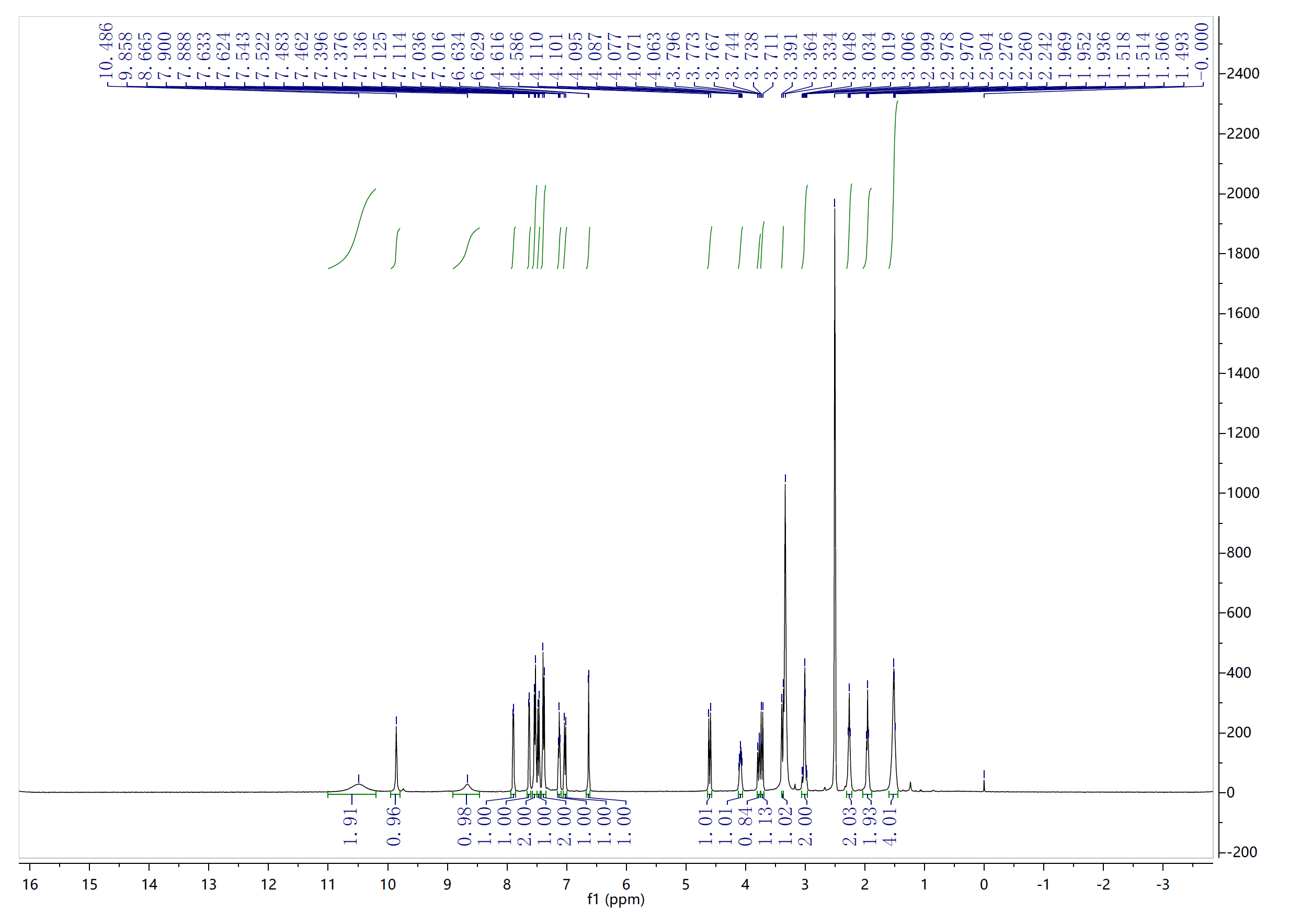


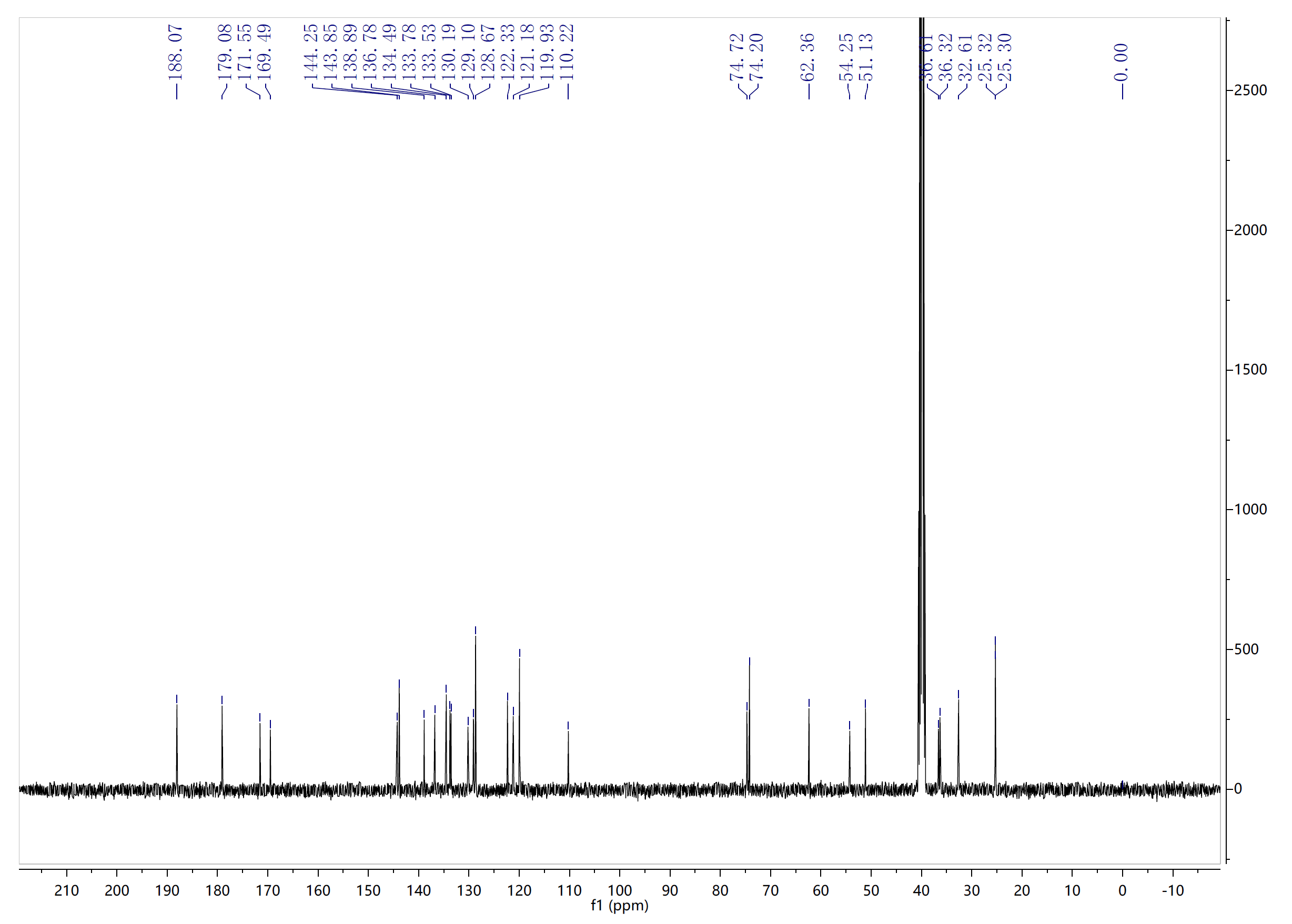

Supplement: Supplementary file 1 [file DataSheet_1.docx]
